# Cross-talk of four types of RNA modification proteins with adenosine reveals the landscape of multivariate prognostic patterns in breast cancer

**DOI:** 10.3389/fgene.2022.943378

**Published:** 2022-09-02

**Authors:** Xuliren Wang, Fangdie Ye, Min Xiong, Bingqiu Xiu, Weiru Chi, Qi Zhang, Jingyan Xue, Ming Chen, Liyi Zhang, Jiong Wu, Yayun Chi

**Affiliations:** ^1^ Department of Breast Surgery, Key Laboratory of Breast Cancer in Shanghai, Fudan University Shanghai Cancer Center, Shanghai, China; ^2^ Department of Urology, Huashan Hospital, Fudan University, Shanghai, China; ^3^ Collaborative Innovation Center for Cancer Medicine, Shanghai, China

**Keywords:** RNA-modifying proteins, breast cancer, risk score, mutation burden, stemness score, immune infiltration, drug sensitivity, prognosis

## Abstract

**Background:** Breast cancer (BC) is the most common malignant tumour, and its heterogeneity is one of its major characteristics. N6-methyladenosine (m6A), N1-methyladenosine (m1A), alternative polyadenylation (APA), and adenosine-to-inosine (A-to-I) RNA editing constitute the four most common adenosine-associated RNA modifications and represent the most typical and critical forms of epigenetic regulation contributing to the immunoinflammatory response, tumorigenesis and tumour heterogeneity. However, the cross-talk and potential combined profiles of these RNA-modified proteins (RMPs) in multivariate prognostic patterns of BC remain unknown.

**Methods:** A total of 48 published RMPs were analysed and found to display significant expression alterations and genomic mutation rates between tumour and normal tissues in the TCGA-BRCA cohort. Data from 4188 BC patients with clinical outcomes were downloaded from the Gene Expression Omnibus (GEO), the Cancer Genome Atlas (TCGA), and the Molecular Taxonomy of Breast Cancer International Consortium (METABRIC), normalized and merged into one cohort. The prognostic value and interconnections of these RMPs were also studied. The four prognosis-related genes (PRGs) with the greatest prognostic value were then selected to construct diverse RMP-associated prognostic models through univariate Cox (uniCox) regression analysis, differential expression analysis, Least absolute shrinkage and selection operator (LASSO) regression and multivariate Cox (multiCox) regression. Alterations in biological functional pathways, genomic mutations, immune infiltrations, RNAss scores and drug sensitivities among different models, as well as their prognostic value, were then explored.

**Results:** Utilizing a large number of samples and a comprehensive set of genes contributing to adenosine-associated RNA modification, our study revealed the joint potential bio-functions and underlying features of these diverse RMPs and provided effective models (PRG clusters, gene clusters and the risk model) for predicting the clinical outcomes of BC. The individuals with higher risk scores showed poor prognoses, cell cycle function enrichment, upregulation of stemness scores, higher tumour mutation burdens (TMBs), immune activation and specific drug resistance. This work highlights the significance of comprehensively examining post-transcriptional RNA modification genes.

**Conclusion:** Here, we designed and verified an advanced forecasting model to reveal the underlying links between BC and RMPs and precisely predict the clinical outcomes of multivariate prognostic patterns for individuals.

## Background

In females, Breast cancer (BC) has overtaken lung cancer as the most commonly diagnosed cancer and is showing continuous acceleration, but with stagnated research progress (Sung et al., 2021; Siegel et al., 2022). Epigenetic changes, defined as stable alterations in transcription or translation without potential modifications in the genetic sequence, play a crucial role in both physiological and pathological processes (Taby and Issa, 2010). In the recent past, an accumulating number of investigations have revealed that RNA modification is an epigenetic regulatory mechanism of the expression of tumorigenesis-related genes and the inflammatory response, and targeting RNA modification enzymes represents a promising anticancer therapy (Frye et al., 2018; Yankova et al., 2021; Qiu et al., 2022). In BC, RMPs have been verified to function in tumour progression and metastasis (Chang et al., 2020).

More than 170 types of RNA modifications have been detected in nature, are widespread among all nucleotides, including A, U, C and G (Roundtree et al., 2017), and can be divided into three specific modification groups: “writers”, “erasers” and “readers” (Barbieri and Kouzarides, 2020). Among them, adenosine most commonly shows alteration, and diverse modifications with adenosine may compensate for each other and form a competitive link (Xiang et al., 2018). Therefore, to explore the underlying mechanism and links, we focused on adenosine-related RNA modifications, including N6-methyladenosine (m6A), N1-methyladenosine (m1A), alternative polyadenylation (APA), and adenosine-to-inosine (A-to-I) RNA editing.

We identified genes with these four types of RNA modification from published articles. m6A is the most typical epigenetic RNA modification type in the eukaryotic transcriptome, affecting RNA metabolism in almost every process, and is catalysed by the m6A “writers”, namely, METTL3/14/15/16, CBLL1, ZC3H13, RBM15/15B, KIAA1429 and WTAP; m6A “erasers”, namely, FTO and ALKBH5; and m6A “readers”, namely, YTHDF1/2/3, YTHDC1/2, ELAVL1, FMR1, HNRNPA2B1, HNRNPC, IGF2BP1/2/3, LRPPRC, RBMX and EIF3A (Meyer et al., 2015; Meyer and Jaffrey, 2017; Choe et al., 2018; Zaccara et al., 2019; Jiang et al., 2021; Xu et al., 2021; Ye et al., 2021).

m1A is widely present at the internal sites of mRNA as a form of posttranscriptional modification. m1A “writers” include TRMT61 A/B, TRMT10C and TRMT6; m1A “erasers” include ALKBH1/3 (Liu et al., 2016; Esteve-Puig et al., 2021).

APA creates specific new 3' ends on mRNAs and other RNA polymerase II transcripts, which is widely present in all eukaryotic species and is considered to be a mechanism for the creation of protein isomers. APA “writers” include CPSF1/2/3/4, CSTF1/2/3, CFI, PCF11, CLP1, NUDT21, and PABPN1 (Elkon et al., 2013; Tian and Manley, 2017).

RNA editing induces non-synonymous substitutions in protein-coding sequences. A-to-I editing by the double-stranded RNA-specific adenosine deaminase (ADAR) enzyme is the most common type. A-to-I “writers” include ADAR, ADARB1, and ADARB2 (Eisenberg and Levanon, 2018; Ishizuka et al., 2019; Chen et al., 2021).

To fully understand the behaviour of these adenosine-related RMPs, we obtained their mutation and expression profiles in BC and established a network to investigate the mechanisms underlying neoplasm progression.

Studies have established a direct link between m6A and dynamic chromatin modification and identified underlying mechanisms for collaborative transcriptional interactions between RNA modification and histone modification (Li et al., 2020).

The underlying links between m6A and tumour immune activation has already been described. Loss of the m6A “writer” protein METTL3 disrupts T-cell homeostasis and differentiation (Li et al., 2017). The loss of FTO overcomes hypomethylation-induced immune escape, sensitizes leukaemia cells to T-cell cytotoxicity, and plays a key role in cancer stem cell self-renewal (Su et al., 2020). Deletion of YTHDF1 enhances the CD8^+^ T-cell antitumor response and elevates the benefit of PD-L1 checkpoint blockade (Han et al., 2019). Since interferons are critical for inhibiting infectious and malignant diseases, ADAR serves as a target for cancer immunotherapy (Chung et al., 2018; Herbert, 2019; Liu et al., 2019). Accordingly, how the regulatory network and underlying links of RMPs influence tumour immunoregulation should be further explored.

During tumour development, cells that dedifferentiate and exhibit a stem cell-like phenotype have a higher degree of malignancy. Through maintaining FOXM1 expression and cell proliferation, ALKBH5 sustains tumorigenicity of stem-like cells in glioblastoma (Zhang et al., 2017). This suggests the potential role of RMPs in tumour cell proliferation and dedifferentiation.

Our study included 4188 BC patients from the Gene Expression Omnibus (GEO), the Cancer Genome Atlas (TCGA), and the Molecular Taxonomy of Breast Cancer International Consortium (METABRIC) databases in total. We revealed the genes with the most distinctive predictive value for RMPs, explored the associated genes, and constructed a risk model which can be utilized to assess the risk score of individuals. We discovered that diverse patterns of adenosine-associated RNA modification were linked not only to the infiltration of immune cells but also to the cell cycle, RNAss score, drug resistance and, most significantly, patients’ clinical outcome.

## Materials and methods

### Data acquisition and processing

From the GEO database (https://www.ncbi.nlm.nih.gov/geo/), the METABRIC database (http://www.cbioportal.org/) and TCGA database (https://portal.gdc.cancer.gov/), we obtained the transcriptome data and overall survival (OS) data of BC patients retrospectively. 4188 BC patients with a follow-up time >30 days were selected for our analysis, including samples from seven GEO datasets (GSE131769, GSE162228, GSE20685, GSE20713, GSE24450, GSE42568, and GSE48391, *n* = 1,193), the METABRIC database (*n* = 1,904) and TCGA database (*n* = 1,091). All data were normalized with log2 transformation of fragments per kilobase of exon per million mapped fragments (FPKM) values and subsequently merged into one dataset with the “ComBat” algorithm of the SVA Package. Half of the 4188 BC patients were randomly assigned to a training cohort (*n* = 2,094), the remaining cases were defined as the testing cohort (*n* = 2,094), and the entire dataset was used to generate internal validation cohorts. GSE3494 (*n* = 234) from the GEO dataset was chosen as an external validation cohort. The RNA expression profiles of BC tissues (*n* = 1,109) and normal breast tissues (*n* = 113), somatic mutation data, somatic copy number variation (CNV) data and stemness score data were all obtained from TCGA database. The clinicopathological information, including age, tumour side, therapy, histology subtype, lymph node state, RFS status and cause of death, was gained from the METABRIC database.

### Unsupervised clustering and gene set variation analysis of prognosis-related gene clusters

The correlation between RMPs and OS was analysed by univariate Cox regression (uniCox), and survival status was displayed with a Kaplan–Meier (K-M) curve using the “survminer” and “survival” R packages with an optimal cut-off value. RMPs with *p* < 0.001 were considered prognostic, and we named them prognosis-related genes (PRGs), which included YTHDF1, EIF3A, PCF11 and CBLL1. An unsupervised clustering algorithm was then applied to the 4188 BC patients based on the expression levels of 4 PRGs to build PRG clusters. The R package “conensusClusterPlus” was utilized with 1,000 repetitions during the above steps. Principal component analysis (PCA) was performed to visualize the independence of each cluster.

### Selection of differentially expressed genes with prognostic value and construction of gene clusters

To identify PRG-related genes that were differentially expressed among subgroups, we used the empirical Bayesian function of the R package “limma” and selected 23 differentially expressed genes (DEGs) among three subgroups. Then, uniCox regression analysis was used and screened out 19 genes from DEGs for further analysis (*p* < 0.05). We named these 19 genes prognosis-related differentially expressed genes (PRDEGs), which were both differentially expressed and prognostic among diverse subgroups. The gene clusters were constructed based on the expression levels of 19 PRDEGs by the unsupervised clustering algorithm mentioned above.

### Construction of the risk model

As mentioned above, we randomly divided the 4188 BC patients into a training group and a testing group. Least absolute shrinkage and selection operator (LASSO) analysis and multivariate Cox regression (multiCox) analysis implemented by the “glmnet” R package were applied to the patients in the training group to construct the risk model. All BC patients were then divided into two groups (low-risk group and high-risk group) according to the median value of the risk score. OS was compared between the high-risk and low-risk groups with K-M analysis. Receiver operating characteristic (ROC) analysis was performed using the “timeROC” R package to estimate the forecasting capability.

### Gene set enrichment analysis of diverse risk groups

First, the R package “limma” was applied between the high-risk and low-risk groups, and 45 upregulated and 69 downregulated DEGs in the high-risk group were subsequently selected with criteria of |logFC| > 1 and adjusted *p* value < 0.05. To reveal diverse patterns of RMPs through biological processes, Gene Ontology (GO) and Kyoto Encyclopedia of Genes and Genomes (KEGG) analyses were performed using the R package “clusterProfiler” (Yu et al., 2012).

### Immune infiltration and tumour microenvironment analysis

The single-sample gene set enrichment analysis (ssGSEA) method of in the R package “GSVA” was used to calculate the infiltration degree according to the expression levels of 28 published immune cell gene sets (Bindea et al., 2013; Hänzelmann et al., 2013). Estimation of STromal and Immune cells in Malignant Tumour tissues using Expression data (ESTIMATE) was applied to determine the components of stromal cells and immune cells according to the gene expression characteristics of tumour samples (Yoshihara et al., 2013). CIBERSORT was used to infer cell composition based on the expression profiles. This deconvolution algorithm was used to calculate the relative proportions of 22 immune cells in each patient with BC (Newman et al., 2015; Becht et al., 2016).

### Estimation of tumour mutation burden, stemness correlation and drug sensitivity

The “MutSigCV” algorithm was applied to screen 20 oncogenes with higher mutation frequencies than the background frequency. The R package “maftools” was applied to display the mutation landscape of the top altered oncogenes in the TCGA-BRCA cohort by waterfall plots (Mayakonda et al., 2018). The R package “pRRophetic” was used to predict the sensitivity of diverse risk groups by calculating the semi-inhibitory concentration (IC50) of commonly used drugs (Geeleher et al., 2014). In addition, we utilized the CellMiner database (https://discover.nci.nih.gov/cellminer/home.do) to analyse the drug sensitivity relevance between model-constructed genes and common antineoplastic drugs (Shankavaram et al., 2009). Spearman correlation analysis was applied to visualize the correlation between the risk score and the RNA-based stemness scores (RNAss).

### Clinicopathological stratification of the risk score

BC patients in the METABRIC cohort were assigned to subclasses based on the following diverse characteristics. According to the median risk score, cases in each clinical subgroup were assigned to the low-risk or high-risk group. Survival curves of the high-risk group and low-risk group in the subgroups were compared using the log-rank test and K-M analysis.

### Statistical analysis

All statistical analyses were performed using R software and its packages (version 4.1.2). Bilateral *p* < 0.05 was considered significant.

## Results

### The Landscape of RMPs with adenosine in BC

**FIGURE 1:**
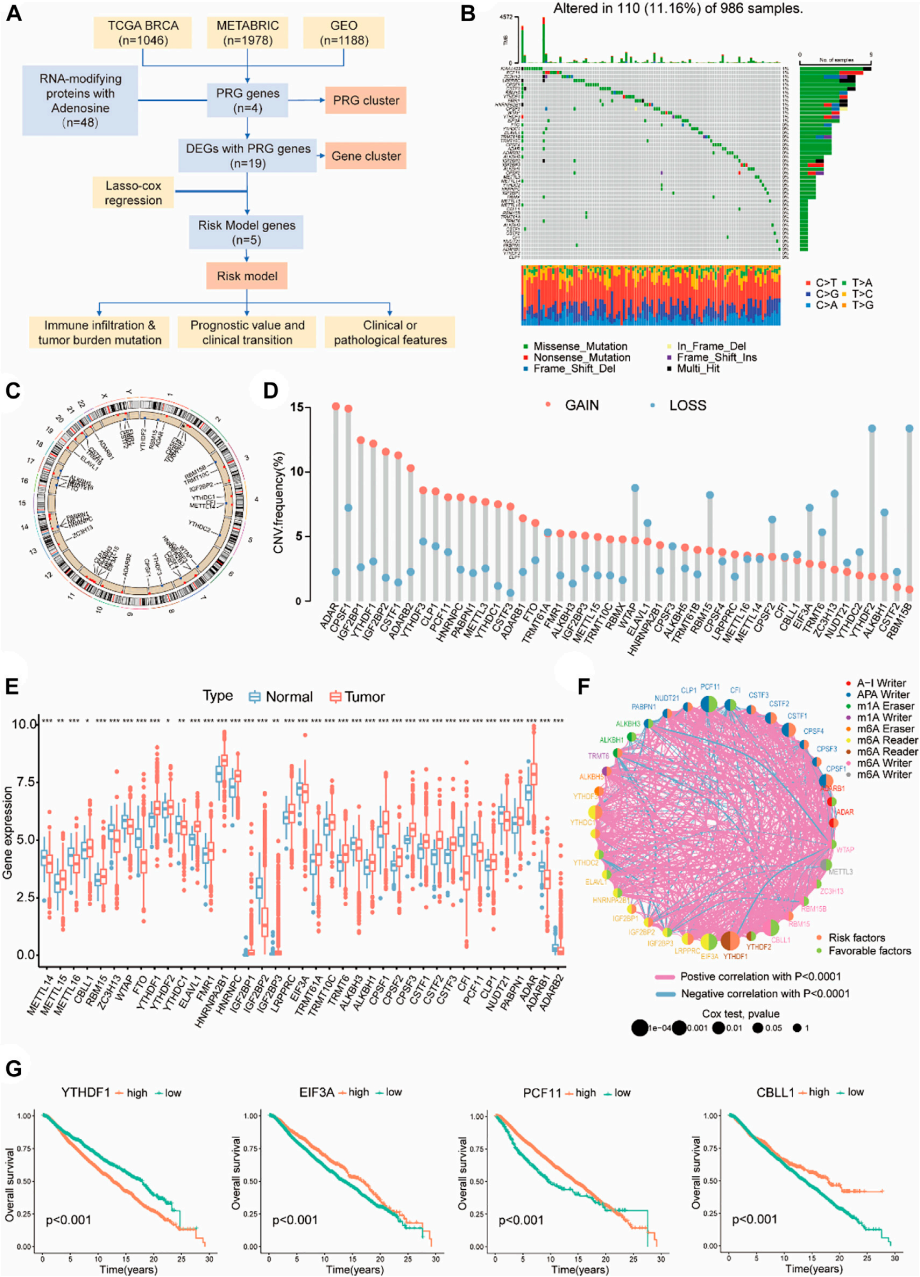
The Landscape of RMPs with adenosine in BC. **(A)** Workflow of the study. **(B)** 110 of 986 samples with breast cancer experienced genetic mutations of 48 RMPs, with a frequency of 11.16%. Each cohort represents an individual sample. The upper bar graph represents TMB; the number on the right side shows the mutation frequency in each RMP. The right bar graph shows the proportion of each mutation type for each BMP. The lower bar graph shows the fraction of transitions in each individual. **(C)** A circular diagram presenting the positions of CNV gains or losses of RMPs on 23 chromosomes. Red indicates CNV gained, and blue indicates CNV lost. **(D)** Barplots showing quantified CNV gains and losses of each RMP. **(E)** Expression levels of RMPs compared between BC and normal samples presented with barplots. Red represents tumours, and blue represents normal tissues. The asterisks represented the statistical *p* value (*p < 0.05; **p < 0.01; ***p < 0.001). **(F)** The intercorrelation and survival connections of RMPs. The colour of the right half of the circle represents its survival association, the colour of the left half represents the type of RMP, and the size of the circle represents the uniCox *p* value. **(G)** K-M curve showing the OS and survival probabilities of the four PGRs in the cohort of 4188 BC patients with the best cut-off value.

An overview of our workflow is outlined in Figure 1A. To obtain a fully picture of the expression and mutation patterns of RMPs associated with adenosine, we summarized 48 reported RMPs associated with adenosine modification, including 10 m6A “writers”, 2 m6A “erasers”, 15 m6A “readers”, 4 m1A “writers”, 2 m1A “erasers”, 12 APA “writers” and 3 A-to-I “writers”, and listed them in Supplementary Table S1.

Non-silent somatic mutations were detected among RMPs to identify whether they were associated with genetic alterations. Of the 986 BC samples in TCGA-BRCA, 110 (11.16%) samples had mutations of RMPs, most of which were missense mutations. As depicted in Figure 1B, KIAA1429 exhibited the highest mutation frequency in BC samples, followed by PCF11 and ZC3H13. On the contrary, YTHDF2 and CLP1 did not exhibit any mutations.

Then, we examined the somatic CNVs of these RMPs. We found extensive CNV gains in ADAR, CPSF1, IGF2BP1 and YTHDF1. Loss of copy numbers was observed in RBM15B and YTHDF2 (Figure 1D). A circular diagram was created to illustrate the locations of these RMPs on the 23 chromosomes (Figure 1C).

BC samples were paired with normal breast tissue samples to compare mRNA expression levels of RMPs. 39 RMPs were found in the TCGA-BRCA cohort, and most of them were abundant in the tumour samples (Figure 1E).

To demonstrate the links between these RMPs and the outcomes of BC patients, the clinical and transcriptome data of 4188 BC patients were enrolled in this study to reveal the relationships between RMPs and tumorigenesis. The results suggested that there were strong correlations between different types of RMPs and that positive correlations are more common than negative ones. Notably, YTHDF1, CBLL1, PCF11, and EIF3A were most related to survival, with a uniCox *p* value < 0.001 (Figure 1F). The results of uniCox for RMPs are displayed (Supplementary Table S2). Patients with higher expression level of YTHDF1 had worse prognoses, and those with higher expression of CBLL, PCF11, and EIF3A had better clinical outcomes (Figure 1G). These four genes were defined as PRGs, and other RMPs associated with survival were also noted (Supplementary Figure S1).

### PRG clusters and gene clusters associated with four RMPs

**FIGURE 2:**
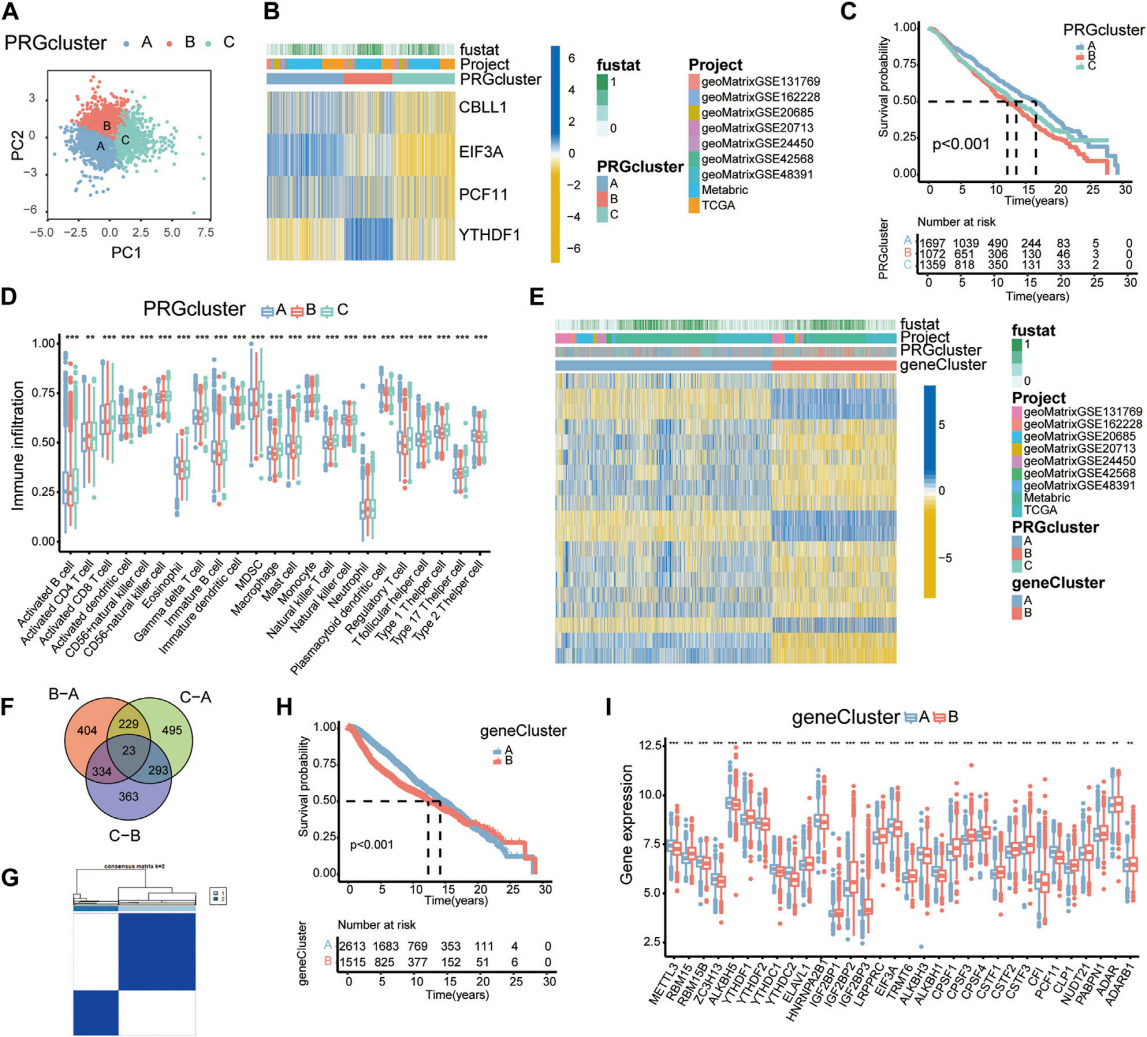
PRG clusters and gene clusters associated with four PRGs. **(A)** PCA of the PRG clusters. **(B)** Heatmap showing the expression profiles of four PRGs in the PRG clusters. **(C)** K-M survival curves of PRG clusters based on 4188 BC patients. Blue/red/green represent PRG clusters A/B/C. **(D)** Boxplots showing immune cell infiltration in each PRG cluster by ssGSEA. *p < 0.05; **p < 0.01; ***p < 0.001. **(E)** Heatmap showing the expression profiles of 19 PRDEGs among gene clusters. **(F)** A Venn diagram showing DEGs. **(G)** Consensus matrix displaying two major gene clusters based on PRDEGs. **(H)** K-M survival curves of gene clusters (*n* = 4,188). **(I)** Expression profiles of RMPs in variant gene clusters.

Utilizing the consensus clustering analysis, 4188 BC patients were then assigned into three subgroups. A total of three PRG clusters were identified, with 1,718 patients in PRG cluster A, 1,084 patients in PRG cluster B, and 1,386 patients in PRG cluster C. PCA confirmed an intergroup distribution among the three subgroups (Figure 2A).

The heatmap was depicted to reveal expression levels of four PRGs in these three PRG clusters. CBLL1, EIF3A, and PCF11 were markedly overexpressed in cluster A patients, while YTHDF1 was expressed at higher levels in cluster B patients (Figure 2B).

The survival analysis of PRG clusters suggested that patients in cluster A had a much more prominent survival advantage than those in cluster B, and the advantage for patients in cluster C was somewhere in between, which was consistent with the expression levels and the prognostic trends of the four PRGs (Figure 2C).

To understand biological processes in diverse RNA modification patterns, the “GSVA” R package was utilized to conduct gene set variation analysis (GSVA). PRG cluster B was markedly enriched in metabolic pathways such as ribosome, fatty acid metabolism, valine leucine and isoleucine degradation, and propanoate metabolism compared to PRG cluster A (Supplementary Figure S2).

Many studies have mentioned a potential link between infiltrating immune cells and RNA modification (Barbieri and Kouzarides, 2020). To investigate the functional role of RMPs in immune infiltration, ssGSEA was applied to the PRG clusters and revealed a strong connection between PRG clusters and immune cells (Figure 2D).

The identified PRG clusters could effectively distinguish the clinical outcome of BC patients; however, the PRG-related genes, therapeutic effect and underlying reasons were still unclear. To identify the genes potentially related to the PRG clusters, overlapping DEGs among the 3 PRG clusters were then selected. We obtained 23 PRG-related DEGs and displayed them with a Venn diagram (Figure 2F).

UniCox analysis was performed and identified 19 prognosis-related DEGs. According to the expression levels of these 19 PRDEGs, we divided the patients into two subgroups: gene cluster A and gene cluster B (Figure 2G).

In the entire transcriptome, the heatmap showed significant inherent differences between the gene clusters (Figure 2E). K-M curves showed significant alterations in survival outcomes between the two gene subtypes. Gene cluster A presented a clear survival advantage, while gene cluster B had a higher risk of death (Figure 2H). The expression levels of RMPs in gene clusters were examined, which revealed substantial discrepancies in RMPs and suggested an underlying correlation. Notably, YTHDF1 showed a higher expression level in gene cluster B, while PCF11 and EIF3A were more highly expressed in gene cluster A (Figure 2I).

### Construction of the risk model

To accurately forecast the survival status of individuals, we designed a risk model. The BC patients in the training group were used for the following model construction procedure.

**FIGURE 3:**
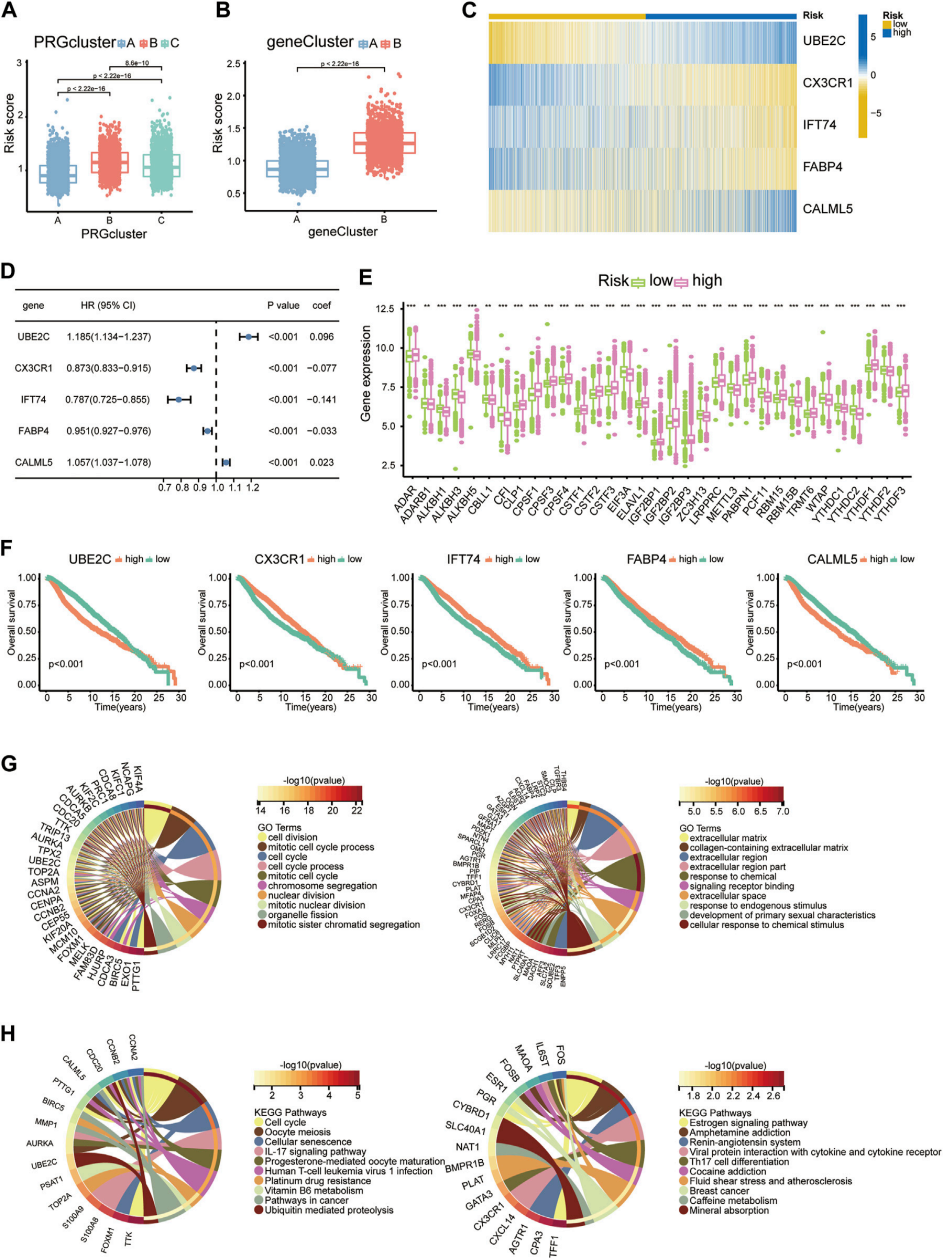
Construction of the risk model. **(A)** Risk scores of diverse PRG clusters. **(B)** Risk scores of diverse gene clusters. **(C)** Heatmap showing the expression profiles of 5 model-constructed genes in the risk groups. **(D)** Table listing the coefficient and HR of each model-constructed gene. **(E)** RMP expression levels in risk groups presented with boxplots. **(F)** K-M curves of 5 model-constructed genes. **(G)** GO functional enrichment analysis of upregulated DEGs (left) and downregulated DEGs (right) in the high-risk group. **(H)** KEGG analysis results of upregulated DEGs (left) and downregulated DEGs (right) in the high-risk group.

Nineteen PRDEGs were engaged in the iterative LASSO algorithm (Figures 7A,B). Next, multiCox analysis was applied to construct the risk model. Ultimately, risk scores were calculated by multiplying the expression values of five chosen genes with their regression coefficients. The formula was as follows: risk score = (expression level of UBE2C × 0.096)—(expression level of CX3CR1 × 0.077)—(expression level of IFT74 × 0.141)—(expression level of FABP4 × 0.033) + (expression level of CALML5 × 0.023). We then evaluated the risk score of each patient in the training and testing groups, which is listed in Supplementary Table S3. The hazard ratio from uniCox and the coefficients of the 5 model-constructed genes are listed (Figure 3D). We compared the risk scores in diverse PRG clusters and gene clusters. It was revealed that PRG cluster B and gene cluster B, which had poor survival, had higher risk scores (Figures 3A,B).

We separate the 4188 BC patients into high- and low-risk groups based on the median cut-off value of the developed risk score. The heatmap showed differential expression of model-constructing genes between the high-risk and low-risk groups among the 4188 BC patients, where UBE2C and CALML5 were distinctly upregulated in the high-risk group, and CX3CR1, IFT74, and FABP4 showed the opposite trend (Figure 3C). The training group and testing group showed the same condition (Figure 7C).

The K–M plot of the five model-constructing genes showed that with higher expression levels of UBE2C and CALML5, patients had worse survival probabilities, while with higher expression of the other three genes, patients had better outcomes (Figure 3F).

The links between the risk groups and expression profiles of RMPs is presented. We observed that YTHDF1 presented a significant expression increase in the high-risk group, while CBLL1, EIF3A, and PCF11 performed downregulation. The expression profiles of ADAR, CLP1, CPSF1/3/4, CSTF1/2/3, ELAVL1, IGF2BP1/2/3, LRPPRC, PABPN1, RBM15, RBM15B, TRMT6, and YTHDF2/3 were markedly positively correlated with the patient’s risk score and most of them were associated with a poor clinical outcome (Figure 3E).

To uncover the potential biological characteristics between the two groups, we searched for DEGs and carried out enrichment analyses. Analysis of DEGs enhanced in the high-risk group revealed enrichment of GO functions such as cell division, mitotic cell cycle process and cell cycle, which indicated that the potential mechanism underlying poor survival may be linked to cell proliferation (Figure 3G). In the KEGG analysis, we also observed proliferation pathways enriched among DEGs elevated in the group with a higher average risk score. A higher risk score may be also associated with poor sensitivity to platinum-based drug therapy, since IL-17 and Th17 cells are both related to inflammation-related tumour development. We observed that the IL-17 signalling pathway and Th17-cell differentiation pathway were enriched in the upregulated and downregulated groups, respectively, which suggested an indispensable role of the immune and inflammatory systems in tumour development with the adenosine-RNA-modification-derived risk model (Figure 3H).

### The relationship between tumour mutation burden, immune infiltration and risk score

**FIGURE 4:**
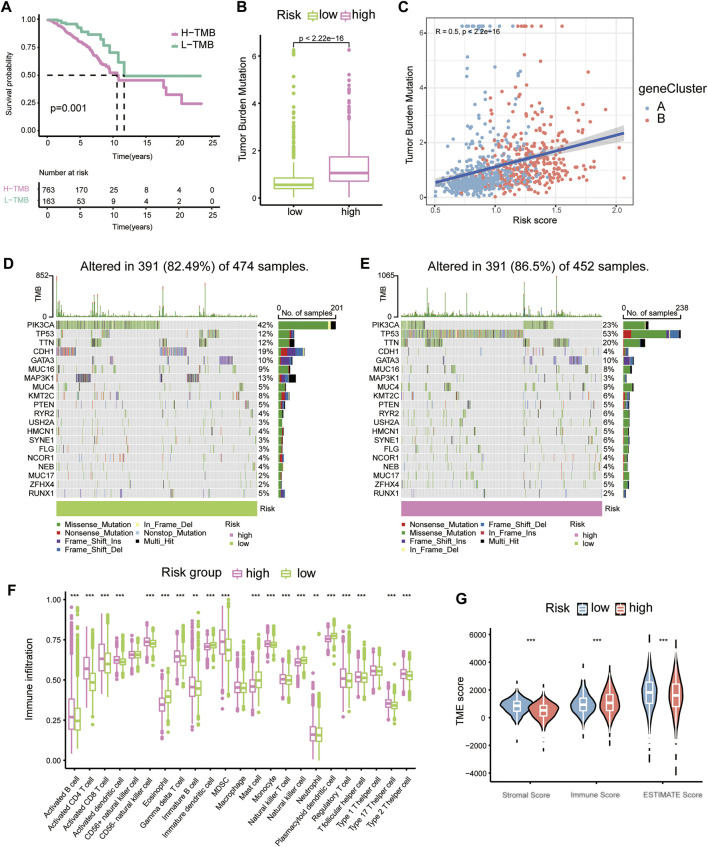
The relationship between tumour mutation burden, immune infiltration and risk score. **(A)** K-M plots of high- and low-TMB groups of the TCGA-BRCA cohort. **(B)** Boxplots showing the relative connection of the risk score and TMB. **(C)** Correlation scatter plot presenting the relationships among gene clusters, risk scores, and TMB. **(D)** Waterfall plots showing TMB for the low-risk group. **(E)** Waterfall plots showing TMB for the high-risk group. **(F)** Immune cell proportions in diverse risk groups shown with boxplots. **(G)** Violin plots for stromal score, immune score and ESTIMATE score in different risk groups.

We then analysed the underlying links between the risk score and tumour mutation burden (TMB) with the TCGA-BRCA database. K–M plots depicted that with lower TMBs, BC patients exhibited a distinct survival advantage, while the others had worse clinical outcomes (Figure 4A). The TMB landscape among the top 20 most common mutations suggested that with higher risk scores, BC patients had a more extensive TMB (Figures 4D,E). The risk score was positively correlated with TMB (Figures 4B,C).

**FIGURE 5:**
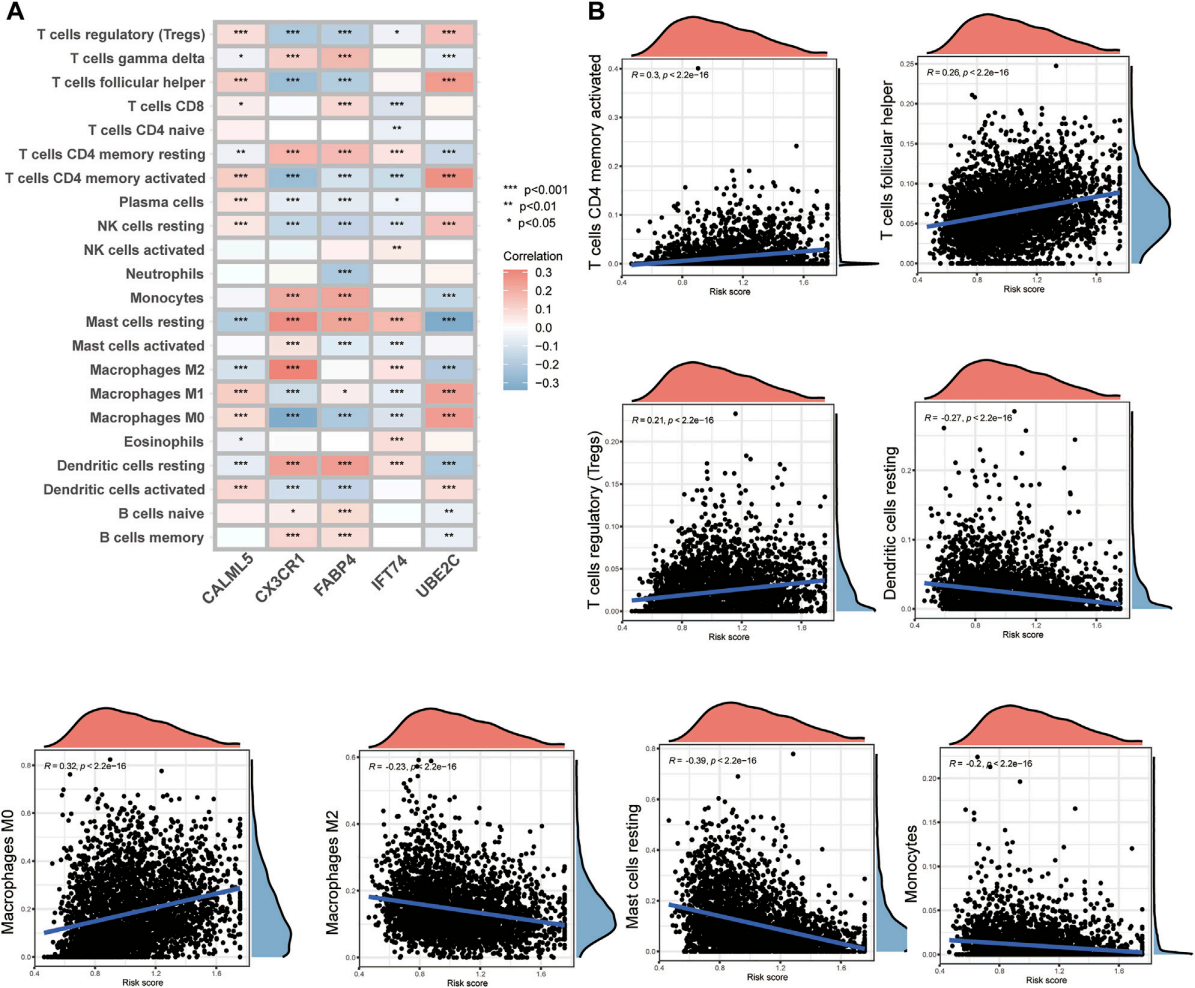
The immune cell infiltration connections associated with model-constructed genes and risk scores. **(A)** A heatmap demonstrating the relationship between model-constructing genes and immune cell infiltration. **(B)** Correlation scatter plots showing the relationship between risk scores and immune cell infiltration.

Through previous analysis, we found that Th17 and IL-17 may be associated with the potential bio-mechanism of risk models, and several RMPs were documented to be linked to immunotherapy resistance. To learn whether the risk score could predict immunotherapy response of BC patients, we analysed the immune cells infiltration profiles by ssGSEA. Most immune cells were upregulated in the high-risk groups (Figure 4F). Immune cell infiltration (immune score) was also evaluated by the ESTIMATE algorithm, and the immune score was obtained, which was consistent with the results of ssGSEA. The tumour microenvironment (TME) score of each case was evaluated, and the stromal score, immune score and ESTIMATE score were determined. The stromal score and ESTIMATE score were both decreased in the high-risk group (Figure 4G). By CIBERSORT, we analysed the correlations of immune cells with the risk score and risk model-building genes, it was depicted that CX3CR1 were strongly positive correlated with M2 macrophages but the trend was opposite to that of M1 macrophages and UBE2C showed the opposite (Figure 5A). There was a significant positive correlation between inhibitory immune cell Tregs and risk, but at the same time M1 macrophages were significantly upregulated, accompanied by downregulation of M2 macrophages (Figure 5B).

### Prognostic value and clinical translation of the risk model

**FIGURE 6:**
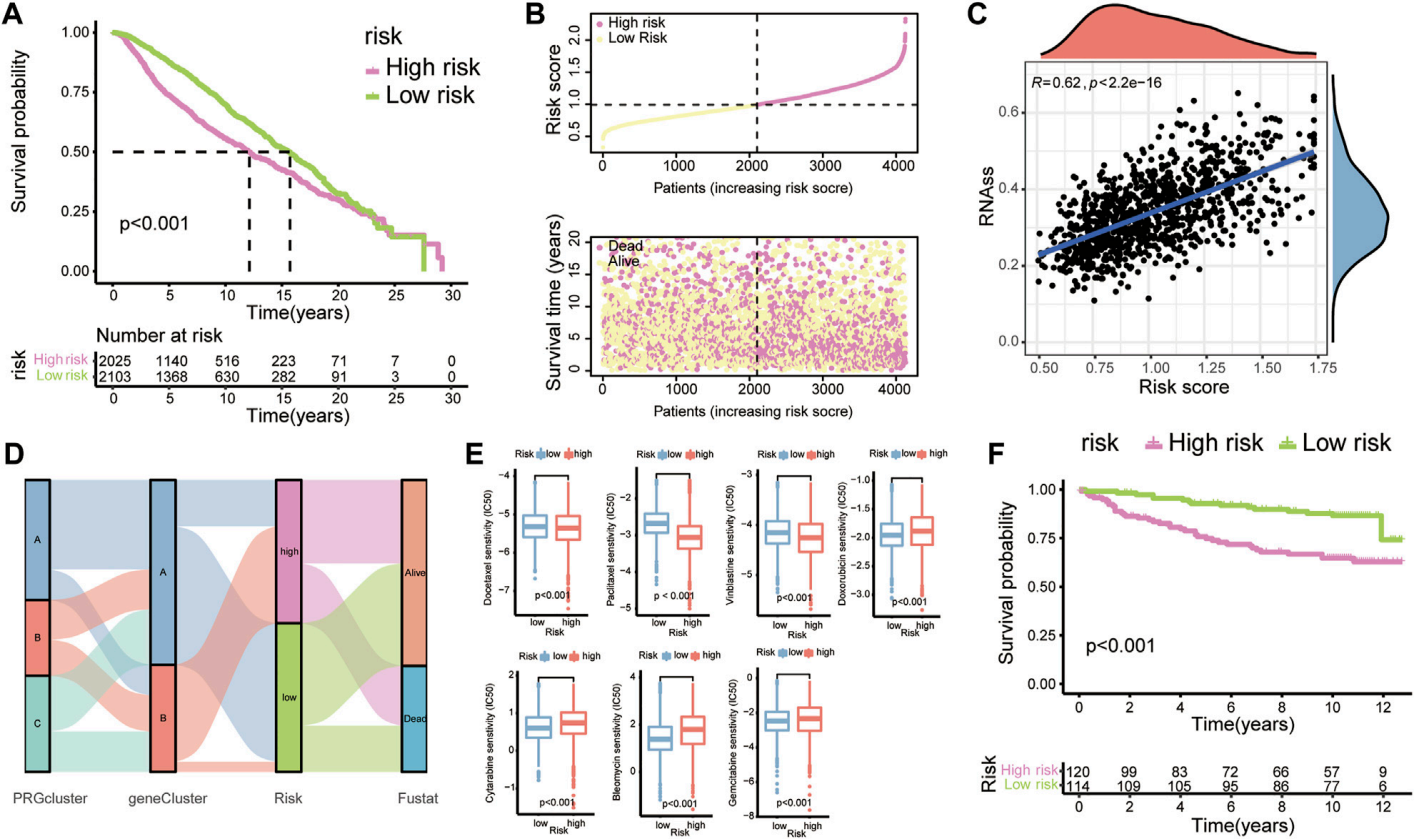
Prognostic value and clinical translation of the risk model. **(A)** K-M curves of risk groups in the merged BC cohort. **(B)** Correlation scatter plots showing the relationship between clinical outcomes and risk scores. **(C)** The relationship between tumour cell stemness and risk score. **(D)** A Sankey map demonstrating the relationship between clinical outcomes and diverse subgroups (PRG clusters, gene clusters, and risk groups). **(E)** Boxplots showing the IC50 values of common anti-tumour drugs. **(F)** K-M plots of BC patients in GSE3494.

**FIGURE 7:**
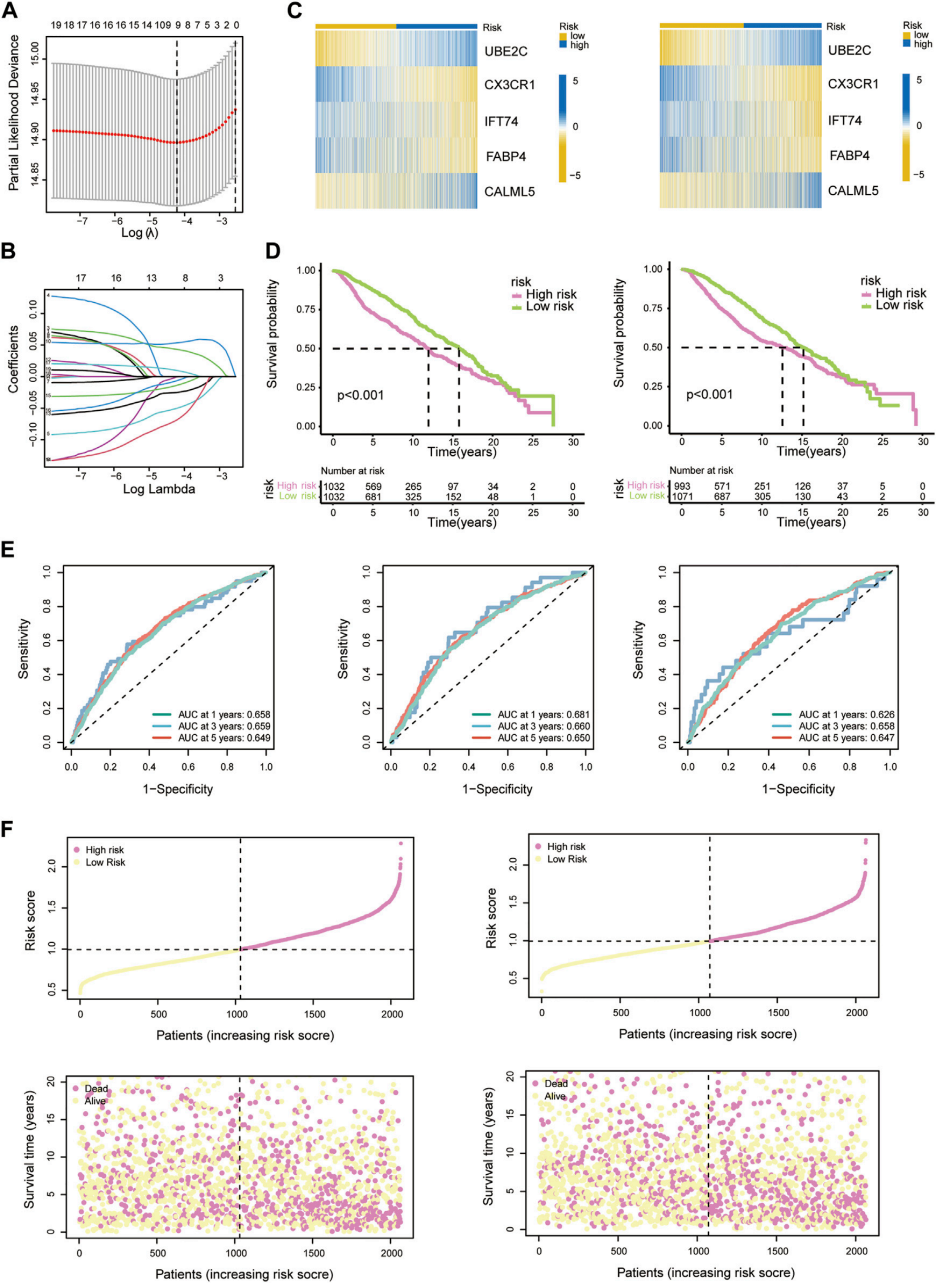
**(A)** LASSO regression analysis reducing variants. **(B)** Coefficients of model-constructed genes obtained by LASSO. **(C)** Heatmap of the expression profiles of model-constructed genes in the training group (left) and testing group (right). **(D)** K-M curves of patients in the training group (left) and testing group (right). **(E)** AUC time-dependent ROC curves for OS in all patients (left), training group patients (middle) and testing group patients (right). **(F)** Correlation scatter plots show the relationship between clinical outcomes and risk scores in the training group (left) and testing group (right).

To better learn the correlation of risk scores with prognosis and drug therapy, the following analysis was performed. The K-M analysis showed that patients in low-risk group had evident advantages of survival, while high-risk patients had poor clinical outcomes (Figure 6A). The K-M analysis results for the training and testing groups are also displayed (Figure 7D). Furthermore, we applied the timeROC method to estimate the AUC values for predicting OS (Figure 7E). The relevance between clinical outcomes and risk scores of patients is also displayed (Figure 6B), and the results for the training and testing groups are also shown (Figure 7F). GSE3494 was chosen as an external cohort for feasibility verification, and there was an apparent discrepancy in clinical outcomes, with the high-risk group having worse outcomes (Figure 6F). All the above results indicate that the risk model is stable and can precisely predict the clinical outcome of patients.

To visualize the relationship between risk scores and diverse clusters, we displayed the relationships among PRG clusters, gene clusters, risk groups and the survival outcome of patients with a Sankey diagram (Figure 6D). Rapidly developed tumour cells can lose differentiation phenotypes and exhibit stem-cell-like characteristics (Batlle and Clevers, 2017). RNAss scores based on mRNA expression were utilized to measure the correlation between tumour stemness and the risk score (Figure 6C).

Next, to determine the effectiveness of the risk score for predicting drug treatment response in BC patients, we estimated the IC50 values of the most common drugs. We found that patients in the high-risk group might be more sensitive to M-phase cell cycle drugs, including docetaxel, paclitaxel and vinblastine, but resistant to the cell cycle-nonspecific drug doxorubicin, the S-phase-specific drugs cytarabine and gemcitabine and the G2-phase-specific drug bleomycin (Figure 6E).

**FIGURE 8:**
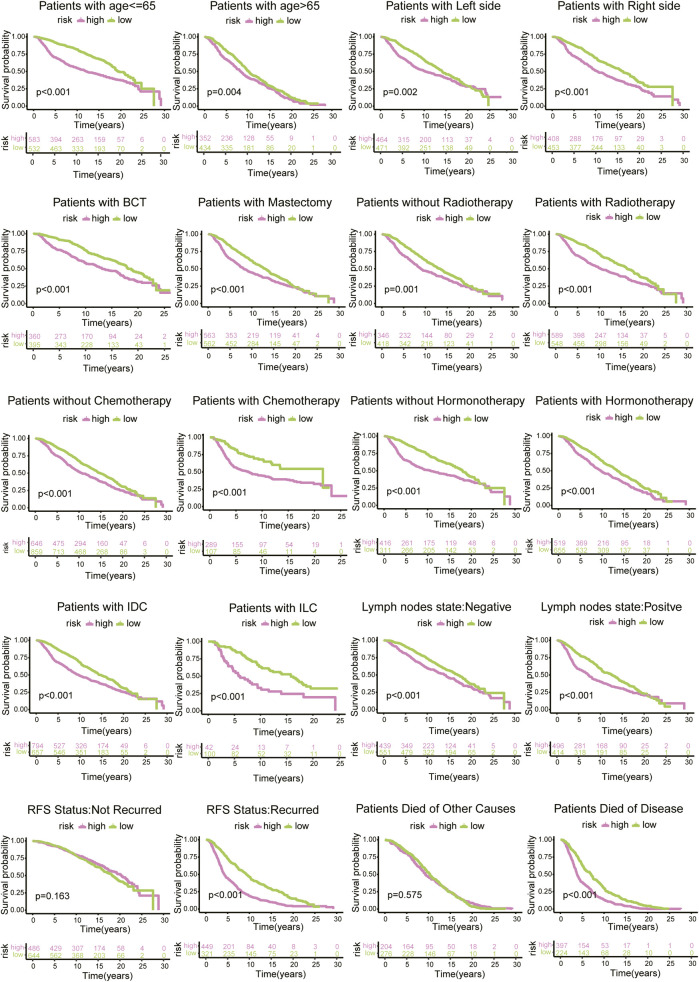
Survival analysis of risk scores based on diverse clinicopathological characteristics of 1130 BC patients from METABRIC database.

According to diverse clinicopathological factors (age of diagnosis, tumour side, surgical type, radiotherapy, chemotherapy, hormonotherapy, histology type, lymph node state, RFS status, and reason for death), BC patients from the METABRIC database were divided into different cohorts. The risk model presented excellent prediction performance. In particular, there was a distinct difference in the survival outcomes of patients with recurrence, and no diversity was found in patients without recurrence. Similarly, the survival curve of patients who died of disease was changed, but there was no such alteration for patients who died for other reasons (Figure 8).

## Discussion

Epigenetic transcriptomics focusing on RNA modification, an unexplored field, has been gradually explored with the continuous development of sequencing technology. The majority of studies focused on only one specific single form of RNA modification mode, and there is increasing evidence that RMPs play an indispensable role in tumorigenesis and interact with each other; nevertheless, the interrelationships of multiple forms of RMPs are still not completely understood (Zhang et al., 2016; Zhao et al., 2017). Here, we ultimately revealed a landscape of transcriptional and genetic alterations of adenosine-related RNA-regulatory enzymes of m6A, m1A, APA, as well as A-to-I and discussed their potential connections, expression patterns and prognostic values in BC.

To obtain a brief understanding, we depicted the connections among RMPs, and most of them were associated with tumour mutation burdens and patient outcomes and showed differential expression levels between BC tumours and normal tissues. The RMPs upregulated in tumours or showing a positive correlation with poor outcomes usually gained more CNVs, such as ADAR, CPSF1, IGF2BP1, and YTHDF1.

We then chose the four most prognostic genes, YTHDF1, EIF3A, PCF11, and CBLL1, according to the survival analysis results and named them PRGs. Three PRG clusters and two gene clusters based on PRGs and PRDEGs were then identified in 4188 BC patients and found to predict the patients’ outcomes in diverse clusters. To more accurately anticipate the prognosis of individuals, a risk model was constructed to forecast prognostic risk scores of BC patients. The patients with higher risk were identified as having higher expression levels of UBE2C, and CALML5 and lower expression levels of CX3CR1, IFT74, and FABP4. UBE2C encodes a E2 ubiquitin-conjugating enzyme, which was found to be a prognostic factor in BC with poor survival in previous studies (Psyrri et al., 2012). Studies have shown that CALML5 ubiquitination is involved in the tumorigenesis of the BC (Debald et al., 2013).

We explored RMP expression patterns between risk groups and connected specific RMPs with BC outcomes. YTHDF1 was highly expressed in high-risk group patients, while EIF3A, PCF11, and CBLL1 showed higher expression levels in low-risk group patients. BC patients with higher risk scores had a worse prognosis. We also confirmed the prognostic value of the risk model by assessing outcomes in BC patients with different clinicopathological features.

To explore the underlying mechanisms, functional enrichment analysis was performed, suggesting that cell proliferation-related functions such as cell division, cell cycle, chromosome segregation and mitotic nuclear division were stronger in the high-risk group. We then visualized the potential therapeutic effects of diverse RMP-related risk patterns in BC. Similarly, the high-risk group was associated with resistance to the cell cycle-nonspecific drug doxorubicin and the S- or G2-phase cell cycle-specific drugs cytarabine, gemcitabine and bleomycin but with sensitivity to M-phase cell cycle-specific drugs. It has been reported that YTHDF1, which was upregulated in the high-risk group, promotes S-phase entry, DNA replication and DNA damage repair (Sun et al., 2022). EIF3A regulates the expression profiles of proteins that contribute to DNA repair, which in turn is involved in response to anti-cancer drugs (Yin et al., 2011). UBE2C can directly override the spindle assembly checkpoint inhibition of APC (Reddy et al., 2007; Meyer and Rape, 2011). These literature reports prove the reliability of our conjecture.

The abundance of TMBs was markedly difference between the two risk groups, and the high-risk group was associated with a higher TMB. The results of RNAss analysis indicated a close relationship between the risk score model and tumour cell stemness, which was reported previously (Du et al., 2021). The difference in the degree of immune cells infiltration is also indisputable. In patients with a higher risk score, we observed higher levels of infiltration in Tregs and MDSCs, which contribute to immune suppression (Wood et al., 2012). This suggested that patients in the high-risk group showed higher infiltration of immune suppressor cells. CX3CR1 was reported to be a marker of T cell differentiation, which indicated a predictive correlate of response to immune checkpoint inhibitor therapy, and CX3CR1 + inhibitory macrophages were negatively correlated with T-cell expansion (Bassez et al., 2021; Yamauchi et al., 2021). Since the exact mechanism is still unclear, the connections in immune cell infiltration should be further analysed.

This study is one of the few to combine four RNA modification types with breast cancer and predict patient outcome, which opens up a whole new way of model prediction and clinical evaluation. However, there are still limitations. While the mechanisms behind RNA modification are still not well understood, the association between RNA modifications and cancer diversity needs further exploration and further investigation. And we must admit genetic testing continues to be an invasive method that imposes a financial burden and risk on patients. Therefore, more economical and convenient detection methods need to be further explored.

## Conclusion

A systematic and comprehensive landscape of four types of adenosine-related RMPs in BC was constructed, revealing expression profiles, tumour mutation burden, immune cell infiltration and connections with BC survival outcomes. We constructed a risk model and evaluated several aspects, including genomic mutation, immune relativity and therapeutic responses. The model presented effective performance in predicting an individual’s clinical outcome in relation to BC. This work highlights the clinical importance of cross-talk between diverse types of RNA modification with a large number of cases and presented a predictive model which contributes to the improvement of prediction and treatment patterns. However, simpler and more economical methods of precise diagnosis still need to be discovered.

## Data Availability

The databases used and analysed during the current study are available from the Gene Expression Omnibus (GEO) database (https://www.ncbi.nlm.nih.gov/geo/) with the accession numbers GSE131769, GSE162228, GSE20685, GSE20713, GSE24450, GSE42568, GSE48391, and GSE3494; the Molecular Taxonomy of Breast Cancer International Consortium (METABRIC) database (http://www.cbioportal.org/) and The Cancer Genome Atlas (TCGA) database (https://portal.gdc.cancer.gov/) with the accession number TCGA447 BRCA.

## Abbreviations

ADAR, double-stranded RNA-specific adenosine deaminase; APA, Alternative polyadenylation; A-to-I, Adenosine-to-inosine; BC, Breast invasive carcinoma; CNVs, Copy number variations; DEG, Differentially expressed genes; GEO, Gene Expression Omnibus; OS, Overall survival; GO, Gene Ontology; KEGG, Kyoto Encyclopedia of Genes and Genomes; LASSO, Least absolute shrinkage and selection operator; M1A, N1-methyladenosine; M6A, N6-methyladenosine; METABRIC, Molecular Taxonomy of Breast Cancer International Consortium; MultiCox, Multivariate Cox regression; PCA, Principal component analysis; SsGSEA, Single sample gene set enrichment analysis; TCGA, The Cancer Genome Atlas; TMB, Tumour mutation burden; TME, The tumour microenvironment; UniCox, Univariate Cox regression.
