# Supplementary material for: Cross-talk of four types of RNA modification proteins with adenosine reveals the landscape of multivariate prognostic patterns in breast cancer
Source: Front Genet. 2022 Sep 2;13:943378. doi: 10.3389/fgene.2022.943378 (PMC9479131; doi:10.3389/fgene.2022.943378)
Supplement: Supplementary file 1 [file DataSheet1.PDF]

Supplementary Material

Supplementary Figure 1 | K-M survival curves of RMPs.

Supplementary Figure 2 | KEGG functional enrichment analysis investigating the biological process alterations of the three PGR clusters.

Supplementary Table 1 | Summary of 48 RMPs.

Supplementary Table 2 | HR of 35 RMPs.

Supplementary Table 3 | The risk score of each sample.

Supplementary Figure 1

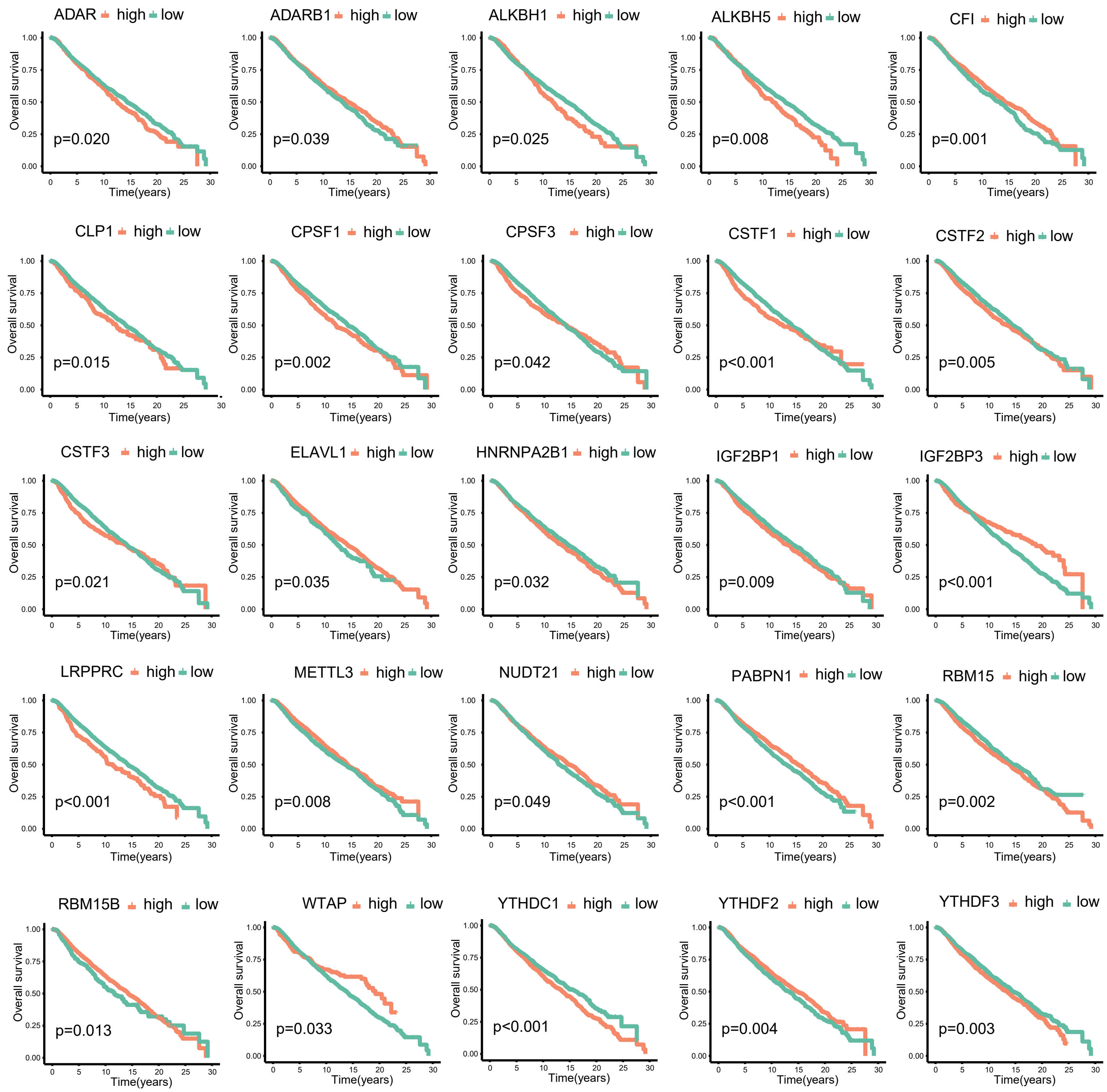

Supplementary Figure 2

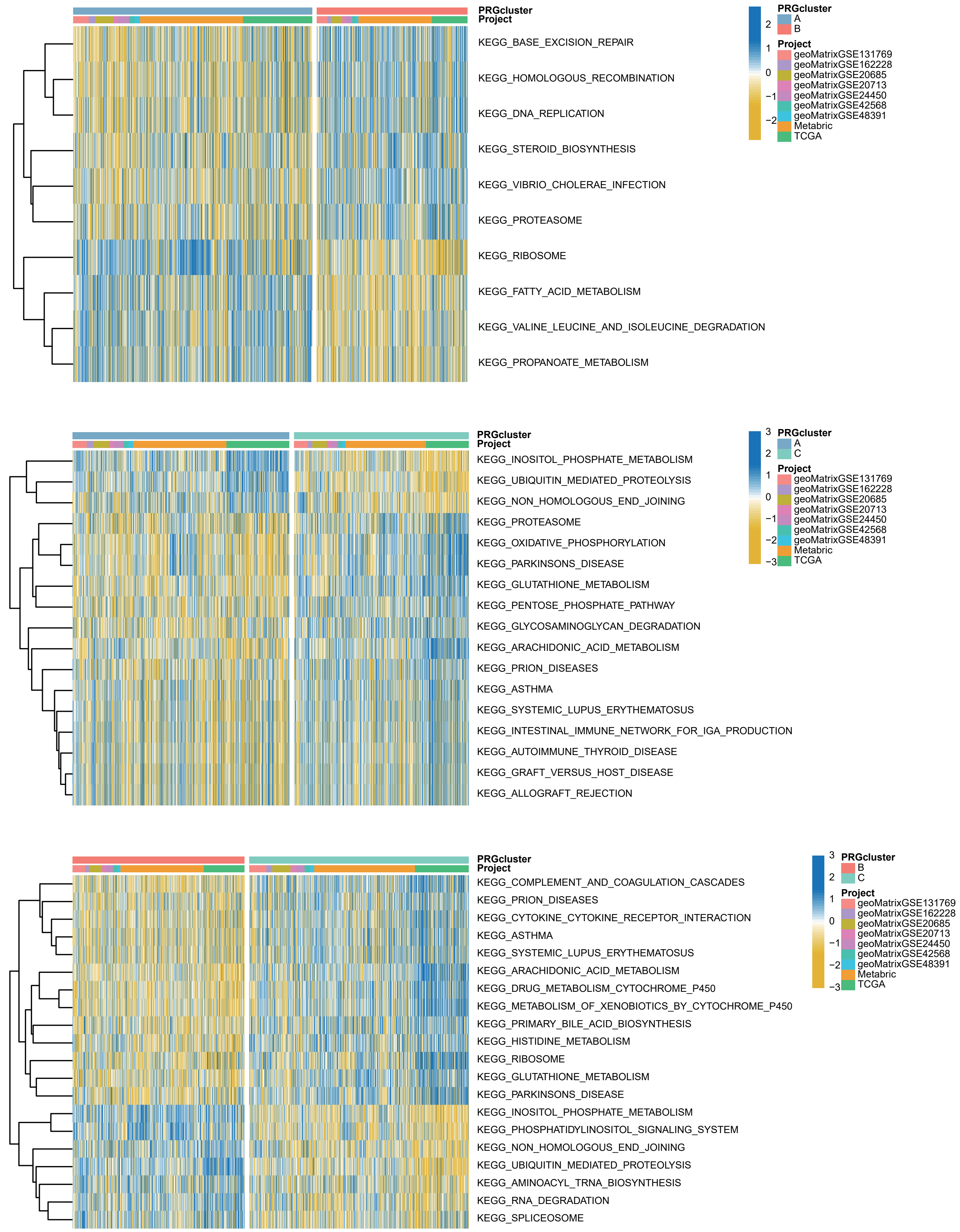

Table S1: summarization of 48 RMPs

Gene	Type
METTL3	m6A Writer
METTL14	m6A Writer
METTL15	m6A Writer
METTL16	m6A Writer
CBLL1	m6A Writer
RBM15	m6A Writer
RBM15B	m6A Writer
ZC3H13	m6A Writer
KIAA1429	m6A Writer
WTAP	m6A Writer
FTO	m6A Eraser
ALKBH5	m6A Eraser
YTHDF1	m6A Reader
YTHDF2	m6A Reader
YTHDF3	m6A Reader
YTHDC1	m6A Reader
YTHDC2	m6A Reader
ELAVL1	m6A Reader
FMR1	m6A Reader
HNRNPA2B1	m6A Reader
HNRNPC	m6A Reader
IGF2BP1	m6A Reader
IGF2BP2	m6A Reader
IGF2BP3	m6A Reader
LRPPRC	m6A Reader
RBMX	m6A Reader
EIF3A	m6A Reader
TRMT61A	m1A Writer
TRMT61B	m1A Writer
TRMT10C	m1A Writer
TRMT6	m1A Writer
ALKBH3	m1A Eraser
ALKBH1	m1A Eraser
CPSF1	APA Writer
CPSF2	APA Writer
CPSF3	APA Writer
CPSF4	APA Writer
CSTF1	APA Writer
CSTF2	APA Writer
CSTF3	APA Writer
CFI	APA Writer
PCF11	APA Writer
CLP1	APA Writer
NUDT21	APA Writer
PABPN1	APA Writer
ADAR	A-I Writer
ADARB1	A-I Writer
ADARB2	A-I Writer

Table S2: HR of 35 RMPs

id	HR	HR.95L	HR.95H	pvalue
YTHDF1	1.441972	1.291724	1.609696	7.05E-11
EIF3A	0.846475	0.775017	0.924521	0.000212234
CBLL1	0.809884	0.715957	0.916133	0.000800343
PCF11	0.823751	0.734304	0.924094	0.000946361
YTHDF3	1.157509	1.050308	1.275652	0.003179502
CSTF1	1.198685	1.054971	1.361977	0.005415581
CPSF1	1.103572	1.023966	1.189368	0.009881705
LRPPRC	1.137163	1.023517	1.263427	0.016727182
CSTF2	1.132037	1.012684	1.265457	0.029131652
METTL3	0.885547	0.791133	0.991229	0.034589968
CFI	0.926795	0.861909	0.996565	0.040084065
IGF2BP1	1.154488	0.994369	1.34039	0.059316356
YTHDC1	1.13979	0.993574	1.307524	0.061771945
YTHDF2	0.898607	0.79369	1.017392	0.091458491
ADAR	1.072725	0.982977	1.170667	0.115302091
PABPN1	0.92606	0.839973	1.020971	0.122814241
WTAP	0.921835	0.830439	1.023289	0.12656079
RBM15B	0.922061	0.815863	1.042082	0.193699543
IGF2BP3	0.950735	0.880411	1.026675	0.197562805
CLP1	1.077696	0.95273	1.219052	0.234081846
RBM15	1.069903	0.954674	1.199041	0.245175134
CSTF3	1.043209	0.95244	1.142628	0.362405468
ALKBH3	0.969647	0.890284	1.056085	0.479279065
CPSF4	1.045504	0.923064	1.184186	0.483788724
IGF2BP2	1.017002	0.964131	1.072772	0.535960423
TRMT6	1.038662	0.913677	1.180744	0.56199004
ADARB1	0.974437	0.879901	1.07913	0.618945495
ELAVL1	0.970914	0.861927	1.093681	0.627045289
ZC3H13	0.982109	0.869303	1.109553	0.771815834
ALKBH5	1.015639	0.898239	1.148383	0.804443285
HNRNPA2B1	0.990902	0.895777	1.096129	0.859122846
NUDT21	1.007382	0.919879	1.103208	0.873955772
ALKBH1	1.007918	0.889679	1.141871	0.901408129
CPSF3	1.00397	0.8854	1.138418	0.950734129
YTHDC2	0.999656	0.898374	1.112357	0.994965351

Table S3: the risk score of each sample

id	futime	fustat	UBE2C	CX3CR1	IFT74	FABP4	CALML5	riskScore	risk
GSM3818017	9.378082192	0	7.109292	6.673984	6.107533	9.122885	4.385984	0.770689489	low
GSM3818019	8.287643836	0	9.03109	5.472622	5.961128	6.728254	4.154842	1.119235858	high
GSM3818021	2.328739726	1	8.876072	5.677637	6.114952	9.141384	4.219406	0.981583431	low
GSM3818022	7.11230137	0	7.184662	6.833609	6.124393	10.724268	6.310711	0.75773355	low
GSM3818023	7.687643836	0	7.644733	5.664227	6.458551	9.795154	4.31933	0.815332842	low
GSM3818026	9.161671233	0	9.79303	5.844202	6.804431	5.629944	8.117836	1.179621696	high
GSM3818027	8.843835616	0	8.290558	8.734142	5.941094	5.754528	4.569293	0.846978301	low
GSM3818028	9.474	0	13.008307	15.19263	5.212061	5.552415	4.559929	0.904063694	low
GSM3818029	9.397232877	0	8.395559	5.985611	7.360696	5.409626	4.58848	0.876587525	low
GSM3818033	9.33969863	0	7.871803	5.839953	6.648138	5.995415	10.725271	1.050621583	high
GSM3818036	9.147945205	0	8.721359	6.361917	6.839252	6.989091	6.637631	0.940001559	low
GSM3818037	9.375369863	0	8.955465	5.808032	7.386994	8.351961	4.17857	0.839559741	low
GSM3818039	1.479452055	1	8.794263	8.409363	6.562328	9.617484	6.390392	0.765661318	low
GSM3818040	6.852082192	0	6.447948	5.600711	6.98237	5.846493	4.541434	0.777177168	low
GSM3818041	8.043863014	0	8.713766	6.299483	6.506084	9.312929	4.634124	0.874892128	low
GSM3818042	7.249315068	0	6.750649	5.472896	6.910579	5.90551	6.053507	0.843230984	low
GSM3818043	7.572575342	0	7.955917	7.705463	5.112688	7.842372	4.388413	0.927069378	low
GSM3818045	8.093178082	0	8.002576	5.115635	5.863257	7.11033	8.836628	1.160113477	high
GSM3818046	4.260246575	0	8.873904	5.776583	6.738836	5.631669	4.458661	1.007852601	high
GSM3818047	8.054794521	0	7.297011	5.396598	6.107968	8.228746	5.606086	0.917322132	low
GSM3818050	4.175342466	0	7.621153	4.987006	6.42217	11.500162	4.289074	0.813522833	low
GSM3818055	8.879424658	0	7.917786	5.935371	5.878308	5.649655	4.722604	1.030440871	high
GSM3818056	10.28769863	0	7.462038	5.268541	5.985004	5.372583	4.376006	1.024145455	high
GSM3818057	9.608219178	0	7.054615	5.420875	6.092867	6.045036	4.750133	0.945302661	low
GSM3818059	10.18356164	0	7.913034	5.746024	6.174161	5.907735	5.164405	1.003942169	high
GSM3818060	5.265780822	1	8.49086	6.856559	5.600336	10.751531	10.294618	1.01025601	high
GSM3818063	8.589041096	0	8.370775	5.563854	6.158278	5.493666	5.479164	1.089048199	high
GSM3818069	9.454767123	0	9.702484	5.800849	6.20928	7.729549	13.00926	1.329740464	high
GSM3818072	2.041068493	0	8.291515	5.563587	6.165076	6.688186	6.103955	1.052532243	high
GSM3818074	9.86030137	0	8.35635	5.394125	6.168899	6.372978	10.143863	1.188019389	high
GSM3818075	9.345205479	0	7.441043	5.133997	5.981287	5.494527	6.279751	1.074608437	high
GSM3818076	8.597260274	0	9.078011	6.377758	6.422408	6.133236	12.548102	1.212622956	high
GSM3818077	4.326	1	9.244795	5.480697	6.232721	5.69137	7.24265	1.220203288	high
GSM3818078	9.098630137	0	7.038356	5.64633	6.140746	8.707621	7.061577	0.888699463	low
GSM3818080	9.542465753	0	6.561257	6.025709	6.039237	8.681698	6.811485	0.832079254	low
GSM3818081	8.556164384	1	8.353776	5.727524	6.340216	5.606737	6.290566	1.061910955	high
GSM3818091	9.249287671	0	8.593841	6.129773	5.568802	5.947191	5.337638	1.136372814	high
GSM3818094	9.956136986	1	7.495378	5.977252	6.104923	7.075006	4.962176	0.915890348	low
GSM3818096	8.630136986	0	10.74538	5.643072	5.530404	6.027917	7.463286	1.527952693	high
GSM3818098	11.18358904	0	8.978813	4.882494	6.163725	6.708239	8.418227	1.248817542	high
GSM3818101	11.26027397	0	9.004379	6.325596	6.222137	5.944856	4.735814	1.047771691	high
GSM3818102	10.81643836	0	7.001423	5.613067	6.249804	6.644176	5.683648	0.907515658	low
GSM3818107	11.08216438	0	9.230436	6.095775	6.343163	5.612117	9.000535	1.193802614	high
GSM3818109	3.728794521	0	10.420467	5.885298	6.230394	5.961671	10.52944	1.415038011	high
GSM3818111	10.37531507	0	8.173718	5.876501	6.040531	8.740864	5.453373	0.951390224	low
GSM3818113	11.44380822	0	8.03607	5.956249	6.137246	5.612222	4.908234	1.008803837	high
GSM3818115	10.33972603	0	6.418364	6.268334	5.99818	5.946009	5.249757	0.856347631	low
GSM3818116	4.36709589	1	8.187031	5.442951	7.385757	6.008605	10.773763	1.007223774	high
GSM3818117	11.37534247	0	8.312011	5.792475	5.71085	9.071415	5.841906	1.014376927	high
GSM3818118	0.430109589	1	7.768838	6.804599	5.966727	9.028315	4.538281	0.834835335	low
GSM3818121	11.09317808	0	8.978232	5.861162	5.44572	7.166673	9.670034	1.29808603	high
GSM3818122	11.6849589	0	9.379563	5.927692	6.473819	5.714174	5.064885	1.097762058	high
GSM3818125	11.21917808	0	9.030233	5.697507	5.669763	6.139069	11.332156	1.375652179	high
GSM3818127	11.38356164	0	6.692347	5.276894	5.660406	7.032291	4.556832	0.945197642	low
GSM3818131	11.46846575	0	7.371652	5.544277	5.304807	5.471824	7.881669	1.180605305	high

GSM3818137	10.91778082	0	10.596281	5.327485	6.037133	5.603519	6.745694	1.433834021	high
GSM3818138	10.70136986	0	7.236191	5.964679	6.622285	8.332761	4.677037	0.792100075	low
GSM3818139	10.30956164	0	8.604107	4.592521	6.114328	6.677217	6.302427	1.183381676	high
GSM3818141	3.819205479	1	8.174819	4.995857	5.451969	6.453843	5.616689	1.198390543	high
GSM3818144	11.2000274	0	9.027532	5.277467	6.338264	4.690953	11.100104	1.349620912	high
GSM3818146	11.49312329	0	7.788741	7.047087	6.776256	7.661209	8.646082	0.841503224	low
GSM3818150	0.389013699	0	7.240973	5.627246	6.079746	10.577912	4.859546	0.818218396	low
GSM3818157	7.326	0	8.517657	6.344205	5.568802	5.506566	4.094265	1.094534958	high
GSM3818160	12.36435616	0	7.652177	8.365031	5.151508	9.921005	8.301079	0.867928689	low
GSM3818161	1.276684932	1	9.672125	7.266981	5.158423	5.293972	12.961071	1.486967778	high
GSM3818162	10.97810959	0	6.415819	6.833609	5.343915	5.434357	5.071246	0.910407167	low
GSM3818163	12.29038356	0	7.959796	7.141381	5.44073	13.540183	4.700793	0.770765339	low
GSM3818165	12.00550685	0	9.401583	5.913246	6.966535	5.253695	11.394101	1.204712257	high
GSM3818168	11.29865753	0	8.817585	6.539629	6.019664	5.473788	4.321226	1.048004306	high
GSM3818172	8.668520548	0	6.633668	6.682734	5.673139	4.841616	5.144608	0.91740808	low
GSM3818173	10.82736986	0	7.102762	5.69554	6.100933	5.867926	5.990171	0.960892853	low
GSM3818174	1.789068493	1	8.166778	6.300542	5.594257	5.508659	12.815569	1.289682807	high
GSM3818178	7.827369863	0	9.668877	5.404546	6.486515	5.288449	8.328076	1.281592843	high
GSM3818179	10.79728767	0	7.603464	5.78048	6.608942	10.323231	7.494957	0.832183469	low
GSM3818180	2.501342466	0	9.992753	6.219555	5.654962	5.871454	8.907131	1.387205317	high
GSM3818184	12.48764384	0	10.366708	5.818186	6.429207	6.086782	4.118998	1.18449664	high
GSM3818189	12.08490411	0	8.323298	5.393645	6.628949	6.780322	4.803843	0.969898538	low
GSM3818190	10.95065753	0	8.025807	5.816585	6.491089	6.619201	5.402599	0.947872467	low
GSM3818193	5.884931507	1	8.780557	5.801398	6.727959	5.532443	10.469202	1.148342608	high
GSM3818195	12.0630411	0	6.581573	5.668438	6.720594	8.720332	8.302956	0.804408602	low
GSM3818200	12.30961644	0	7.817845	5.682849	6.779805	6.137767	4.061168	0.888338322	low
GSM3818202	11.6109863	0	9.027106	5.384323	4.996703	5.710737	9.922656	1.521732074	high
GSM3818206	12.00550685	0	9.370294	6.009734	5.113685	5.870027	3.690076	1.272806645	high
GSM3818208	10.83838356	1	8.352167	5.199122	6.546943	6.452531	8.300253	1.093246387	high
GSM3818211	9.783534247	1	7.866735	5.26514	6.752258	5.046077	4.833215	0.976585014	low
GSM3818213	5.13969863	0	7.189171	6.529813	5.00429	5.749526	8.608809	1.12967001	high
GSM3818219	7.317780822	1	9.701923	6.59193	5.752248	5.749996	8.372134	1.282305734	high
GSM3818220	9.290383562	0	8.356983	5.459793	7.071693	5.861232	4.564297	0.93269635	low
GSM3818223	12.33427397	0	9.438942	5.438416	6.55898	5.430388	9.827997	1.274394972	high
GSM3818224	4.008246575	0	6.461958	4.505109	7.110742	5.331749	4.486143	0.845077026	low
GSM3818225	12.27123288	0	6.617281	5.42735	7.102872	5.813441	8.048643	0.853383871	low
GSM3818228	11.78079452	0	7.721667	4.688103	5.48369	5.708397	4.107644	1.158453858	high
GSM3818229	5.073945205	0	7.19487	5.646283	6.174392	6.054197	5.516434	0.946945414	low
GSM3818230	6.435616438	0	6.957779	6.397569	6.256894	6.194695	7.090488	0.890532765	low
GSM3818235	6.498657534	0	7.833905	5.765694	6.888972	6.504439	5.314531	0.884843707	low
GSM3818237	5.909589041	0	8.08492	5.626023	6.075422	6.128944	11.196502	1.189347987	high
GSM3818241	5.495917808	0	6.743281	6.680159	6.971423	5.751867	5.311515	0.752161105	low
GSM3818242	5.884931507	0	7.824459	6.089829	7.209074	5.198356	5.53646	0.865099472	low
GSM3818243	6.446547945	0	6.23369	6.316226	7.117411	6.534467	4.567568	0.691200052	low
GSM3818244	7.326	0	8.004277	5.998222	6.716719	10.627545	4.77475	0.779587533	low
GSM3818245	6.42739726	0	9.778349	5.367886	6.352672	6.098356	4.623601	1.184395652	high
GSM3818247	6.526027397	0	7.637147	5.734704	6.409793	9.661821	6.975861	0.870519813	low
GSM3818248	6.024657534	0	6.113661	6.016155	7.205142	13.56129	5.831942	0.56269276	low
GSM3818249	6.035589041	0	9.60464	5.791493	6.433555	6.183912	11.563164	1.301107622	high
GSM3818251	6.42739726	0	7.114077	6.335964	6.938509	7.330986	4.346403	0.746566866	low
GSM3818252	5.926027397	0	7.456631	6.09051	6.425095	9.335481	9.92107	0.897688411	low
GSM3818253	1.235589041	0	6.99155	7.787377	6.97015	6.378145	3.870887	0.670468735	low
GSM3818254	6.312328767	0	7.902042	6.108285	6.97806	5.49359	5.354454	0.886684876	low
GSM3818257	6.263013699	0	9.135904	5.635121	6.414494	9.373273	9.926007	1.093647681	high
GSM3818259	6.208191781	0	8.551602	8.703667	7.116697	6.158124	4.848329	0.732533634	low
GSM3818260	6.991808219	0	7.014971	6.261947	6.608615	10.016234	10.35574	0.816787629	low
GSM3818262	6.293178082	0	7.191928	6.671752	6.865289	6.579645	5.984808	0.788068422	low
GSM3818263	5.523287671	1	9.996018	5.01759	5.930851	5.790101	8.108421	1.442162664	high

GSM3818265	6.202767123	0	5.664385	6.665292	5.921833	6.714532	5.069584	0.757795842	low
GSM3818268	7.093150685	0	7.941164	6.074137	6.34766	8.826144	11.141709	0.995647458	high
GSM3818270	7.50139726	0	7.996001	5.664227	7.58909	7.010572	4.780299	0.797339373	low
GSM3818271	7.24660274	0	8.020799	5.732857	7.113919	7.643296	5.361061	0.843420081	low
GSM3818272	2.931534247	0	10.59912	9.726321	4.810461	5.493118	10.603734	1.329731589	high
GSM3818273	5.605479452	0	7.871254	7.131271	6.938048	9.901692	5.071416	0.704940995	low
GSM3818277	7.074	0	8.428471	5.360538	7.316125	6.474832	10.512637	1.025536785	high
GSM3818278	6.019150685	0	9.047523	6.039658	6.988119	6.397348	5.126722	0.95967853	low
GSM3818279	7.24109589	0	9.86321	5.329252	6.560095	5.909914	4.844176	1.176399355	high
GSM3818281	6.635589041	0	6.946524	5.958331	6.752136	6.035762	5.88969	0.839575457	low
GSM3818282	6.153452055	0	6.781798	6.375663	6.100341	6.53909	5.457182	0.854083607	low
GSM3818287	5.008191781	0	5.969434	5.844065	6.591304	10.793257	6.551426	0.683339898	low
GSM3818289	7.120520548	0	7.479181	6.153919	6.626721	12.239423	11.398621	0.817051604	low
GSM3818290	6.460273973	0	6.749095	7.216843	6.600986	6.248473	5.101523	0.744678379	low
GSM3818294	2.934246575	0	7.624461	5.269187	5.833651	6.454922	6.580906	1.077780057	high
GSM3818298	10.10408219	0	7.270043	6.092603	6.752583	5.711772	3.96866	0.829462113	low
GSM3818301	9.989013699	1	9.189615	5.042823	6.624696	9.814819	5.696758	1.00004997	high
GSM3818303	10.98632877	0	6.717407	5.395771	6.592045	7.265575	5.986935	0.843976679	low
GSM3818307	4.567150685	0	7.845623	8.014031	6.919357	5.510162	5.462484	0.768938124	low
GSM3818310	5.180794521	0	7.910562	4.970433	5.479178	8.694342	3.900582	1.040941479	high
GSM3818311	5.041068493	0	8.306226	5.815131	5.821005	6.903302	8.868209	1.147193125	high
GSM3818316	2.706821918	0	8.508659	5.767871	5.706115	5.120837	10.955654	1.327614473	high
GSM3818317	5.452027397	0	7.296783	5.400136	5.653249	5.146477	7.374832	1.127675285	high
GSM4947169	7.4	0	5.130626	6.42287	6.160766	11.132795	6.347268	0.630252069	low
GSM4947174	1.1	1	9.576138	6.179377	5.760528	8.093314	7.084053	1.173553617	high
GSM4947176	5.2	0	8.771216	6.59068	5.79668	6.653228	9.453707	1.158694919	high
GSM4947179	2.7	1	11.303008	3.617861	5.778934	5.443827	4.801587	1.74729158	high
GSM4947181	7.4	0	8.836159	5.40114	5.96008	4.487914	12.032647	1.423382633	high
GSM4947187	1.9	1	9.298769	4.514464	6.00212	5.828135	9.409531	1.427661476	high
GSM4947190	7.3	0	8.878491	4.952363	5.806111	6.714568	5.187714	1.201876156	high
GSM4947194	5.9	0	6.592062	6.877033	6.073243	7.453636	5.104363	0.779296558	low
GSM4947196	5.9	1	5.567761	7.04363	6.278935	7.947326	8.389932	0.717678233	low
GSM4947200	7.8	0	8.814909	5.680208	5.731897	5.15482	4.044813	1.171027413	high
GSM4947202	3.4	1	6.486097	6.503417	6.276306	9.587638	9.048196	0.786099113	low
GSM4947205	3.7	1	6.208285	6.738887	5.837584	11.559517	8.429453	0.738244611	low
GSM4947207	6.8	0	8.682429	5.058484	6.314945	7.65076	5.153163	1.054621794	high
GSM4947209	6.7	0	9.581334	4.139968	6.532206	6.263522	5.403776	1.261160779	high
GSM4947210	6.8	0	10.14947	4.537011	5.863411	2.629129	11.478954	1.839093657	high
GSM4947212	5.8	0	9.502307	4.540603	6.166222	7.609956	4.832203	1.205938835	high
GSM4947214	5	0	8.255785	5.245194	6.868448	4.7493	6.432679	1.046246566	high
GSM4947220	6.7	0	6.406361	9.246445	6.175672	8.840786	8.253682	0.644492411	low
GSM4947232	6.6	0	8.973516	6.013073	6.540657	5.994469	4.147187	1.007965946	high
GSM4947234	6.3	0	9.740571	4.975224	6.480383	7.331892	8.002761	1.238248746	high
GSM4947237	5.9	0	8.860208	4.362057	7.968863	8.135815	4.107928	0.861781737	low
GSM4947239	6.4	0	7.681389	7.211614	7.11409	9.468346	5.788998	0.691952684	low
GSM4947243	5.5	0	7.527606	8.247267	7.000912	11.114673	4.813555	0.592125243	low
GSM4947244	6.4	0	8.7186817	4.9149733	5.5449358	4.1566701	4.271121	1.313096278	high
GSM4947245	5.9	0	9.419249	5.187776	5.822071	4.966842	9.700764	1.456837519	high
GSM4947248	5.5	1	7.519387	4.640723	6.434874	6.059382	9.499022	1.114144789	high
GSM4947249	4.8	0	9.061133	5.331777	5.659441	4.744804	4.201642	1.266104231	high
GSM4947250	5.5	0	7.592346	6.823096	6.524036	9.379216	4.231862	0.74370575	low
GSM4947251	6.3	0	7.391416	8.308298	7.014668	6.839556	11.323357	0.775904704	low
GSM4947254	5.9	0	10.01311	4.889422	5.065319	6.918683	4.787144	1.471995896	high
GSM4947256	4.6	0	6.16685	5.880435	6.480383	5.189618	4.340145	0.808307019	low
GSM4947259	5.3	0	6.597302	6.418742	6.274443	10.088116	3.967911	0.701009536	low
GSM4947260	9.4	1	9.323169	5.077071	6.929347	6.346335	8.378553	1.154479316	high
GSM4947262	5.5	1	9.19465	5.302665	6.234834	7.350216	10.072131	1.242058301	high
GSM4947263	5	0	8.582475	6.040167	7.477804	5.114297	7.516924	0.943612398	low

GSM4947264	6.2	0	7.080053	7.107009	7.218125	9.300384	5.99852	0.655438701	low
GSM4947265	2.4	1	6.670937	6.057706	6.64306	7.471894	4.183463	0.75557718	low
GSM4947269	3.3	0	8.658602	6.349527	5.691524	5.082839	9.993083	1.26409531	high
GSM4947270	0.9	0	10.522226	5.016453	6.679055	6.800846	10.725643	1.401344044	high
GSM4947272	5.9	0	9.614311	4.420322	7.147314	4.272801	5.25964	1.209042975	high
GSM4947274	4.7	0	8.625815	6.289465	6.467992	5.69395	3.838177	0.966827682	low
GSM4947275	5.5	0	7.691583	6.500279	7.160232	7.52272	4.440672	0.752205916	low
GSM4947276	2.3	1	6.45194	6.405108	5.997802	6.348986	6.706803	0.867062483	low
GSM4947277	5.7	0	7.487281	6.599686	6.369659	10.261241	9.12471	0.830787093	low
GSM4947279	4.4	0	8.854243	5.011835	5.773549	6.804905	7.898785	1.27142853	high
GSM4947280	5.6	0	8.177737	5.802413	6.68612	8.815355	4.121746	0.845858246	low
GSM4947281	0.8	0	8.110713	6.51896	5.770233	6.391332	10.779293	1.140769032	high
GSM4947283	5.9	1	8.879479	5.86986	7.21346	6.241957	4.846671	0.92571839	low
GSM4947284	4.3	0	9.167939	5.188638	6.028518	4.863273	7.769398	1.32636439	high
GSM4947286	0.8	0	9.917751	4.933311	6.051678	3.126449	11.76953	1.681791516	high
GSM4947287	3.6	1	9.321904	5.615445	5.665406	6.532311	9.858696	1.359946315	high
GSM4947289	3.9	0	9.485939	6.787833	6.676297	8.082382	7.271407	0.980151345	low
GSM4947290	2.8	1	8.030953	6.525645	7.387131	6.17193	4.039632	0.778693378	low
GSM4947291	5.3	0	9.043514	5.599014	6.540841	9.123458	5.520965	0.97414738	low
GSM4947293	0.8	0	5.901628	7.576424	7.257553	9.113908	7.895341	0.589434776	low
GSM4947296	6	0	8.958832	4.39158	5.069701	7.16113	10.649315	1.565098644	high
GSM519117	7	0	7.999279	5.405911	6.3258	4.944748	8.40241	1.130619354	high
GSM519118	7.4	0	7.840352	7.526274	6.779846	8.964959	9.17732	0.789469666	low
GSM519119	7.2	0	7.163677	6.693705	6.328796	7.885897	4.643078	0.785887779	low
GSM519120	6.3	0	8.582478	5.077607	6.477828	6.229674	5.729918	1.082729505	high
GSM519121	7	0	8.271661	5.370143	6.786865	4.097496	10.769987	1.18390395	high
GSM519128	6.9	0	7.134708	7.160047	6.876419	4.597753	6.52236	0.814767418	low
GSM519131	7	0	7.648521	6.617157	6.597461	9.182388	4.944761	0.769216154	low
GSM519133	1.8	1	8.056692	5.77226	7.512436	7.012158	5.97387	0.826050451	low
GSM519140	6.8	0	8.886064	6.015928	6.326811	7.683655	4.852715	0.989323066	low
GSM519143	5.4	0	8.354947	6.976258	6.454413	6.790449	5.959086	0.905506264	low
GSM519144	1.2	1	8.327187	5.955655	5.773578	5.346347	6.173394	1.133946879	high
GSM519145	5.8	1	8.88243	5.545152	6.633971	5.067589	2.9327	1.025700917	high
GSM519148	6.9	0	6.686754	6.835032	6.689834	6.75633	6.787953	0.769201058	low
GSM519153	3.7	1	10.257832	5.494874	5.685284	7.712892	5.546065	1.30596989	high
GSM519154	0.8	1	9.867754	3.430579	5.523147	6.861422	11.338497	1.771055257	high
GSM519157	6	0	8.102327	7.250686	7.456867	5.889363	2.902743	0.722148452	low
GSM519158	6.3	0	8.527701	6.553843	6.010303	6.198851	7.130794	1.060657765	high
GSM519160	5.1	0	7.355802	9.003931	6.703812	7.733857	4.567231	0.637392465	low
GSM519162	5.9	0	9.405977	5.649858	5.305343	9.54882	5.444794	1.177039712	high
GSM519163	5.4	0	8.346807	7.114203	5.438276	3.742765	10.16771	1.257883658	high
GSM519164	5.9	0	6.650658	7.320718	6.299851	11.338816	4.716197	0.6389493	low
GSM519165	6.4	0	9.421149	5.268461	6.193364	5.352329	9.97833	1.365337512	high
GSM519166	2.3	1	8.803688	7.02197	5.619397	6.881216	4.414297	1.020187888	high
GSM519170	6.5	0	9.826203	5.485388	6.910026	7.409411	7.025085	1.102110644	high
GSM519171	6.9	0	7.984778	6.349124	6.653679	9.682478	4.938561	0.791404846	low
GSM519172	1.8	1	8.932061	5.461919	5.980932	3.186425	9.30298	1.399202656	high
GSM519174	6.8	0	7.309302	6.569051	6.668024	8.60612	4.490268	0.746409126	low
GSM519177	6.6	0	8.911725	6.597969	5.966931	7.521238	8.209444	1.082373377	high
GSM519179	4.9	0	8.792202	6.487922	6.9384	6.286193	6.096241	0.934567527	low
GSM519184	5	0	8.382662	7.311755	6.430298	8.83544	10.575584	0.921080953	low
GSM519187	6.5	0	9.891417	5.507029	5.948567	6.609047	4.046819	1.216800423	high
GSM519188	6.8	0	5.572904	4.731604	5.846046	8.897054	1.033311	0.74795354	low
GSM519189	6.4	0	8.457097	5.650178	6.455738	6.27864	5.18439	1.012328417	high
GSM519192	6.5	0	6.719765	6.759455	6.719017	8.473453	5.549814	0.7098295	low
GSM519193	4	1	9.5348	5.410407	5.112752	7.34379	9.299973	1.465189366	high
GSM519196	2.3	1	8.391844	5.902182	7.09502	3.99544	10.790474	1.104901957	high
GSM519197	13.8	0	8.481707	5.901869	6.104222	9.223377	8.646142	1.025768315	high

GSM519198	13.8	0	9.171849	7.728087	5.937913	6.267369	7.001782	1.035912636	high
GSM519199	14.1	0	9.111401	6.059178	6.693799	4.205709	5.66827	1.094332458	high
GSM519201	13.2	0	8.864704	5.868979	6.086159	8.475912	9.487242	1.117872826	high
GSM519204	2.9	1	8.068836	6.06015	6.211936	8.865468	10.328781	1.008403498	high
GSM519205	11.6	0	8.263765	7.692234	6.63729	7.552324	6.962198	0.825584595	low
GSM519206	11.5	0	5.17897	7.09689	6.305325	10.060057	5.348604	0.596650203	low
GSM519211	11.6	0	8.868305	3.670679	6.895847	9.222341	5.270385	1.047992847	high
GSM519214	8.3	1	7.674216	9.412818	6.432956	6.103172	1.273824	0.648102081	low
GSM519217	1.3	1	9.865859	4.105958	6.657108	9.452892	11.013405	1.304620422	high
GSM519218	12.2	0	9.902939	3.631135	6.312637	2.777352	9.110055	1.704774808	high
GSM519219	8.1	0	6.570517	6.136942	6.810985	11.21896	4.45257	0.645180593	low
GSM519220	11	0	7.003851	5.969091	6.556534	10.135352	4.911248	0.739950884	low
GSM519226	9.8	0	7.179867	7.149374	6.342353	9.551989	3.816948	0.704218237	low
GSM519230	12.9	0	7.76241	5.708978	6.384085	9.074281	5.905976	0.881834297	low
GSM519231	12.7	0	9.378857	5.887982	6.535704	6.786403	9.494417	1.164806959	high
GSM519234	10.9	0	8.43138	5.490115	6.876758	2.212477	5.645082	1.114621054	high
GSM519235	2.5	1	9.138937	5.032821	6.046016	7.99408	9.579919	1.253883066	high
GSM519236	2	1	5.222816	6.384204	6.358435	8.951492	4.428158	0.638472295	low
GSM519237	1.2	1	9.321405	2.572317	6.978056	3.340756	8.405831	1.538288288	high
GSM519240	12.3	0	7.098966	6.683874	5.902959	10.074799	6.402158	0.803120643	low
GSM519242	11.1	0	8.108869	7.381534	6.580493	8.847981	4.493318	0.760493901	low
GSM519243	10.8	0	9.562058	5.071899	6.378625	4.003186	11.039184	1.466746072	high
GSM519245	7	1	9.197124	4.369605	6.223472	5.296696	4.76437	1.269165981	high
GSM519248	5.9	1	8.098194	5.727352	6.988737	9.19692	7.031533	0.853446428	low
GSM519253	2.4	1	8.130784	5.435014	6.375592	4.168792	4.309162	1.060746151	high
GSM519260	12.5	0	7.170877	7.256408	6.896181	11.458097	2.353798	0.5859876	low
GSM519261	12.5	0	8.495755	5.535331	6.894515	7.02377	4.341103	0.922284585	low
GSM519262	9.9	0	8.759192	5.771267	5.320495	8.677337	9.753919	1.241132768	high
GSM519263	2.9	1	8.104717	6.588403	5.887279	10.981087	9.430402	0.928661588	low
GSM519265	5.7	1	9.370775	3.310414	5.177236	2.740937	12.329974	2.098496569	high
GSM519267	13.1	0	8.992098	5.877661	6.499709	3.928329	9.68569	1.246725341	high
GSM519268	11.1	0	7.056833	7.790654	5.927728	7.670003	8.037683	0.822702067	low
GSM519270	12.1	0	7.84669	5.775897	6.607825	8.020155	9.844244	0.970673856	low
GSM519275	10.2	0	8.633012	6.11142	6.612106	6.160682	6.879976	1.014110154	high
GSM519276	12.4	0	8.936037	6.376431	6.572766	6.659603	8.142854	1.041235408	high
GSM519277	7.4	0	9.906805	5.537221	6.413891	9.38311	10.431738	1.20041298	high
GSM519279	7.5	1	6.498565	3.927433	6.169186	7.643915	11.879876	1.109146616	high
GSM519283	10.3	0	8.186139	9.486198	6.549823	9.503526	4.053878	0.63357328	low
GSM519285	11.5	1	9.760392	5.666585	6.011694	9.112228	5.693362	1.123609786	high
GSM519286	10.5	0	7.807298	5.94136	6.548784	6.802521	4.760969	0.893107874	low
GSM519287	11.7	0	8.374347	6.289873	6.356706	8.851323	5.090706	0.887921624	low
GSM519290	10	0	8.544953	6.419327	6.741941	9.029204	6.581259	0.870459472	low
GSM519292	12.5	0	9.096209	5.916261	5.948567	5.431302	10.222885	1.306621953	high
GSM519294	10.2	0	9.902939	5.291869	6.068354	7.063347	5.530047	1.240703823	high
GSM519296	11.2	0	9.560547	3.803014	5.482839	8.453959	10.361467	1.558464084	high
GSM519301	10	0	9.341905	5.428321	6.383402	3.828783	7.292035	1.289509715	high
GSM519302	11.5	0	8.588784	6.035887	6.324081	7.089677	5.340872	0.990335466	low
GSM519304	2.1	1	10.830158	3.933468	6.64201	3.07944	12.52888	1.860286188	high
GSM519305	1.8	1	8.731354	4.580853	6.201215	7.474031	11.879876	1.309377331	high
GSM519306	5.8	1	7.352395	7.201502	6.402351	8.584696	5.501439	0.758764094	low
GSM519311	9.5	0	7.630973	6.726847	5.985997	8.325207	3.852134	0.832990113	low
GSM519313	11.4	0	10.89952	5.682287	6.538356	7.413344	5.734799	1.231729714	high
GSM519315	10.4	0	9.846781	5.375501	5.42164	6.244497	8.328143	1.470676432	high
GSM519316	11	0	6.142567	7.530582	6.717443	7.700172	4.56159	0.634782028	low
GSM519318	6	0	8.611688	4.315015	6.57203	3.787813	10.292778	1.367286909	high
GSM519320	11	0	8.317122	7.109386	6.399263	9.06296	5.979796	0.834832938	low
GSM519321	11.2	0	9.06422	5.546123	5.965562	6.808664	10.592772	1.288089493	high
GSM519324	4.7	1	6.853554	4.941267	5.872233	3.661255	9.750903	1.203701135	high

GSM519327	6.2	1	9.452979	5.142274	6.156566	6.332707	7.182406	1.262716763	high
GSM519330	10.2	0	6.588122	6.476942	7.655235	8.814854	7.811996	0.653484746	low
GSM519339	11	0	7.514045	6.242882	7.490768	6.400615	6.96683	0.791500063	low
GSM519341	10.8	0	9.46887	5.632111	7.11767	7.170373	8.077445	1.055536084	high
GSM519345	9.2	0	9.608923	6.281504	5.799264	9.099823	4.350397	1.05576038	high
GSM519346	10.6	0	9.582439	4.77036	6.672698	4.467555	11.039184	1.421105639	high
GSM519351	7.9	0	7.227504	7.171538	7.39538	6.5171	6.501133	0.715903951	low
GSM519352	8.1	1	6.912463	5.227197	5.491451	5.412243	4.846232	1.054381132	high
GSM519354	9.4	0	7.810398	7.457458	6.72727	5.374125	5.896996	0.833859433	low
GSM519356	10.2	0	5.816008	7.160656	6.665066	7.036513	3.985001	0.643366364	low
GSM519357	5.3	1	7.523742	5.367909	5.130836	7.937168	3.348802	1.034414293	high
GSM519358	8.9	0	8.016764	4.415362	5.809966	11.058005	9.704177	1.105085703	high
GSM519360	9	0	9.721649	5.79411	7.254046	7.572224	7.134869	1.01198634	high
GSM519364	10.3	0	6.74628	5.833856	5.754321	5.992666	3.846183	0.914854047	low
GSM519365	9.6	0	7.627666	7.471422	6.611722	9.873001	7.810877	0.748092537	low
GSM519366	10.1	0	6.895787	4.785321	6.548784	8.720354	4.650462	0.837002162	low
GSM519368	3.4	1	8.256585	5.820934	6.279378	7.809782	4.277041	0.935229365	low
GSM519369	10.4	0	9.941031	5.547364	5.877345	4.821272	4.860908	1.330929296	high
GSM519371	7.5	1	8.410239	7.199003	6.919324	7.580525	5.436405	0.806718716	low
GSM519373	7.2	0	8.879486	4.923621	6.094681	6.830871	2.682124	1.088437925	high
GSM519374	10.1	0	7.857257	6.314464	7.588865	3.348914	7.896716	0.907133288	low
GSM519376	9.4	0	9.991479	5.112269	6.054668	5.139492	10.266783	1.509279603	high
GSM519377	10.2	0	8.826828	6.183507	5.742811	7.384306	10.562559	1.212378375	high
GSM519381	9.9	0	8.943717	6.179198	6.539001	6.621457	5.197294	0.995484375	high
GSM519384	8.3	0	9.022381	6.09694	5.803721	8.184551	5.864896	1.07916238	high
GSM519385	9.8	0	8.196333	6.408847	5.977928	7.393916	5.765156	0.972314443	low
GSM519386	10.4	0	9.209257	5.568887	6.837287	6.905182	9.30097	1.116300267	high
GSM519387	8.9	0	7.621057	5.7864	5.404728	5.005047	8.370133	1.201918799	high
GSM519389	9.5	0	8.043255	5.999157	7.030591	7.09913	4.568389	0.837885686	low
GSM519390	9.6	0	6.582921	5.446686	7.195149	5.771671	4.777955	0.77935696	low
GSM519394	9.4	0	7.990144	6.671303	5.991868	6.608482	7.114322	0.98667185	low
GSM519399	9.1	0	9.400738	6.158724	5.651764	6.374787	10.568904	1.344980283	high
GSM519400	3.3	1	9.373599	5.071278	6.552155	2.533853	6.916044	1.343991753	high
GSM519402	8.1	0	7.18109	6.648947	6.514942	6.229096	1.714542	0.76072779	low
GSM519404	9.1	0	7.620048	6.678084	5.840732	7.441464	9.762949	1.004158331	high
GSM519408	7.7	0	7.017889	7.547741	7.039402	3.357701	5.580487	0.779431391	low
GSM519409	8.7	0	6.372315	6.46113	6.077674	6.811493	5.840846	0.817945213	low
GSM519410	8.1	0	9.835809	4.737182	6.606865	3.409903	4.417025	1.313103138	high
GSM519411	8.6	0	8.630527	5.399717	5.976509	8.257234	4.412475	1.033029231	high
GSM519413	7.8	0	6.938806	6.520115	6.704153	8.604745	8.747756	0.792372912	low
GSM519417	2.4	1	8.845098	6.929815	5.380004	5.04411	6.669569	1.193826202	high
GSM519423	8	0	8.301389	5.687146	6.364864	8.6611	3.55321	0.896631987	low
GSM519426	8.1	0	8.479525	5.220943	6.685507	9.042693	4.846535	0.919072379	low
GSM519427	8.5	0	5.810145	7.649403	7.079068	6.863606	4.457191	0.593806369	low
GSM519428	8.1	0	7.88637	6.312547	6.32942	5.187907	1.900102	0.89180216	low
GSM519429	6.4	0	5.41309	6.205236	7.179235	8.833862	8.298913	0.643867262	low
GSM519430	1.5	0	6.926697	7.693877	5.110219	7.12018	9.186032	0.960126934	low
GSM519431	6.8	1	7.329988	7.013597	6.222456	7.78359	10.364283	0.903730749	low
GSM519432	6.7	0	8.394564	7.452607	6.843235	7.373663	5.26925	0.800849541	low
GSM519435	5.8	0	9.326541	6.064779	5.468362	5.822038	4.879857	1.235433048	high
GSM519439	4.7	0	7.709753	6.651931	6.189313	10.921819	9.60293	0.857563743	low
GSM519440	4.1	0	6.810703	5.641135	6.067655	9.918324	5.755823	0.819399716	low
GSM519442	3.8	0	7.181487	7.227348	5.147527	10.102523	2.54929	0.790310468	low
GSM519443	8.1	0	6.884974	7.192948	5.416558	9.39079	4.864573	0.800209407	low
GSM519722	8.28	0	8.313527	4.685375	6.943439	5.348857	9.062653	1.131203779	high
GSM519724	8.64	0	8.160526	5.861717	5.053355	5.304582	9.721777	1.350362009	high
GSM519725	8.4	0	8.30693	6.367394	5.94827	11.560144	5.719124	0.86103198	low
GSM519726	7.21	0	9.200353	5.981197	5.845642	5.245576	4.227331	1.169882911	high

GSM519727	8.19	0	8.480436	5.145247	5.192623	4.153641	8.160914	1.447374045	high
GSM519728	2.52	1	8.175811	5.52803	6.89688	8.916417	10.706974	0.970637384	low
GSM519729	8.32	0	9.046313	3.792946	6.572786	4.601118	10.140228	1.439716561	high
GSM519730	8.82	0	8.876143	6.433771	6.071644	7.651779	6.43322	1.029376887	high
GSM519731	9.03	0	9.028567	5.954611	5.898365	9.135454	4.537335	1.012734321	high
GSM519734	9.45	0	9.207048	4.652764	6.423267	4.531476	9.348538	1.37563567	high
GSM519736	6.07	0	5.035963	8.434533	7.618643	7.268974	3.541104	0.464492192	low
GSM519739	8.03	0	8.377978	5.551327	6.16687	5.129638	5.443809	1.101924421	high
GSM519740	1.85	0	8.680359	4.961965	5.738637	8.395016	4.512123	1.107724219	high
GSM519742	5.72	0	8.583215	5.881036	6.3243	7.335712	8.978796	1.079144735	high
GSM519744	4.48	0	8.601116	4.70616	6.553476	5.503317	11.321884	1.284725731	high
GSM519750	8.39	0	9.526695	4.983562	5.91402	5.028436	7.812255	1.411266382	high
GSM519752	1.52	1	8.60698	5.261769	5.985811	3.687037	3.908763	1.197341966	high
GSM519753	7.16	0	8.383894	6.315856	7.416021	8.568047	5.204291	0.773224536	low
GSM519754	7.23	0	8.148613	5.51251	6.917649	10.307066	10.632288	0.921184984	low
GSM519755	6.71	0	9.164041	5.398158	6.255674	8.834205	8.90034	1.136017737	high
GSM519757	6.55	0	8.027108	5.974313	5.773798	7.180378	10.425339	1.139885981	high
GSM519760	6.21	0	9.824622	3.81672	6.292438	4.490448	5.673134	1.461293541	high
GSM519761	5.56	1	6.935276	8.316961	6.255114	5.383587	4.766093	0.746792783	low
GSM519764	6.45	1	6.812435	9.094495	6.310762	8.929132	4.234618	0.605502582	low
GSM519767	1.97	1	7.64145	6.670213	7.318766	8.718212	4.651068	0.697666734	low
GSM519768	14.08	0	8.368009	5.241831	5.701663	3.9197	8.297133	1.337248828	high
GSM519771	12.8	0	9.228866	5.058927	6.403593	7.854931	3.722407	1.055535497	high
GSM519772	5.95	0	8.49398	6.574963	5.753675	12.000253	5.497461	0.869461895	low
GSM519773	4.58	1	8.529704	5.763302	6.342615	5.471321	4.341497	1.034734476	high
GSM519774	5.74	0	9.905234	4.718911	6.421842	7.087931	11.301581	1.405927491	high
GSM519776	6.12	0	10.265556	4.938541	6.415902	5.244131	4.15159	1.294533968	high
GSM519778	5.43	0	8.683122	5.940624	6.27282	5.750092	10.295663	1.186615545	high
GSM519779	5.33	1	8.688453	5.530158	6.263403	7.421638	4.347748	1.014002966	high
GSM519780	7.08	0	7.627305	6.438762	6.685418	7.753277	5.488896	0.816155811	low
GSM519781	13.96	0	8.714947	5.453288	5.339687	5.252325	8.153883	1.365001175	high
GSM519782	13.39	0	9.528944	5.065443	6.369839	5.861269	7.373344	1.266804272	high
GSM519784	7.42	1	9.112907	5.119883	6.47138	4.663955	7.881381	1.257633686	high
GSM519789	5.96	0	6.951234	8.363857	6.564595	7.276018	5.04827	0.674240386	low
GSM519794	8.74	0	9.270096	4.937398	5.981513	10.145131	4.191291	1.063288574	high
GSM519795	5.36	0	6.043886	7.102121	6.475868	8.23922	4.659066	0.661946064	low
GSM519797	0.87	1	9.219905	4.749307	6.597655	4.874122	9.446641	1.321762751	high
GSM519799	10.82	0	8.163304	5.813883	5.725818	8.236311	5.667856	1.020190141	high
GSM519800	1.21	0	9.456565	5.499884	5.868062	10.534218	7.621615	1.123996695	high
GSM519803	4.69	1	8.381423	5.254809	5.524229	5.010575	6.711162	1.275814471	high
GSM519805	5.58	0	7.366443	7.822469	6.052259	5.952328	10.133518	0.92259847	low
GSM519809	6.11	0	4.720011	7.593011	6.482442	11.066006	4.955475	0.513584798	low
GSM519810	6.1	0	6.602467	8.527737	6.557663	7.342422	4.034045	0.628300474	low
GSM602338	4.149041096	0	7.075028	7.447185	6.251933	6.362291	4.645272	0.781806166	low
GSM602340	2.755890411	1	11.737694	4.476978	5.609289	4.474477	10.09442	2.034044253	high
GSM602341	1.843561644	1	9.118566	4.027688	5.528711	5.037399	4.422342	1.427494009	high
GSM602342	9.863013699	0	7.933903	6.074182	6.313664	9.482746	6.523844	0.880691371	low
GSM602343	9.863013699	0	8.879955	4.771648	6.290721	7.951222	9.269759	1.198837414	high
GSM602344	2.915342466	1	8.873453	5.594226	5.436771	8.808616	5.226518	1.124222224	high
GSM602345	9.863013699	0	9.649256	6.081898	6.106118	8.052468	4.768733	1.077536526	high
GSM602348	6.770136986	0	5.692017	7.576385	6.485432	5.053345	4.553586	0.683222291	low
GSM602352	7.434246575	0	9.357971	4.5273	4.770817	4.909204	12.198564	1.874137904	high
GSM602353	9.863013699	0	8.294467	4.216757	5.758012	4.998705	5.007462	1.276512922	high
GSM602356	9.863013699	0	7.110513	7.580638	6.341021	4.897364	4.443802	0.801357298	low
GSM602359	3.244931507	1	9.09579	3.93845	5.60035	5.07574	4.59019	1.423423352	high
GSM602360	4.152328767	1	9.479545	5.497939	5.774687	6.712255	10.341152	1.378929438	high
GSM602361	9.863013699	0	8.117824	6.105082	5.876169	8.547818	4.333981	0.933568484	low
GSM602362	9.863013699	0	9.378344	4.859085	5.768956	5.647534	12.733748	1.570638038	high

GSM602363	5.792054795	1	6.74911	6.560504	6.418121	7.399525	8.928504	0.843956727	low
GSM602364	7.51890411	1	7.9846	6.951945	6.280906	8.533822	8.394277	0.894679961	low
GSM602365	9.736438356	0	7.16907	7.093311	5.654533	10.233394	4.68486	0.776142176	low
GSM602366	1.849315068	1	9.211587	5.462226	5.552562	4.486555	11.175471	1.525760982	high
GSM602370	3.578630137	1	10.367884	4.563092	4.736939	4.279608	7.385143	1.89476437	high
GSM602371	9.863013699	0	8.24484	7.329572	6.008248	9.084259	5.370628	0.848767735	low
GSM602374	9.820273973	0	9.876742	5.181563	5.511722	4.371589	10.587808	1.656213206	high
GSM602376	6.451232877	1	9.215092	4.693211	6.843626	6.025622	4.641748	1.105951015	high
GSM602378	9.863013699	0	8.557654	5.111817	7.397478	4.23051	12.429724	1.177770188	high
GSM602379	8.168219178	1	8.198751	5.521785	6.16986	5.126525	4.614027	1.06490374	high
GSM602380	4.726027397	1	9.600387	5.180189	5.716653	10.534579	4.166329	1.103242631	high
GSM602382	9.863013699	0	7.859998	7.554392	6.247163	7.179866	6.164455	0.843046769	low
GSM602383	9.817808219	0	8.004532	7.542717	6.642496	8.439303	5.720735	0.768318392	low
GSM602384	4.808219178	1	10.716777	4.512026	6.307268	4.812061	4.902002	1.464232294	high
GSM602385	6.287671233	0	9.029781	4.959347	7.155823	4.44885	4.553296	1.071140071	high
GSM602386	9.863013699	0	9.722514	5.369803	6.33353	5.707659	7.159226	1.267452897	high
GSM602387	9.863013699	0	8.050207	6.007488	6.262079	4.638955	4.931899	1.021720709	high
GSM602389	7.851780822	0	6.679636	6.819409	6.371819	8.505845	6.096567	0.747535759	low
GSM602391	7.764657534	0	7.305963	7.558182	6.750822	5.830611	6.232925	0.779598799	low
GSM602394	9.863013699	0	9.330793	5.001639	5.650806	4.632143	12.506999	1.617922394	high
GSM602398	8.751780822	0	9.275081	5.424684	6.280405	9.192758	5.074479	1.034419681	high
GSM602401	1.189315068	1	10.480701	4.432317	6.280034	4.162705	8.397824	1.599345191	high
GSM602404	7.522191781	0	6.21147	6.404946	6.411556	8.788645	8.606964	0.769467643	low
GSM602406	7.686575342	0	9.142592	4.789471	6.249602	4.501177	4.530486	1.243745415	high
GSM602408	9.863013699	0	8.401569	5.878911	6.784328	7.630039	5.324776	0.905915188	low
GSM602409	9.863013699	0	6.604564	5.570475	8.183587	7.20959	4.353202	0.635416205	low
GSM602411	8.749315068	0	8.941249	5.545097	5.484773	9.294766	5.109041	1.107190049	high
GSM602412	2.098356164	1	9.306143	4.321965	5.560648	4.347378	12.124134	1.724157933	high
GSM602414	6.859726027	0	8.736962	6.200728	6.338439	5.712421	8.722069	1.119021883	high
GSM602415	6.12	0	8.737592	4.087451	5.922792	5.136882	4.399436	1.290751022	high
GSM602417	6.119178082	0	7.261723	6.077673	6.67406	6.403632	7.25404	0.883454498	low
GSM602424	6.124931507	0	9.62947	4.276495	5.375266	4.26346	5.191826	1.569573899	high
GSM602426	5.956438356	0	8.663895	4.777977	6.802177	4.275929	5.176301	1.124321526	high
GSM602429	5.958082192	0	8.585953	6.141429	6.907447	8.889131	9.756229	0.941931133	low
GSM602430	6.042739726	0	7.284438	7.120889	6.478693	8.316457	7.637642	0.794786864	low
GSM602432	2.713972603	1	8.726611	3.986539	5.320612	5.172137	5.100688	1.43555887	high
GSM602438	7.005205479	0	8.754892	6.255503	6.995036	6.407648	4.501876	0.903424029	low
GSM602441	5.874246575	0	8.41242	4.862036	4.534896	4.168359	11.037112	1.720465323	high
GSM602443	1.765479452	1	8.287534	4.915589	6.864226	5.986556	4.666185	0.993064014	low
GSM602446	5.874246575	0	8.938776	4.152183	5.538158	5.016	4.924928	1.404622904	high
GSM602447	5.875890411	0	7.718635	6.215233	6.809643	9.375768	5.188316	0.774635916	low
GSM602451	5.787945205	0	6.132449	7.984996	6.812733	5.753342	5.094906	0.652388594	low
GSM602457	2.914520548	1	9.475325	4.335839	6.48372	4.750954	5.376486	1.301074177	high
GSM602460	5.464931507	0	9.993385	4.765835	5.487766	6.209358	11.383957	1.662374487	high
GSM602462	5.069589041	0	6.021849	7.853581	6.283969	9.377663	5.694029	0.631251853	low
GSM602464	5.236438356	0	6.656618	8.355463	7.003688	6.72263	4.749216	0.623642479	low
GSM602465	4.648767123	0	7.87943	5.156208	6.741935	9.815113	4.882195	0.843597626	low
GSM602466	5.594794521	0	7.203325	6.69927	6.732389	7.853114	8.261862	0.809709388	low
GSM602468	4.855068493	0	6.588336	6.038976	5.704085	10.091708	4.645687	0.793634407	low
GSM602469	4.563287671	0	8.799245	4.186796	5.560648	5.078151	4.337778	1.356710931	high
GSM602470	4.561643836	0	7.515817	7.22235	6.310986	9.208273	4.388655	0.744479772	low
GSM602471	4.566575342	0	6.466097	7.30495	6.931406	8.16919	4.749968	0.63934848	low
GSM602473	4.564931507	0	6.845923	7.017192	6.598405	10.851831	5.710218	0.664360176	low
GSM602477	4.566575342	1	8.880468	6.62446	7.027847	8.595112	11.152843	0.956778104	low
GSM602478	5.247945205	0	10.091888	4.82388	7.029594	7.892106	5.472584	1.111566613	high
GSM602481	4.543561644	0	6.424396	6.941743	6.231275	9.832926	10.706103	0.783001937	low
GSM602482	4.39890411	0	8.538769	6.563763	5.796835	4.378206	5.236369	1.112664781	high
GSM602485	4.399726027	0	6.597593	5.917055	6.021858	9.378594	9.781388	0.882347503	low

GSM602487	4.75890411	0	8.736305	4.016527	6.281436	4.588042	4.825588	1.268697601	high
GSM602490	4.314246575	0	9.419835	5.39706	6.481369	7.09289	5.250561	1.100157661	high
GSM602495	4.315890411	0	6.850715	6.841013	5.956337	6.692033	4.818996	0.830000092	low
GSM602499	3.408493151	1	8.104914	7.112238	6.786258	9.837672	5.689685	0.749702713	low
GSM602500	4.318356164	0	9.194994	6.035077	5.184033	4.947664	5.861626	1.339735708	high
GSM602501	4.148219178	0	8.316515	5.639949	5.553228	4.78636	11.487373	1.37641318	high
GSM602504	4.232876712	0	7.753193	5.757367	6.120849	10.310587	4.168664	0.840392316	low
GSM602505	5.310410959	0	8.294079	5.604325	7.663344	6.329672	7.36482	0.884978474	low
GSM602506	4.232054795	0	7.688966	7.107156	7.154814	4.367544	11.890685	0.944693082	low
GSM602510	4.149041096	0	10.194628	4.66814	6.016186	5.202054	5.540731	1.435444172	high
GSM602511	4.153150685	0	7.20165	6.391102	6.736849	6.829278	6.402838	0.821796852	low
GSM602512	4.149041096	0	6.786709	6.854595	6.199832	8.276526	4.920261	0.757113004	low
GSM602513	4.150684932	0	7.02472	5.978073	6.126608	6.926669	4.893139	0.875468382	low
GSM602518	2.507671233	1	5.845538	7.232363	6.064649	9.104898	5.738846	0.6783588	low
GSM1045209	2.068493151	1	10.004207	7.013283	6.307042	7.851616	4.661008	1.012967378	high
GSM1045210	8.257534247	0	8.946028	6.009695	6.594506	7.725727	5.191185	0.964746925	low
GSM1045211	4.728767123	1	9.2062	5.374316	6.651331	5.791161	7.455923	1.15727789	high
GSM1045215	6.709589041	0	8.76065	5.874263	5.643563	6.599644	4.545659	1.120121971	high
GSM1045217	1.150684932	1	8.221204	5.431042	7.696195	4.608159	4.546661	0.880510849	low
GSM1045218	3.04109589	1	9.352515	4.645657	5.939573	4.625458	6.544847	1.397653235	high
GSM1045219	1.336986301	1	8.777182	5.355528	6.193527	6.015694	7.318574	1.173578869	high
GSM1045224	5.101369863	1	9.441147	6.172396	5.600045	4.937748	4.703004	1.247319368	high
GSM1045225	3.350684932	1	8.65688	4.327924	6.56263	7.566024	8.259251	1.156803347	high
GSM1045228	2.594520548	0	7.763116	5.597377	6.492917	9.530705	11.800905	0.98647688	low
GSM1045231	6.679452055	0	8.310045	5.983844	5.723653	5.131537	10.482799	1.263615992	high
GSM1045238	0.378082192	1	8.234424	6.460653	6.100619	5.657334	4.872491	0.991809292	low
GSM1045244	7.073972603	0	7.343053	5.360331	5.759229	4.884559	4.165293	1.049738689	high
GSM1045248	8.076712329	0	9.843943	4.549129	4.644167	4.761682	4.596981	1.687640567	high
GSM1045250	0.531506849	1	9.910749	3.944197	6.226988	5.663534	10.839737	1.592643798	high
GSM1045252	7.942465753	0	7.112946	4.965631	5.974015	11.939547	4.682167	0.821781888	low
GSM1045253	7.750684932	0	7.946562	6.144923	5.852237	4.743815	4.633789	1.049540109	high
GSM1045254	7.630136986	0	9.554698	6.971854	6.395301	5.342473	7.844532	1.123096648	high
GSM1045259	6.057534247	0	8.390053	6.003119	6.298909	7.41279	5.037606	0.960373109	low
GSM1045261	1.068493151	1	9.721682	5.38545	6.872987	6.473983	5.233283	1.094695855	high
GSM1045264	2.512328767	0	8.827705	5.326328	5.988614	4.87684	10.980291	1.373185155	high
GSM1045265	7.556164384	0	7.232186	6.66228	5.18374	10.013284	12.552499	1.039134516	high
GSM1045267	5.471232877	0	5.904056	7.240466	6.454025	10.131503	4.805914	0.610636132	low
GSM1045269	4.912328767	0	8.293848	6.541209	5.707569	8.687567	9.92509	1.062579362	high
GSM1045270	1.493150685	1	10.105177	5.069411	5.692664	5.566583	9.420052	1.558087615	high
GSM1045271	3.860273973	1	8.67533	6.365738	5.567988	5.67562	5.800314	1.146978488	high
GSM1045272	8.063013699	0	7.98294	6.423451	5.829425	5.544686	4.808424	1.010953458	high
GSM1045273	2.860273973	1	8.752972	5.422317	6.003997	6.123298	4.898837	1.128285719	high
GSM1045277	5.342465753	0	9.189941	5.428805	6.205453	5.482587	4.505502	1.157568772	high
GSM1045278	2.95890411	1	7.994728	5.152002	6.158037	11.113956	5.486384	0.899564522	low
GSM1045279	6.208219178	0	6.562861	7.035324	6.609083	11.436532	4.303248	0.612291922	low
GSM1045281	6.306849315	0	8.946028	5.382736	8.283288	5.29044	9.666047	0.958207023	low
GSM1045283	6.057534247	0	8.544751	6.87274	5.935569	5.816245	8.813947	1.102300104	high
GSM1045284	6.421917808	0	9.689807	4.59834	6.352192	7.749342	4.666413	1.180900647	high
GSM1045286	6.805479452	0	8.221204	5.430642	6.456793	10.161152	11.081877	1.011283327	high
GSM1045288	8.095890411	0	5.542767	8.849991	6.231864	5.398048	4.364734	0.622668015	low
GSM1045290	6.531506849	0	7.054518	5.31784	6.231864	11.931704	6.821493	0.805276015	low
GSM1045292	7.235616438	0	7.62134	6.029805	6.37621	8.038359	10.954806	0.986247033	low
GSM1045293	1.15890411	0	9.406264	4.315921	6.762981	4.599324	4.678962	1.231116018	high
GSM1045295	5.621917808	0	6.603164	7.760761	6.987577	11.404469	4.50963	0.554182686	low
GSM1045299	7.057534247	0	6.992753	6.535926	6.715016	6.222932	8.744384	0.859724386	low
GSM1045302	5.539726027	1	9.113074	5.859177	7.843148	4.903708	7.716696	0.96771268	low
GSM1045303	7.432876712	0	7.398235	5.783682	6.057875	8.321005	11.753376	1.037945175	high
GSM1045304	7.643835616	0	7.674721	6.392983	6.550795	6.33727	4.575634	0.860930772	low

GSM1045305	7.183561644	0	10.075418	5.694446	6.063025	4.794073	4.720646	1.295677938	high
GSM1045308	8.189041096	0	6.422434	6.193285	6.954446	7.936645	4.778055	0.697315154	low
GSM1045311	8.115068493	0	7.343984	5.14302	6.762252	9.055745	8.526335	0.890927945	low
GSM1176873	5.5	0	7.456663	6.60212	6.060849	4.679115	4.973332	0.947693608	low
GSM1176875	4.08	0	6.465799	6.487179	6.071728	6.200405	5.227	0.829652919	low
GSM1176876	5.67	0	5.229303	6.460505	6.209487	11.107947	6.361501	0.630815815	low
GSM1176880	2.08	0	9.484637	6.249388	5.817761	7.944615	7.040133	1.152218924	high
GSM1176881	3.58	0	8.807531	6.681703	5.911651	6.47602	9.353904	1.140188417	high
GSM1176882	3.08	0	8.741434	5.636978	5.990624	8.786182	11.33167	1.176466854	high
GSM1176886	2.17	0	7.485085	7.070471	6.210427	5.453559	5.593994	0.887023968	low
GSM1176889	1.67	1	9.333677	4.564155	6.113477	5.638624	9.266375	1.409066142	high
GSM1176891	2.67	0	8.04694	6.149934	6.355209	5.488192	5.6186	0.984534639	low
GSM1176892	5.58	0	8.881303	4.967502	5.836355	6.578347	5.083425	1.198277435	high
GSM1176894	5.67	0	6.71229	7.006108	6.14662	7.376543	5.170417	0.77568149	low
GSM1176895	5	0	5.657439	7.084434	6.322959	7.790886	8.235286	0.718383089	low
GSM1176898	3.42	1	6.475788	6.618315	6.311544	9.557227	8.91052	0.772886359	low
GSM1176899	1.33	1	9.69795	2.664962	4.642618	3.698914	10.577995	2.28478571	high
GSM1176900	1.75	1	6.161865	6.831114	5.905613	11.519497	8.526827	0.725336047	low
GSM1176901	5	0	8.670151	5.109758	6.309157	7.46396	4.902524	1.050608328	high
GSM1176905	4.92	0	8.349186	5.289594	6.942218	4.453378	6.435641	1.051588019	high
GSM1176907	4.67	0	10.197005	5.793091	6.053125	4.836219	12.042238	1.536500294	high
GSM1176911	5.75	0	7.445933	7.154379	6.205969	9.643821	4.719033	0.749206931	low
GSM1176912	4.5	0	9.574891	3.634761	5.979734	6.593383	10.277353	1.565246061	high
GSM1176915	3.83	0	9.508022	6.422365	5.82414	4.924677	5.947103	1.227859782	high
GSM1176917	4.58	0	8.974124	6.078401	6.688225	5.706239	4.127042	0.991323359	low
GSM1176918	4.58	0	7.23807	7.054667	6.215075	9.354239	3.430896	0.724694226	low
GSM1176919	4.58	0	9.806011	5.073235	6.612772	7.193192	8.00343	1.219468046	high
GSM1176921	4.25	0	8.594769	6.429128	6.465641	5.408751	4.081991	0.968350355	low
GSM1176922	4.25	0	8.955866	4.337885	8.190515	8.080285	3.958789	0.843302671	low
GSM1176925	4.5	0	6.695151	7.158969	7.413263	7.422514	4.005318	0.622560725	low
GSM1176927	3.83	0	9.095624	5.491901	7.09263	6.937055	4.157605	0.95227719	low
GSM1176930	4.17	0	9.428161	5.226099	5.946953	4.802613	10.133567	1.45044575	high
GSM1176931	2.17	1	8.751807	7.118603	5.530939	9.428824	3.509741	0.918242471	low
GSM1176933	4.67	0	7.623803	4.728288	6.552218	5.884029	9.55244	1.107279319	high
GSM1176936	4.5	0	7.480591	8.470819	7.076312	6.710593	11.400027	0.770793999	low
GSM1176939	4.33	0	10.047323	4.955039	5.121476	6.675648	4.768498	1.469021978	high
GSM1176940	4.25	0	9.025385	4.56738	6.169957	7.655052	4.419603	1.136326569	high
GSM1176945	4.08	0	9.323597	5.072282	7.106468	6.153757	8.464652	1.135950444	high
GSM1176949	4.33	0	7.144246	7.100484	7.346968	9.200531	6.006678	0.65024819	low
GSM1176950	2.42	1	6.771	5.922006	6.715634	7.313444	4.109639	0.765845779	low
GSM1176953	4	0	8.97058	4.432971	5.126663	6.965473	10.815217	1.565392187	high
MB-0362	3.865753425	1	8.710001	6.532034	5.261377	7.891562	9.964663	1.21138247	high
MB-0386	11.35342465	0	8.418493	8.116345	5.808238	11.455956	4.918895	0.764174148	low
MB-0574	9.846575342	0	8.049695	7.050752	5.337835	7.169254	4.831836	0.984750846	low
MB-0641	8.430136989	0	6.069653	6.799168	5.220423	10.296799	9.388792	0.843013943	low
MB-0218	10.77260274	0	8.943273	4.861396	5.696076	6.712733	5.821437	1.254980505	high
MB-0891	12.27945205	0	6.432788	6.851673	6.264314	10.902683	5.191175	0.668711028	low
MB-0658	7.994520548	0	8.745429	5.414847	6.486281	8.524348	8.924634	1.066353425	high
MB-0899	14.44931507	0	8.027845	7.21288	6.499606	9.179294	7.298667	0.815098687	low
MB-0506	3.764383562	1	8.715977	5.424935	5.900766	4.521372	7.461967	1.27505376	high
MB-0223	6.684931507	1	7.558396	5.576143	5.862679	7.15563	4.272124	0.965572547	low
MB-0445	10.89315068	0	7.809374	7.61722	6.017477	4.583251	4.746174	0.910291161	low
MB-0199	11.86849315	0	7.450364	7.371809	6.150008	5.166735	6.684416	0.901513542	low
MB-0517	5.087671233	0	7.364529	6.730521	6.545741	4.385148	5.191468	0.881524822	low
MB-0428	4.597260274	0	8.238746	5.985607	6.210705	6.808866	9.716083	1.088882645	high
MB-0660	1.556164383	1	9.116465	5.902492	6.408005	10.853049	9.894874	1.018150818	high
MB-0143	4.465753425	1	8.031816	7.195167	6.213134	5.43612	7.041738	0.957241435	low
MB-0513	9.619178079	0	7.856911	6.866965	6.176049	5.280441	4.579103	0.922403855	low

MB-0541	14.32328767	0	8.586789	5.43055	6.261029	5.316761	5.083042	1.103886597	high
MB-0455	8.164383562	0	8.752616	5.706643	6.705574	5.641031	4.633755	1.009931516	high
MB-0540	15.98904109	0	6.510591	6.8476	6.370705	6.226147	4.093111	0.756532069	low
MB-0157	9.432876715	0	8.692942	5.78755	5.470301	4.674116	7.728364	1.315703433	high
MB-0443	3.701369863	0	7.590095	8.620719	6.148004	9.371154	4.771845	0.69095838	low
MB-0584	9.715068493	0	8.676277	9.465465	6.461704	6.682829	6.1011	0.7750866	low
MB-0322	4.153424658	1	6.525265	7.23	5.702557	4.697274	9.709508	0.96604834	low
MB-0501	5.876712329	0	6.430938	8.945126	6.274974	4.47542	4.769516	0.696532065	low
MB-0401	2.15890411	1	9.414418	5.170753	5.399731	8.708452	4.800403	1.22241928	high
MB-0606	2.257534247	1	7.630249	7.348996	6.359436	7.129455	4.22977	0.790472799	low
MB-0666	2	1	8.490339	5.876119	6.482453	5.191727	4.99484	1.026422339	high
MB-0138	12.37534247	0	7.221417	7.745815	6.137479	7.223177	4.343323	0.759976813	low
MB-0471	8.832876715	0	8.553076	5.773927	6.357783	4.648543	5.170959	1.082908884	high
MB-0619	7.745205479	0	8.443369	6.681385	6.20827	8.309687	5.208838	0.904066491	low
MB-0171	0.446575342	0	6.54076	8.137125	5.938181	10.394872	5.977069	0.66325234	low
MB-0621	2.75890411	0	5.673641	8.190657	5.893525	9.231067	9.82183	0.693512337	low
MB-0372	5.15890411	1	9.592184	6.860313	6.78122	6.595234	10.557681	1.098503301	high
MB-0374	0.117808219	0	9.008806	6.119982	5.461666	4.774099	5.464961	1.253083726	high
MB-0382	11.21643836	0	5.558825	6.527909	5.925549	6.681865	4.770197	0.753408265	low
MB-0066	12.93972602	0	8.806891	6.478935	6.489316	8.413537	5.152043	0.909830586	low
MB-0229	5.876712329	0	7.565189	6.578019	6.61383	4.034589	5.683779	0.921529914	low
MB-0505	4.989041096	0	5.947223	8.665437	6.522811	7.610828	4.555248	0.588176587	low
MB-0102	11.56986302	1	8.698976	5.106459	6.732879	4.78747	4.970341	1.086823545	high
MB-0569	4.890410959	1	9.941975	7.30585	5.942409	7.043796	4.725653	1.066074354	high
MB-0585	9.509589041	1	7.539556	7.282503	6.547441	4.386728	4.395889	0.843466957	low
MB-0463	9.909589044	1	6.905736	8.460833	6.618633	5.705373	5.199684	0.699081839	low
MB-0203	4.830136986	1	8.005828	6.399804	6.361532	10.405156	6.027392	0.823686867	low
MB-0371	10.77808219	0	8.884648	6.020026	5.972285	10.650891	4.815302	0.941040962	low
MB-0380	5.698630137	1	9.965929	4.635109	6.048306	4.871596	5.1793	1.405263272	high
MB-0221	1.660273973	1	8.534218	5.302938	5.462999	5.903748	5.585085	1.231133009	high
MB-0659	1.917808219	1	6.888585	6.340636	6.999021	4.602923	8.089551	0.863191451	low
MB-0379	11.15068493	0	7.478958	7.685275	5.342839	9.602305	4.822932	0.817722486	low
MB-0624	5.263013699	0	5.269699	8.278463	5.743881	10.150821	4.485211	0.581296709	low
MB-0654	5.704109589	0	7.090222	8.083106	6.476519	5.955509	7.214046	0.77604566	low
MB-0467	8.679452055	1	8.702948	5.712577	6.341608	4.55926	12.47018	1.309907805	high
MB-0349	12.00273972	1	8.246146	6.309487	6.534897	6.282932	10.575168	1.053574529	high
MB-0378	7.997260274	1	8.953807	5.41936	5.14437	4.866075	11.42446	1.570648251	high
MB-0352	4.536986301	1	7.413489	4.723618	5.573078	9.366552	5.859232	1.020392194	high
MB-0601	2.438356165	1	10.143631	4.158741	5.715994	4.191155	10.308401	1.786311388	high
MB-0328	10.32328767	1	9.885922	5.081977	5.861923	9.513646	9.429444	1.305333169	high
MB-0325	14.59178082	0	10.196807	4.175417	5.462001	4.057292	4.147045	1.622898364	high
MB-0358	2.701369863	0	9.464321	5.336027	5.63799	8.641981	10.436637	1.335894525	high
MB-0413	11.51232877	0	8.189888	6.419655	6.266364	6.250005	4.621717	0.94354759	low
MB-0195	12	0	9.133952	4.643159	5.815629	5.056185	5.729324	1.347858764	high
MB-0317	12.46575343	1	7.147152	9.313705	5.898858	6.447501	5.620942	0.730299983	low
MB-0139	8.975342466	0	5.527929	7.580619	5.593678	10.182408	5.216886	0.652494731	low
MB-0480	7.77260274	0	6.303948	5.648452	5.659944	5.72481	4.545633	0.923832874	low
MB-0368	5.087671233	1	8.742343	4.330173	6.233409	5.267492	4.920935	1.222236728	high
MB-0389	7.969863014	0	8.694959	4.692819	6.487578	5.188267	5.36787	1.156138529	high
MB-0248	5.835616438	0	6.691037	6.968697	6.708657	4.798588	8.113749	0.835491207	low
MB-0904	11.89315068	1	7.767532	6.936388	6.12454	8.343233	5.487011	0.844747531	low
MB-0204	1.997260274	1	6.668289	7.922439	5.682832	8.768551	5.06329	0.731667325	low
MB-0184	8.961643833	0	7.34809	8.064967	6.411354	4.638562	4.546579	0.790635046	low
MB-0400	1.846575343	1	9.622085	5.968281	5.791147	7.460923	6.497223	1.202201774	high
MB-0511	5.071232877	0	7.643321	6.591879	6.302464	11.492054	5.497548	0.75305251	low
MB-0500	5.545205479	0	9.903702	4.916483	5.70618	4.278094	11.145634	1.675315105	high
MB-0150	9.139726027	0	9.132566	6.449023	6.307359	4.723749	5.093999	1.090060499	high
MB-0895	3.542465753	1	9.598095	5.03605	6.431598	5.722066	4.898671	1.203375196	high

MB-0366	5.279452055	0	7.76241	6.350648	6.518059	7.348685	10.109454	0.959553343	low
MB-0545	3.383561644	1	9.096545	6.365514	5.623148	10.39692	5.440091	1.004814821	high
MB-0642	6.920547945	1	6.865015	7.517754	6.647642	4.13979	9.330332	0.863101267	low
MB-0181	7.065753425	0	7.476431	7.381397	6.388697	10.457247	5.099277	0.706461545	low
MB-0295	13.52054795	0	6.801304	7.851323	6.060025	7.873203	5.465289	0.73466793	low
MB-0618	8.375342466	0	7.372282	7.556819	5.797406	6.760141	3.989432	0.82691157	low
MB-0496	10.75890411	0	6.612213	7.611495	5.782254	11.08579	9.36318	0.750383436	low
MB-0285	0.276712329	0	6.081188	5.349715	6.125717	12.388651	5.270824	0.705840455	low
MB-0360	10.89589041	1	8.441689	8.971968	6.04597	4.584275	4.45193	0.862083223	low
MB-0344	12.56986301	0	5.06597	7.131419	5.700756	9.61675	5.168396	0.647800004	low
MB-0583	5.147945205	0	5.992491	8.277383	6.222457	6.472816	4.590056	0.66004952	low
MB-0485	3.021917808	1	8.443369	7.209945	5.652076	8.987346	7.258743	0.961479241	low
MB-0609	8.112328767	1	8.449277	5.292835	5.868324	5.065507	6.192537	1.203344304	high
MB-0197	5.81369863	0	8.975073	6.419216	5.63799	4.272568	5.156785	1.202107039	high
MB-0410	5.178082192	0	7.17812	7.667282	5.99048	8.560514	10.161413	0.848631831	low
MB-0528	15.94246576	0	6.531302	7.433804	6.241517	4.475093	5.399409	0.80558901	low
MB-0165	3.915068493	1	8.620514	6.23039	5.556196	5.530594	8.818467	1.242802762	high
MB-0152	5.180821918	0	8.777392	5.071272	5.706918	7.82085	4.54873	1.136114793	high
MB-0594	3.635616438	0	7.234388	4.894662	6.745451	5.995524	4.969898	0.919837517	low
MB-0521	17.44109589	0	6.334986	7.533029	6.454907	7.746754	7.266571	0.71233618	low
MB-0536	12.11232877	1	9.782927	5.207066	4.951415	9.340318	6.384458	1.365744633	high
MB-0319	3.345205479	1	8.745429	7.872982	5.625984	5.149799	4.678588	1.011350657	high
MB-0580	6.523287671	1	8.939714	5.476759	5.634115	4.55591	4.957384	1.271325719	high
MB-0320	5.238356164	0	6.018878	7.201114	6.418065	10.240778	4.702372	0.618744139	low
MB-0207	14.27123287	0	7.11551	5.629776	6.621267	8.303864	5.050973	0.811206641	low
MB-0167	3.542465753	1	8.959708	4.552807	6.907138	7.195632	4.775166	1.043001187	high
MB-0591	9.819178085	1	7.885981	6.759145	6.555239	4.670539	4.386128	0.898380962	low
MB-0599	10.13150685	0	6.225985	6.934308	6.946547	5.342391	5.897727	0.723340631	low
MB-0575	10.56712329	0	7.813436	5.396553	6.736896	5.610848	4.49255	0.938621978	low
MB-0214	12.0739726	0	8.369762	6.97243	5.846184	5.35486	10.720117	1.154971669	high
MB-0524	6.643835616	0	6.956643	7.849687	6.261455	5.616217	4.163348	0.75870825	low
MB-0880	0.101369863	0	6.088997	8.601521	6.946547	4.765386	4.680546	0.622294467	low
MB-0664	0.909589041	1	10.162031	5.202458	6.617761	4.75163	12.822239	1.510974933	high
MB-0446	5.939726027	0	8.319995	6.115484	5.85065	6.17787	9.963153	1.173832356	high
MB-0008	3.4	1	8.923404	5.113899	6.454907	10.376796	5.727001	0.975122353	low
MB-0154	9.432876715	0	5.078182	7.238357	6.743183	8.859488	5.004171	0.567420009	low
MB-0550	10.87671233	1	9.097911	5.210069	6.701097	7.972728	11.836277	1.183288993	high
MB-0616	2.38630137	1	8.630073	6.428067	6.132104	4.330624	4.778926	1.072509231	high
MB-0122	11.41643836	0	5.570216	7.304017	6.82049	5.089118	6.503577	0.686795425	low
MB-0425	2.005479452	1	7.162814	7.586335	6.867546	7.939787	7.06376	0.717016203	low
MB-0314	0.364383562	1	8.6821	4.772223	5.447133	9.714205	10.144413	1.2741977	high
MB-0356	17.54794521	0	7.220016	6.334078	6.673641	9.085288	11.561514	0.870246627	low
MB-0440	8.28767123	0	9.939261	5.193268	7.432356	8.328642	5.157624	0.984232682	low
MB-0398	3.550684932	1	8.741329	5.228666	6.115453	4.814172	5.186231	1.184001297	high
MB-0449	9.252054797	0	6.689039	7.167165	7.388195	6.792207	10.397455	0.73683631	low
MB-0628	8.709589044	0	8.824019	7.523981	7.089648	3.957628	5.437011	0.90168761	low
MB-0341	14.29315068	1	7.750392	5.768303	6.099213	9.383852	10.540579	1.003667035	high
MB-0373	2.219178082	1	9.216136	5.098502	6.168012	5.06211	11.668001	1.428150946	high
MB-0225	17.44109589	0	7.874489	4.705017	5.35415	5.820249	5.004171	1.215818387	high
MB-0424	0.808219178	1	5.445583	5.928833	6.025663	11.214437	6.048613	0.681422929	low
MB-0209	6.769863014	0	6.036828	5.407915	6.784356	12.396809	5.112704	0.635217352	low
MB-0115	5.484931507	1	9.679852	4.666349	6.714037	4.927149	8.356547	1.332151539	high
MB-0136	7.249315068	0	7.313297	7.166024	6.787951	7.491387	5.301485	0.741114084	low
MB-0542	3.994520548	1	8.903826	5.797402	6.154411	8.649441	6.644164	1.041578544	high
MB-0151	4.046575342	1	8.007773	5.879615	5.839119	8.116238	4.819894	0.969162593	low
MB-0590	5.956164384	1	9.406032	4.159096	5.813796	4.144768	10.262593	1.641689678	high
MB-0657	2.501369863	0	6.832608	6.845545	6.151204	10.665308	5.107566	0.710739692	low
MB-0395	2.383561644	1	8.222579	6.291562	6.27988	5.044118	10.642639	1.13877747	high

MB-0294	5.991780822	0	6.38418	5.932741	5.937806	8.222539	10.64958	0.92593995	low
MB-0439	9.216438353	0	9.019979	6.655038	6.898309	4.516598	4.621798	0.972750685	low
MB-0529	3.989041096	1	8.107136	6.571994	6.657327	7.096092	6.020746	0.878731115	low
MB-0224	14.52876713	0	7.775525	8.386627	6.468068	9.113519	5.123279	0.696110048	low
MB-0126	10.49041096	0	7.194443	8.924048	6.651584	4.917557	5.358314	0.711223016	low
MB-0220	3.273972603	1	7.503419	6.000997	5.660662	11.623759	4.758683	0.833414564	low
MB-0192	11.88767123	0	7.798162	7.020478	5.793357	8.98073	5.272365	0.859261302	low
MB-0239	7.032876712	1	6.22915	5.783408	6.149219	8.119634	4.175529	0.775818963	low
MB-0238	15.87671233	0	9.154247	5.057425	6.314802	6.48759	4.918692	1.141259001	high
MB-0194	7.528767123	0	6.035465	6.634771	6.18933	8.338046	5.425423	0.724127161	low
MB-0882	3.482191781	1	8.826194	6.778739	6.145239	5.525685	5.541442	1.038273961	high
MB-0010	0.64109589	1	9.622085	6.470381	6.222858	5.030544	10.979516	1.306340474	high
MB-0236	16.85205479	0	8.28227	6.028758	7.014274	7.007756	5.285448	0.874169193	low
MB-0377	11.04383562	0	6.178205	6.904689	6.489316	9.426115	5.436665	0.664937373	low
MB-0321	14.35342465	0	7.85868	5.299565	5.972285	4.646782	5.267408	1.111702554	high
MB-0101	12.16712328	0	7.494713	7.592029	5.937048	7.415624	10.603909	0.930406276	low
MB-0662	5.736986301	1	8.166302	6.158336	5.747544	4.506019	10.81455	1.260631122	high
MB-0166	8.580821918	0	7.251117	6.433434	5.832751	9.291062	4.668601	0.828056722	low
MB-0180	5.123287671	0	7.508024	6.5033	6.857021	4.130049	5.261876	0.879430044	low
MB-0525	5.41369863	0	9.321916	4.703209	5.44747	4.603572	10.603909	1.63180762	high
MB-0307	14.57534246	0	7.222804	5.45889	7.178586	8.756378	4.584677	0.748382064	low
MB-0002	6.956164384	0	7.493677	5.516308	6.93295	6.16386	5.184193	0.874836724	low
MB-0466	3.605479452	0	9.088731	6.066006	7.165166	6.768402	5.168396	0.92723145	low
MB-0120	2.389041096	1	8.8112	6.074109	7.695855	5.313007	4.690729	0.869223683	low
MB-0869	9.238356164	1	9.443489	4.779537	6.027597	4.333967	8.818895	1.465495984	high
MB-4627	15.33972602	1	7.871416	5.914843	6.576014	6.251783	9.630488	1.020524488	high
MB-4004	21.11232877	1	7.073424	4.845665	6.283175	5.450809	3.782838	0.961719185	low
MB-4708	18.73150685	1	6.957066	7.595625	6.04597	8.811372	7.038227	0.765642145	low
MB-4622	23.24657534	1	8.752616	5.342536	5.985074	6.157733	11.726512	1.327769903	high
MB-4688	7.010958904	1	9.575003	4.58565	6.686502	4.870938	4.256506	1.215916012	high
MB-4691	23.22465754	1	7.791168	7.220559	6.615144	5.932864	6.29151	0.852940732	low
MB-4723	17.42191781	0	9.067994	5.286454	6.103156	7.723833	5.187968	1.106217851	high
MB-4729	2.301369863	1	8.638658	4.659337	6.951694	4.997803	9.752092	1.200601521	high
MB-4724	12.00547945	0	8.844523	4.361262	5.666676	7.34649	5.967565	1.274582446	high
MB-4770	12.49863014	1	9.431406	4.682811	5.122207	7.026848	7.826823	1.497730393	high
MB-4762	20.72054795	0	7.343715	6.735378	5.733299	5.470895	5.016853	0.947330532	low
MB-4796	10.54246576	0	9.139409	4.508076	6.328815	6.816214	11.254144	1.355541867	high
MB-4790	5.791780822	1	8.293703	5.836497	6.270462	7.600972	4.999034	0.960808614	low
MB-4797	8.167123288	1	8.27545	7.269736	6.56522	10.136744	5.295156	0.762179478	low
MB-5541	16.44931507	0	6.541145	7.096872	6.279052	8.285734	5.882057	0.733266	low
MB-5549	7.309589041	1	9.856573	4.441489	5.819725	5.366305	10.428833	1.615551688	high
MB-5519	13.90958904	1	8.308376	5.086078	6.151204	8.351326	5.252041	1.017144287	high
MB-5495	18.80547945	0	8.167005	7.21288	6.166011	7.377887	5.053996	0.873597116	low
MB-4832	11.64931507	1	6.934053	7.086736	6.264314	6.664638	4.767197	0.785899379	low
MB-4698	14.02739726	1	7.157446	6.694312	5.911328	9.95235	4.751912	0.779560578	low
MB-4715	2.895890411	1	9.188929	5.421101	6.084707	5.043001	11.53699	1.402464754	high
MB-4655	8.136986301	1	8.562951	4.267699	7.242425	6.030091	12.375858	1.209386765	high
MB-4661	19.25205479	0	7.711314	6.253874	7.239344	8.123706	5.122185	0.756197877	low
MB-4674	6.663013699	1	6.881528	7.181176	7.292635	4.957026	4.864182	0.712368997	low
MB-5152	9.528767121	1	7.903058	8.522051	6.498309	4.281607	4.588038	0.805613643	low
MB-5212	14.72054795	1	8.005828	5.66791	6.35197	5.112151	12.790869	1.213645257	high
MB-5183	9.742465751	1	7.571512	5.509906	6.762607	8.866623	12.811353	0.981885188	low
MB-4994	14.31232876	1	8.10578	6.715009	5.746818	7.000105	6.778111	1.008247778	high
MB-5004	17.81369863	0	9.019979	4.478732	6.947015	4.056215	4.839514	1.166351604	high
MB-5328	1.167123288	1	9.41272	4.83797	6.502615	7.624816	5.333483	1.12654744	high
MB-5318	1.164383562	1	8.839113	5.103265	5.986223	10.119462	4.890026	1.02333232	high
MB-4701	10.48219178	0	6.538091	5.917564	6.62348	7.769173	6.961114	0.797506111	low
MB-4709	12.0630137	0	7.456379	6.450338	6.320583	5.373332	4.657248	0.896833693	low

MB-4706	4.575342466	1	6.770155	8.593309	6.207456	7.132556	4.433987	0.678255921	low
MB-4719	6.704109589	1	7.207655	7.362892	6.465939	5.373692	4.777252	0.801619215	low
MB-4908	3.936986301	1	9.057779	4.439041	6.803962	7.732928	11.897964	1.244672815	high
MB-4871	3.271232877	1	9.355118	4.717306	6.044812	7.16241	12.898479	1.454555675	high
MB-4906	19.22191781	1	8.662684	5.123175	6.533151	5.154988	6.979842	1.150327981	high
MB-4911	2.583561644	1	10.936349	4.361262	6.580756	4.750426	8.277049	1.575175451	high
MB-4866	18.43561644	0	7.828204	5.212371	6.650247	7.480602	3.884382	0.89450715	low
MB-4858	10.63013698	1	8.241689	5.525916	5.626689	7.266138	5.848471	1.105307268	high
MB-4872	12.91232877	0	7.943366	7.740355	5.903398	10.15952	5.585441	0.785938714	low
MB-4887	2.909589041	1	8.829406	5.840735	5.715254	6.417755	9.985872	1.274087439	high
MB-4867	18.23835616	0	7.132404	6.983729	6.562197	5.973952	5.195866	0.800036777	low
MB-4888	18.94520548	0	9.417788	5.908671	6.620832	7.945598	11.598634	1.164144746	high
MB-4929	3.287671233	1	8.081307	4.48968	6.556119	8.182835	12.33851	1.16272695	high
MB-4945	1.65479452	1	10.786184	5.406523	6.532287	6.057459	13.078619	1.539747203	high
MB-4930	4.802739726	0	10.225572	4.609265	6.316045	5.081341	6.234372	1.414166867	high
MB-4670	5.57260274	1	7.335573	7.160199	7.23182	4.722123	5.580318	0.770200011	low
MB-5013	20.64657534	1	7.08047	6.287728	6.808512	5.361784	4.937465	0.823200655	low
MB-4977	17.81369863	1	7.963553	7.466303	6.467211	9.091309	10.603909	0.862755748	low
MB-4967	10.74246575	1	6.676894	5.594924	6.51206	9.542235	4.549448	0.751241674	low
MB-4981	14.85753425	1	5.146934	7.029785	6.187688	8.46901	9.090989	0.697811519	low
MB-4003	8.758904112	1	10.353648	4.007933	6.746375	4.502465	5.095047	1.402252087	high
MB-5072	4.128767123	1	7.722047	6.343269	5.02779	5.580198	11.250623	1.284117047	high
MB-5045	13.83287671	1	8.249157	4.864609	5.817507	4.149788	10.387609	1.393510917	high
MB-5116	5.898630137	1	10.328029	5.709969	6.02917	4.03782	4.202345	1.350308843	high
MB-5120	2.284931507	1	9.217537	4.347934	5.829018	4.80288	6.943019	1.438354158	high
MB-4764	20.17808219	0	7.708492	6.466823	5.770523	9.865663	7.102441	0.902862143	low
MB-4735	24.47671233	0	7.266916	5.905583	6.062367	4.715785	4.742549	0.975414286	low
MB-5033	4.334246575	1	8.29525	4.634621	6.194155	5.103531	4.611033	1.147968517	high
MB-4741	4.643835616	1	8.015499	6.339748	6.034263	7.02839	4.416119	0.935558502	low
MB-4732	12.31780822	0	9.681953	5.526279	6.221647	6.957463	4.75961	1.151058114	high
MB-5305	12.28219178	1	7.300585	6.03154	6.24961	4.463551	9.044139	1.04957331	high
MB-5236	13.69863014	0	9.885922	4.52681	6.381145	4.77871	11.892844	1.567740317	high
MB-5244	17.82465754	1	8.559414	6.191876	6.137672	4.904123	5.14754	1.072457939	high
MB-4959	9.663013701	1	6.68786	5.924562	6.608627	9.065314	8.37311	0.801485199	low
MB-4616	7.408219178	1	5.798255	5.18408	7.031061	6.468232	6.458792	0.766044192	low
MB-4644	7.397260274	1	9.720266	4.441489	6.532287	6.359019	6.166518	1.266532843	high
MB-4233	2.989041096	1	10.642399	4.635833	5.603855	4.936914	6.13376	1.628282668	high
MB-4935	15.96164384	0	10.904556	4.364584	6.697145	4.363972	10.538335	1.646884315	high
MB-4900	18.46027397	0	8.670411	6.700503	6.182866	8.117592	10.007344	1.039325154	high
MB-4941	4.819178082	1	7.306647	5.154172	6.4583	8.752072	5.266472	0.868390738	low
MB-5221	10.79178082	1	6.866658	6.217776	7.2338	6.482512	4.809551	0.733552377	low
MB-5139	7.394520548	1	8.513853	5.536618	6.027597	8.191037	4.949611	1.018041139	high
MB-5222	9.191780819	1	10.096366	4.942207	6.311912	5.026406	11.477474	1.537129364	high
MB-5097	5.063013699	0	9.550131	5.149323	5.806349	6.990401	9.137026	1.368849472	high
MB-5315	3.536986301	1	8.356265	5.523435	5.476641	5.582491	5.363573	1.194193266	high
MB-5226	16.48767123	0	8.024589	5.546278	6.470188	8.521214	4.779355	0.898308892	low
MB-5126	2.243835616	1	10.265495	5.002532	6.872649	4.658366	9.996662	1.406580272	high
MB-5279	5.652054795	1	7.645462	6.318067	6.672295	4.400262	5.555533	0.92569547	low
MB-4944	19.50136987	1	8.761861	6.983729	6.066328	4.662474	5.429486	1.05421835	high
MB-4961	18.03835617	0	7.84965	6.274979	6.515472	4.987796	7.862221	1.000778842	high
MB-5121	9.443835616	1	9.042506	6.501073	5.968065	5.934931	5.670146	1.098714465	high
MB-4886	3.95890411	1	9.150234	5.534817	5.329146	6.170609	10.235734	1.440638013	high
MB-4950	12.37808219	1	7.770362	6.036725	6.105552	4.964375	8.085642	1.078035029	high
MB-4965	21.66575342	1	8.712976	5.70108	5.985838	5.723233	5.363256	1.129443302	high
MB-4962	16.26027397	1	7.522928	4.973994	5.949315	5.424726	7.831663	1.143738995	high
MB-5267	5.84109589	1	8.318464	7.12745	6.12256	8.894766	5.790401	0.868037582	low
MB-5396	13.61643836	0	9.364897	5.072224	6.285643	9.733369	4.489755	1.038445109	high
MB-4938	5.802739726	1	8.806891	4.696183	5.483354	5.676687	6.104486	1.346600815	high

MB-5351	16.6109589	0	7.278567	6.505542	6.065145	7.70776	7.582164	0.899896989	low
MB-5347	17.41643836	1	9.305065	4.357349	6.808961	5.875497	5.262793	1.172705929	high
MB-5312	3.115068493	1	9.244969	5.049563	5.854765	6.393047	4.973676	1.234526467	high
MB-5311	4.739726027	1	6.452104	6.789721	5.802236	6.891243	5.223411	0.821576964	low
MB-5064	7.520547945	0	7.28512	7.857952	5.892003	5.460259	4.48482	0.834797914	low
MB-5061	3.24109589	0	8.786882	7.918108	6.279052	5.046359	4.698414	0.926527826	low
MB-4266	2.78630137	1	9.681953	4.239101	5.577921	5.174496	8.597309	1.611708859	high
MB-4276	10.86575342	1	9.213269	4.878562	5.580727	5.384202	11.821526	1.566124282	high
MB-5331	10.20273972	1	8.415176	4.963075	5.75805	8.489218	5.028632	1.086021755	high
MB-4743	18.91780822	0	5.760191	7.129727	5.816	8.099462	6.522201	0.739252261	low
MB-4785	10	1	8.565643	6.422329	6.673189	7.169254	4.47894	0.892965824	low
MB-4849	3.726027397	1	8.643464	6.283032	6.62215	4.819011	7.091657	1.051034504	high
MB-4784	11.97808219	1	8.208073	4.765387	6.21677	8.717767	10.257173	1.132484542	high
MB-5043	10.55068493	1	7.527631	4.830307	6.226498	7.322989	4.197547	0.9618472	low
MB-4992	13.59178082	0	7.726545	6.178979	6.180448	7.195099	9.83171	1.014982521	high
MB-5048	7.098630137	1	8.880153	5.712577	5.705458	6.601376	6.792955	1.196907438	high
MB-5035	17.52328767	1	8.296796	6.141836	6.318124	5.874262	5.151763	0.990950311	low
MB-5377	8.383561644	1	7.775525	6.709687	7.127955	6.762071	6.344184	0.802735816	low
MB-5403	7.868493151	1	6.569296	7.001051	7.223213	6.029655	7.820207	0.730468128	low
MB-5397	14.20821918	1	8.311494	5.626132	6.912306	4.611998	9.186092	1.085576095	high
MB-5378	1.008219178	1	10.128641	4.649868	5.869832	4.910008	10.477494	1.647131425	high
MB-5388	12.07671233	1	7.64765	6.024009	6.698442	6.597602	4.641062	0.859143393	low
MB-5393	12.65753425	1	8.04571	7.077252	7.799877	4.701189	4.989719	0.75656198	low
MB-5147	1.665753425	0	6.581536	8.617934	6.264314	5.62664	4.416119	0.693046834	low
MB-4140	9.410958904	1	7.670121	7.577117	7.382375	5.158371	12.316929	0.866160538	low
MB-5123	7.421917808	1	6.296065	8.941593	6.951222	6.364891	4.345319	0.581518932	low
MB-5065	15.1890411	0	8.763905	5.245944	5.527705	5.590099	10.442686	1.413663201	high
MB-5050	15.15068493	0	6.74809	6.47083	6.134895	9.708665	7.302391	0.789253448	low
MB-5062	13.83013699	0	8.226217	4.247201	5.966159	6.354473	4.801462	1.168955314	high
MB-5383	9.342465756	1	8.138951	6.664108	5.687803	5.954242	5.608253	1.032366617	high
MB-5258	8.306849318	1	9.255219	4.68615	5.741324	7.65069	12.33851	1.464136011	high
MB-5268	15.29863013	0	6.379774	6.889115	6.138083	6.685236	4.350797	0.762256365	low
MB-5261	9.572602742	1	8.325511	6.501073	6.288512	6.740774	4.355795	0.926071906	low
MB-5259	3.794520548	1	9.155626	5.132171	5.388486	5.988324	9.248461	1.450599073	high
MB-5306	13.12876712	0	9.421164	4.797477	5.75262	4.790514	5.264027	1.379096917	high
MB-5270	15.43835616	1	7.769217	6.392372	6.152414	5.297388	13.963065	1.177554654	high
MB-5411	2.136986301	1	10.586235	4.337932	6.557857	4.477087	7.766096	1.52670773	high
MB-5421	15.99178082	0	8.814372	4.864314	7.590349	8.314791	7.379616	0.932156913	low
MB-5405	9.665753425	0	8.877304	5.462059	5.876973	6.112088	5.043266	1.163191241	high
MB-5414	11.55616438	1	8.902725	5.152875	6.736896	4.533161	11.469868	1.290341414	high
MB-5481	3.810958904	1	9.306527	6.388012	5.922868	6.510331	11.267649	1.274766372	high
MB-5505	13.52876712	1	8.470516	5.519157	7.557347	6.754784	10.407271	0.971738967	low
MB-5485	2.61369863	1	9.194629	4.853947	7.05394	5.148288	4.970566	1.097830569	high
MB-4289	7.95890411	1	10.242036	3.872422	6.714475	4.755095	4.289621	1.371047876	high
MB-5293	17.17534247	1	8.279219	5.629776	6.907138	4.215578	5.991062	1.020225878	high
MB-4805	10.66849315	1	8.391461	4.490697	5.702926	4.294932	5.371815	1.312281467	high
MB-4809	23.51232877	0	8.938555	4.496204	6.240294	5.011698	6.131631	1.273571775	high
MB-5418	17.02739726	0	8.719997	5.245604	6.934378	9.181385	4.538217	0.8960265	low
MB-5441	0.797260274	1	7.294062	5.352489	7.545077	6.320208	12.811353	0.943250708	low
MB-5424	6.865753425	1	7.363067	6.817223	7.015236	8.885697	4.933512	0.701450312	low
MB-5230	14.50684932	0	7.67178	7.655273	6.213134	7.568156	5.350062	0.799875244	low
MB-5433	14.49589041	1	8.397108	5.563501	5.673461	5.588939	4.609672	1.142525901	high
MB-5427	12.8	1	9.282129	4.584511	5.890873	7.471073	12.107019	1.449700035	high
MB-5491	5.410958904	1	7.73782	4.582877	6.228926	8.175969	4.638597	0.981960643	low
MB-5493	4.704109589	1	9.053918	4.814486	5.124638	5.654317	5.069361	1.404975381	high
MB-5451	15.60821918	0	7.174947	6.292424	6.59034	6.870446	5.642989	0.827611808	low
MB-5229	2.791780822	1	8.434177	5.569995	5.840978	7.171869	10.663515	1.21852816	high
MB-5366	19.98630137	0	9.103241	5.043587	5.677775	7.575042	12.478427	1.424252937	high

MB-5382	9.553424655	1	7.556302	5.535887	6.973813	4.370255	4.391516	0.91090603	low
MB-4357	9.372602737	1	8.495545	4.918563	7.299302	4.899173	4.830032	0.991497316	low
MB-5390	2.542465753	1	10.976906	4.452287	6.89272	5.941623	6.137161	1.375832703	high
MB-5205	15.23013699	0	10.190387	5.053967	5.425758	5.217151	9.057357	1.63860741	high
MB-5292	3.980821918	1	9.856573	4.741735	5.968835	5.936617	12.720377	1.597732749	high
MB-5218	20.95616439	0	9.542592	4.87277	6.034263	7.507944	4.077481	1.185679005	high
MB-5163	17.44109589	1	8.334042	9.150721	5.628463	5.490008	5.236889	0.881563422	low
MB-5188	1.594520548	1	9.837218	5.958092	5.599269	6.599008	9.359761	1.38600771	high
MB-5329	3.75890411	1	8.531559	5.411727	4.634049	9.868592	6.390214	1.224457848	high
MB-5322	8.408219178	1	8.305287	6.214853	4.681834	9.725175	5.078363	1.090752867	high
MB-5310	12.6	1	7.227916	7.876318	5.095341	7.097125	3.830847	0.865397548	low
MB-5228	14.85479452	1	9.204659	4.372966	5.259296	4.577057	12.843229	1.789915964	high
MB-5215	9.756164384	1	8.468781	5.57106	5.276252	6.480671	4.812796	1.185881465	high
MB-5223	11.89041096	1	7.761267	7.363519	4.830717	7.065477	8.925531	1.105808626	high
MB-5179	20.26849315	1	8.143815	6.205659	4.845463	8.308951	11.284683	1.267550013	high
MB-5330	9.413698627	1	7.713559	5.715177	5.385289	6.312995	6.607465	1.124714084	high
MB-5196	7.728767123	1	9.074482	5.616339	5.123285	8.806986	7.104973	1.248158874	high
MB-5583	9.15342466	0	7.732214	6.679474	6.222858	10.319791	5.105689	0.786217052	low
MB-5601	17.65479452	1	7.791742	6.115887	7.056365	4.948982	4.555742	0.867136477	low
MB-5632	3.136986301	1	9.387778	4.424959	5.764979	6.232283	6.381365	1.380836307	high
MB-5616	14.84657534	0	9.873692	4.341633	5.752263	8.310414	10.084926	1.481158166	high
MB-5589	15.21643835	0	6.838303	6.402029	6.921671	5.871802	5.088492	0.773983084	low
MB-5582	18.2739726	0	6.450632	6.169909	6.77531	8.262156	5.346136	0.719878152	low
MB-5571	12.64657535	1	7.30104	7.247934	7.059272	5.100292	4.960433	0.760661434	low
MB-5629	8.531506849	1	8.254352	6.016847	7.020979	7.316907	5.172119	0.860661685	low
MB-5623	11.3260274	1	8.406188	4.597368	7.432879	5.633233	4.950679	0.967702324	low
MB-5654	14.22191781	1	7.890817	6.478034	6.755379	4.70865	5.668217	0.918230033	low
MB-5638	16.08493151	1	8.81649	6.833936	6.750454	4.466063	4.772121	0.965467318	low
MB-5642	12.88767123	1	7.501372	7.232372	6.108749	4.741855	4.708341	0.89319209	low
MB-5584	7.487671233	1	9.878594	4.699049	4.842083	6.699706	5.029348	1.541240882	high
MB-5626	6.819178082	0	8.132676	7.699619	6.37445	7.656503	10.418872	0.911410226	low
MB-5602	18.44657534	0	9.794406	5.382385	5.074906	5.418658	4.742926	1.454959671	high
MB-5653	15.99452055	0	8.899419	6.009751	6.469342	7.367941	11.94047	1.153168141	high
MB-5646	13.73424658	1	9.300435	5.330202	7.142801	4.73934	9.51965	1.186732964	high
MB-5634	5.169863014	1	10.676246	5.056478	6.05221	4.438077	5.795481	1.497858876	high
MB-5591	12.76986302	1	7.731074	7.439118	6.903883	7.022187	5.050973	0.750636926	low
MB-5613	17.45479452	1	9.116465	5.308031	6.083141	4.953244	7.556737	1.287543563	high
MB-5580	5.887671233	1	7.269674	9.660795	5.881441	9.365413	5.854029	0.657979984	low
MB-5645	1.82739726	1	7.47846	6.017248	6.663059	4.834045	4.832335	0.905168551	low
MB-5526	12.41643836	0	9.673451	4.654351	6.713141	7.92131	9.838778	1.24775246	high
MB-5474	18.63287671	0	8.436642	5.973786	6.493158	7.060861	10.002757	1.06555081	high
MB-5473	10.34794521	1	7.050272	6.724202	7.428061	5.052241	4.646073	0.729942991	low
MB-5484	19.60273973	0	7.808759	5.484085	7.347293	7.069036	4.589005	0.81640645	low
MB-5497	19.52054795	0	9.329672	4.708935	6.277019	8.869319	4.582709	1.099055886	high
MB-5300	15.63013699	0	6.617993	7.988963	7.112248	7.050501	9.806622	0.698469797	low
MB-5520	1.339726027	1	8.506017	5.588767	6.847249	6.300374	4.758415	0.957105214	low
MB-5529	1.216438356	1	9.681953	5.050503	7.039296	8.443078	13.86977	1.245908662	high
MB-5554	18.89041096	0	7.995515	8.432923	6.962997	9.050446	5.847286	0.673152519	low
MB-5483	3.487671233	1	8.064491	5.40135	5.876973	4.763601	5.305272	1.136869125	high
MB-5518	2.536986302	1	9.109896	4.534235	5.72355	7.105957	8.828346	1.376908899	high
MB-5486	10.13424658	1	8.695925	6.048698	6.397041	6.301735	9.149875	1.107505769	high
MB-5511	13.06575343	0	8.018131	4.971272	5.962704	4.90788	8.985391	1.250798156	high
MB-5422	3.78630137	1	7.802832	5.04831	6.568238	7.344322	4.397419	0.929124349	low
MB-5472	1.608219178	1	7.225094	6.630069	6.914168	5.566851	4.330963	0.784632227	low
MB-5294	16.10410959	0	9.705154	4.727345	6.50992	7.438966	4.854964	1.162041654	high
MB-4801	10.7150685	1	7.713559	5.917932	7.440395	5.429754	4.809702	0.819449351	low
MB-5459	7.463013699	1	8.879005	4.609746	6.981468	6.566454	4.81311	1.042098358	high
MB-4120	17.84383562	1	7.999339	5.770179	7.326575	5.283722	4.745923	0.86873972	low

MB-4966	4.052054795	1	7.851451	7.072763	6.761714	7.525642	4.812958	0.779541634	low
MB-4011	15.87671233	0	7.887185	4.877106	7.037346	6.468694	4.262613	0.911937957	low
MB-4822	7.531506849	1	6.873239	5.904038	7.079794	6.872881	4.515281	0.753550092	low
MB-4017	11.44931507	1	8.898267	5.095989	6.791113	6.451667	5.659892	1.056972218	high
MB-5018	8.926027397	1	8.279219	7.513577	7.478024	7.494246	6.948895	0.745887617	low
MB-4806	16.58356165	1	6.831799	6.160795	7.874827	7.906061	8.057936	0.688911513	low
MB-2964	0.789041096	1	8.014178	6.306072	6.225696	5.269473	7.736514	1.043770242	high
MB-2957	21.54520548	0	8.586789	5.003149	6.200214	11.537344	10.109454	1.04893699	high
MB-2916	21.93972602	0	6.576941	7.242551	6.631793	6.800554	4.69287	0.707955474	low
MB-2711	16.55890411	0	7.514658	6.551318	6.440473	8.571699	4.327761	0.785211311	low
MB-2730	10.99178082	0	8.819654	5.145804	6.533583	5.217151	5.262176	1.118820115	high
MB-2669	6.057534247	1	7.431837	7.178267	5.956206	5.460621	4.638174	0.887375828	low
MB-2735	22.56164384	0	8.324725	5.569284	6.396199	6.529925	4.691617	0.994585772	high
MB-3035	21.43013698	0	6.871195	6.315916	6.61471	7.860711	4.515654	0.754049945	low
MB-3060	3.665753425	1	9.321916	6.785205	6.090608	4.335776	4.772662	1.121488263	high
MB-3063	2.347945206	1	10.441306	6.337567	5.984312	4.73663	7.033668	1.363692439	high
MB-3367	14.7479452	0	9.505738	5.163899	6.792947	6.638746	10.979516	1.249998055	high
MB-3371	19.83013699	0	8.293703	7.212298	6.332921	9.88219	5.260643	0.798539515	low
MB-3360	2.2	1	9.298848	4.35269	7.628388	10.450886	4.778228	0.887342164	low
MB-3403	11.36986301	0	7.333668	6.668926	6.909007	8.73735	5.086413	0.724254766	low
MB-3350	18.72328767	1	7.440284	7.045204	7.155722	4.579017	4.705728	0.781515427	low
MB-3467	3.189041096	1	7.981367	5.046714	6.45999	4.832589	12.406473	1.251821429	high
MB-3298	19.9369863	0	7.587441	6.441888	7.268475	10.38476	9.201342	0.746380761	low
MB-3357	3.126027397	1	10.7163	4.487847	6.777106	8.136697	5.726225	1.252492722	high
MB-3365	19.85753425	0	6.251876	8.413673	7.125013	5.520034	4.924622	0.613352363	low
MB-2752	6.734246575	1	8.152176	6.917631	6.533151	7.494824	5.463902	0.852098667	low
MB-2745	13.88767124	1	8.310715	6.110547	6.62348	8.98543	5.456604	0.865124841	low
MB-2753	22.55342466	0	10.590741	5.264623	6.55872	7.687438	10.364574	1.355447826	high
MB-2966	21.78082192	0	8.243169	6.265663	7.514371	8.841518	5.613452	0.755383835	low
MB-2969	14.2109589	1	7.973026	7.50813	6.924467	7.955224	4.828576	0.735051287	low
MB-2947	9.205479452	0	6.131951	8.147038	6.865704	9.743199	5.479251	0.564909727	low
MB-2960	18.2630137	1	7.93342	7.004228	6.746375	10.775547	4.735896	0.709272391	low
MB-2971	21.71780822	0	7.200362	7.824231	7.126968	7.238136	5.04154	0.665924934	low
MB-2933	13.30958904	1	6.370275	7.039124	7.003777	6.833433	5.181581	0.675718787	low
MB-3487	19.49041096	0	9.329672	4.914735	5.647481	6.032645	5.392978	1.323108566	high
MB-3476	13.07397261	1	9.598095	5.694819	5.450775	5.959688	6.966052	1.365459805	high
MB-3452	9.852054797	1	7.819905	5.78264	5.802976	5.352074	4.468724	1.048211573	high
MB-3328	15.70958904	1	6.848551	6.99343	6.632678	6.379826	6.676889	0.78585039	low
MB-3277	2.706849315	1	9.16799	5.466255	5.738787	5.245697	5.8266	1.277559141	high
MB-3254	10.18356164	0	6.253642	6.711131	6.525824	7.794587	4.351447	0.696711101	low
MB-3275	3.41369863	1	9.450389	6.255556	6.154411	5.509615	8.462697	1.226037097	high
MB-3292	17.89863014	0	9.188929	5.101361	5.69358	9.243963	9.133071	1.250697524	high
MB-3840	23.60821918	0	8.114633	7.173022	6.644098	8.490765	4.70399	0.779253866	low
MB-2536	3.936986301	1	6.075079	6.653596	7.216671	9.657555	4.980869	0.59503866	low
MB-2513	4.906849315	1	8.129195	6.313761	6.691345	7.572733	4.686067	0.853701534	low
MB-2617	7.323287671	1	8.726134	6.039917	7.001872	7.13573	5.537857	0.914556185	low
MB-2614	5.336986301	1	7.676727	6.746456	7.29879	9.303193	6.217464	0.709250826	low
MB-2632	1.569863014	1	9.351913	4.433203	6.265549	4.970979	11.02613	1.485229506	high
MB-2556	18.15616438	1	6.08797	6.654069	6.271289	7.248856	11.908536	0.863104932	low
MB-2867	22.1369863	0	7.393838	7.554714	6.875437	10.448788	5.476437	0.651449551	low
MB-2922	1.183561644	1	9.086073	4.496816	5.879587	5.567624	11.340745	1.5018556	high
MB-2917	2.624657534	1	9.832365	4.88937	6.421487	8.553381	4.464503	1.123506513	high
MB-2858	11.63561644	1	8.913017	5.724468	6.069865	5.753178	11.602737	1.307556928	high
MB-2854	22.22739726	0	8.322346	6.176481	7.703387	9.201415	4.728606	0.722738151	low
MB-3706	3.189041096	1	8.762877	5.580456	5.846559	4.295224	6.657705	1.261596415	high
MB-3488	2.257534247	1	8.919953	5.000076	6.696269	5.279771	12.607656	1.316690574	high
MB-3711	18.25479452	0	7.535926	6.237539	6.437956	6.916241	5.726616	0.879572252	low
MB-3453	2.698630137	1	8.318464	5.038867	6.130908	4.551608	11.055999	1.326740234	high

MB-2763	22.65205479	0	7.618961	6.8695	6.577311	7.783561	4.765179	0.787059757	low
MB-2767	22.30136986	0	8.962065	7.668037	6.427804	8.534443	4.8215	0.840082851	low
MB-2790	9.610958901	0	7.828204	7.272769	7.43719	4.662474	11.628461	0.894216102	low
MB-2842	1.621917808	1	9.658829	4.432079	6.323467	5.009813	5.975029	1.351234807	high
MB-2774	10.82191781	1	7.971762	7.133705	6.975715	8.424117	4.453107	0.73318422	low
MB-2983	14.8630137	1	9.474983	5.947892	7.046113	6.666078	4.52351	0.976613861	low
MB-3025	21.04109589	0	9.520252	4.136968	6.963462	10.84225	9.316249	1.107677742	high
MB-2984	21.46849315	0	8.701961	4.917671	6.593822	6.797613	7.545586	1.115600923	high
MB-2845	21.76164384	0	6.337542	6.429867	5.99125	11.860208	4.48921	0.678188133	low
MB-2844	12.06027397	0	9.443489	4.630952	6.102363	4.301326	6.646014	1.397727607	high
MB-2847	3.249315068	1	9.244969	4.808887	6.426959	6.202351	5.816709	1.190258891	high
MB-2849	3.550684932	1	8.566585	5.484085	6.218393	6.293683	10.055893	1.196350872	high
MB-2848	18.71780822	1	6.933627	7.517754	6.021755	7.381214	6.410342	0.79725332	low
MB-2863	21.23287671	0	7.486058	8.682668	6.295909	4.788033	6.517183	0.807947728	low
MB-3016	21.27671233	0	9.72892	5.412782	7.085688	7.970774	12.47018	1.189788646	high
MB-3021	17.88219178	1	7.897568	6.224489	5.99512	9.330163	4.558644	0.872082453	low
MB-3033	5.78630137	1	6.621082	8.711925	6.492738	9.806959	4.766123	0.586639465	low
MB-3026	21.46849315	0	7.731074	6.276266	6.408422	8.572505	5.041784	0.836127833	low
MB-3006	1.290410959	1	7.015249	7.99163	6.421065	11.975897	4.800102	0.605951757	low
MB-3439	15.51232876	0	7.087127	7.762277	6.950738	9.028906	6.918056	0.667113845	low
MB-3395	20.03561644	0	9.12851	6.548565	6.066716	8.678415	11.164711	1.125799272	high
MB-3386	4.57260274	1	9.607989	5.066215	6.349073	5.830347	11.267649	1.400049615	high
MB-3382	11.19726027	0	9.482234	5.865709	6.318532	5.024203	4.835162	1.159004997	high
MB-3379	9.356164381	1	7.444345	6.717429	6.311912	5.030817	4.761973	0.890755213	low
MB-3389	19.78356164	0	9.61597	4.53794	6.84911	4.61513	4.984937	1.227876202	high
MB-3378	7.194520548	1	6.282488	7.713272	6.922134	9.743199	4.673174	0.577334442	low
MB-3079	12.29589041	0	7.881774	6.928027	6.76079	5.847136	4.798159	0.835827469	low
MB-3102	21.23287671	0	8.392271	5.226311	6.444718	6.763547	7.834028	1.088044368	high
MB-3123	13.76164383	0	9.839714	4.149073	6.140066	7.595213	5.088227	1.296969282	high
MB-3121	10.79452055	1	7.159212	6.393698	7.25111	10.448788	5.396705	0.6595294	low
MB-2838	22.21643836	0	8.793144	7.460313	7.190514	9.142172	5.719255	0.7544612	low
MB-2801	22.29589041	0	9.381192	6.371231	6.859759	9.272317	4.759871	0.886516728	low
MB-2834	12.64383561	1	8.760843	5.314154	6.263909	4.679006	5.126826	1.157669761	high
MB-2796	7.208219178	1	8.42834	6.403337	7.18059	11.475911	4.662464	0.714977619	low
MB-2819	22.23835617	0	7.140022	8.765978	7.17461	7.665902	4.759077	0.598996988	low
MB-2781	3.323287671	1	8.924551	5.67525	5.601016	9.127826	4.545515	1.068794926	high
MB-2793	21.96438356	0	7.382023	8.141019	6.458735	4.319085	12.47018	0.947890696	low
MB-2814	18.98904109	1	7.920995	6.34932	6.520652	6.897209	4.652856	0.873505669	low
MB-2791	22.35068493	0	6.264659	7.466994	6.208676	9.963171	6.050716	0.665289193	low
MB-2803	7.057534247	1	8.64824	5.106459	6.554792	8.712648	5.008854	0.974140022	low
MB-2786	5.610958904	1	7.798162	6.236709	5.75262	7.946233	6.87275	0.985475443	low
MB-3556	3.126027397	1	8.799438	4.584969	5.913648	5.12834	10.788919	1.447044464	high
MB-3707	18.80273973	1	7.506995	7.061246	5.912101	11.207482	4.703127	0.750796849	low
MB-3600	17.64657534	0	9.742284	5.544503	6.695371	4.480769	4.665247	1.171838041	high
MB-2642	3.712328767	1	9.31259	6.110547	6.112325	4.929584	5.612311	1.176032416	high
MB-2616	18.01369863	1	6.41868	7.855457	7.10786	7.515937	5.732795	0.621758962	low
MB-3842	18.58082192	0	9.817936	4.880881	5.799635	8.984521	4.994132	1.222648093	high
MB-3866	18.53424658	0	8.990697	5.12542	5.743513	5.13495	9.906593	1.418946989	high
MB-3874	15.32328767	0	7.161011	7.295951	6.654665	8.180775	5.214793	0.718607279	low
MB-3702	18.94246576	0	8.405377	6.744032	6.406304	9.424939	8.165617	0.898390945	low
MB-3228	20.71232877	0	7.028994	7.521939	6.17404	6.447501	4.324871	0.774526667	low
MB-3502	18.84931507	0	8.895964	5.161612	6.528415	4.467056	10.062329	1.288092806	high
MB-3752	6.191780822	1	8.636752	5.113234	5.521608	6.470514	4.595965	1.200688343	high
MB-3450	19.76438356	0	7.097305	6.055978	6.070262	5.649622	4.549625	0.91446521	low
MB-0476	10.72054794	0	6.40882	6.879796	6.137285	7.68323	6.110809	0.770255824	low
MB-0610	6.304109589	0	6.249392	5.849959	5.547584	4.72891	4.667672	0.952818825	low
MB-0451	4.115068493	0	7.472432	7.383328	5.462669	7.291719	5.05953	0.892968483	low
MB-0005	13.45479452	1	7.951488	6.976747	7.513805	8.343233	5.31322	0.702046197	low

MB-0053	13.23835617	0	8.509472	7.745815	8.228376	9.785343	4.685058	0.59318151	low
MB-0079	2.342465753	1	8.960888	6.485698	7.293146	9.435615	10.654176	0.90269904	low
MB-0014	13.50684931	0	6.394846	7.052996	8.454227	10.423635	4.799947	0.485258986	low
MB-0039	13.44109589	0	6.492226	6.441888	9.479692	4.260303	4.80176	0.5455631	low
MB-0054	13.17534247	0	7.898777	5.878454	8.124109	8.775412	7.174176	0.717445568	low
MB-0064	8.953424655	0	8.030475	7.423982	8.858809	5.986607	4.863457	0.605310578	low
MB-0107	12.98904109	0	9.913789	4.666855	7.923967	8.158005	4.98786	0.955879486	low
MB-4681	20.69589041	1	6.935746	5.883901	6.736451	6.641619	5.118356	0.814146901	low
MB-4626	11.75342466	1	9.485797	4.225857	7.140805	4.695328	4.727074	1.182017583	high
MB-4639	9.780821918	1	8.772154	4.346565	6.476519	4.41221	8.94254	1.333587041	high
MB-4711	2.58630137	1	8.367369	5.79852	5.93354	8.142879	8.93803	1.093090478	high
MB-4607	10.00547945	0	7.544279	5.331573	6.873125	5.030817	5.150922	0.933123212	low
MB-4669	9.819178085	1	9.023669	5.225303	4.949224	7.050501	4.596605	1.313778691	high
MB-4015	0.928767123	1	8.862183	4.508913	6.007407	8.683433	10.612856	1.278920624	high
MB-2931	18.74794521	1	6.850211	6.923869	6.383251	9.331294	4.745166	0.710089545	low
MB-2927	21.6739726	1	8.0665	6.741627	6.869872	7.48624	4.491291	0.799339394	low
MB-2944	2.235616438	1	7.499328	7.474306	6.473575	8.486028	8.41803	0.799758771	low
MB-2929	21.60547945	1	7.697235	6.268199	5.857018	10.073467	9.487445	0.94854487	low
MB-2724	8.882191784	1	7.558902	5.938172	5.85328	9.74594	4.45615	0.866247614	low
MB-2712	19.26849315	1	7.624852	7.206402	6.669244	7.459239	5.162151	0.772554693	low
MB-3062	12.03013699	0	9.698898	5.613429	5.916741	11.691856	4.694227	1.019804162	high
MB-3050	20.96712329	1	8.235806	5.162587	7.311136	6.149916	6.846742	0.95135618	low
MB-4722	7.076712329	0	6.696589	6.141411	6.579023	6.067256	5.021367	0.810994895	low
MB-4643	3.408219178	1	8.14938	4.903493	6.200214	5.004708	12.422047	1.327223646	high
MB-3363	0.893150685	1	10.168289	3.948339	6.678538	5.469056	4.544984	1.336057173	high
MB-3344	19.83013699	0	7.941491	6.061187	7.007102	8.09477	11.887624	0.946526811	low
MB-3381	19.44931507	0	7.77323	6.909058	6.007802	10.954411	4.575353	0.773243113	low
MB-3301	20.44657535	0	7.959054	7.95049	7.2465	8.012233	4.864182	0.677202129	low
MB-3088	21.08219178	0	7.790587	6.224904	6.866153	4.746605	5.19235	0.902037225	low
MB-2764	22.20273972	1	6.218193	7.312101	5.819725	11.221228	6.943998	0.692952718	low
MB-2744	22.65479452	0	6.367369	6.642792	6.147599	9.715554	5.628758	0.721290796	low
MB-7113	11.23561644	1	7.225562	7.068417	5.545873	5.896251	6.548185	0.956791236	low
MB-7130	8.463013701	1	9.582571	4.842799	4.969943	4.75319	11.887624	1.813947902	high
MB-7149	3.668493151	1	10.375599	5.348371	6.024873	7.716706	6.479072	1.300637491	high
MB-7208	3.775342466	1	8.954984	4.26492	4.590116	5.249628	10.389475	1.790659413	high
MB-7170	12.77260274	0	7.731074	5.256292	5.589109	5.377847	4.318645	1.110753991	high
MB-7174	11.56986302	0	9.618009	5.044542	5.583179	5.757198	4.748333	1.351654224	high
MB-7252	2.934246575	1	9.389453	6.138506	4.537249	6.417755	4.674034	1.37502773	high
MB-7244	15.1369863	0	6.593818	6.266089	5.699672	4.92666	5.104891	0.936758791	low
MB-7187	12.06849315	1	6.857579	6.051961	4.416734	7.820241	10.401303	1.199022647	high
MB-7251	1.77260274	1	8.848871	5.191937	5.760232	4.698639	5.413892	1.272728485	high
MB-7173	5.221917808	1	8.938555	7.440453	5.892377	7.798822	10.077658	1.062250041	high
MB-3001	21.63561644	0	10.248809	5.195912	6.692692	9.297598	10.228743	1.22258052	high
MB-7097	5.356164384	1	8.379336	5.903262	7.663352	6.780553	5.30718	0.819700948	low
MB-7104	10.09589041	1	9.552002	5.448016	6.334586	6.615615	5.149234	1.148559278	high
MB-7069	9.419178082	0	9.104564	5.344421	5.900383	7.562932	9.779227	1.269017757	high
MB-7100	12.84931507	1	7.811686	4.928656	7.072922	6.568832	7.622483	0.965183167	low
MB-7067	8.630136986	0	8.794197	5.302938	5.250077	7.667111	12.137334	1.423689954	high
MB-7073	11.3369863	1	9.434843	5.732688	5.673114	4.557907	11.654688	1.513860561	high
MB-7068	8.093150685	0	10.361052	5.027285	5.840978	5.605076	7.774396	1.509557757	high
MB-7075	9.175342463	0	9.607989	4.310301	6.241517	7.001136	6.647415	1.304900506	high
MB-7057	3.602739726	1	7.124399	6.893275	6.354056	7.832623	9.523295	0.859818936	low
MB-7051	7.063013699	1	9.596142	4.810272	6.514191	7.547457	4.780197	1.135817484	high
MB-7106	3.082191781	1	6.199234	7.27944	6.339136	9.988461	4.918092	0.641265792	low
MB-7107	4.679452055	1	8.144516	4.761036	6.549609	4.381517	7.276092	1.159734868	high
MB-7015	6.424657534	0	8.718993	5.775467	6.186074	4.395519	5.731248	1.151270225	high
MB-7013	3.816438356	0	7.198539	5.354884	6.523231	4.097029	4.778648	0.968048439	low
MB-7023	2.298630137	1	10.149747	6.925936	5.369075	4.654319	5.257876	1.330676069	high

MB-7024	5.775342466	0	9.671274	5.019797	5.638684	4.645522	11.797193	1.644940759	high
MB-7014	5.646575342	0	8.330918	6.331875	6.097633	5.194577	4.572864	1.020230317	high
MB-7017	11.12328767	0	8.498144	6.295807	6.226498	4.520193	10.015005	1.181611852	high
MB-3510	4.898630137	1	7.045482	6.336697	6.736896	4.254152	9.541358	0.951067556	low
MB-3396	18.63561644	0	8.003262	6.179794	5.362397	4.417464	11.145634	1.321753035	high
MB-7186	0.4	0	9.112526	5.086078	7.067582	4.729073	4.88678	1.08069347	high
MB-7142	14.45753425	1	8.544948	5.445902	6.471044	7.923209	4.710326	0.969364564	low
MB-7137	14.77808219	0	6.76167	7.409025	7.016675	5.662167	4.385019	0.695044404	low
MB-7181	12.9479452	0	8.364178	5.022591	6.229332	4.678444	4.901194	1.139335451	high
MB-7155	12.30958904	0	10.929993	4.869263	7.083219	5.993391	9.792819	1.400513784	high
MB-3295	13.03013698	1	5.608856	7.065097	6.681195	6.897703	7.395476	0.688121101	low
MB-3218	20.44657535	0	10.047488	6.524264	5.94047	7.777559	11.493125	1.30237361	high
MB-7281	4.071232877	1	8.488574	4.646151	5.781525	7.866955	11.005413	1.306409577	high
MB-7270	14.39726028	0	9.476764	6.406864	6.460857	6.251323	12.398617	1.24140912	high
MB-7231	16.35890411	1	7.284188	5.761908	6.498309	4.898256	5.170102	0.93249283	low
MB-7220	15.05753425	1	7.36503	7.360985	7.090657	4.55926	4.952615	0.768944392	low
MB-7277	15.37260274	0	6.917154	6.544398	6.714475	7.876962	6.417924	0.765763516	low
MB-7269	1.739726028	1	9.321916	4.721496	7.080289	6.574942	8.88382	1.166037676	high
MB-7278	19.83287671	1	5.979409	6.141411	7.466118	7.123182	6.973925	0.674111318	low
MB-2610	23.07123288	0	6.364334	7.164222	7.226187	10.283453	5.828091	0.586499385	low
MB-2517	22.0630137	1	9.034998	5.78755	5.900007	8.498469	10.016578	1.187251195	high
MB-2901	17.94246575	1	8.715977	5.30633	6.712242	5.370517	12.187693	1.242325156	high
MB-2900	1.698630137	1	6.277516	7.792574	7.273073	11.111138	5.895299	0.536281965	low
MB-7008	4.183561644	1	6.848966	5.643352	5.610526	10.978165	8.905052	0.909419066	low
MB-7007	4.816438356	0	9.381192	4.998525	5.609122	5.16798	10.827346	1.546706451	high
MB-7003	7.498630137	1	7.422878	8.357489	6.246395	7.29662	4.903136	0.735389107	low
MB-7010	6.084931507	0	6.527146	7.040771	6.500896	4.475549	12.368135	0.937612046	low
MB-7081	11.52602739	0	9.285103	5.323323	5.516504	6.875387	6.968503	1.310426555	high
MB-7093	10.34246575	1	5.915486	8.21986	6.003925	6.379385	7.303439	0.724067267	low
MB-7083	8.668493153	0	8.637719	4.55842	5.980039	8.54943	4.30866	1.089373838	high
MB-7065	8.865753427	0	7.916624	6.057164	6.184853	4.618658	4.424654	1.004808961	high
MB-7096	10.19178082	0	6.840774	6.523807	6.332514	5.897924	4.962623	0.830185106	low
MB-7088	10.15342465	0	9.139409	4.480945	5.751897	8.033973	7.798125	1.308051316	high
MB-7055	0.750684932	1	9.753404	4.557523	5.938948	6.19711	13.091692	1.610996086	high
MB-7115	11.64931507	1	8.822899	4.330746	6.199005	4.116396	8.688424	1.400941129	high
MB-7176	15.97808219	1	8.779505	5.546996	6.345746	4.428368	5.868849	1.154757528	high
MB-7164	17.37534247	0	7.262199	6.806188	6.122961	6.50569	4.954344	0.8537113	low
MB-7128	4.953424658	1	6.651065	7.972777	6.888944	8.697589	4.827748	0.612087649	low
MB-7092	10.06027397	0	9.057779	4.742269	6.214736	7.843803	5.053996	1.126646775	high
MB-7135	2.197260274	1	8.714968	4.859965	5.811192	5.176366	11.887624	1.459273636	high
MB-3754	7.621917808	1	8.347524	6.492916	6.103946	6.674661	5.95446	0.99052154	low
MB-3748	18.73972603	0	7.382023	7.006382	6.913713	6.565982	4.766657	0.755981828	low
MB-2770	22.55068493	0	7.451337	6.358939	6.569953	6.819649	9.393213	0.925041497	low
MB-2758	2.876712329	1	8.432497	6.296674	6.035429	7.006723	10.178372	1.114518884	high
MB-7152	16.09315068	1	9.813276	5.003774	5.684953	6.201905	4.855846	1.34531004	high
MB-7144	8.435616436	1	9.092633	5.192919	5.453399	4.816975	4.595321	1.330134488	high
MB-7185	10.27671233	1	6.643994	5.397925	6.543141	5.66532	6.40856	0.898355277	low
MB-7158	3.816438356	1	8.80049	4.919456	5.430694	10.01262	6.56005	1.165674977	high
MB-7163	4.326027397	1	7.570982	7.240177	6.303276	9.635032	5.082772	0.749416949	low
MB-7193	7.189041096	1	8.711018	4.955497	6.272931	6.780056	4.455972	1.086460148	high
MB-7198	1.375342465	1	8.38743	5.50039	6.069865	5.379629	5.511697	1.114938197	high
MB-7258	1.238356165	1	10.368337	5.530198	5.341892	4.155278	12.33851	1.814674316	high
MB-7254	15.79726027	0	7.843637	5.266277	6.865257	6.431069	11.508838	1.065786141	high
MB-7262	8.550684929	1	10.047488	4.4635	7.717971	4.100242	4.303309	1.140970995	high
MB-2993	15.37260274	0	9.300435	6.188945	6.063559	9.701897	8.58808	1.073268315	high
MB-3031	2.928767123	1	8.440851	5.080406	8.066938	7.465981	8.340174	0.869317039	low
MB-2904	10.32328767	1	9.527672	6.6296	5.407887	4.17151	11.721793	1.500905228	high
MB-2846	12.30410959	1	9.267165	6.700033	6.114671	7.846328	11.309149	1.155300033	high

MB-7229	9.17260274	1	7.621057	6.084605	7.104941	6.146462	10.739744	0.939187478	low
MB-7165	12.09589041	0	10.46143	4.669385	7.023861	4.428989	11.046814	1.485837899	high
MB-7150	10.6	0	8.846636	5.457468	5.005765	7.197241	5.988996	1.292655832	high
MB-3462	19.25205479	0	7.537515	6.802715	6.582936	6.317472	5.12054	0.830183345	low
MB-2821	11.18356165	1	7.112837	8.373758	6.297527	11.09529	4.585971	0.61925287	low
MB-2820	20.92876712	0	7.90061	6.788219	7.261342	11.190319	5.377389	0.669145292	low
MB-7284	16.68493151	1	7.877534	7.184697	5.444179	8.413537	5.099277	0.911561826	low
MB-7279	1.750684932	1	10.02528	4.015734	5.356981	4.335202	12.865109	1.981137642	high
MB-7233	18.24931507	1	7.47442	6.395858	5.682108	6.109484	5.697132	0.986252325	low
MB-7250	4.191780822	1	8.442541	5.141889	4.956669	5.365959	6.912659	1.392241888	high
MB-7285	8.95890411	1	7.992957	7.134818	6.176848	8.775412	4.65232	0.81626434	low
MB-7260	2.561643836	0	10.158977	4.650365	6.235007	3.997599	4.246693	1.403868487	high
MB-7264	3.531506849	1	8.24466	5.271482	6.527562	6.481127	4.486488	0.9883789	low
MB-7268	18.69589041	0	8.23655	5.472897	6.5397	6.102603	7.219763	1.045984927	high
MB-7276	15.23287671	0	7.388931	7.177114	6.744984	6.710829	5.990236	0.782310833	low
MB-7267	16.05753425	1	10.843321	4.824319	6.404217	5.511466	8.323271	1.507177488	high
MB-7216	17.0520548	1	7.240434	6.302639	6.420221	4.764677	4.563907	0.892066111	low
MB-7195	6.610958904	1	5.263308	5.341494	6.668358	6.184878	4.662928	0.733044351	low
MB-7217	7.430136986	1	7.333668	6.406414	6.648073	11.341692	6.772867	0.730622341	low
MB-7124	11.4739726	0	6.744125	5.363115	6.881942	4.322263	4.973452	0.877637121	low
MB-7122	2.457534247	1	9.466061	5.429856	5.937048	8.690039	7.251476	1.181061886	high
MB-7084	9.578082189	0	10.116774	4.359378	6.498735	4.396334	8.355841	1.492726665	high
MB-7101	10.21369863	0	6.553292	6.742097	6.448948	9.237508	7.052758	0.732969019	low
MB-7116	2.742465753	1	8.144516	5.728179	6.885218	4.790701	4.605782	0.953054954	low
MB-7131	11.0739726	0	8.574818	6.687161	6.632678	4.559716	5.391621	0.98063602	low
MB-7090	9.021917811	0	9.457324	4.246135	6.169215	4.649565	12.240404	1.603255915	high
MB-7094	9.317808222	0	9.656699	4.097109	6.223267	4.625387	4.460642	1.375992326	high
MB-7087	0.416438356	1	9.474983	4.105895	7.433418	4.03291	4.804023	1.171169077	high
MB-7077	8.323287674	0	6.503793	6.623954	6.427804	4.814172	4.619202	0.809349722	low
MB-7041	8.380821921	1	7.131952	7.556131	6.07533	10.66288	11.184253	0.803627963	low
MB-7044	7.136986301	0	7.174947	7.60935	5.615097	12.239946	5.278264	0.711484165	low
MB-7070	8.882191784	0	8.674335	5.96634	5.431984	8.661845	5.448342	1.083008422	high
MB-7079	7.484931507	0	6.272493	7.649346	5.603161	10.075139	7.888285	0.742650346	low
MB-7025	6.594520548	0	8.518273	6.530678	5.413392	10.617799	5.093228	0.951806343	low
MB-7036	9.898630134	0	8.947938	4.8803	5.544489	4.990958	7.248303	1.400942323	high
MB-7046	12.03287671	1	9.096545	4.263311	5.857018	5.319167	4.881524	1.336976652	high
MB-4839	24.60821918	0	8.079278	6.669881	5.91326	9.371154	4.771022	0.870436886	low
MB-5559	1.547945205	1	8.411131	5.8465	5.657438	6.173495	7.339613	1.170681768	high
MB-4744	12.57534247	1	9.259741	5.517375	6.329229	5.068363	6.948895	1.219050847	high
MB-4769	1.783561644	1	9.358444	4.362836	5.937806	4.435949	7.879115	1.483025309	high
MB-5558	2.057534246	0	8.265608	6.228686	6.294286	7.326824	5.459731	0.944825548	low
MB-4793	6.852054795	1	10.502766	4.481353	5.846184	6.357192	4.812796	1.454422364	high
MB-5513	3.010958904	1	8.8112	4.471375	6.476938	4.927393	4.434248	1.176281836	high
MB-5656	10.3890411	1	6.426481	7.047967	5.971118	6.271788	5.505661	0.805955834	low
MB-5553	12.69863014	1	8.221104	6.518778	6.458735	9.894182	4.750754	0.812118933	low
MB-4679	24.05479452	1	10.171405	4.881764	5.854019	4.293071	11.164711	1.688103211	high
MB-4033	1.249315068	1	7.852047	5.554529	6.061574	10.248151	4.618348	0.879817563	low
MB-4692	8.816438359	1	8.583122	5.314839	6.976654	4.06818	5.531769	1.060062229	high
MB-4675	21.61917808	0	6.629596	6.832398	6.521515	5.492967	8.701376	0.853417776	low
MB-4663	19.67123287	0	10.357294	4.254679	6.250426	4.225393	9.542583	1.647924254	high
MB-2618	7.323287671	1	9.047617	5.121562	7.303919	9.20037	4.687903	0.888784279	low
MB-2718	22.87123288	0	9.703066	5.013308	6.559151	4.31224	7.909178	1.342232275	high
MB-2643	4.353424658	1	7.773812	5.01671	6.322632	12.53149	4.838337	0.817300639	low
MB-2645	3.679452055	1	10.190387	5.54017	7.151721	6.186155	8.206504	1.17537726	high
MB-2626	15.92054795	0	8.235806	6.697166	6.827446	10.809726	4.81017	0.739701961	low
MB-6305	7.35890411	1	8.650163	5.495401	5.869832	6.694863	9.460485	1.232066194	high
MB-6185	16.25205479	1	6.29785	5.613429	6.201418	10.259294	4.571889	0.738169856	low
MB-6248	15.02739726	0	9.191783	4.145224	5.338478	5.693208	7.340704	1.529908868	high

MB-6184	15.2630137	1	10.477788	4.557523	6.270462	4.399437	6.062757	1.491907595	high
MB-6329	6.698630137	1	9.718001	4.559228	6.008476	5.018132	4.619394	1.363690037	high
MB-6337	9.11232877	1	9.494854	4.937975	6.081595	4.264702	10.262593	1.495425816	high
MB-6256	10.50684931	1	7.200362	8.203077	6.280291	7.417837	4.811713	0.72053053	low
MB-6201	18.30410959	1	7.880566	6.316348	6.518491	7.552621	11.504853	0.99762469	high
MB-6194	4.312328767	1	7.681662	4.27463	6.206646	9.004638	8.415832	1.063544844	high
MB-6223	18.24109589	0	10.409729	5.102632	5.603161	9.361986	12.260679	1.523795326	high
MB-6226	2.906849315	1	9.363322	6.17482	6.455345	5.795863	5.482434	1.08544726	high
MB-6017	5.82739726	1	6.367369	7.528846	6.47527	4.763601	4.382216	0.73714625	low
MB-6019	1.112328767	1	8.364974	4.573167	6.020199	8.131199	12.494791	1.287158149	high
MB-6163	7.287671233	1	7.251575	7.225268	5.621042	6.931742	5.069622	0.875989729	low
MB-6157	1.304109589	1	9.373019	5.315868	5.651001	9.101345	4.589499	1.141536481	high
MB-6105	15.79726027	1	8.806891	7.415484	5.760962	7.62658	4.632678	0.951354164	low
MB-6047	3.112328767	1	10.656751	4.745701	6.122168	4.668089	11.520758	1.713881215	high
MB-6001	11.12054795	1	7.356273	5.489721	6.047905	9.138082	7.641894	0.938669148	low
MB-4564	17.29589041	1	7.593325	4.64267	7.03396	5.045521	8.086934	1.032825651	high
MB-4593	11.52602739	0	7.665194	5.39483	7.520909	5.79182	5.564285	0.843990625	low
MB-5186	16.70684932	0	7.982633	5.861844	6.852368	6.100449	4.913235	0.899462089	low
MB-4974	14.4739726	1	9.849226	4.464485	5.193406	4.414182	4.367319	1.58303442	high
MB-5086	6.923287671	1	7.501372	5.047038	6.280712	5.814119	4.348287	0.987865714	low
MB-5345	17.75616438	1	7.228374	7.999836	6.201418	5.377139	5.462161	0.806047685	low
MB-4696	20.98082192	0	9.065463	4.487223	6.337467	4.827416	11.237002	1.437937855	high
MB-4705	17.51506849	1	6.009069	6.612194	6.168304	9.285481	5.21079	0.699764535	low
MB-4882	21.87123288	1	6.74728	6.619272	6.401298	4.856431	5.192929	0.841711404	low
MB-4846	8.353424655	1	9.283621	5.30023	6.522811	7.718502	4.690369	1.051719513	high
MB-4870	18.82739726	0	7.922869	5.697018	6.165609	9.085288	5.783813	0.921530306	low
MB-4865	18.89589041	0	9.611985	5.309064	5.743513	5.13136	6.895083	1.387249418	high
MB-4876	12.37808219	1	8.474807	6.7513	5.854019	8.335886	5.318683	0.949620944	low
MB-4897	16.22739726	0	6.958358	7.095768	6.184446	9.009447	5.709299	0.752317698	low
MB-4925	8.446575345	1	8.24988	6.454344	6.020592	8.483702	5.339001	0.924629905	low
MB-4883	22.10136986	0	7.299202	6.547157	6.938583	5.911199	4.85321	0.792936133	low
MB-4987	18.22739726	1	6.317974	6.773351	6.349073	12.107655	4.734572	0.625120364	low
MB-4374	3.498630137	1	7.55786	4.559318	7.263907	4.678723	4.878932	0.943860409	low
MB-4407	6.852054795	1	9.324961	3.875672	6.993317	5.152546	11.698879	1.408837141	high
MB-5039	5.106849315	1	8.653986	4.465677	5.551377	5.205636	4.625945	1.314069681	high
MB-4119	5.57260274	1	9.464321	5.041374	7.204574	4.230543	4.306901	1.104155658	high
MB-5079	16.43287671	0	7.472928	5.641506	5.987005	8.798207	7.909805	0.962906476	low
MB-5073	15.95890411	1	7.314711	7.843057	5.859646	10.726763	4.492196	0.706825756	low
MB-5105	16.80273972	1	8.003262	4.822355	6.111111	9.443898	5.607526	0.985449865	low
MB-5174	12.42739726	1	9.201765	3.964507	5.764979	5.108296	8.530964	1.531957766	high
MB-4282	2.802739726	0	8.057743	5.280739	6.772146	5.266816	4.74795	0.981610964	low
MB-4853	21.85753424	0	5.676376	7.018295	6.599476	9.999593	5.35269	0.605485424	low
MB-4362	21.96712329	0	6.480578	5.112912	6.625661	5.412605	4.466607	0.862189023	low
MB-4602	10.09315068	1	9.715841	4.433017	6.864812	4.692374	4.905898	1.241653902	high
MB-4893	14.52328767	0	9.057779	6.688097	5.615097	8.226053	5.643745	1.055590181	high
MB-4873	9.671232879	1	8.445893	5.406523	6.143647	6.787343	5.136998	1.057561459	high
MB-4942	10.20273972	1	8.752616	4.777911	7.767883	4.925933	4.646593	0.956997323	low
MB-5313	3.824657534	1	8.899419	5.25094	5.582828	6.009582	4.837173	1.233395042	high
MB-4145	17.88767123	1	7.766388	5.227998	6.582503	5.181701	4.010984	0.970658062	low
MB-5278	8.216438356	1	6.247963	6.318932	5.851023	7.716706	6.473662	0.830473166	low
MB-5470	3.457534247	1	9.166599	5.865315	5.537656	7.113745	5.174451	1.179846736	high
MB-5317	4.408219178	1	8.376966	6.178979	6.706893	8.131884	4.800255	0.867568734	low
MB-5308	8.610958907	1	7.686656	6.756223	6.247595	8.954301	6.813137	0.843563709	low
MB-5277	18.16986302	1	7.806397	5.466607	6.247595	6.269541	5.63136	1.003476463	high
MB-4046	24.49589041	1	8.984753	5.384818	7.070482	5.291056	5.082772	1.027945773	high
MB-4012	22.79726028	1	8.23729	4.555949	6.206239	5.530971	11.347857	1.317391057	high
MB-4001	4.082191781	1	8.831546	4.506184	6.792503	6.889741	5.210307	1.072031111	high
MB-4792	3.339726027	1	10.235385	5.47534	5.825685	4.506538	10.882942	1.607070483	high

MB-4782	20.63561644	0	5.853383	6.969772	6.623038	8.503187	9.692496	0.714731999	low
MB-5008	18.35342466	0	10.485902	4.174722	6.497031	4.401135	5.582155	1.473311983	high
MB-4426	14.04657534	1	8.601678	4.084248	7.928391	6.561783	5.226398	0.933549716	low
MB-4434	12.17260274	1	9.191783	3.977424	7.035872	4.90247	11.528818	1.377843694	high
MB-4368	9.819178085	1	7.967325	4.820936	7.064141	6.148209	5.104891	0.947343055	low
MB-4442	24.4	0	8.194227	4.566334	7.197999	4.982069	10.529778	1.139568914	high
MB-4418	25.2849315	0	8.530649	4.296434	7.357204	5.576361	5.193232	1.020691334	high
MB-4395	11.4739726	1	10.289643	4.187482	7.595439	5.090286	4.676842	1.184566828	high
MB-4630	7.876712329	1	9.196061	6.691449	7.292124	4.965391	6.250779	0.954233798	low
MB-6010	17.9369863	0	9.404357	5.107107	6.773512	7.920682	4.897515	1.040450073	high
MB-6154	16.05205479	0	8.34594	5.351786	6.637945	8.012888	4.717762	0.932991755	low
MB-6107	10.1369863	1	8.921132	6.653127	6.142865	7.332856	9.783275	1.097512775	high
MB-6152	18.81369863	0	9.803759	5.11995	5.460675	5.632831	11.573745	1.632168206	high
MB-6018	11.76712329	1	8.050311	5.837585	6.642251	10.211172	5.173461	0.819758373	low
MB-6082	8.424657534	1	7.969835	6.253043	5.943912	11.487991	9.880482	0.927076834	low
MB-5295	6.221917808	1	10.445294	4.765657	6.699775	6.402187	4.597535	1.246662272	high
MB-5386	15.79726027	0	8.437493	4.616962	6.928266	9.868592	9.216565	0.995862285	high
MB-6022	4.279452055	1	8.653986	4.565427	6.03619	9.178268	4.523013	1.064685486	high
MB-6053	20.61369863	0	8.784758	5.212043	6.313973	8.025388	4.365973	1.021199739	high
MB-6135	6.460273973	1	8.804788	5.046405	6.4295	7.768576	4.470134	1.030801879	high
MB-6058	1.863013699	1	8.938555	5.714069	6.344929	6.803964	4.474445	1.036397012	high
MB-5368	8.230136984	1	7.43481	6.30435	7.534045	6.842777	4.205237	0.719090001	low
MB-5335	2.073972602	1	8.728151	4.482569	6.8012	5.950883	10.626485	1.239220853	high
MB-4008	3.676712329	1	8.196429	4.901449	6.780757	8.506306	4.586516	0.915305146	low
MB-5130	20.95890411	1	7.864159	6.119982	6.680741	8.451449	5.57485	0.838373246	low
MB-5054	15.42739726	0	8.672341	6.41436	5.277143	5.504785	11.351284	1.357820156	high
MB-4760	10.25753425	1	7.523439	8.267578	6.154816	6.14513	6.215755	0.810833716	low
MB-5102	2.495890411	1	10.441306	4.106171	6.402961	6.713212	10.749571	1.556278451	high
MB-5492	1.920547945	1	6.876146	8.241977	6.545741	10.911243	4.908052	0.598450028	low
MB-5407	16.89863014	0	7.118648	5.647369	6.408005	5.24307	4.546519	0.914000724	low
MB-5406	8.008219178	1	9.331217	4.521838	5.796668	5.352767	4.622433	1.342543055	high
MB-4329	13.83013699	1	7.460395	6.672763	6.439217	6.091449	5.092173	0.855226531	low
MB-6272	1.84109589	1	8.917616	5.135092	5.596112	8.829872	7.950087	1.215983444	high
MB-6217	17.38630137	1	9.632093	4.681789	5.532866	5.197104	7.431302	1.517994336	high
MB-6233	16.53424658	0	8.232144	6.746925	5.504963	9.355154	4.476064	0.924387619	low
MB-4281	21.37260274	1	10.143631	4.018591	6.816324	4.185844	4.528948	1.356375958	high
MB-4283	14.36986301	0	6.74969	5.909817	6.745903	3.910649	6.695679	0.904631009	low
MB-4303	7.317808219	1	9.096545	5.006239	6.798916	6.362192	5.738225	1.088844046	high
MB-4292	27.58356164	1	8.222579	4.669385	7.448408	4.894497	5.929996	0.988599808	low
MB-4278	20.07123288	1	8.686049	4.305715	7.562348	5.039681	5.224629	1.024709404	high
MB-5399	8.778082192	1	7.197623	7.072763	7.08617	8.13804	4.049377	0.673330751	low
MB-5364	10.3369863	1	6.651065	6.246745	7.171647	8.403782	9.614056	0.756660718	low
MB-6100	0.827397261	1	9.286577	5.380988	5.913648	4.637349	12.617038	1.511157592	high
MB-6085	1.964383562	0	8.133372	4.848221	6.246395	5.684146	9.996662	1.223359424	high
MB-6113	10.23835617	1	7.50597	6.361124	5.601384	6.515939	4.592222	0.965247825	low
MB-6131	21.17808219	0	9.694586	4.688974	5.462343	6.803477	10.246395	1.557912298	high
MB-6114	5.493150685	0	9.120447	5.601455	5.734395	6.689593	10.479698	1.334108056	high
MB-6092	0.189041096	1	7.69389	5.656539	6.373211	9.74594	5.643745	0.856355	low
MB-6011	17.35342465	1	9.043768	4.241902	6.931076	6.282492	4.409856	1.09737035	high
MB-6346	23.1369863	0	8.67724	5.281413	6.805781	6.075472	11.068033	1.165454347	high
MB-6319	12.71506849	0	8.06984	6.10445	6.468068	10.957324	6.351665	0.826150175	low
MB-6200	2.471232877	1	7.652552	6.008537	6.150804	8.220423	11.319546	1.025101471	high
MB-6211	3.476712329	1	7.499328	5.410346	6.034263	7.447407	5.031547	0.956437571	low
MB-6234	17.5369863	0	5.920406	7.760693	6.05221	6.72725	4.81436	0.696380186	low
MB-5533	12.98904109	0	8.037828	5.945598	5.914028	9.088306	10.601673	1.056524945	high
MB-5543	2.830136986	1	7.426328	6.216951	5.56859	7.425575	4.717645	0.94677606	low
MB-4270	8.112328767	1	10.036423	4.35408	6.016711	5.996818	10.62196	1.583190125	high
MB-4235	27.59452055	1	9.308008	4.457458	5.813064	7.850712	5.709299	1.266960822	high

MB-4250	8.232876715	1	7.69501	5.102632	5.861923	6.587203	4.861673	1.048250259	high
MB-4323	10.40821918	1	8.143815	4.662358	7.831589	5.598929	8.233896	0.957366262	low
MB-4333	17.09315069	1	6.180992	5.584413	7.638632	6.313429	4.938731	0.686933042	low
MB-4339	14.26849315	1	8.807962	4.689228	7.609644	5.170768	4.521547	0.979748575	low
MB-4375	23.93150685	0	6.823265	5.005014	7.942984	5.874669	6.833713	0.775650752	low
MB-4798	21.1369863	0	7.95525	5.08292	6.505639	8.150426	5.038363	0.937149448	low
MB-5426	2.846575342	1	9.54822	6.421866	5.941612	8.446847	11.06494	1.211438297	high
MB-5444	19.76164383	0	6.744529	9.470915	7.13291	5.733537	5.05625	0.589664825	low
MB-5339	16.09863014	0	8.23141	6.73242	5.985074	6.637795	9.032845	1.049909761	high
MB-5208	12.48493151	0	8.861081	4.87306	5.179822	5.776511	4.992744	1.354350825	high
MB-5428	12.36712329	0	7.08978	5.535174	6.253275	5.204366	5.088751	0.952528927	low
MB-5440	16.16164383	0	9.963175	4.824895	5.59541	6.56457	10.216436	1.564027896	high
MB-5298	6.978082192	1	10.943118	4.461565	5.755879	5.097924	10.672848	1.833745216	high
MB-6014	15.77534246	1	5.964146	7.487031	6.942331	7.946233	4.97527	0.607273156	low
MB-6145	2.821917808	1	10.563764	5.008083	6.327161	9.473086	9.452497	1.315031826	high
MB-6012	11.87123287	1	7.950232	5.693341	7.662779	4.928121	12.174996	0.9937897	low
MB-6124	9.504109586	1	7.841857	6.574791	7.078813	6.363526	4.735661	0.80308946	low
MB-6144	20.95342465	0	9.088731	4.871324	6.301221	8.14629	7.251985	1.150234646	high
MB-5346	15.96986301	0	10.053131	4.220948	6.167604	6.543913	6.235661	1.394048974	high
MB-5373	0.208219178	0	7.666846	7.975464	6.829733	6.952422	4.229024	0.711507323	low
MB-6063	1.282191781	1	8.614905	4.250524	6.528415	5.451893	10.931674	1.327695255	high
MB-6069	21.21643835	1	7.257556	7.195762	6.294286	7.381214	7.175205	0.825730792	low
MB-6141	6.482191781	1	8.258823	5.082608	6.34327	9.969288	4.750497	0.923328918	low
MB-6118	21.74794521	0	7.625943	7.068955	6.875437	9.519806	5.854029	0.719514961	low
MB-6065	15.49589041	1	7.761267	5.714427	6.31522	8.643651	5.426439	0.893009883	low
MB-4310	21.82739726	1	9.188929	4.082152	8.203319	4.035574	4.630632	1.019963337	high
MB-4350	2.553424658	1	8.979906	4.459215	8.111094	4.617975	12.691494	1.158683497	high
MB-4324	7.169863014	1	9.384477	4.003142	7.007588	5.282407	4.97412	1.196636169	high
MB-4306	19.29041096	1	7.257556	5.062421	7.635783	5.396633	5.942593	0.83720415	low
MB-7205	13.64657534	0	9.104564	5.552002	5.990869	5.952137	9.285254	1.286341894	high
MB-7159	1.802739726	1	10.394435	4.322747	6.410527	6.766967	11.446993	1.543484987	high
MB-7207	4.580821918	1	8.967944	5.279397	5.532866	5.603548	10.383732	1.434420352	high
MB-7237	1.967123287	1	8.3979	5.849959	6.300387	11.27002	4.857952	0.851797192	low
MB-7236	16.55890411	1	7.740095	6.098832	6.31934	7.356932	4.388604	0.881378571	low
MB-7235	14.03835616	0	8.020715	6.496083	6.338283	9.570025	6.228331	0.848442158	low
MB-4331	19.36164384	1	8.768015	5.686713	7.174108	9.910825	4.790728	0.825703199	low
MB-4328	16.37808219	1	7.545855	5.270979	7.232793	5.85246	4.213188	0.849009141	low
MB-4348	18.59178082	1	11.120412	4.088501	6.473153	6.351342	12.168553	1.722085611	high
MB-6030	11.94794521	1	7.666846	5.323323	6.721622	10.63191	5.550444	0.808477367	low
MB-6133	5.079452055	1	8.64824	7.027576	6.643654	9.254816	10.581743	0.924493478	low
MB-6168	18.55068493	1	6.741712	7.451694	6.731091	9.773914	5.150922	0.638841495	low
MB-6156	4.843835616	1	8.770067	4.977346	6.298338	4.436984	10.436637	1.346049959	high
MB-6051	15.35616438	1	6.175072	6.023217	6.418065	11.492054	5.374114	0.670078921	low
MB-6143	21.00821918	0	9.3376	6.881882	6.060025	7.856923	5.800547	1.019459722	high
MB-6164	16.92328767	0	7.69501	7.035274	5.970738	9.257017	6.504484	0.844552045	low
MB-0551	4.271232877	1	5.957184	7.963726	5.134308	9.575174	8.418766	0.77299006	low
MB-0391	11.70958904	0	8.080628	5.161286	4.561697	7.033044	6.906777	1.34257356	high
MB-5171	15.20547945	1	6.159701	6.809721	5.422821	9.116613	6.707915	0.808083254	low
MB-5199	16.8	0	8.802622	5.648452	5.40591	8.022728	5.65315	1.157484807	high
MB-5177	5.361643836	1	8.777392	5.489014	5.300241	5.155597	4.423537	1.269244596	high
MB-4024	10.2	0	7.959694	5.661692	6.768046	4.541482	5.483138	0.984090622	low
MB-6314	11.08219178	0	9.834777	4.624561	5.572625	4.524513	11.897914	1.750006801	high
MB-6358	3.112328767	1	8.306851	5.019195	5.562405	8.759013	4.870498	1.086255662	high
MB-6254	5.005479452	1	7.35581	6.342827	5.568927	10.150821	4.779643	0.851733677	low
MB-6204	4.665753425	1	7.064272	7.273391	5.653883	8.921538	5.059021	0.798343657	low
MB-5154	14.04109589	0	7.555773	6.278414	5.228848	7.494246	11.167954	1.156265149	high
MB-5162	13.50684931	0	7.528133	5.271315	5.428373	11.725037	4.244948	0.899609556	low
MB-5164	6.616438356	0	9.088731	5.715177	5.464661	5.474951	5.679976	1.278569246	high

MB-7253	4.928767123	1	8.059779	5.172053	7.257252	5.672773	4.450897	0.906144284	low
MB-7298	7.087671233	1	8.141066	5.50425	6.250829	5.411536	7.605979	1.111579035	high
MB-7299	16.59452055	1	9.236234	5.000379	6.556986	4.475024	4.517916	1.183110354	high
MB-7293	16.37534246	0	6.93532	7.987172	6.479894	7.690409	4.191593	0.678465992	low
MB-2923	1.15890411	1	8.794197	4.215457	7.03396	5.420816	4.421868	1.089074813	high
MB-0381	10.70136986	0	8.584038	4.129931	5.267892	4.187275	7.853739	1.551881983	high
MB-5271	13.76712329	0	9.785286	4.603303	4.846743	4.207604	4.191019	1.63911048	high
MB-7031	7.016438356	0	11.234576	4.16003	6.654665	4.978695	12.082932	1.763234771	high
MB-7050	5.353424658	1	8.687019	5.023534	5.525995	4.410666	10.46509	1.485754183	high
MB-7054	2.501369863	1	10.375599	4.938873	5.261377	4.74937	4.791608	1.587839396	high
MB-7048	7.271232877	0	8.774282	5.737137	6.175248	5.174178	6.472758	1.152160911	high
MB-7056	9.616438356	0	6.958358	6.228262	6.279475	4.519798	6.311252	0.934268284	low
MB-7086	10.15342465	0	8.189888	5.100407	6.718933	5.533907	5.515215	1.024455848	high
MB-0177	3.747945205	1	6.788324	7.214089	6.032313	7.308109	4.90197	0.778435464	low
MB-0399	11.26849315	1	8.130576	5.60618	5.402026	4.55618	10.907241	1.377034276	high
MB-0615	2.857534247	0	7.895102	5.398943	5.960797	10.131515	10.31941	1.036402808	high
MB-0265	15.54520548	0	7.682202	6.835945	6.615584	8.975063	5.07401	0.764328458	low
MB-0558	8.04109589	1	8.915269	4.842799	5.662421	4.539519	8.621588	1.442672556	high
MB-0611	8.591780819	0	8.398733	6.477136	6.637508	5.014966	4.74367	0.950418465	low
MB-0640	5.04109589	0	6.741298	7.081153	6.544009	10.147305	6.083362	0.680866399	low
MB-0114	1.101369863	0	7.682751	6.320221	6.746833	4.618658	5.57741	0.912975114	low
MB-0459	2.926027397	1	8.965608	6.588605	5.988942	7.129965	4.49255	1.010578866	high
MB-0639	6.197260274	0	9.965929	5.087032	5.355702	9.45844	9.212477	1.407761516	high
MB-0318	13.86575342	0	5.921728	5.455694	6.238671	5.510346	10.428833	0.958970424	low
MB-0417	0.515068493	1	7.892047	5.759297	5.878477	6.180968	4.543863	1.019540319	high
MB-6238	14.21643836	1	7.084034	6.141002	6.014018	6.728247	5.238722	0.896237161	low
MB-6286	4.071232877	1	6.621474	7.433804	5.883337	9.359674	4.620461	0.7137916	low
MB-6328	10.41095891	0	8.312271	4.659081	5.465973	9.144215	4.638597	1.112469171	high
MB-6284	7.260273973	1	9.908766	5.017017	4.90398	5.922058	11.322975	1.770199991	high
MB-6224	2.915068493	1	8.360252	5.143179	4.738199	5.357981	7.950087	1.458629447	high
MB-6297	14.04383562	0	7.428309	6.87672	5.74607	6.675145	9.254789	0.997591598	high
MB-6312	13.37808219	0	8.872253	5.201481	5.781891	7.25099	4.863634	1.152862349	high
MB-6302	10.34520548	1	9.584501	4.468805	5.448132	7.665902	11.203856	1.560207311	high
MB-0342	10.30136986	1	5.931981	8.292552	6.02917	10.107519	7.185479	0.633110725	low
MB-7289	10.41095891	1	6.57082	8.473084	6.457023	6.489917	9.041305	0.735463502	low
MB-7291	0.561643836	1	9.353515	5.967112	5.643597	6.523848	9.834554	1.331270017	high
MB-7243	12.23013699	1	7.333668	7.974574	6.387028	6.238016	6.305097	0.787313666	low
MB-7249	4.821917808	1	6.433155	5.061487	7.045134	5.461348	4.483298	0.811220676	low
MB-7295	16.18082192	0	7.367943	7.582764	6.817243	5.160231	5.51275	0.78010459	low
MB-7296	3.676712329	1	10.338921	4.985638	6.32469	8.754663	4.800853	1.188211131	high
MB-3402	4.112328767	1	8.910732	5.804989	6.463393	5.356257	12.227121	1.263228392	high
MB-5113	14.43561644	0	8.021347	6.7513	5.1745	7.435077	7.485098	1.08273906	high
MB-5450	15.63287671	0	10.87945	5.500737	5.53971	8.032002	9.835946	1.543128512	high
MB-7032	5.873972603	0	6.764505	6.585369	6.545741	4.769175	4.900623	0.825202003	low
MB-7043	7.523287671	0	8.018774	6.753766	5.456716	6.402187	9.03756	1.115079043	high
MB-7028	7.380821918	0	9.183324	5.388622	5.830131	7.502808	6.112502	1.186486637	high
MB-5191	11.68493151	1	7.633507	6.376124	5.336287	7.798204	4.898093	0.977676133	low
MB-5214	8.824657537	1	8.614905	5.635648	4.99014	10.4383	6.076634	1.124122755	high
MB-5614	9.569863011	1	8.12708	7.30649	5.904164	9.316662	5.044249	0.840276516	low
MB-0028	3.005479452	1	8.738298	5.388622	6.225696	4.284517	5.363573	1.176288831	high
MB-0100	0.663013699	1	9.771375	5.371671	6.447263	9.643011	9.976759	1.172000188	high
MB-5572	14.68219178	0	8.619587	5.749141	6.734195	6.086283	5.346136	0.991161181	low
MB-5577	4.553424658	1	8.272433	6.227825	6.269238	5.85246	11.203856	1.135481943	high
MB-5652	8.602739729	1	5.860185	6.398044	6.469771	8.662681	9.407937	0.754872233	low
MB-0537	9.334246578	0	5.136732	7.531666	6.403371	11.584274	7.118781	0.560819774	low
MB-0468	10.65205479	1	8.888063	5.968672	5.728973	6.101303	4.293625	1.1243933	high
MB-0205	11.87397261	0	5.726816	8.649982	6.423998	12.505313	7.149923	0.526939539	low
MB-0200	10.75616439	1	10.523975	4.736687	6.912306	6.691516	6.583451	1.265998477	high

MB-0244	12.30684931	0	5.880539	6.657896	7.012858	9.563667	4.780481	0.599931756	low
MB-5633	2.879452055	1	8.509472	4.951236	6.127331	5.443216	8.332387	1.242188415	high
MB-0278	0.479452055	1	10.21274	5.065274	6.756739	6.630285	10.953984	1.354910479	high
MB-0577	5.375342466	0	8.29525	6.463245	6.759442	5.592785	5.904978	0.932616694	low
MB-0245	13.5369863	0	7.354359	7.662774	6.336234	9.89719	5.485958	0.707377531	low
MB-0534	10.2	0	5.241106	8.130191	5.721742	10.157743	6.821723	0.62013753	low
MB-0369	11.8630137	0	8.608286	5.115487	6.482453	7.598124	4.235713	0.998935106	high
MB-0492	6.668493151	1	8.083356	6.498823	6.105552	4.408028	4.685558	1.010921781	high
MB-0111	10.44657534	0	6.68626	7.153779	6.862522	5.003902	4.824849	0.742499477	low
MB-0634	1.460273973	0	5.911184	8.208273	4.703822	9.956908	6.284625	0.754755119	low
MB-0490	8.380821921	0	6.369919	7.655273	5.433639	4.804461	4.830525	0.853062103	low
MB-4010	0.961643836	1	9.360167	4.288535	6.240294	8.385174	4.815932	1.169340406	high
MB-4529	10.39452055	1	6.800904	5.7653	6.566098	7.967574	5.006041	0.792864202	low
MB-0268	2.342465753	1	8.541346	6.106858	7.121573	5.599314	4.814515	0.909816217	low
MB-0275	15.29589041	0	7.119526	6.713053	6.113494	10.655633	4.618815	0.734165652	low
MB-0045	13.55342466	0	8.443369	7.921584	5.634115	6.584379	5.978726	0.959923867	low
MB-0396	4.98630137	0	10.364661	4.469399	6.838541	4.744911	11.340745	1.528636416	high
MB-0874	1.364383562	1	11.031969	4.496816	6.454041	4.378497	11.040947	1.726578529	high
MB-0179	1.473972602	1	7.740095	7.456353	5.786717	8.62977	7.551148	0.88111981	low
MB-0508	9.397260271	0	8.558491	6.354143	5.99048	9.180333	5.38434	0.942935437	low
MB-0269	1.82739726	0	7.604587	5.489014	5.377638	9.824482	4.525003	0.962207124	low
MB-0901	11.19178082	0	11.011073	4.260259	6.104751	4.409826	8.772209	1.748938174	high
MB-0000	11.54794521	0	5.338409	7.234767	5.926688	12.813336	6.823623	0.596663605	low
MB-0535	16.36712328	0	7.918506	6.661258	6.430765	4.982069	5.603422	0.940234142	low
MB-0170	7.673972603	1	5.620492	7.409025	5.830882	8.417333	5.029348	0.681300244	low
MB-0464	3.230136986	1	8.919953	6.158755	5.596468	8.176647	5.447321	1.084917221	high
MB-0623	9.128767126	0	6.512088	6.868497	5.835776	10.541933	4.992045	0.720207897	low
MB-0241	6.010958904	1	8.958455	4.436706	5.394451	10.325548	7.169912	1.23909446	high
MB-0163	8.063013699	1	9.789828	6.683274	4.979436	4.21988	8.26046	1.502745873	high
MB-0350	3.78630137	1	9.274562	4.564299	6.39958	8.911254	6.325648	1.128914648	high
MB-0046	1.161643835	1	8.919953	6.016847	6.224091	5.948795	12.512123	1.269568998	high
MB-0134	1.063013698	1	7.376175	6.932732	6.580756	5.881302	4.760132	0.814576	low
MB-0470	7.252054795	0	4.673819	6.274979	5.340651	4.564614	4.933512	0.824950823	low
MB-0872	12.49863014	1	7.212651	6.237955	6.56478	7.613177	11.10184	0.924576662	low
MB-0339	2.197260274	1	7.131075	8.074445	5.869458	4.196507	4.700329	0.850403705	low
MB-0264	3.542465753	1	7.607748	8.414883	6.149219	8.562102	6.292825	0.747682339	low
MB-0175	5.947945205	0	7.877534	5.832677	5.978477	7.29337	4.563466	0.962375871	low
MB-0303	4.942465753	0	8.830467	5.900914	5.943912	8.771134	4.68627	1.006806355	high
MB-0172	11.35068493	0	7.341814	6.595108	7.472102	6.254883	4.795033	0.726499692	low
MB-0340	13.53972602	0	8.798418	4.487643	6.698442	4.740345	6.790603	1.207354339	high
MB-0234	7.745205479	1	7.96032	5.246923	6.755379	8.109525	6.008393	0.914963797	low
MB-0305	5.219178082	1	7.336502	7.175953	6.38157	8.710992	10.460986	0.84857666	low
MB-0663	4.550684932	0	7.960964	4.740931	5.711977	6.351776	13.502407	1.385431821	high
MB-0130	12.62191781	0	6.772166	6.518325	6.664816	9.495079	8.582621	0.758454929	low
MB-0343	7.490410959	1	6.961736	8.200007	6.344107	7.524489	4.796527	0.69529318	low
MB-0488	9.282191778	0	7.816981	6.690986	6.638812	10.249992	7.085032	0.783068721	low
MB-0655	9.128767126	0	6.627275	6.41788	6.928732	6.751877	4.557283	0.726068733	low
MB-0235	11.71780822	1	6.527146	6.433434	7.141787	7.054161	8.655652	0.757371309	low
MB-0877	1.18630137	1	10.559262	4.206034	6.588602	5.762795	4.649748	1.36724619	high
MB-0097	8.112328767	0	7.287027	5.272298	6.088658	6.195339	5.357666	0.987121307	low
MB-0897	2.54520548	1	7.111056	5.02636	6.690469	6.734531	5.475732	0.894975352	low
MB-0095	4.090410959	1	8.21604	7.958463	6.540559	4.909069	6.384458	0.879473135	low
MB-0646	1.246575343	1	8.187678	9.083901	6.612951	7.252065	5.312259	0.718505904	low
MB-0552	5.594520548	1	8.52714	5.946352	6.188095	6.598533	9.409065	1.126590613	high
MB-0260	13.95890411	1	6.963861	8.216698	6.891783	10.533038	4.894028	0.583044852	low
MB-0626	2.920547945	1	9.353515	4.414294	6.226498	5.743581	5.046979	1.272677259	high
MB-0211	3.682191781	1	8.516516	5.351786	5.813434	7.837624	5.339632	1.086759091	high
MB-0510	10.55342466	0	8.613968	7.236557	5.963485	9.011366	9.304385	0.977193546	low

MB-0479	10.91232877	0	8.793144	5.49612	5.381477	6.382093	5.913046	1.247334662	high
MB-0263	14.46849315	1	6.798089	6.865935	6.536643	10.495789	4.522413	0.664772586	low
MB-0462	10.91232877	0	8.830467	5.276363	5.722459	6.676103	9.16482	1.293904588	high
MB-0262	14.98082192	1	9.801494	5.332939	5.493791	7.839493	10.146114	1.43727651	high
MB-0418	8.389041099	0	9.373019	6.349779	6.387028	5.79182	11.171254	1.231759364	high
MB-0652	7.942465753	1	7.662468	6.241722	5.203659	4.446539	5.633636	1.147349131	high
MB-0638	8.528767126	1	4.974302	7.084501	5.723897	9.639	4.815773	0.636735451	low
MB-0617	7.64109589	1	4.908607	7.455028	6.423581	9.736149	4.466933	0.550966176	low
MB-7141	11.3890411	0	7.337483	6.682335	5.365889	7.639498	7.955097	0.995808977	high
MB-7030	2.575342465	1	7.728247	7.918963	5.498862	8.799964	10.414943	0.93850137	low
MB-7004	4.147945205	0	6.818807	7.391635	5.393923	9.10334	4.491791	0.786365697	low
MB-7119	10.55068493	0	9.76686	4.270051	5.710902	4.403676	6.939061	1.571937958	high
MB-7263	13.55890411	1	9.810943	6.366837	6.277019	4.175297	4.22613	1.174433358	high
MB-7012	1.630136986	1	9.00389	4.716774	6.084707	4.038487	10.364574	1.464564973	high
MB-0174	6.473972603	0	8.465379	5.108079	6.038959	6.149063	7.968948	1.198685087	high
MB-7112	5.317808219	1	9.516684	8.113351	5.510053	7.11166	4.543568	1.015224973	high
MB-5552	2.852054795	1	10.174472	4.853077	5.953534	6.152527	5.417563	1.376665907	high
MB-5547	8.101369863	0	11.159938	4.295395	7.043179	4.723749	8.737471	1.53299179	high
MB-6189	19.74246575	0	8.988287	6.080969	6.552646	4.597277	4.61134	1.061237256	high
MB-6122	21.38630137	0	8.9715	5.234945	5.355702	6.501943	9.670384	1.409745558	high
MB-6192	3.504109589	1	8.760843	4.643649	6.537528	8.716038	4.51878	1.011658346	high
MB-5167	17.12876712	0	8.303765	6.77483	6.771696	4.733481	4.653207	0.909732604	low
MB-5465	1.545205479	1	9.289596	5.851128	6.595142	5.598929	11.689923	1.255836175	high
MB-5127	15.7369863	1	7.908594	6.319365	7.098531	6.790748	5.515922	0.824995482	low
TCGA-D8-A1Y2	1.18630137	0	7.528107	7.084167	6.010286	6.829628	4.712414	0.856936048	low
TCGA-BH-A1EX	4.131506849	1	6.980165	6.672218	6.667985	8.09609	9.387904	0.815521416	low
TCGA-D8-A1XZ	1.276712329	0	8.915781	6.550754	6.955783	4.892244	3.607072	0.929353257	low
TCGA-A7-A4SD	1.208219178	0	8.801453	4.966226	6.474891	3.390442	7.241174	1.269187223	high
TCGA-BH-A0H9	3.416438356	0	9.215472	6.152515	6.290928	9.429533	10.725941	1.095201097	high
TCGA-E2-A2P5	2.249315068	1	8.91138	4.716793	7.020014	4.95693	5.467868	1.104134476	high
TCGA-A8-A09V	1.252054795	0	7.505184	7.943943	7.400827	5.31616	4.981838	0.695965927	low
TCGA-AR-A252	7.775342466	0	6.925779	6.632546	6.594512	9.756583	5.458757	0.711583935	low
TCGA-AC-A3OD	1.235616438	0	5.896375	6.432011	5.513192	7.395094	3.169764	0.782557216	low
TCGA-BH-A18P	2.523287671	1	8.667233	6.296689	6.938782	8.359931	9.987683	0.955485847	low
TCGA-E9-A22E	3.476712329	0	8.285633	5.612832	5.85937	5.590992	9.577753	1.227916467	high
TCGA-GM-A3NY	3.183561644	0	8.25801	5.180522	6.19152	8.263308	6.428406	1.029245748	high
TCGA-E9-A3HO	3.17260274	0	8.741646	4.716987	6.394211	8.039164	5.660998	1.075439203	high
TCGA-A8-A08R	0.082191781	0	9.604032	6.156404	5.515426	4.496789	4.666956	1.301742314	high
TCGA-AC-A23G	6.15890411	0	7.255433	6.645766	6.005839	7.90874	5.751894	0.853459233	low
TCGA-BH-A1EV	1	1	7.956378	6.232353	7.065265	4.191013	10.473204	1.023000846	high
TCGA-B6-A0IA	22.9890411	0	8.548664	4.427642	6.560547	3.395272	6.880073	1.265126698	high
TCGA-AO-A12A	8.526027397	0	7.682774	7.370237	7.159988	9.222215	6.683134	0.698849633	low
TCGA-B6-A0RI	19.52328767	0	8.826934	6.833358	6.78077	6.00961	5.799751	0.935812545	low
TCGA-EW-A1J2	1.104109589	0	5.804459	6.345277	6.814771	9.947734	5.257576	0.626158316	low
TCGA-GM-A2DL	9.64109589	0	8.805695	4.758437	6.397824	9.97593	8.531768	1.078987831	high
TCGA-D8-A1Y1	0.82739726	1	9.378416	7.25829	7.013944	4.349801	6.758583	0.998150846	high
TCGA-AR-A24L	7.852054795	1	8.421231	6.716125	6.959174	7.16124	3.515226	0.809169221	low
TCGA-D8-A1X8	2.145205479	0	7.572384	8.983824	6.74972	7.115302	3.747257	0.648908862	low
TCGA-BH-A0DH	3.167123288	0	7.742299	7.057173	6.273908	8.181755	5.204181	0.816621253	low
TCGA-A2-A1G1	1.6	0	9.805231	5.519482	5.943529	5.336397	3.794805	1.251392189	high
TCGA-C8-A1HK	1.002739726	0	9.071933	4.908773	6.311563	5.011338	9.348658	1.330949734	high
TCGA-B6-A0I9	0.991780822	1	8.989241	4.400748	5.812845	5.944985	5.734493	1.315532886	high
TCGA-A2-A25C	1.432876712	0	8.446946	7.217465	7.228119	7.988298	8.231074	0.813652508	low
TCGA-GM-A5PV	1.128767123	0	6.967324	7.133732	6.778136	8.737992	7.28779	0.722236469	low
TCGA-BH-A0BM	5.139726027	0	5.245784	6.825134	6.213251	10.208701	6.526201	0.634954409	low
TCGA-W8-A86G	0.950684932	0	6.813968	6.907569	6.41698	7.985664	4.5959	0.734919045	low
TCGA-AO-A0J9	4.419178082	0	8.214611	4.891824	5.65335	11.428214	5.913516	1.005824978	high
TCGA-EW-A1PE	0.876712329	0	7.005287	6.499932	6.71359	6.785397	6.588453	0.8068242	low

TCGA-E2-A15P	1.630136986	0	6.341116	6.514419	6.862812	7.964822	3.704657	0.666586321	low
TCGA-A2-A1FV	1.956164384	0	7.17493	5.6002	7.078848	9.364008	3.490064	0.714334646	low
TCGA-EW-A1PC	0.512328767	0	10.775517	4.456012	7.602594	4.732887	7.094841	1.298628441	high
TCGA-A2-A04Y	3.010958904	0	8.39166	6.059763	6.507155	8.727802	6.208205	0.912908469	low
TCGA-A8-A09R	0.747945205	0	8.397759	6.495954	6.070087	6.565319	9.594553	1.090034222	high
TCGA-AR-A5QN	2.775342466	0	7.441674	5.015472	6.486943	10.593149	4.210907	0.813324377	low
TCGA-HN-A2NL	0.216438356	0	10.387021	4.535737	6.049046	6.007118	8.612454	1.535190355	high
TCGA-AR-A5QP	3.246575342	0	7.796083	7.212954	6.540008	8.200092	7.285043	0.818536092	low
TCGA-AO-A0JL	4.610958904	0	9.734145	4.04669	5.550177	6.258076	4.5359	1.451755629	high
TCGA-A2-A0CV	8.249315068	0	5.222958	7.251831	7.20637	6.514061	10.38093	0.65782984	low
TCGA-A7-A3RF	1.117808219	0	7.361432	4.981082	6.190666	3.570476	3.493143	1.048391054	high
TCGA-OL-A5RZ	1.860273973	0	8.66704	4.869346	5.758952	7.930732	7.276986	1.201516375	high
TCGA-B6-A0IM	10.6109589	1	7.724302	4.916373	6.654411	9.053796	3.967253	0.861004409	low
TCGA-D8-A1J9	1.457534247	0	8.548927	5.481558	5.997557	3.698956	11.512939	1.38857044	high
TCGA-D8-A27T	1.090410959	0	8.014592	6.732419	7.045896	9.170135	7.923596	0.79369023	low
TCGA-AN-A0FK	0.583561644	0	7.528974	5.208934	6.669446	5.560184	7.35396	0.999930507	high
TCGA-D8-A1JF	1.002739726	0	7.511357	5.508998	5.899606	7.36921	5.804903	0.988213319	low
TCGA-GM-A2DF	5.904109589	0	9.202207	4.659807	5.474985	9.464507	10.191311	1.35879433	high
TCGA-A2-A0T5	1.454794521	0	7.735231	7.45875	6.786765	4.770463	9.212726	0.903031131	low
TCGA-E9-A1RF	0.547945205	0	8.471606	5.226981	5.666768	7.631364	4.797495	1.109279897	high
TCGA-AO-A12F	5.046575342	0	10.460184	3.990191	6.060252	10.021791	10.826431	1.481531583	high
TCGA-S3-A6ZG	1.539726027	0	8.949139	6.195159	6.868472	6.375911	5.635016	0.967018958	low
TCGA-BH-A209	10.84657534	1	8.947097	5.79308	5.466689	7.417114	3.360802	1.114598643	high
TCGA-WT-AB44	2.419178082	0	4.980203	4.312863	5.635467	7.564448	5.11441	0.861999558	low
TCGA-BH-A0B1	3.145205479	0	8.155667	5.551378	7.310473	6.58637	5.692362	0.879607215	low
TCGA-AR-A2LJ	7.210958904	0	7.727819	6.667128	5.9467	11.128244	7.562626	0.841859161	low
TCGA-C8-A12P	0.980821918	0	8.351824	4.948102	5.770373	4.740798	10.631634	1.387922373	high
TCGA-C8-A12Q	1.054794521	1	8.359688	5.927812	4.499192	8.394924	11.505777	1.391398939	high
TCGA-E2-A1L6	4.515068493	0	7.552927	7.961663	7.034331	8.501741	7.000931	0.692363938	low
TCGA-E9-A3X8	2.536986301	0	7.388611	5.819472	5.539189	8.882	6.917788	0.978317975	low
TCGA-C8-A1HF	0.909589041	0	10.133667	4.859587	6.787508	4.511738	4.943635	1.273204371	high
TCGA-BH-A0DO	4.504109589	0	5.735026	7.031957	6.709232	8.80019	6.72229	0.643020943	low
TCGA-A8-A0AD	3.169863014	0	7.336804	8.662183	6.63951	7.475989	3.686963	0.651699142	low
TCGA-E2-A15A	1.945205479	0	9.742343	6.080222	6.255556	5.2739	5.639488	1.19114878	high
TCGA-B6-A0IE	5.460273973	1	10.125425	5.415722	6.334618	7.591711	5.821743	1.196156544	high
TCGA-A8-A0A2	1.58630137	0	6.933461	7.886653	6.263199	5.060222	3.825385	0.762872022	low
TCGA-B6-A0RV	14.1260274	0	8.361697	6.472194	6.407224	6.96409	5.701038	0.937382762	low
TCGA-OL-A66I	1.956164384	0	10.200465	5.790183	6.260855	7.522937	11.117144	1.337008367	high
TCGA-C8-A274	1.391780822	0	8.229928	5.922186	6.672126	4.145046	11.738177	1.172025427	high
TCGA-E9-A2JS	2.476712329	1	9.724741	5.313659	6.318983	6.092036	4.901224	1.196639325	high
TCGA-BH-A0B3	3.295890411	0	9.961417	4.60536	5.835898	7.188598	8.721142	1.455637825	high
TCGA-3C-AALI	10.97260274	0	9.438857	5.064037	5.682138	6.823413	8.663033	1.380035237	high
TCGA-AR-A1AH	10.43013699	0	9.920478	4.853184	6.119529	5.948666	3.236682	1.257343936	high
TCGA-EW-A1P3	4.41369863	0	8.473447	6.524043	6.11306	8.94296	7.321733	0.955585369	low
TCGA-A8-A0A7	0.082191781	0	8.278441	5.429865	5.297219	6.868253	10.699183	1.324399828	high
TCGA-OL-A6VO	2.350684932	0	9.696387	4.347976	7.415097	8.275161	11.771648	1.197805067	high
TCGA-OL-A5D6	3.024657534	1	6.881731	6.176309	4.933236	5.583479	11.848877	1.232132319	high
TCGA-A1-A0SO	2.334246575	0	9.173346	4.025801	4.824397	6.496394	11.3909	1.768979175	high
TCGA-E9-A1R4	0.509589041	0	8.174975	6.947427	5.763699	7.167472	7.009069	0.994253697	high
TCGA-BH-A0RX	0.465753425	0	9.526058	5.646774	6.4492	7.388212	5.817853	1.09856925	high
TCGA-E2-A2P6	2.879452055	0	8.648764	6.766101	6.457137	7.629101	10.752388	1.026294414	high
TCGA-AC-A8OR	0.109589041	0	7.216189	7.493166	6.595396	3.688418	4.730733	0.823976762	low
TCGA-AC-A8OP	1.682191781	0	7.94561	6.54167	5.833688	9.143232	3.845626	0.865900816	low
TCGA-AC-A3TN	1.249315068	0	7.03893	5.435169	6.358944	8.362537	5.57751	0.856744191	low
TCGA-EW-A424	1.95890411	0	8.046781	6.131377	6.424959	5.710025	6.700119	0.993228586	low
TCGA-BH-A0DV	5.654794521	0	5.95476	7.198177	6.23149	10.02495	5.55304	0.648412671	low
TCGA-E2-A1IE	6.471232877	0	8.205511	5.626696	6.824583	8.575854	3.717285	0.842076848	low
TCGA-AO-A0J8	1.863013699	0	7.110589	6.967732	6.769478	7.304201	4.601131	0.732774486	low

TCGA-AC-A2BK	6.087671233	0	9.656538	3.954899	6.91282	3.891264	3.388091	1.26243928	high
TCGA-AC-A6IW	1.131506849	0	10.105494	6.641503	5.553095	7.738678	4.348695	1.1667695	high
TCGA-3C-AAAU	11.08767123	0	8.497839	5.677503	6.516111	5.407332	3.245743	0.99053213	low
TCGA-LL-A8F5	1.632876712	0	9.931067	4.129049	4.769793	5.575483	4.793303	1.688572493	high
TCGA-A7-A425	1.224657534	0	8.248123	7.281609	6.234242	10.291213	11.199236	0.9053154	low
TCGA-E9-A295	1.02739726	0	8.305483	5.308838	6.068938	8.49092	8.058327	1.072754459	high
TCGA-EW-A1P8	0.654794521	1	9.03902	7.176304	6.146324	8.799733	4.526056	0.900565384	low
TCGA-AC-A2QH	2.753424658	0	8.322659	4.097104	4.992567	8.671355	3.169764	1.221441272	high
TCGA-GM-A2DC	6.945205479	0	5.010225	5.439751	6.859277	7.526785	5.39117	0.671988533	low
TCGA-AC-A3YI	1.936986301	0	6.270161	6.136486	5.973801	7.335787	6.428177	0.83929898	low
TCGA-E2-A1B5	2.695890411	0	7.149862	5.593916	5.747865	10.272625	9.314466	0.952468876	low
TCGA-A8-A07W	0.832876712	0	9.657483	6.434024	6.207274	8.129285	10.445981	1.174068972	high
TCGA-A2-A0CZ	4.42739726	0	6.106771	5.945467	5.303705	9.760715	6.172444	0.84515056	low
TCGA-AN-A0FS	0.575342466	0	6.804019	7.165988	6.253684	9.524743	4.650354	0.700573328	low
TCGA-BH-A1FN	6.005479452	1	9.459591	4.596874	7.067129	4.022582	3.529081	1.152073858	high
TCGA-B6-A0RS	8.391780822	1	8.936014	4.476572	6.397905	7.886679	9.162622	1.214295214	high
TCGA-A7-A3J1	0.939726027	0	7.106989	8.755895	6.59124	9.337692	4.434305	0.609145101	low
TCGA-A2-A04U	7.271232877	0	10.666998	3.887445	6.158327	4.20589	7.109895	1.675463374	high
TCGA-E9-A1RI	3.969863014	0	6.645577	7.075493	7.233132	5.191507	5.273991	0.709035284	low
TCGA-EW-A1P5	1.926027397	0	7.795354	7.121887	6.75906	5.276628	5.24533	0.840915622	low
TCGA-C8-A278	0.81369863	0	8.847921	5.093932	6.555229	4.678586	9.861765	1.269202604	high
TCGA-D8-A4Z1	1.805479452	0	6.478955	8.243902	6.732166	7.727866	5.418157	0.630861386	low
TCGA-A8-A07S	0.665753425	0	8.254137	6.921209	6.540592	7.020762	8.920669	0.944377258	low
TCGA-BH-A0W7	3.734246575	0	8.78105	6.146813	6.698671	8.399963	8.053814	0.96615167	low
TCGA-E9-A1N9	3.016438356	0	9.142644	5.780733	6.441044	6.015882	5.030485	1.078512625	high
TCGA-BH-A0BZ	6.178082192	0	9.771324	5.299976	6.119527	7.043176	10.940208	1.375426756	high
TCGA-D8-A1X5	1.547945205	0	7.66483	4.641003	6.504405	5.094049	11.553926	1.210571143	high
TCGA-A8-A06X	2.583561644	1	8.832291	4.638337	6.500637	6.461499	8.035001	1.195977667	high
TCGA-E2-A109	3.882191781	0	9.914551	4.915332	5.998935	5.460772	7.215703	1.415187131	high
TCGA-E2-A1IJ	2.369863014	0	6.742654	8.127097	6.417596	7.04148	6.554121	0.71657993	low
TCGA-BH-A202	2.178082192	0	8.812131	5.037596	7.186527	4.914479	4.39293	1.018405608	high
TCGA-A2-A0CY	4.583561644	0	8.610217	3.896017	4.339497	3.69527	11.653254	2.000804107	high
TCGA-OL-A5RX	2.405479452	0	6.022175	6.184421	6.649346	8.993066	8.314083	0.73328123	low
TCGA-BH-A1FG	1.580821918	1	7.090477	5.831449	6.539755	6.924549	4.377856	0.830963494	low
TCGA-D8-A27P	0.134246575	0	6.846123	7.721885	7.033686	5.516819	5.011432	0.695494785	low
TCGA-A2-A0CS	6.432876712	1	7.54855	6.118279	7.089653	4.207843	5.201033	0.876715172	low
TCGA-A2-A04W	8.498630137	0	8.473993	4.293163	5.519366	4.801182	10.691004	1.529408582	high
TCGA-A2-A0D1	2.879452055	0	8.635972	4.230924	6.039564	4.45566	7.393088	1.361482553	high
TCGA-E9-A1RE	3.887671233	0	8.820009	5.093914	6.207333	8.312273	3.226053	1.013167127	high
TCGA-E2-A1LI	8.550684932	0	9.950033	6.246358	6.329162	5.065765	11.630257	1.369982694	high
TCGA-OL-A6VR	3.342465753	0	6.919242	5.064321	6.802265	6.781646	6.502441	0.881286875	low
TCGA-S3-AA12	1.57260274	0	7.920996	5.595405	6.352504	3.619555	3.273222	1.024628781	high
TCGA-D8-A27R	0.84109589	0	9.543677	5.18255	5.665473	7.999475	5.727161	1.24555976	high
TCGA-A7-A5ZX	0.920547945	0	5.975579	6.730752	6.743033	6.54504	6.901399	0.725558891	low
TCGA-A2-A4S1	2.246575342	0	6.960846	7.748241	4.872732	6.832161	3.299239	0.876047844	low
TCGA-PE-A5DE	7.246575342	0	8.268173	6.904513	6.638018	7.899875	7.277939	0.873825089	low
TCGA-A8-A099	0.832876712	0	6.855935	7.391137	6.374901	4.864303	4.699255	0.795148566	low
TCGA-BH-A0HW	4.276712329	0	7.898475	4.919422	6.754337	4.545759	3.945903	1.002256828	high
TCGA-A8-A06O	1.084931507	0	8.416589	5.009388	5.664471	9.625642	3.586613	1.021940203	high
TCGA-B6-A0IN	7.049315068	1	7.475316	4.964729	5.275475	9.21086	8.048575	1.11001787	high
TCGA-E2-A1B4	2.750684932	1	6.428643	6.465507	7.311999	4.397384	5.814424	0.748211313	low
TCGA-A1-A0SQ	1.517808219	0	8.55513	6.193007	6.528619	6.627888	6.538497	0.988727966	low
TCGA-BH-A0E1	1.306849315	0	9.212566	4.955388	7.079338	6.738643	8.378847	1.114287357	high
TCGA-LD-A9QF	0.884931507	0	7.539819	5.657562	5.386023	7.60153	10.449094	1.161335524	high
TCGA-B6-A0WT	15.72328767	0	7.599378	6.555545	7.034516	5.981228	5.119513	0.807746035	low
TCGA-AR-A1AY	2.810958904	0	10.278355	5.251805	6.76141	7.009839	10.142625	1.302045131	high
TCGA-BH-A0B0	6.78630137	0	7.122345	6.819131	6.323542	10.61698	7.999492	0.76458274	low
TCGA-B6-A0RO	13.50410959	0	6.979514	7.278269	5.895026	7.152876	5.72795	0.823889003	low

TCGA-D8-A1JS	1.016438356	0	6.723582	6.438009	6.148797	5.910708	4.976328	0.847883338	low
TCGA-D8-A27L	1.367123288	0	7.126318	7.485724	7.271005	8.287329	6.337391	0.661461194	low
TCGA-LL-A6FR	1.339726027	0	8.405009	4.041085	5.951168	3.526282	12.45249	1.582960029	high
TCGA-A2-A0CQ	7.383561644	0	7.978835	7.9034	6.681171	6.787773	6.85766	0.803657347	low
TCGA-AR-A24R	9.397260274	0	9.861649	5.340979	6.800214	8.997236	5.757041	1.04671027	high
TCGA-A1-A0SG	1.189041096	0	7.163652	6.307955	6.50122	7.420521	6.55507	0.838189004	low
TCGA-BH-A1FL	4.583561644	1	7.745074	6.974926	7.271217	8.111713	4.917038	0.711308778	low
TCGA-A8-A0A6	1.753424658	0	6.434382	6.92054	6.415638	7.533735	8.039831	0.777150733	low
TCGA-AR-A0U3	11.17808219	0	9.27331	5.628231	4.665223	6.980166	6.846842	1.43237784	high
TCGA-BH-A18N	1.282191781	1	7.159957	7.889501	6.881917	5.017679	3.457048	0.709523999	low
TCGA-BH-A0BA	3.101369863	0	8.306887	6.304631	6.45129	9.396124	10.092868	0.95667757	low
TCGA-E2-A10C	3.342465753	0	9.108889	4.914847	6.21213	7.360069	8.865487	1.238509824	high
TCGA-AR-A256	7.819178082	1	10.234693	5.814106	6.726835	7.963562	4.223243	1.056469948	high
TCGA-D8-A1XO	4.608219178	0	6.726041	9.19139	7.163111	5.499774	4.245864	0.592569324	low
TCGA-E2-A15M	0.920547945	1	8.962835	7.185393	6.644333	10.768841	8.825157	0.860083587	low
TCGA-A8-A07I	1.167123288	0	8.900147	5.619533	6.642476	7.986909	4.520377	0.959962068	low
TCGA-A7-A26J	1.717808219	0	7.7227	6.040701	6.708849	5.543684	7.81609	0.960653803	low
TCGA-A2-A04R	10.16164384	0	9.481953	4.172112	6.524711	6.983857	4.964832	1.205740039	high
TCGA-A2-A3XZ	4.197260274	0	8.254742	5.917318	5.865942	7.200936	11.171227	1.174175236	high
TCGA-OL-A66P	1.17260274	0	7.942369	4.526837	4.397893	7.410746	11.540672	1.56217899	high
TCGA-GM-A2DO	7.112328767	0	8.838645	4.508634	6.355784	4.152727	4.259619	1.222608833	high
TCGA-OL-A5RY	2.060273973	0	8.628483	5.476089	4.918949	9.313328	10.723195	1.327988649	high
TCGA-E2-A155	1.753424658	0	8.569905	4.791131	6.726732	7.598223	3.437251	0.96824431	low
TCGA-BH-A0DK	1.15890411	0	8.559165	5.790863	4.893399	9.016687	6.730182	1.191714359	high
TCGA-BH-A8G0	1.81369863	0	5.720998	6.936753	6.460912	8.158453	6.347846	0.678573084	low
TCGA-E2-A150	5.301369863	0	9.152464	7.237741	6.586747	5.985787	8.600986	1.02592254	high
TCGA-D8-A27V	1.043835616	0	7.059388	7.181563	7.10582	6.6136	8.868007	0.771184729	low
TCGA-A8-A084	1.254794521	0	8.537531	4.956218	6.349288	5.431191	11.340232	1.292628747	high
TCGA-BH-A0H6	2.046575342	0	7.099104	6.460607	6.769302	8.942211	5.20383	0.73080997	low
TCGA-LL-A442	2.435616438	0	7.105628	8.37087	7.130767	4.509969	6.610035	0.717410002	low
TCGA-AR-A2LL	5.512328767	0	7.982939	4.890142	6.713893	10.041825	3.964926	0.848759899	low
TCGA-B6-A0IB	10.79726027	1	8.995733	4.589553	6.263894	8.527361	10.958451	1.258066811	high
TCGA-EW-A1IY	0.706849315	0	8.558709	6.303449	7.462611	6.465816	8.221003	0.898100369	low
TCGA-BH-A0E0	0.367123288	0	9.494912	4.33894	6.438076	4.721521	9.981235	1.457738951	high
TCGA-E2-A1B1	7.268493151	0	7.518135	7.078001	6.778054	10.241929	7.478458	0.730771932	low
TCGA-A8-A086	1.084931507	0	8.288197	6.443835	6.477333	5.73677	4.91817	0.945149074	low
TCGA-A2-A0YM	2.643835616	0	9.972614	5.194069	6.00917	7.038423	10.870357	1.434005497	high
TCGA-E2-A3DX	3.630136986	0	6.333806	5.35754	6.136774	8.58246	8.70792	0.885598607	low
TCGA-BH-A0W5	3.528767123	0	7.264694	6.962904	6.541462	8.35588	6.009038	0.766022907	low
TCGA-A2-A0T2	0.698630137	1	8.582996	4.884811	6.821041	4.308252	8.073873	1.177317633	high
TCGA-AO-A0J5	2.169863014	1	7.981829	7.257555	5.393666	9.172512	3.74643	0.871941644	low
TCGA-A2-A0T4	1.709589041	0	8.674269	7.140554	6.40123	10.628172	5.385185	0.807079855	low
TCGA-E2-A1LH	8.895890411	0	9.691907	4.578704	4.793932	8.922093	11.991787	1.673598367	high
TCGA-AR-A0TY	4.654794521	1	9.379592	4.47461	5.772616	6.579044	5.56761	1.33237839	high
TCGA-BH-A0HK	0.487671233	0	6.953403	6.463577	5.916477	10.153145	5.257138	0.781298281	low
TCGA-C8-A26Z	1.287671233	0	8.362953	5.628355	6.868539	5.087621	9.662525	1.092071641	high
TCGA-AO-A0JD	6	0	9.747558	5.223719	6.625277	7.652804	5.185322	1.10524766	high
TCGA-D8-A1Y3	1.178082192	0	9.581837	4.82206	6.166951	3.88	3.222023	1.297619035	high
TCGA-AR-A1AO	7.17260274	0	8.060751	6.888182	5.962888	8.987933	10.125835	0.970470762	low
TCGA-BH-A18U	4.282191781	1	9.455717	4.745356	6.21211	6.518539	3.366094	1.177582678	high
TCGA-D8-A27H	1.087671233	0	9.428668	7.111447	5.20047	6.36521	3.632483	1.140820922	high
TCGA-BH-A1FJ	5.279452055	1	8.862768	6.245535	7.449357	4.9587	7.416836	0.960819178	low
TCGA-AR-A2LH	1.687671233	1	7.988417	7.278466	5.906254	7.897135	10.319825	0.981732394	low
TCGA-D8-A1XT	1.38630137	0	8.428633	5.545091	6.080483	5.608924	6.612805	1.133397444	high
TCGA-C8-A26V	1.687671233	0	8.389354	6.155434	6.317311	3.390195	3.989214	1.056632485	high
TCGA-D8-A1J8	1.180821918	0	9.649667	5.080699	7.314566	3.98486	5.614667	1.146035033	high
TCGA-E2-A1LS	4.394520548	0	7.98489	4.970267	5.630209	5.005038	5.203142	1.195201634	high
TCGA-AR-A0U0	5.446575342	0	8.909793	5.398037	7.266659	3.470515	4.750324	1.045676383	high

TCGA-A1-A0SK	2.649315068	1	10.581049	4.196302	6.772312	5.376492	8.840652	1.488623693	high
TCGA-A8-A082	1.504109589	0	9.040514	8.794376	6.807913	5.896675	4.289955	0.793260023	low
TCGA-E9-A22B	3.197260274	0	8.409419	5.586033	6.001516	9.035431	4.839999	0.977356715	low
TCGA-A8-A0A1	1	0	7.656268	7.115584	6.376124	9.852264	5.211794	0.751889422	low
TCGA-C8-A26X	1.030136986	0	8.0347	4.669732	5.142392	5.68712	11.963666	1.500798565	high
TCGA-BH-AB28	0.78630137	0	7.384517	7.169369	6.065584	8.964897	8.989902	0.855204239	low
TCGA-A8-A09M	2.756164384	0	9.106326	4.845038	6.031659	5.055484	4.156598	1.238759888	high
TCGA-A2-A0YG	1.824657534	0	8.949393	5.694672	5.918769	6.308024	9.104976	1.246132389	high
TCGA-BH-A0DE	6.498630137	0	6.97643	7.758545	6.087959	10.175364	3.818712	0.6688519	low
TCGA-D8-A1JH	1.167123288	0	6.49592	8.179947	6.88367	8.206714	3.916273	0.591266467	low
TCGA-A2-A0CL	8.260273973	0	9.201693	6.076034	5.930836	8.977355	4.562723	1.021455245	high
TCGA-BH-A1F6	8.123287671	1	9.489752	5.375865	7.041116	7.177175	9.32997	1.121735518	high
TCGA-A7-A0CE	2.942465753	0	9.030761	4.28903	6.665092	8.401935	4.010015	1.046995732	high
TCGA-AR-A24S	8.153424658	0	8.4376	5.678172	6.290471	10.255162	4.24119	0.884990354	low
TCGA-A8-A07R	0.747945205	0	9.75254	4.184454	6.158488	3.433982	8.483399	1.586972411	high
TCGA-BH-A0H5	4.438356164	0	7.120305	5.779382	6.450388	8.513899	5.281219	0.820364167	low
TCGA-A8-A076	4.498630137	0	9.453043	5.152997	5.82073	7.971123	10.695619	1.356764957	high
TCGA-Z7-A8R5	9.005479452	0	6.366134	6.293769	5.947348	8.247637	9.139919	0.866703301	low
TCGA-A2-A259	4.37260274	0	6.583196	6.357707	6.701432	6.913661	3.706248	0.731680484	low
TCGA-BH-A0AW	1.704109589	0	9.282726	5.313011	5.325363	7.385848	11.37657	1.463806919	high
TCGA-A8-A093	1.495890411	0	7.768464	5.891914	6.302908	8.293835	4.365861	0.872051221	low
TCGA-B6-A1KN	11.59726027	0	8.936916	4.995037	6.786175	7.198808	4.359204	1.013314147	high
TCGA-BH-A0BC	2.668493151	0	8.114166	7.536729	6.014312	9.053593	6.187689	0.840431574	low
TCGA-E2-A106	6.961643836	0	7.627336	5.34071	6.196469	5.911687	10.397639	1.131270208	high
TCGA-BH-A0EB	2.04109589	0	6.975351	8.692328	6.435715	5.759673	8.53936	0.763911261	low
TCGA-WT-AB41	4.41369863	0	7.58443	4.869963	5.796852	9.170487	4.005918	0.959106735	low
TCGA-E9-A6HE	2.320547945	0	8.730716	4.441753	6.194504	10.444296	5.871516	1.046939999	high
TCGA-AC-A6IV	1.556164384	0	7.731649	6.762336	6.007653	10.675828	9.770381	0.884705564	low
TCGA-AC-A4ZE	2.438356164	0	7.798075	6.179367	6.24444	9.855908	9.459122	0.919222826	low
TCGA-AR-A1AM	8.194520548	0	6.380677	7.000696	6.689953	9.163792	6.268912	0.672613743	low
TCGA-AC-A23E	1.912328767	0	6.906572	8.11807	7.135625	4.656152	5.497705	0.695867408	low
TCGA-AN-A0AK	0.61369863	0	9.850434	4.529615	6.069378	6.271788	4.199192	1.303996549	high
TCGA-E9-A247	3.249315068	0	9.247026	4.987705	6.298367	8.433087	5.258422	1.096148396	high
TCGA-GM-A2DD	6.252054795	0	8.852927	4.893721	5.578686	8.096776	10.625196	1.343910242	high
TCGA-AO-A1KR	6.884931507	0	10.01826	3.811996	5.584381	7.302686	6.346377	1.521784585	high
TCGA-AN-A0XW	0.465753425	0	9.066092	5.976633	5.93856	3.762791	3.973876	1.190972549	high
TCGA-E2-A1IG	5.863013699	0	7.716336	6.786231	6.675103	4.829387	4.741328	0.869612199	low
TCGA-C8-A27A	2.046575342	0	9.760374	6.392472	7.263255	3.683054	3.376658	1.012134408	high
TCGA-AO-A03T	5.819178082	0	9.533499	4.914531	5.625437	8.027243	5.736755	1.276652933	high
TCGA-B6-A0RQ	11.69041096	1	5.429455	7.341179	6.38409	8.719382	5.473302	0.621996839	low
TCGA-D8-A27F	1.336986301	0	9.76424	5.485481	5.329035	6.718886	5.291373	1.346528933	high
TCGA-BH-A1F2	2.62739726	1	8.965548	5.846555	6.25092	10.192955	5.53804	0.953927223	low
TCGA-BH-A0AZ	5.257534247	0	7.048878	7.391918	6.008823	8.836513	6.09284	0.77140809	low
TCGA-C8-A1HM	1.02739726	0	10.404936	4.861876	6.197604	5.169383	9.850064	1.553084431	high
TCGA-BH-A6R8	0.802739726	0	8.172116	4.808545	6.943006	3.954432	6.054722	1.081351826	high
TCGA-BH-A0E6	0.802739726	0	8.206739	4.432493	5.123056	9.312501	12.085092	1.385186751	high
TCGA-A2-A3KC	3.019178082	0	8.622953	7.718079	6.428832	8.708988	7.359787	0.85292085	low
TCGA-BH-A0EI	5.276712329	0	7.587655	7.255064	6.808673	5.074753	3.764135	0.788586257	low
TCGA-AO-A0JE	6.397260274	0	8.455158	6.538913	6.04333	5.215898	6.697521	1.073835579	high
TCGA-HN-A2OB	5.205479452	1	5.805473	6.51717	6.350024	9.677164	6.298685	0.681672276	low
TCGA-E2-A1BD	3.104109589	0	7.962651	6.042548	7.145064	6.124982	7.174195	0.893584319	low
TCGA-E9-A1R3	0.21369863	0	4.131171	5.584703	6.748946	5.007811	8.062835	0.716445352	low
TCGA-BH-A0C7	7.580821918	0	9.35087	5.392811	6.332949	8.221018	6.131073	1.096949858	high
TCGA-B6-A0WW	1.528767123	1	8.436413	4.977605	5.636133	4.988879	5.301377	1.250097483	high
TCGA-A8-A07L	2.671232877	0	9.684201	5.630338	7.426801	5.056576	4.865023	1.029110847	high
TCGA-E9-A226	2.871232877	1	9.001284	6.776596	6.573667	6.219366	8.192907	1.031937769	high
TCGA-AR-A2LO	3.282191781	0	8.635975	7.238706	6.543114	8.973284	6.557743	0.848812555	low
TCGA-GM-A2DN	8.468493151	0	7.832041	5.906186	6.596313	5.572605	6.294914	0.96186342	low

TCGA-E9-A1NE	2.980821918	0	8.571845	6.490144	6.23773	7.849265	6.032563	0.957133948	low
TCGA-D8-A1JJ	1.673972603	0	8.519452	5.694162	6.016175	4.609307	10.734897	1.29489984	high
TCGA-BH-A18J	1.676712329	1	8.384824	6.462809	6.358348	4.324045	10.10089	1.142195835	high
TCGA-S3-AA14	1.449315068	0	7.839878	6.156184	6.598942	7.451872	6.172986	0.884198357	low
TCGA-A2-A0YD	2.106849315	0	7.438594	7.886993	6.75391	9.663243	9.36755	0.727426325	low
TCGA-B6-A0IP	10.75616438	1	7.67344	6.180763	6.581379	7.298745	5.204788	0.856024395	low
TCGA-S3-AA10	1.605479452	0	10.319687	4.965592	5.441199	4.233231	9.000835	1.720132675	high
TCGA-E9-A1NI	0.821917808	0	8.812152	5.490561	6.515178	5.284894	9.067252	1.18735319	high
TCGA-B6-A0I8	2.052054795	1	7.408541	6.436786	5.284563	5.740139	12.308104	1.215501322	high
TCGA-BH-A1FM	3.802739726	1	8.867253	4.628935	5.777637	3.559341	4.001877	1.336125187	high
TCGA-BH-A0HX	2.271232877	0	8.697214	5.926381	6.456504	7.983577	4.71966	0.94810061	low
TCGA-BH-A18G	0.408219178	0	8.89452	6.222914	5.847243	3.431691	4.382415	1.188105809	high
TCGA-E2-A1AZ	6.380821918	0	9.683803	5.800194	7.50029	6.819961	4.864877	0.947943375	low
TCGA-A2-A04X	4.619178082	0	9.041213	4.530511	6.475257	4.819846	7.167539	1.278764215	high
TCGA-A7-A13H	2.463013699	0	6.653922	6.653494	6.445009	7.942942	4.990747	0.742779285	low
TCGA-LL-A5YP	1.232876712	0	8.854832	5.264767	5.673937	7.10708	5.96789	1.198134773	high
TCGA-BH-A0DG	5.591780822	0	8.089992	6.284323	5.804635	9.514283	7.490381	0.965019977	low
TCGA-AQ-A54N	0.21369863	0	9.333742	3.827458	6.662436	3.452764	7.103831	1.41348093	high
TCGA-E2-A1L9	1.638356164	0	7.856814	6.086458	7.266712	9.285036	5.825722	0.756556775	low
TCGA-A2-A0EQ	6.646575342	0	9.256341	5.16359	4.832728	6.413944	4.484239	1.398130889	high
TCGA-AO-A0JA	1.794520548	0	8.734732	6.649651	5.790154	6.705214	7.040717	1.087170802	high
TCGA-AO-A0J7	1.693150685	0	8.799963	4.914545	6.65	5.1066	3.882288	1.087802521	high
TCGA-AO-A0JM	5.983561644	0	9.378921	4.717146	6.415101	6.619774	7.016234	1.232717645	high
TCGA-UL-AAZ6	1.419178082	0	8.455511	3.980463	6.097014	3.60638	6.951445	1.378000864	high
TCGA-OL-A66O	1.446575342	0	9.205827	4.442656	6.52076	5.698849	5.707473	1.221078	high
TCGA-D8-A147	1.6	0	9.710677	5.672785	6.849721	3.598454	9.812594	1.310203043	high
TCGA-E2-A1B6	2.375342466	0	9.559779	5.357556	6.083454	7.594605	9.335037	1.276581871	high
TCGA-E9-A1QZ	2.068493151	0	8.5895	6.925409	6.322094	11.616328	4.475521	0.780148386	low
TCGA-AO-A0JF	5.424657534	0	6.950258	7.723705	6.652983	10.520884	5.434914	0.633569816	low
TCGA-D8-A1JG	4.416438356	0	9.205075	5.343378	4.993036	3.983195	7.419513	1.554659521	high
TCGA-BH-A0C0	3.479452055	0	9.068984	5.370635	5.772828	6.842368	4.952529	1.179436165	high
TCGA-E2-A15C	1.901369863	0	6.89416	7.354896	6.767587	5.873789	5.381369	0.743691184	low
TCGA-D8-A73W	1.054794521	1	6.944594	5.841031	5.993789	8.183828	11.252539	0.991317005	low
TCGA-GM-A3XL	5.775342466	0	10.314643	4.306883	6.628335	4.68771	10.773451	1.569457824	high
TCGA-A2-A0YK	1.610958904	0	6.962128	7.698232	6.101622	10.007065	9.457163	0.765586965	low
TCGA-E2-A15G	1.517808219	0	6.267458	8.437524	7.07059	6.873176	8.704114	0.643648997	low
TCGA-AR-A24Z	8.221917808	0	8.628907	5.321078	6.369538	7.494359	4.3573	1.007187707	high
TCGA-BH-A0DS	0.21369863	0	6.486788	5.640669	6.226177	9.664605	7.391965	0.81294213	low
TCGA-BH-A204	6.942465753	1	8.490412	6.569491	6.930151	5.300276	3.300461	0.8757666	low
TCGA-BH-A0DI	2.498630137	0	6.678962	6.552091	5.703346	9.071973	5.639628	0.814298063	low
TCGA-AO-A12C	6.498630137	0	6.990669	5.847461	6.538028	5.594342	6.166124	0.895014865	low
TCGA-A2-A04P	1.501369863	1	8.8083	4.841242	5.627853	6.261636	9.158378	1.37177093	high
TCGA-BH-A0AY	2.128767123	0	8.006162	4.704697	6.113098	9.982294	3.400193	0.929013288	low
TCGA-BH-A18L	2.221917808	1	9.678819	5.799154	6.113169	8.251388	6.960914	1.152088012	high
TCGA-E9-A1NG	2.153424658	1	7.040608	7.975742	6.815636	5.414218	9.053734	0.78820161	low
TCGA-B6-A0WX	1.750684932	1	8.752248	6.832734	6.631082	6.432092	6.235642	0.945018479	low
TCGA-A2-A3XY	2.994520548	1	9.136337	5.322586	5.991562	10.652528	11.750011	1.187903441	high
TCGA-EW-A1PF	1.202739726	0	7.287788	7.103946	6.258861	8.300287	6.383991	0.798491166	low
TCGA-EW-A1P0	3.42739726	0	8.094129	5.334728	6.736732	7.551638	5.349309	0.926307791	low
TCGA-A7-A0CG	2.857534247	0	7.229894	7.486724	6.379554	9.647856	9.14327	0.771595071	low
TCGA-D8-A1JL	1.673972603	0	7.992723	6.235212	6.75479	4.330773	7.347404	0.994100631	low
TCGA-BH-A0BW	6.495890411	0	8.718568	5.43402	6.347652	4.544928	4.900348	1.128160716	high
TCGA-A2-A0CM	2.065753425	1	10.181677	5.337451	6.475561	5.968422	11.030803	1.409283388	high
TCGA-E2-A153	1.936986301	0	7.071128	7.483533	6.482044	5.05917	6.204979	0.816301802	low
TCGA-D8-A146	1.761643836	0	7.246761	7.303606	6.901632	9.589319	6.413594	0.685811141	low
TCGA-A2-A4S3	1.824657534	0	9.485908	4.815099	5.999914	6.318503	4.211271	1.242018819	high
TCGA-BH-A18M	6.046575342	1	7.308876	7.136435	6.72474	9.553317	5.280039	0.699148201	low
TCGA-AR-A254	7.136986301	0	8.514661	5.275965	6.721989	8.455042	5.529993	0.946183275	low

TCGA-B6-A0I6	2.715068493	1	9.729804	6.471268	6.685663	4.528379	11.660659	1.277019608	high
TCGA-D8-A1JA	1.375342466	0	7.638973	5.913537	8.037119	3.535376	5.627878	0.811842853	low
TCGA-EW-A2FS	4.394520548	0	8.482468	4.960609	6.22645	6.940805	3.609659	1.043376973	high
TCGA-A8-A06P	1.084931507	0	6.629011	6.92718	6.417127	9.129923	6.231475	0.720182898	low
TCGA-AR-A1AI	9.030136986	0	9.290256	5.295996	5.469737	3.676334	5.658188	1.427830495	high
TCGA-A2-A4S0	1.934246575	0	5.935741	7.755062	6.742631	6.931184	3.282884	0.607264691	low
TCGA-BH-A0B6	6.802739726	0	7.069827	6.457733	5.643337	5.9564	5.845377	0.95722718	low
TCGA-BH-A0HO	0.208219178	0	7.190431	7.923664	7.067096	7.344784	4.049392	0.648641846	low
TCGA-GM-A5PX	1.509589041	0	5.09757	5.678188	6.757334	10.273679	5.57122	0.61853319	low
TCGA-BH-A6R9	0.438356164	0	7.551076	6.485837	5.593528	5.783496	5.279623	1.000429693	high
TCGA-D8-A1XS	1.35890411	0	8.749088	6.342477	6.573819	4.974789	6.294817	1.039654699	high
TCGA-AN-A0FN	0.597260274	0	7.109713	7.67708	6.354427	9.653173	4.617903	0.680409877	low
TCGA-A2-A25E	8.778082192	0	9.355799	4.953078	6.336632	7.325922	3.560876	1.102762714	high
TCGA-C8-A12O	1.054794521	0	8.349158	6.483801	5.95145	4.444034	3.434057	1.030171571	high
TCGA-AR-A24O	9.882191781	0	7.792304	8.263824	6.66739	10.364034	8.045055	0.701584897	low
TCGA-BH-A0BD	1.517808219	0	8.88087	6.035131	6.896041	9.45716	7.872159	0.920101915	low
TCGA-AO-A12B	8.189041096	0	7.795763	6.715798	6.365423	6.429235	3.821683	0.854632986	low
TCGA-BH-A0W3	1.994520548	0	8.296597	8.268354	6.67112	6.250463	9.423529	0.870626182	low
TCGA-E2-A14O	3.723287671	0	8.739967	4.115533	5.999763	7.014469	9.384189	1.34081308	high
TCGA-A8-A08A	0.082191781	0	7.320115	8.293338	6.455818	7.526891	6.159747	0.725486087	low
TCGA-LL-A7T0	1.030136986	0	9.62214	5.12877	6.301235	6.141576	6.693025	1.252918291	high
TCGA-A2-A0SV	2.260273973	1	9.247587	4.117692	5.972706	5.635926	4.073044	1.31130235	high
TCGA-E2-A15I	4.635616438	0	5.42141	7.196837	7.105771	6.438726	9.922266	0.677618502	low
TCGA-A2-A4S2	1.761643836	0	7.051935	6.249365	6.827654	7.965946	5.47834	0.762350106	low
TCGA-A8-A06Y	2.167123288	0	6.908982	5.061422	6.869125	4.572666	3.568336	0.878295432	low
TCGA-AC-A6NO	0.139726027	0	8.28547	6.590129	7.085775	6.326647	8.941149	0.921375971	low
TCGA-AO-A0JC	4.238356164	0	7.745995	5.69091	6.134914	8.865375	4.054495	0.881647624	low
TCGA-S3-AA0Z	1.723287671	0	9.871192	5.546912	7.032508	6.751663	10.053483	1.185599777	high
TCGA-AR-A24T	8.77260274	0	6.248761	8.452369	6.63253	7.58546	5.158148	0.615016708	low
TCGA-GI-A2C9	9.156164384	0	8.957206	6.406674	5.616591	3.949129	8.463488	1.312893964	high
TCGA-A2-A3Y0	4.235616438	0	10.084067	4.430934	5.564647	3.8725	13.091528	1.912755013	high
TCGA-BH-A0BT	6.479452055	0	7.696749	5.395047	7.000553	6.387291	5.30978	0.887923776	low
TCGA-BH-A1EO	7.665753425	1	7.056628	7.256524	6.523758	8.51761	3.169764	0.686127921	low
TCGA-AC-A62X	1.142465753	0	9.156136	4.036103	7.220481	4.537399	3.628614	1.126549184	high
TCGA-E9-A227	2.671232877	0	7.547871	6.870564	6.476051	8.122577	4.076469	0.771786377	low
TCGA-B6-A0IO	13.81369863	0	8.216844	5.204675	3.455208	5.176344	5.236863	1.622620271	high
TCGA-BH-A1FE	6.22739726	1	7.754631	6.19088	6.522714	7.71964	3.88055	0.831741648	low
TCGA-A7-A26G	1.978082192	0	8.509283	7.502544	5.626666	7.684865	6.132664	0.966354222	low
TCGA-AR-A0TX	5.402739726	0	8.578307	6.514828	6.329215	9.400013	5.616908	0.887809459	low
TCGA-AQ-A54O	2.742465753	0	9.093328	5.662524	6.258425	7.630425	4.4163	1.038769895	high
TCGA-E2-A1IO	5.082191781	0	7.621181	7.520339	6.106817	7.409172	5.978093	0.832585106	low
TCGA-E9-A1NH	1.578082192	0	6.682369	7.282645	6.708384	7.966444	9.076288	0.74956885	low
TCGA-A2-A4RX	2.032876712	0	8.350415	7.269483	6.263598	8.307019	10.741511	0.963459366	low
TCGA-5L-AAT0	4.046575342	0	6.931133	7.401411	6.211602	6.0055	5.514305	0.803166446	low
TCGA-BH-A0EA	2.715068493	1	5.012461	6.799397	6.66519	10.604266	6.478476	0.575441856	low
TCGA-A2-A25B	3.536986301	0	9.319707	5.631501	5.512768	4.315663	6.910706	1.396849181	high
TCGA-AR-A24U	8.569863014	0	8.104906	5.331416	6.312119	10.430943	10.639726	1.009073252	high
TCGA-A2-A0EW	5.161643836	1	6.506301	6.704353	6.590003	10.358729	6.537488	0.683087084	low
TCGA-C8-A3M8	1.079452055	0	8.058248	5.376417	6.406242	8.819344	4.661713	0.90986982	low
TCGA-A8-A07Z	3.756164384	0	9.033211	7.450534	6.784998	5.563691	4.687844	0.900182071	low
TCGA-BH-A42T	0.876712329	1	9.528101	6.933819	5.306919	9.576187	3.780932	1.037335193	high
TCGA-AC-A2FE	7.221917808	1	7.378248	6.804846	6.348783	9.918015	6.265626	0.769235281	low
TCGA-A8-A0A4	1.084931507	0	7.938646	7.367129	6.506612	5.813904	6.135565	0.868953974	low
TCGA-A2-A0ST	8.265753425	0	8.980751	6.64576	6.175934	7.203007	8.484562	1.071984854	high
TCGA-A7-A0CH	2.956164384	0	6.927215	7.47524	7.058145	4.768541	6.903026	0.761978244	low
TCGA-BH-A0BV	4.161643836	0	8.071492	7.987304	6.588173	5.919413	4.952027	0.804563884	low
TCGA-BH-A1FR	4.498630137	1	7.78173	8.59292	6.392025	6.536816	3.365808	0.725334809	low
TCGA-D8-A27I	1.202739726	0	7.591505	7.765702	6.383665	10.398596	5.433667	0.700599619	low

TCGA-A2-A1FZ	1.871232877	0	6.988006	7.248612	6.556789	10.739261	4.029333	0.642944254	low
TCGA-AO-A0JB	4.224657534	0	8.009588	4.629115	4.776493	6.604347	7.09133	1.373054244	high
TCGA-E2-A14Y	5.778082192	0	9.787542	3.89088	5.490264	4.529632	9.267481	1.756630602	high
TCGA-A8-A09B	1	0	7.319914	6.931431	6.210536	9.171124	6.297123	0.792365092	low
TCGA-C8-A1HN	1.079452055	0	9.206536	6.703416	7.274424	4.75912	6.384179	0.966207531	low
TCGA-PE-A5DC	3.917808219	1	9.718688	5.237005	4.143551	7.539452	5.847939	1.591527305	high
TCGA-BH-A0BL	6.24109589	0	8.610797	6.204176	6.168501	10.635517	7.712211	0.939224606	low
TCGA-D8-A1X9	1.991780822	0	7.904851	4.79882	6.288165	4.938038	4.908835	1.090561635	high
TCGA-A7-A26H	1.983561644	0	7.023618	6.558887	6.90271	8.481164	10.769176	0.814266975	low
TCGA-E9-A2JT	0.789041096	0	7.463777	6.866529	5.743273	9.819677	5.00322	0.819555782	low
TCGA-E2-A1IK	4.931506849	0	7.433367	7.376017	6.506498	6.933761	4.396674	0.765969927	low
TCGA-A2-A0EN	11.2	0	6.378517	7.323935	5.911634	10.172672	4.126228	0.674112937	low
TCGA-D8-A1XL	1.660273973	0	8.943702	5.187115	7.043442	4.816465	4.971132	1.057524223	high
TCGA-A7-A13D	2.643835616	0	9.625011	4.498048	5.495521	4.38968	4.165512	1.475209421	high
TCGA-BH-A0DQ	0.268493151	0	8.613916	5.741322	5.99083	9.606676	6.359182	1.00185214	high
TCGA-A8-A097	1	0	7.906909	6.698415	5.902546	7.885827	4.037192	0.883947753	low
TCGA-A8-A08L	0.082191781	1	9.581659	5.092023	6.54	7.182132	9.977144	1.259607659	high
TCGA-EW-A1P7	2.506849315	0	7.238889	7.030295	6.845489	9.09524	6.686324	0.721615532	low
TCGA-E2-A15H	1.076712329	0	8.471195	5.725817	6.041085	5.525096	8.913585	1.192419054	high
TCGA-A8-A08J	3.087671233	1	9.298542	5.385698	5.796327	5.90789	6.810402	1.291742153	high
TCGA-A2-A0CP	7.706849315	0	5.986635	6.170828	6.262414	8.103886	4.739481	0.733626859	low
TCGA-E2-A14V	2.854794521	0	10.258698	4.808552	6.641319	6.591638	5.886964	1.259102874	high
TCGA-AO-A0JI	4.18630137	0	8.334353	5.222737	6.987128	6.808857	4.122408	0.920178729	low
TCGA-D8-A1XV	1.263013699	0	6.178333	6.504529	6.525493	3.85967	3.294399	0.782073388	low
TCGA-LD-A74U	1.101369863	0	7.636379	6.761932	6.04137	9.800011	10.693475	0.917315202	low
TCGA-A7-A6VV	0.857534247	0	9.784035	5.585186	5.232823	5.911797	11.235484	1.595527126	high
TCGA-AQ-A04J	2.243835616	0	8.474751	6.040771	5.887101	6.649146	8.044846	1.123635016	high
TCGA-LL-A50Y	2.087671233	0	7.736656	7.814952	6.464058	8.374116	11.137533	0.8521318	low
TCGA-AR-A1AK	8.654794521	0	8.223351	8.253677	6.801373	8.09324	7.52094	0.765415892	low
TCGA-E2-A572	3.309589041	0	6.748034	5.02165	5.839599	8.111872	4.51688	0.91089545	low
TCGA-AC-A23C	1.602739726	0	7.647441	6.656043	6.733001	8.351805	7.00523	0.810468896	low
TCGA-BH-A0AV	4.98630137	0	8.544876	7.055554	6.286471	7.674641	10.565871	1.011938034	high
TCGA-E9-A1NA	3.046575342	0	8.007677	5.523035	6.087033	5.149864	3.5125	1.030685657	high
TCGA-B6-A402	6.249315068	0	9.148426	4.176907	5.778355	3.717334	11.868702	1.690821379	high
TCGA-A1-A0SJ	1.139726027	0	9.50985	4.991092	6.541286	9.630781	8.880482	1.133309569	high
TCGA-A2-A0YL	4.038356164	0	7.847829	7.364898	6.895282	7.180196	6.340952	0.783040272	low
TCGA-AQ-A0Y5	0.471232877	1	7.647429	4.489092	6.624694	4.226361	7.826712	1.13696313	high
TCGA-E2-A14R	3.216438356	0	9.478032	3.904834	6.974094	4.663583	8.081526	1.339155724	high
TCGA-AR-A1AX	7.202739726	0	7.801034	7.290354	6.448235	5.386418	4.62616	0.852430172	low
TCGA-E9-A5UO	2.150684932	0	8.22255	4.906451	5.839721	4.735305	4.641367	1.18868833	high
TCGA-AC-A62V	0.953424658	1	9.704439	4.14047	5.261585	9.388012	4.644716	1.352166212	high
TCGA-E2-A108	2.293150685	0	8.066683	7.034279	5.900112	11.012721	5.133204	0.808455085	low
TCGA-B6-A400	0.589041096	0	9.900901	4.728462	5.944136	6.915543	8.755356	1.425766659	high
TCGA-A8-A079	0.750684932	0	10.334479	4.981181	5.613694	6.778687	5.521924	1.425639969	high
TCGA-EW-A1PB	1.665753425	0	10.073379	5.383769	6.18052	3.689417	6.334325	1.404509943	high
TCGA-BH-A0EE	2.583561644	0	8.541978	4.263873	6.837763	3.391484	9.553317	1.30865353	high
TCGA-A8-A08B	3.167123288	0	9.221946	4.225475	7.392443	6.173612	7.033647	1.115849829	high
TCGA-AR-A0TP	11.71232877	0	8.607915	7.649607	5.851725	3.523888	6.531064	1.082903303	high
TCGA-BH-A0BP	6.290410959	1	7.624565	6.649253	5.673761	10.903223	9.679448	0.916968019	low
TCGA-E9-A3QA	2.515068493	0	9.275262	5.065745	6.085462	5.708068	7.10187	1.285309219	high
TCGA-AR-A1AW	7.210958904	0	7.783326	4.976216	6.092016	6.769284	4.093006	1.009512329	high
TCGA-BH-A1EN	5.82739726	1	7.150179	4.63081	5.580239	6.91619	10.866735	1.216811459	high
TCGA-BH-A0B9	4.306849315	0	9.72286	6.446891	6.938017	5.959411	9.892154	1.130224928	high
TCGA-C8-A135	1.076712329	0	8.574142	6.316383	5.774758	7.791191	10.594271	1.151014594	high
TCGA-A2-A0CU	0.432876712	1	8.028545	6.083182	6.659014	8.959041	3.691834	0.807181365	low
TCGA-EW-A1IW	1.016438356	0	8.409606	6.960692	7.008648	9.515347	6.839443	0.785468548	low
TCGA-AN-A0XO	1.02739726	0	7.815377	5.334648	6.573783	5.241452	9.388615	1.092148935	high
TCGA-E2-A1L8	6.136986301	0	7.309896	5.5025	7.152344	10.146642	5.428501	0.734788021	low

TCGA-E9-A5UP	2.2	0	6.377312	4.137007	6.231736	4.152771	7.498394	1.087009212	high
TCGA-BH-A0GZ	0.898630137	0	7.791643	6.417411	6.007034	6.849476	3.910799	0.908560678	low
TCGA-EW-A1P6	1.539726027	0	7.764025	6.523974	6.991386	5.448238	4.253602	0.82607522	low
TCGA-AO-A03O	6.802739726	1	9.255442	5.240435	6.938462	7.897497	4.256813	0.978210627	low
TCGA-AC-A2FB	3.380821918	0	6.994077	7.451258	6.3808	6.41673	5.350205	0.772429831	low
TCGA-BH-A42U	9.216438356	0	6.352804	6.918967	5.802415	10.117928	8.467236	0.779032154	low
TCGA-S3-A6ZH	1.756164384	0	8.257401	5.462759	6.145759	7.549717	5.826618	1.0237448	high
TCGA-A8-A09X	1.167123288	1	8.522296	5.360676	5.994903	7.371472	6.164203	1.096086111	high
TCGA-EW-A1PG	2.879452055	0	5.621171	6.148664	6.745812	7.300651	5.698013	0.695664709	low
TCGA-C8-A8HP	1.084931507	0	8.527982	4.711572	5.643338	8.359299	10.846391	1.304095228	high
TCGA-BH-A8FZ	1.57260274	0	7.191647	6.374145	6.177917	8.033672	6.947787	0.865155088	low
TCGA-D8-A1JB	4.624657534	0	7.042912	7.639796	6.15291	5.337832	6.62775	0.84277411	low
TCGA-C8-A1HL	0.868493151	0	9.830651	4.844555	5.411078	6.920047	9.934214	1.553794575	high
TCGA-B6-A0IG	12.20821918	1	8.631297	4.857734	6.146169	8.082981	5.885842	1.093967615	high
TCGA-E2-A1LE	2.408219178	1	8.725623	6.42387	5.451946	9.570083	11.459994	1.165254315	high
TCGA-C8-A1HO	1.02739726	0	8.512309	4.768818	6.934983	4.840849	3.4506	1.026916582	high
TCGA-A8-A08S	2.750684932	0	8.488931	5.403876	6.852118	5.628338	10.605445	1.131249889	high
TCGA-A8-A08T	9.339726027	1	7.451776	6.389407	6.740573	6.60125	3.823031	0.799652656	low
TCGA-OK-A5Q2	0.175342466	0	8.38643	6.516962	6.354946	9.30307	10.839875	0.980807572	low
TCGA-OL-A5RV	2.909589041	0	6.651438	5.803807	6.760568	6.888527	7.469621	0.831087605	low
TCGA-BH-A18I	2.994520548	0	7.752018	6.187117	6.643022	7.784664	4.809788	0.833443685	low
TCGA-BH-A0DD	6.810958904	0	9.384593	5.489948	6.995092	3.903451	3.334236	1.07791335	high
TCGA-A8-A09K	2.498630137	0	8.132487	5.051473	4.158446	6.630686	3.169764	1.340897222	high
TCGA-AC-A8OS	0.191780822	0	6.504346	7.285585	5.76491	8.985146	10.890507	0.847562787	low
TCGA-AO-A03R	5.728767123	0	9.706249	5.437168	5.887766	8.919509	5.317471	1.155314458	high
TCGA-A7-A6VX	0.868493151	0	8.404094	5.88458	7.645582	7.302525	4.377066	0.793758638	low
TCGA-A8-A07C	2.832876712	0	8.942619	4.725284	7.069261	5.733512	10.497357	1.200749393	high
TCGA-E2-A1B0	4.468493151	0	8.727963	4.554795	6.434173	8.541175	5.319202	1.055324	high
TCGA-E2-A10F	2.405479452	0	7.665818	7.388778	6.852393	8.326599	3.580799	0.698535475	low
TCGA-LL-A73Y	1.306849315	0	8.152037	5.844249	6.135402	7.376273	10.000521	1.089711952	high
TCGA-3C-AALJ	4.038356164	0	9.159466	4.514372	6.334236	9.657789	6.415663	1.105657515	high
TCGA-AR-A1AV	5.106849315	0	7.395062	5.943463	7.019041	7.818558	5.045188	0.781503096	low
TCGA-AO-A129	9.002739726	0	9.44887	5.335157	6.347511	5.70681	11.023552	1.348740289	high
TCGA-UU-A93S	0.317808219	1	9.328311	5.181561	5.454176	5.212039	5.393777	1.368588335	high
TCGA-A7-A5ZW	0.893150685	0	6.472182	6.535734	7.002826	6.256528	5.841229	0.734211499	low
TCGA-OL-A66L	3.564383562	0	6.306689	5.259825	5.364364	11.210391	6.61403	0.866997902	low
TCGA-E9-A22D	3.419178082	0	9.46268	5.092413	6.328543	4.3973	10.134792	1.412380903	high
TCGA-E9-A1R2	2.912328767	0	8.528391	6.673871	6.424947	8.373015	4.843264	0.875455614	low
TCGA-BH-A0HY	4.232876712	0	8.903286	5.300221	5.215836	8.329941	7.428942	1.270955275	high
TCGA-E2-A15L	1.715068493	0	8.364052	4.584311	6.958836	7.449471	6.064415	0.995813853	high
TCGA-BH-A0HU	1.073972603	0	9.698965	4.556364	6.425967	7.724853	5.18971	1.188528348	high
TCGA-BH-A0H3	5.282191781	0	6.19483	7.197647	6.998494	7.742078	5.840317	0.646847806	low
TCGA-E2-A1BC	1.37260274	0	4.820727	6.590562	7.088876	10.936027	5.419478	0.522172912	low
TCGA-BH-A0HF	1.991780822	0	7.760236	6.815761	6.267139	10.252097	7.63872	0.823020435	low
TCGA-BH-A0HP	1.134246575	0	7.767723	8.07418	6.406949	7.194793	5.275235	0.768745801	low
TCGA-AR-A251	8.301369863	0	10.169091	5.258544	7.091285	9.223525	9.041432	1.113792287	high
TCGA-AR-A2LN	3.180821918	0	5.30677	7.468631	6.606772	10.428846	6.412616	0.569288581	low
TCGA-AC-A5XS	1.610958904	0	8.4	6.538789	6.544894	9.931934	7.255854	0.86168021	low
TCGA-E9-A24A	2.046575342	0	7.044954	9.193291	6.809465	4.881702	9.458451	0.737964843	low
TCGA-C8-A1HE	1.02739726	0	7.12861	7.337714	6.638834	6.647443	7.579019	0.794656411	low
TCGA-BH-A1FB	10.05205479	1	6.318744	8.061403	6.87202	7.307253	5.949368	0.634027333	low
TCGA-A8-A09N	0.084931507	0	8.856312	5.275299	6.201733	4.39936	6.918941	1.242839531	high
TCGA-A8-A0AB	1.419178082	0	7.554117	5.738761	6.734593	7.673026	4.163815	0.826542126	low
TCGA-A2-A0SU	4.553424658	0	7.618959	6.663332	6.335315	6.796612	5.538585	0.870193748	low
TCGA-E9-A1N4	2.739726027	0	7.407929	5.995379	6.729775	6.649223	5.727208	0.857124504	low
TCGA-OL-A66J	5.468493151	0	7.113874	5.73418	5.735615	11.511006	4.705794	0.812867943	low
TCGA-LL-A9Q3	1.457534247	0	7.774055	4.502229	5.461533	6.366524	5.023882	1.183589916	high
TCGA-BH-A0BG	5.126027397	0	8.546017	6.746318	6.829688	8.404342	5.822673	0.841381402	low

TCGA-B6-A0RL	6.764383562	1	10.046538	5.390254	7.416208	4.462413	4.460951	1.098832366	high
TCGA-A8-A08C	2.41369863	0	6.871192	6.464793	7.742984	7.753999	3.246358	0.620000304	low
TCGA-OL-A5RW	3.030136986	0	9.879609	4.079036	6.576672	4.030616	11.58219	1.606229221	high
TCGA-BH-A1F0	2.150684932	1	8.741172	5.991099	6.580683	9.768745	8.784809	0.962196001	low
TCGA-E2-A1LG	4.17260274	0	8.796875	3.88634	5.453978	3.708415	11.202647	1.723950282	high
TCGA-A2-A3XS	2.82739726	1	9.458455	5.221634	6.202156	6.109175	12.631213	1.422609956	high
TCGA-AQ-A1H2	1.301369863	0	7.177374	7.265714	3.727529	3.583814	7.177836	1.327510754	high
TCGA-EW-A3E8	2.835616438	0	7.703738	5.893396	6.271377	10.55446	6.258012	0.842821771	low
TCGA-B6-A2IU	14.18082192	0	7.939278	6.731607	6.915076	7.566952	6.142179	0.813019382	low
TCGA-JL-A3YX	0.964383562	0	8.479152	4.884215	6.735468	7.038629	5.742336	1.021780841	high
TCGA-EW-A1OZ	3.367123288	0	8.959907	4.241916	6.060046	7.928619	4.592658	1.169933164	high
TCGA-EW-A6SB	2.082191781	0	8.861649	4.938462	4.862269	3.37321	10.876592	1.744878565	high
TCGA-A2-A0EY	5.273972603	0	8.370339	5.410991	7.531689	6.988758	10.157818	0.960974899	low
TCGA-C8-A138	1.04109589	0	9.654233	5.222136	7.024793	7.114072	11.37518	1.213471906	high
TCGA-BH-A0BF	3.62739726	1	8.550491	6.057541	5.841245	7.952258	3.302882	0.978187194	low
TCGA-B6-A1KF	8.460273973	0	9.891187	5.170531	6.514501	4.926453	5.109903	1.249299053	high
TCGA-A2-A0CT	6.271232877	0	9.330083	6.482221	6.354299	5.516744	4.838568	1.066014317	high
TCGA-E2-A1IL	0.323287671	0	6.402726	6.691243	6.958635	10.140698	6.532385	0.647327325	low
TCGA-D8-A1JM	1.616438356	0	9.850828	4.247612	5.749816	4.067231	3.875907	1.489199065	high
TCGA-AN-A0XL	0.446575342	0	8.361614	6.628624	6.306768	10.693715	11.168724	0.939523046	low
TCGA-BH-A0GY	2.528767123	0	8.635671	5.35347	6.362953	7.657223	8.553828	1.100963529	high
TCGA-AR-A0TV	6.268493151	0	9.208667	5.495199	6.06153	4.256251	3.745665	1.20537224	high
TCGA-B6-A40B	8.635616438	0	8.207287	5.1039	5.973668	7.82181	7.955393	1.11619391	high
TCGA-C8-A134	1.049315068	0	10.02413	5.316219	6.30891	5.256616	4.704735	1.262284999	high
TCGA-AC-A3TM	2.087671233	0	9.134163	5.628442	6.108889	7.03739	6.132821	1.132469252	high
TCGA-BH-A18T	0.61369863	1	10.218278	4.755834	6.108128	8.046323	9.659301	1.409216923	high
TCGA-A2-A0CX	4.734246575	0	8.711534	5.04683	6.129636	5.185257	4.741297	1.168386671	high
TCGA-AN-A04C	0.147945205	0	8.930716	4.336532	5.632186	7.441683	11.230889	1.453736521	high
TCGA-C8-A131	1.126027397	0	9.723141	4.748185	5.042724	4.967054	9.681385	1.731294375	high
TCGA-A2-A0ES	6	0	6.805238	6.393675	6.623079	9.120304	5.187619	0.724365159	low
TCGA-EW-A6S9	1.268493151	0	8.068021	6.363726	8.415693	6.490284	7.222398	0.728174957	low
TCGA-D8-A13Y	4.734246575	0	10.781757	4.323341	6.24267	3.74327	3.957058	1.530112747	high
TCGA-B6-A0WS	8.123287671	1	7.361009	6.630694	6.870505	7.931617	5.620002	0.761337192	low
TCGA-A2-A0D4	2.101369863	0	9.528753	4.398048	6.829432	9.057686	5.438491	1.075758173	high
TCGA-A8-A07F	1.580821918	0	7.354111	6.456719	7.700243	6.457808	5.154636	0.712940836	low
TCGA-OL-A5D7	4.876712329	0	11.303574	4.55941	5.589476	7.179498	11.098561	1.817484101	high
TCGA-D8-A1XG	1.22739726	0	7.118741	8.270547	6.863104	4.174527	3.974644	0.715968591	low
GSM3818018	8.147917808	0	9.107765	6.272478	6.210225	11.190962	4.607588	0.891406514	low
GSM3818020	9.268520548	0	8.649469	5.502129	6.274212	6.872986	7.388405	1.103106586	high
GSM3818024	3.77260274	0	7.392445	5.715753	6.419365	6.855601	9.563252	0.990020845	low
GSM3818025	1.942438356	1	11.292016	6.628185	6.148814	5.529766	4.402861	1.297507583	high
GSM3818030	6.189041096	0	8.526948	7.216843	6.360441	6.106101	7.779578	0.976387774	low
GSM3818031	8.663013699	0	7.907059	8.04529	7.271537	5.308199	7.097701	0.767226547	low
GSM3818032	4.15890411	0	8.42046	4.716402	6.867125	5.671853	5.718657	1.056770057	high
GSM3818034	8.23290411	0	7.49789	6.433851	7.357022	6.364545	4.572955	0.752395947	low
GSM3818035	9.471205479	0	8.632665	6.597045	7.367282	17.173638	5.255798	0.58680808	low
GSM3818038	9.043808219	0	7.390419	6.247522	7.134807	5.350828	5.311661	0.819827702	low
GSM3818044	7.054767123	0	7.978481	6.779724	6.177111	12.826845	4.784542	0.734368652	low
GSM3818048	7.347945205	0	9.482943	5.898894	6.905798	5.23147	5.73876	1.078938564	high
GSM3818049	7.479452055	0	10.088621	5.877704	6.243989	7.275041	7.009242	1.209444074	high
GSM3818051	6.857506849	0	6.530004	6.184306	5.520238	9.992631	9.065111	0.888374061	low
GSM3818052	3.805479452	0	7.326766	5.995541	5.78182	5.889305	6.50778	1.014645076	high
GSM3818053	9.482219178	0	8.760106	6.194473	6.171029	5.848662	8.061495	1.126608551	high
GSM3818054	9.506876712	0	9.837188	5.430187	5.672066	12.145479	5.172775	1.080428479	high
GSM3818058	0.334273973	0	11.020194	5.600711	6.766264	6.723974	10.610873	1.388160688	high
GSM3818061	5.419150685	0	9.447799	5.846808	5.269217	5.036991	12.042169	1.579121238	high
GSM3818062	8.736986301	0	10.163902	5.281342	6.48096	5.996913	5.033081	1.230845333	high
GSM3818064	8.630136986	0	8.048007	4.839317	6.336488	5.634994	4.777181	1.066510805	high

GSM3818065	9.254794521	0	7.803742	5.409635	6.561848	6.811148	6.379648	0.963039156	low
GSM3818066	10.09586301	0	7.505817	4.979305	5.671423	5.88342	7.16868	1.151506026	high
GSM3818067	8.4	0	7.149128	4.823358	6.292594	6.172349	5.190142	0.976918934	low
GSM3818068	12.03558904	0	8.694341	5.102038	5.78932	6.553078	11.155323	1.347039099	high
GSM3818070	9.032876712	0	6.799487	5.380661	6.294681	10.828954	5.055244	0.772341531	low
GSM3818071	2.630136986	0	7.745253	4.998461	6.914514	6.053585	4.823749	0.931104082	low
GSM3818073	10.074	0	6.793516	5.986287	6.653707	7.938084	9.432673	0.851584654	low
GSM3818079	9.17260274	0	9.727044	5.625329	5.562651	6.99862	11.301449	1.458635211	high
GSM3818082	9.169890411	0	9.005209	5.578152	6.514666	5.515858	4.26558	1.069139745	high
GSM3818083	10.01646575	0	9.22795	6.055367	5.390543	5.413281	4.420278	1.242038582	high
GSM3818084	10.32328767	0	8.063175	6.2873	6.206575	7.719652	4.563806	0.903148172	low
GSM3818085	9.090410959	0	9.244795	4.811533	5.704489	6.081022	9.371242	1.43409525	high
GSM3818086	10.38082192	0	8.234967	6.094737	6.782122	6.734883	4.623357	0.889223759	low
GSM3818087	1.37260274	0	8.34689	5.591176	6.429594	5.596393	4.845827	1.025172882	high
GSM3818088	10.08764384	0	10.320865	5.676979	6.425095	6.366399	9.970677	1.349939337	high
GSM3818089	9.013726027	0	10.336801	6.212235	6.536336	6.101319	12.66843	1.36984511	high
GSM3818090	9.230136986	0	8.352754	6.724538	5.834283	6.294164	7.245352	1.054497517	high
GSM3818092	3.474	0	6.927176	5.773046	6.694988	6.59671	5.296168	0.829856395	low
GSM3818093	8.320520548	1	9.661397	4.776712	5.926716	8.074605	11.465839	1.423888094	high
GSM3818095	8.449315068	0	7.630632	6.25687	5.849807	6.555849	9.399596	1.05931565	high
GSM3818097	9.490438356	1	8.329525	5.400419	6.525152	6.718062	9.0135	1.08531072	high
GSM3818099	9.857506849	0	8.418308	5.844523	5.532423	8.782219	13.628958	1.261384023	high
GSM3818100	8.909589041	0	8.197313	5.748773	6.322121	5.726683	5.76445	1.030399411	high
GSM3818103	4.578082192	0	9.307734	5.647505	5.2029	6.051364	7.195859	1.382979497	high
GSM3818104	11.05208219	0	6.530706	5.161081	6.385264	7.309131	5.353271	0.855369152	low
GSM3818105	10.08221918	0	8.116539	5.493522	5.339951	6.535882	11.063061	1.314862984	high
GSM3818106	10.68493151	0	9.739284	5.516748	6.256894	5.610371	4.680099	1.203183325	high
GSM3818108	3.221917808	1	9.364967	5.231048	5.363512	5.691045	10.736673	1.539945057	high
GSM3818110	11.14249315	0	7.596836	5.978466	5.849807	7.456068	4.666619	0.940241407	low
GSM3818112	10.83838356	0	8.344464	5.525384	5.305107	5.510139	3.863281	1.183717905	high
GSM3818114	9.145232877	0	8.658961	4.184049	6.302012	5.674532	7.483224	1.269921729	high
GSM3818119	3.701342466	1	10.80351	5.985355	5.490197	5.891623	6.525975	1.479924024	high
GSM3818120	10.45750685	0	7.662284	5.662817	5.672123	7.398847	5.199215	1.008144478	high
GSM3818123	6.060246575	1	7.919618	5.18545	5.737702	7.984073	4.529942	1.026229816	high
GSM3818124	3.180821918	0	8.215673	5.239696	5.839672	8.814677	5.986331	1.042224631	high
GSM3818126	10.47673973	0	9.022512	5.985611	5.825414	6.438782	5.521606	1.141103148	high
GSM3818128	10.33421918	0	6.890986	6.004011	5.890819	5.848099	4.29087	0.911651435	low
GSM3818129	8.243835616	0	6.964189	5.964176	5.17928	9.082339	9.564173	1.030611408	high
GSM3818130	5.306876712	0	9.221054	4.721442	4.950873	5.257948	4.4049	1.470913915	high
GSM3818132	8.178082192	0	7.99963	5.67385	6.054694	7.660976	7.160078	1.02197376	high
GSM3818133	10.26024658	0	7.743571	5.674114	6.117912	6.729813	6.106546	0.99515579	high
GSM3818134	10.84660274	0	9.641972	5.596235	6.763915	6.417916	7.015344	1.132280159	high
GSM3818135	10.62189041	0	8.028339	5.486668	6.813473	6.638059	11.834239	1.074982355	high
GSM3818136	10.65756164	0	7.081055	5.794206	6.290678	6.018001	6.057764	0.923308636	low
GSM3818140	2.323315068	0	7.452872	5.882233	5.901463	10.337147	8.490468	0.91919274	low
GSM3818142	10.04934247	0	8.969183	5.473707	5.910614	7.139478	12.135453	1.324964327	high
GSM3818143	10.50139726	0	6.663024	6.671322	6.27806	7.771046	5.020205	0.764944879	low
GSM3818145	11.35890411	0	9.388338	7.13593	6.967628	8.943041	10.301036	0.944438285	low
GSM3818147	11.16164384	0	8.968312	5.925929	6.734027	8.365831	4.707015	0.923842683	low
GSM3818148	5.575315068	0	7.700428	7.185914	6.433555	4.967386	4.506557	0.862420368	low
GSM3818149	7.558931507	1	8.800444	5.300505	6.187111	6.29291	3.933319	1.084706953	high
GSM3818151	11.86027397	0	9.375781	6.047024	6.33746	5.684793	7.773273	1.179891826	high
GSM3818152	4.265753425	1	7.563548	7.412025	7.121474	7.201744	12.11635	0.837809853	low
GSM3818153	10.35616438	0	7.59897	7.72201	5.514622	4.850221	4.216007	0.93010239	low
GSM3818154	10.70136986	0	10.194251	5.87878	5.254965	9.740463	9.541053	1.370446016	high
GSM3818155	4.435643836	0	11.971158	6.924519	6.018175	5.290725	6.992451	1.474383041	high
GSM3818156	3.920547945	1	7.460142	6.343919	4.958215	5.631891	9.811626	1.221585034	high
GSM3818158	3.556191781	1	9.941779	4.920135	5.711963	5.674151	8.27476	1.502173452	high

GSM3818159	8.430164384	0	10.570089	7.284708	6.437605	5.166684	11.232598	1.305660755	high
GSM3818164	11.98906849	0	7.317095	5.339566	6.49498	5.512057	9.799308	1.052486236	high
GSM3818166	1.832876712	0	6.836423	6.039067	5.603794	5.670412	4.304418	0.947606484	low
GSM3818167	6.098630137	0	9.009348	5.734319	6.112065	5.471881	4.433096	1.124315876	high
GSM3818169	11.72326027	0	7.035138	6.389923	6.162888	5.730565	5.356146	0.888145884	low
GSM3818170	12.09312329	0	8.982067	5.072054	5.532829	5.451398	10.15281	1.459299611	high
GSM3818171	11.26027397	0	6.79189	5.458323	5.619251	5.552228	7.432845	1.06140504	high
GSM3818175	12.126	0	8.198453	6.141071	6.269816	6.38787	3.96566	0.945783005	low
GSM3818176	10.63010959	0	7.472484	5.802644	7.427732	5.467031	4.558173	0.803615486	low
GSM3818177	10.82194521	0	6.792953	5.655087	7.050945	6.892407	5.480376	0.781824308	low
GSM3818181	5.501342466	1	9.799794	5.368198	7.290071	5.516817	4.125123	1.04836693	high
GSM3818182	11.58632877	0	7.882248	5.067045	7.122922	8.525108	6.153792	0.865198093	low
GSM3818183	11.55887671	0	8.139998	5.311144	7.308861	6.114073	4.675326	0.888330437	low
GSM3818185	1.000027397	0	6.820438	5.443307	6.679401	5.03957	5.857555	0.90069471	low
GSM3818186	11.45753425	0	8.059652	6.656278	7.091791	6.024537	4.445205	0.817308702	low
GSM3818187	9.589068493	0	9.984926	5.391829	4.982149	5.42082	10.935539	1.726913652	high
GSM3818188	11.44380822	0	8.019648	6.944724	6.4562	5.389642	4.445587	0.889391384	low
GSM3818191	5.005479452	1	8.486569	6.70262	5.803343	5.511279	9.845408	1.17015647	high
GSM3818192	10.99454795	0	9.079343	6.102812	7.111245	5.150319	4.260595	0.962207937	low
GSM3818194	12.30682192	0	8.859077	6.526134	6.914119	5.019079	4.356244	0.943502212	low
GSM3818197	9.109561644	0	7.583413	6.36371	7.305896	5.308379	5.545127	0.813498208	low
GSM3818198	3.671260274	0	6.87578	5.485806	6.626568	13.34849	4.938832	0.675515471	low
GSM3818199	11.42736986	0	7.656938	5.59676	5.279184	7.323941	4.690001	1.0607463	high
GSM3818201	12.19175342	0	7.927085	5.890898	7.006518	5.151423	3.948103	0.881926335	low
GSM3818203	11.83010959	0	7.56028	5.653682	6.650902	5.342299	4.984924	0.927389087	low
GSM3818204	12.61915068	0	9.73178	5.699418	5.885345	5.388185	4.512151	1.253638398	high
GSM3818205	10.63010959	1	9.01489	6.633938	5.986379	9.910395	6.433344	0.964423533	low
GSM3818207	4.002739726	0	8.511118	6.122864	6.704546	5.707784	4.722508	0.955410777	low
GSM3818209	4.717808219	0	8.695694	5.527508	7.166919	9.0089	4.8167	0.856758323	low
GSM3818210	4.674	0	7.396366	5.469729	8.053083	5.618176	5.102654	0.755023781	low
GSM3818212	12.75345205	0	8.637785	6.378397	7.067545	10.251356	5.412194	0.786865639	low
GSM3818214	1.419205479	1	8.367001	5.962651	6.198719	6.106509	11.442606	1.177709	high
GSM3818215	10.9890411	0	9.073927	10.292703	5.032233	5.638979	4.423984	0.920938052	low
GSM3818216	12.14243836	0	9.322366	5.701648	6.421167	6.219627	4.764043	1.093071665	high
GSM3818217	12.69591781	0	7.522747	6.054417	5.39536	5.334533	4.235508	1.051422235	high
GSM3818221	2.065726027	0	7.621153	5.60453	7.174076	6.079706	6.414047	0.876811448	low
GSM3818226	4.676712329	1	10.029811	5.387009	5.804938	6.100309	7.871179	1.409023344	high
GSM3818227	6.054821918	0	8.71038	6.294441	7.190453	7.421393	10.164142	0.959412355	low
GSM3818231	6.128794521	0	7.772366	5.740733	6.447485	6.923871	4.571504	0.909382888	low
GSM3818232	6.315041096	0	7.531151	5.361137	6.835434	6.445408	5.02563	0.889276818	low
GSM3818233	5.750712329	0	7.818086	5.975281	6.838087	7.533351	11.060906	0.964132628	low
GSM3818234	6.547972603	0	6.469621	6.093354	7.059751	9.31791	7.6613	0.709285254	low
GSM3818236	6.082191781	0	7.087667	5.889732	7.400604	6.074805	5.501884	0.773049226	low
GSM3818238	6.106849315	0	6.232559	6.589441	7.285196	7.041865	6.19051	0.674239525	low
GSM3818239	6.402739726	0	6.429491	6.005814	7.078278	9.00922	4.999105	0.674728238	low
GSM3818240	5.630136986	0	9.0133	5.710942	6.770011	8.403681	5.356968	0.951394207	low
GSM3818246	6.304109589	0	8.753364	5.110246	6.738578	5.595234	4.900162	1.060724011	high
GSM3818250	5.975342466	0	8.231658	5.442951	6.883144	6.107103	4.947296	0.948028482	low
GSM3818255	5.901369863	0	6.902228	6.440158	7.110175	5.692218	4.221869	0.745826053	low
GSM3818256	5.871205479	0	5.917758	6.27193	7.090745	12.038033	5.364555	0.572627676	low
GSM3818258	0.361643836	1	7.546293	4.995745	4.96759	4.90661	10.533442	1.42162273	high
GSM3818261	6.476712329	0	7.717706	5.583865	6.404635	6.837211	9.20836	1.026488871	high
GSM3818264	4.564356164	0	9.196791	4.310188	6.538844	5.536123	7.138644	1.276958749	high
GSM3818266	5.923315068	0	7.774314	5.373373	6.75081	6.10505	5.401779	0.938889509	low
GSM3818267	6	0	8.012525	5.711084	6.965784	6.316402	5.458441	0.902849219	low
GSM3818269	3.830136986	0	7.46427	5.584095	6.339485	8.074786	6.518417	0.912639054	low
GSM3818274	2.874	0	8.171394	6.043102	6.463275	10.573397	12.179339	0.969886078	low
GSM3818275	5.775369863	0	7.061913	9.868658	5.959538	6.220751	4.191971	0.671079949	low

GSM3818276	7.328794521	0	8.193717	5.909591	6.95163	10.72291	7.409457	0.818509903	low
GSM3818280	5.117835616	1	8.415321	5.25854	6.183731	7.674058	7.814553	1.094349111	high
GSM3818283	6.219205479	0	9.618362	5.191657	6.386249	6.632638	7.39116	1.230981206	high
GSM3818284	7.095863014	0	6.353955	6.251738	6.570716	15.04004	5.141723	0.579531474	low
GSM3818285	5.287643836	0	7.056834	7.715423	6.557613	6.761558	4.633629	0.72236312	low
GSM3818286	7.060273973	0	8.79597	5.223686	6.446469	6.973345	10.260703	1.187003427	high
GSM3818288	6.219205479	0	7.190898	5.855127	6.247027	6.642398	12.232236	1.053152518	high
GSM3818291	7.093150685	0	9.438942	4.982763	6.654383	6.32881	4.605496	1.122632761	high
GSM3818292	7.060273973	0	9.307276	5.942663	5.359268	5.710238	6.039159	1.302684606	high
GSM3818293	6.756164384	0	6.46762	5.679238	5.379998	11.778228	4.267091	0.791348713	low
GSM3818295	7.298630137	0	5.879567	14.062362	7.669395	6.918726	3.912335	0.33046237	low
GSM3818296	4.849315068	0	8.785536	5.272094	6.936102	7.332833	5.312593	0.97366783	low
GSM3818297	6.501369863	0	7.550368	6.371595	6.277963	8.598326	4.445709	0.818904321	low
GSM3818299	10.86575342	0	8.529171	5.231048	6.611984	5.078541	4.855814	1.063973551	high
GSM3818300	8.778082192	0	7.71963	6.327003	7.124598	5.980691	10.422584	0.926423991	low
GSM3818302	9.364356164	0	10.585045	5.893706	6.083306	5.176178	10.523988	1.505453935	high
GSM3818304	5.238328767	0	7.781053	6.138399	6.470783	9.488612	4.276651	0.802405354	low
GSM3818305	4.720520548	0	7.266314	6.81975	5.443767	5.265198	4.670959	0.972076651	low
GSM3818306	5.021917808	0	8.196983	6.149914	5.97842	5.812852	5.09386	1.02967381	high
GSM3818308	4.958876712	0	8.477209	5.432978	5.853881	5.477035	4.391804	1.132470962	high
GSM3818309	4.71509589	0	5.729141	6.681749	5.762851	14.291539	8.175902	0.649628852	low
GSM3818312	1.221945205	1	7.948093	6.191158	6.236472	5.357104	4.015345	0.957290589	low
GSM3818313	5.523287671	0	8.38689	6.156202	5.150327	12.378233	4.68026	0.938001828	low
GSM3818314	5.778082192	0	9.309479	5.676838	5.431928	5.779993	3.90814	1.25131236	high
GSM3818315	2.608191781	0	8.148463	5.628212	5.65487	6.279367	11.158908	1.262067405	high
GSM4947165	7.6	0	7.39098	6.563274	6.028934	4.747588	4.96496	0.946458505	low
GSM4947167	6	0	7.818289	6.448537	6.344664	8.460053	10.308576	0.949834677	low
GSM4947168	6	0	6.454769	6.393524	6.053122	6.433409	4.993495	0.826123983	low
GSM4947171	0.1	1	8.588372	5.245194	4.276213	4.224615	5.109179	1.536992492	high
GSM4947172	5.5	0	8.689829	6.191314	5.866916	9.424014	4.147421	0.949025506	low
GSM4947173	5.5	0	8.860208	6.459577	6.129103	7.265053	3.999563	0.975199896	low
GSM4947177	4.2	0	8.682429	5.607328	5.905292	8.927834	11.170119	1.176749332	high
GSM4947180	4.5	0	7.985425	6.470632	6.330872	8.101449	5.695708	0.879842198	low
GSM4947183	5	0	7.420859	6.997209	6.179331	5.590165	5.476195	0.884037216	low
GSM4947184	1.3	0	6.515593	5.932704	5.569602	8.128855	8.684148	0.947480806	low
GSM4947185	7.5	0	7.588725	4.866326	5.894771	10.015709	7.425176	0.994753676	high
GSM4947188	5.3	0	8.989824	6.182065	4.771483	4.561581	11.123909	1.571248938	high
GSM4947189	5	0	7.982698	6.060562	6.223225	5.63211	5.53166	0.996958965	high
GSM4947192	7.4	0	6.71955	5.6732	5.943993	4.964569	4.641266	0.947790947	low
GSM4947198	5.7	0	7.711816	6.107436	5.509366	6.031773	9.493532	1.155480181	high
GSM4947204	1.3	1	9.699031	2.570002	4.550454	3.802148	10.722063	2.331556396	high
GSM4947216	6.8	0	8.771216	4.741258	6.025937	6.260691	7.946226	1.267006878	high
GSM4947218	6.6	0	10.138852	5.663994	5.972847	5.038358	12.098765	1.552309212	high
GSM4947222	6.8	0	9.110906	6.713362	6.350969	5.695644	7.288961	1.078035392	high
GSM4947223	1.6	1	6.581818	5.949327	5.769066	11.371164	8.231931	0.822798701	low
GSM4947225	7.6	0	7.390035	7.099021	6.116468	9.643092	4.800674	0.759294085	low
GSM4947227	5.5	0	9.528897	3.507072	5.897238	6.702264	10.034505	1.577677394	high
GSM4947228	6.1	0	7.932379	6.019225	6.608013	9.286447	5.344691	0.831367941	low
GSM4947229	5.8	0	6.602902	7.033569	6.427223	6.717821	4.175204	0.735708381	low
GSM4947230	5.7	0	9.46539	6.361926	5.789167	5.011391	5.995573	1.232414284	high
GSM4947231	1.1	1	9.95634	5.508385	6.682127	9.21421	6.956152	1.081684743	high
GSM4947233	6.4	0	7.164419	6.954119	6.114	9.463748	3.011347	0.725947847	low
GSM4947235	1.2	1	9.346425	4.416114	6.643264	8.580583	5.532096	1.103258463	high
GSM4947236	6.3	0	8.514136	6.399159	6.368609	5.56213	4.295219	0.97608328	low
GSM4947238	7	1	7.526137	5.724579	6.306213	7.370177	4.770331	0.897711858	low
GSM4947240	6.3	0	6.641316	7.145919	7.267197	7.579434	3.924511	0.628402165	low
GSM4947241	3.4	1	7.883728	6.68695	6.608013	8.436872	11.114249	0.921646376	low
GSM4947242	5.7	0	9.03639	5.413014	6.944585	7.083848	3.974441	0.963960204	low

GSM4947246	2.3	1	8.666884	7.059183	5.518166	9.630279	3.41426	0.908498369	low
GSM4947247	7.4	1	7.468424	7.313339	6.587169	9.099709	4.535379	0.712745845	low
GSM4947252	6.2	0	8.183539	6.657439	6.701272	4.25587	8.84741	1.024273294	high
GSM4947253	6.3	0	7.493978	6.100421	7.017435	5.84222	4.487541	0.822138745	low
GSM4947255	6.1	0	9.025757	4.455168	6.094881	7.842293	4.366196	1.14986041	high
GSM4947257	4.6	0	7.62023	6.343058	6.626602	8.021357	9.7096	0.903790837	low
GSM4947258	6.2	0	9.023886	6.925835	7.041231	4.961367	3.924372	0.905795577	low
GSM4947261	5.9	0	8.125274	7.111563	7.037184	4.059139	5.033735	0.865753437	low
GSM4947266	3.8	1	9.824551	5.211841	6.74986	5.633594	10.040554	1.307790447	high
GSM4947267	4.8	1	6.093599	6.868472	6.972713	3.594524	8.209582	0.798850302	low
GSM4947268	4.6	0	7.894507	6.994135	6.887928	6.993493	4.254249	0.777552889	low
GSM4947271	5	0	7.070275	6.126323	7.084803	7.52272	4.309094	0.734818039	low
GSM4947273	5.5	0	8.663249	6.545089	6.975389	6.527597	3.878968	0.862305548	low
GSM4947278	5.3	0	7.296438	5.610928	6.915575	5.911146	4.352899	0.845282199	low
GSM4947282	4.4	0	7.711816	9.380195	7.097268	3.438492	3.935403	0.689319441	low
GSM4947285	1.2	0	7.479351	8.704	6.443847	2.867951	5.381256	0.820079261	low
GSM4947288	4.5	0	9.390262	4.338974	4.739384	10.643252	8.420074	1.453090008	high
GSM4947292	4.9	0	7.435102	6.798005	6.606623	9.379216	4.96085	0.737563795	low
GSM4947294	6.1	0	6.367446	6.440143	6.138068	7.852019	9.374639	0.849917193	low
GSM4947295	6.1	0	7.899087	6.09334	6.332018	9.223948	4.392097	0.840039544	low
GSM519122	7.3	0	9.902939	4.85119	5.885343	6.343243	5.131717	1.336956999	high
GSM519123	6.8	0	6.546918	6.022124	7.049563	7.545036	5.75035	0.730829603	low
GSM519124	7.1	0	10.172674	5.044918	5.827155	6.726872	6.916044	1.401337927	high
GSM519125	6.5	0	7.619066	6.865229	5.123779	7.605956	9.318887	1.077924021	high
GSM519126	5.4	1	8.504549	5.339967	6.027115	8.704598	7.961482	1.087199681	high
GSM519127	7.1	0	7.453031	5.492945	5.712494	4.888828	7.617763	1.143161556	high
GSM519129	7	0	8.4384632	7.6009656	6.4199729	0.8835698	6.9155112	1.087141346	high
GSM519130	6.4	0	10.32270378	5.580459	6.44019974	1.58940208	8.47610139	1.537963422	high
GSM519132	8	0	5.833997	7.440453	6.782455	7.991249	3.679547	0.596815593	low
GSM519134	6.1	0	9.176484	5.035013	5.551407	7.367044	4.965718	1.240323159	high
GSM519135	3.5	1	7.837723	5.765133	5.248021	9.567537	8.659982	1.086968847	high
GSM519136	7	0	9.04079	4.988037	5.372758	4.83686	4.262817	1.348847519	high
GSM519137	7.2	0	10.020917	5.384306	5.465727	4.45259	5.553476	1.480174903	high
GSM519138	5.2	1	10.182796	7.265052	6.146099	3.015144	4.980676	1.223147734	high
GSM519139	7.1	0	9.267346	5.015661	6.94894	7.161532	4.089623	1.015803066	high
GSM519141	1.9	1	8.5382652	5.5870069	5.5553637	0.3376004	8.9628382	1.545312286	high
GSM519142	6.9	0	9.457346	4.557962	4.86659	7.155944	10.599571	1.666658633	high
GSM519146	6.9	0	8.007723	6.162449	6.836958	8.096352	5.18551	0.831291639	low
GSM519147	6.5	0	6.840614	7.938927	6.940388	5.80419	11.472629	0.794519649	low
GSM519149	4	1	9.694421	6.639283	6.13029	5.001311	11.06096	1.31919384	high
GSM519150	6.7	0	4.97938	6.575958	6.608137	8.545442	6.130074	0.625026244	low
GSM519151	6	0	8.406029	6.159026	6.199153	8.321777	6.778721	0.972829832	low
GSM519152	6	0	10.359432	5.391233	4.354648	6.625417	10.922226	1.878607508	high
GSM519155	6.7	0	7.603259	6.530847	6.054296	6.478998	5.144109	0.914723667	low
GSM519156	5.8	0	7.472992	8.852074	7.488741	3.629096	3.585059	0.65459975	low
GSM519159	6.4	0	8.313879	7.31078	7.180232	7.228906	4.742085	0.760677447	low
GSM519161	6.4	0	7.925877	6.76556	6.115199	7.275887	9.100339	0.97889222	low
GSM519167	7	0	8.565421	6.123569	7.740655	5.726611	7.084257	0.875159165	low
GSM519168	6.5	0	7.88749	7.438196	6.343631	7.294891	10.058718	0.915735532	low
GSM519169	6	0	6.859629	6.163423	6.611371	6.183889	2.863971	0.776519978	low
GSM519173	6.8	0	6.7375	6.875639	6.573102	8.277224	4.185439	0.701932314	low
GSM519175	5.3	0	6.997591	7.149015	6.744274	9.995087	8.646142	0.719044958	low
GSM519176	5.7	0	5.073512	8.218815	6.810985	8.691472	7.594514	0.555468166	low
GSM519178	5.2	0	7.112383	7.974566	7.07082	5.301005	3.399537	0.675934322	low
GSM519180	5.1	0	7.613821	7.974958	6.421376	7.535096	9.779552	0.834299162	low
GSM519181	6.1	0	9.817037	6.337319	5.403387	5.426337	9.178252	1.429970793	high
GSM519182	9.2	0	8.79922	4.062494	5.828504	4.012116	5.305329	1.397054855	high
GSM519183	6.5	0	6.656854	7.167683	7.085624	6.023015	1.957374	0.649129925	low

GSM519185	6.1	0	6.818601	6.714557	6.833294	7.390402	8.561971	0.785554237	low
GSM519186	6.6	0	8.377145	6.516144	5.321163	8.046814	10.066917	1.161407271	high
GSM519190	0.9	1	8.912623	3.509025	5.791935	5.912647	10.422804	1.562511983	high
GSM519191	6.4	0	8.333113	6.894935	7.20206	7.204815	7.978061	0.84509346	low
GSM519194	10.6	0	9.34694	5.112599	7.118353	6.099449	9.277658	1.156285473	high
GSM519195	14.1	1	6.328993	6.997668	6.48221	12.087022	5.450041	0.613922122	low
GSM519200	4.3	1	9.854518	4.727877	5.567596	5.032318	5.913696	1.493882609	high
GSM519202	4.7	1	9.69103	5.913238	5.697852	6.212738	8.23354	1.335307116	high
GSM519203	9	1	8.111216	4.710393	6.652352	3.976749	5.317052	1.108892585	high
GSM519207	13	0	8.18242	4.419114	5.433355	7.125427	9.847555	1.353501691	high
GSM519208	13.1	0	7.591277	6.198099	7.046907	4.062146	5.875542	0.898144274	low
GSM519209	12.4	0	6.827259	6.198435	6.551828	7.085139	6.445429	0.819585714	low
GSM519210	0.8	1	8.827797	5.106211	5.116083	6.386445	10.220784	1.476391334	high
GSM519212	6.3	1	8.05034	6.473592	6.62922	6.731078	4.493887	0.864373613	low
GSM519213	4.9	1	9.362754	5.944633	6.595472	3.38356	9.353574	1.281673127	high
GSM519215	13.3	0	9.44171	5.200986	5.739094	9.155036	7.846659	1.230835672	high
GSM519216	12.2	0	8.608549	6.358479	5.641285	7.536565	4.294063	1.025124303	high
GSM519221	12.2	0	6.586686	7.112925	6.519391	7.312121	5.06421	0.720968061	low
GSM519222	5	1	10.045271	5.253578	6.128204	9.507988	6.697289	1.184308248	high
GSM519223	1.4	1	9.235602	5.421051	5.57293	8.206587	7.542918	1.244664537	high
GSM519224	6.5	1	6.990145	6.163078	6.777567	10.218745	7.283327	0.74268336	low
GSM519225	5.7	1	8.520905	5.7779	5.976858	6.146062	6.094496	1.106377309	high
GSM519227	11.6	0	7.375824	7.068835	6.563861	6.42684	5.57001	0.80816198	low
GSM519228	12.1	0	7.894526	5.860092	6.963712	9.299765	4.536445	0.782737278	low
GSM519229	9.2	1	7.582281	6.614149	6.896181	8.408419	7.125297	0.790309099	low
GSM519232	12.1	0	8.760852	5.517079	6.010303	7.539488	3.835077	1.042876948	high
GSM519233	12.8	0	5.931247	7.245251	6.612436	8.627368	4.698237	0.62766727	low
GSM519238	11.4	1	7.686466	5.556502	6.956212	5.877065	11.322172	1.027888563	high
GSM519239	4.6	1	9.266129	4.307368	7.947909	4.605375	7.592174	1.098717504	high
GSM519241	11	0	9.873675	5.624704	8.401077	3.352626	2.248291	0.911515047	low
GSM519244	11	0	8.45426	5.742956	6.034832	5.39294	10.287671	1.233095406	high
GSM519246	8.9	1	7.437489	7.367392	6.85006	7.145575	7.394927	0.77657765	low
GSM519247	12.6	0	10.342363	5.266235	6.727583	8.495135	8.036465	1.193041063	high
GSM519249	11.2	0	9.708008	4.6096	5.603414	7.01281	9.30298	1.495558481	high
GSM519250	11	0	7.062379	7.753778	6.685179	8.299159	2.655785	0.642940656	low
GSM519251	11.3	0	9.509511	6.20457	6.004909	5.102816	6.446299	1.224086053	high
GSM519252	11.6	0	6.651043	7.31722	7.377418	7.246273	5.919848	0.646633659	low
GSM519254	11.9	0	6.767211	7.975973	6.640017	7.765939	6.129798	0.680933429	low
GSM519255	12.7	0	8.152592	6.3043	6.647309	6.001952	7.26367	0.962545325	low
GSM519256	12.8	0	6.52909	6.551185	6.924869	7.308257	5.721815	0.717939041	low
GSM519257	4.6	1	8.558546	5.285295	5.606446	7.337883	1.494301	1.052096876	high
GSM519258	10.9	0	6.451045	7.78094	6.422347	8.611829	6.090289	0.671611477	low
GSM519259	11.4	1	5.93015	5.287736	6.951172	4.141863	5.525688	0.823335292	low
GSM519264	11.1	0	10.065424	5.834526	5.848435	5.76232	3.146449	1.233072339	high
GSM519266	1	1	10.020917	5.353155	6.065601	5.820179	9.699905	1.431618399	high
GSM519269	5.5	1	6.402176	6.446639	5.79158	10.373079	8.626143	0.809123203	low
GSM519271	9.4	0	8.585723	7.069199	6.821368	10.028484	2.259497	0.720629064	low
GSM519272	11.1	0	8.712664	5.858139	5.587073	8.33738	11.101335	1.232688608	high
GSM519273	12.1	0	8.48843	4.317275	6.827325	6.144264	1.819614	0.993915939	low
GSM519274	10.8	0	10.208261	4.0525	5.925075	4.794778	5.519893	1.546895957	high
GSM519278	11.6	0	7.507162	6.924205	6.572766	7.105717	6.797918	0.831034909	low
GSM519280	2.2	1	8.78036	7.253218	5.708825	8.418161	9.366941	1.049915704	high
GSM519281	7.1	0	9.216581	5.328478	5.969951	6.119441	7.530792	1.267929094	high
GSM519282	10.2	0	9.027481	4.450324	5.755682	7.609063	10.606628	1.401479711	high
GSM519284	11.8	0	5.938342	7.373177	5.996613	8.659655	10.419974	0.771635648	low
GSM519288	11.7	0	8.273053	4.157829	6.365199	9.5504	4.829815	1.005697726	high
GSM519289	1.6	1	8.910799	4.525117	5.87023	5.673008	11.276622	1.468102696	high
GSM519291	11.7	0	6.844694	6.111115	6.215379	8.608899	4.891893	0.795242691	low

GSM519293	1.3	1	8.698595	4.92849	6.395609	5.438449	9.139304	1.243052229	high
GSM519295	10.4	0	8.207333	4.775625	6.79976	7.610169	5.148888	0.962882494	low
GSM519297	9.8	0	9.034495	5.335185	6.256262	8.559632	10.274596	1.173720288	high
GSM519298	1.2	1	9.277121	5.783754	5.170074	8.545442	6.891202	1.252980848	high
GSM519299	9.4	0	9.006258	4.613766	5.933919	4.166235	8.100298	1.426585581	high
GSM519300	11.3	0	9.972342	3.734672	4.756569	7.263482	10.803304	1.897672978	high
GSM519303	7.1	1	9.80763	6.120353	5.168789	7.747457	6.359256	1.303833257	high
GSM519307	11.3	0	7.983692	6.911977	6.361551	7.159889	3.429426	0.82962463	low
GSM519308	11.1	0	7.555538	7.552943	6.64201	7.100442	4.644753	0.750140756	low
GSM519309	1.6	1	6.211684	6.211151	6.799107	9.38311	6.96711	0.698588133	low
GSM519310	3.1	1	9.681114	6.017614	6.700375	8.577654	4.725568	0.980742496	low
GSM519312	11.1	0	9.232192	4.319566	4.95244	3.863222	10.318252	1.819454719	high
GSM519314	9.4	0	7.756899	6.66031	6.627198	6.175871	3.973521	0.833980287	low
GSM519317	11.1	0	7.782216	4.982248	6.936049	5.329166	6.827705	1.000026842	high
GSM519319	9.7	0	7.635277	5.706398	6.290136	6.418476	6.070854	0.968011311	low
GSM519322	3.4	1	7.726106	5.834192	6.684224	9.238399	7.040626	0.851394732	low
GSM519323	10.3	1	6.980161	8.059868	6.592198	6.0696	8.182551	0.770709848	low
GSM519325	11	0	8.486166	5.94301	5.680535	7.950045	11.06637	1.196883749	high
GSM519326	11.2	0	7.268818	4.929497	6.933112	4.679605	5.097163	0.939212736	low
GSM519328	7	0	8.232928	5.909655	6.498393	4.786154	5.89344	1.030793825	high
GSM519329	9.1	0	9.55598	5.258323	7.251045	6.655158	6.219159	1.048582057	high
GSM519331	11.1	0	7.747879	6.949532	7.21689	5.464592	5.172605	0.789048857	low
GSM519332	11	0	9.778721	3.438901	6.384424	4.043378	4.952644	1.476416064	high
GSM519333	7.5	1	10.250069	4.798917	6.179614	5.649304	4.66887	1.348523527	high
GSM519334	9.5	0	8.321129	4.452911	6.593173	8.309865	7.461882	1.058097479	high
GSM519335	11	0	8.340587	5.828743	6.247724	8.35215	8.684732	1.027487715	high
GSM519336	11	0	8.47082	6.717208	6.212639	4.742422	4.796879	1.007707929	high
GSM519337	2.7	1	8.365923	5.745915	6.725288	7.939021	5.73889	0.918952474	low
GSM519338	10	0	5.816386	6.910654	6.429313	10.456623	4.627357	0.614086915	low
GSM519340	11	0	8.73402	7.710372	6.847093	7.296855	4.70223	0.802409922	low
GSM519342	5.5	1	8.769578	5.790255	6.616749	7.068812	9.407444	1.081738631	high
GSM519343	10	0	8.172432	6.613485	6.312637	10.456623	7.824026	0.862083745	low
GSM519344	9.9	0	10.056327	5.823245	6.471083	6.508051	7.570223	1.21837241	high
GSM519347	10.1	1	8.563937	5.683959	5.489436	9.298481	3.970803	1.028468102	high
GSM519348	10.5	0	8.200913	6.37209	5.641608	4.496536	4.448965	1.093108272	high
GSM519349	1.9	1	7.656204	4.659636	6.573449	8.891878	4.902546	0.906334805	low
GSM519350	9.2	0	9.321405	6.032896	4.838491	8.533499	8.025348	1.327676893	high
GSM519353	7.5	0	6.546547	6.902096	7.691878	2.947769	7.756721	0.760638211	low
GSM519355	10.6	0	9.422593	4.673477	5.854414	5.595984	4.528682	1.314307199	high
GSM519359	1.3	1	8.356911	5.645685	5.560409	5.624316	10.638742	1.316229639	high
GSM519361	8.4	0	8.675164	6.047397	5.65475	7.255337	4.835413	1.077910228	high
GSM519362	9.1	0	9.755088	4.184919	4.935218	6.24359	7.152893	1.666451865	high
GSM519363	8.7	0	6.818969	6.393479	6.61876	7.362456	5.624036	0.777137131	low
GSM519367	3.7	1	9.2622447	4.3434506	6.0027593	4.8270881	10.8364178	1.539334252	high
GSM519370	5	1	6.867939	6.932792	6.962388	7.984074	5.870509	0.702889744	low
GSM519372	10.1	0	8.363853	4.496053	5.950911	5.977037	8.881293	1.293780806	high
GSM519375	8.9	0	9.309856	3.903743	6.183668	5.61052	7.090618	1.395495305	high
GSM519378	9.4	0	8.700261	2.016431	6.749312	3.690076	5.326585	1.439579337	high
GSM519379	9.2	0	6.171736	7.053454	7.11733	6.918266	4.109566	0.634189434	low
GSM519380	9.2	0	10.244903	5.14452	5.457006	5.764453	8.574135	1.581740045	high
GSM519382	3.1	1	7.079051	6.772785	6.979623	10.539936	5.129479	0.654358549	low
GSM519383	9.8	0	6.975561	6.972347	6.827325	6.459963	3.379856	0.717400855	low
GSM519388	9.5	0	7.86194	6.503241	6.321679	6.4116	5.213235	0.908543733	low
GSM519391	9.6	0	7.298172	6.492227	6.748314	8.733176	4.383252	0.736690118	low
GSM519392	8.3	0	7.235045	5.111952	6.10621	9.732418	6.512712	0.905346214	low
GSM519393	9.4	0	7.984243	3.658902	5.601769	6.397299	7.5474	1.337135122	high
GSM519395	8.6	0	7.304154	6.270362	6.531416	6.987494	5.051496	0.831868684	low
GSM519396	6.6	1	8.547289	6.008382	6.780201	6.981528	5.662649	0.937128852	low

GSM519397	9.3	0	6.122895	7.266993	6.660102	5.535519	4.068225	0.692700185	low
GSM519398	7	1	8.206012	5.040165	6.550147	4.062447	4.525093	1.083916006	high
GSM519401	9	0	10.548953	4.177105	5.949218	7.40309	5.531764	1.447165502	high
GSM519403	0.4	1	9.322718	7.420558	4.455687	7.069126	4.464542	1.219051542	high
GSM519405	8.9	0	8.980084	4.982568	5.383266	4.414319	5.221006	1.38841724	high
GSM519406	8.9	0	7.088163	7.040205	6.736057	10.379443	5.631014	0.675141745	low
GSM519407	8.2	0	8.037479	6.288886	6.563861	8.385595	5.10688	0.848228138	low
GSM519412	9	0	8.207982	6.169923	5.665385	8.7738	10.592772	1.104450114	high
GSM519414	8.7	0	7.989598	4.362494	6.267127	7.631092	9.332894	1.153163557	high
GSM519415	8.4	0	7.048199	7.610148	6.304312	8.358631	8.373068	0.77850534	low
GSM519416	8.3	0	6.62451	4.957223	6.322705	4.849947	9.61469	1.057596431	high
GSM519418	8.5	0	9.297176	5.555538	6.011343	4.870874	6.82381	1.28048741	high
GSM519419	2.9	1	7.433602	6.571052	5.826806	5.229203	6.266984	0.990571301	low
GSM519420	8.1	0	8.712664	5.739759	4.809248	7.418611	11.180299	1.433708165	high
GSM519421	8.1	0	7.730647	7.268619	5.736771	7.31932	8.399428	0.957834681	low
GSM519422	8	0	7.658693	4.336767	5.656756	6.997159	2.758493	1.072784685	high
GSM519424	8.4	0	7.80934	6.146046	5.906727	7.622886	9.844244	1.051289745	high
GSM519425	3.5	1	7.955652	4.607181	7.133845	6.585542	2.715063	0.889249919	low
GSM519433	6.5	0	7.332542	6.137882	6.85922	9.322374	1.927523	0.693556155	low
GSM519434	4	1	7.523742	7.889846	6.55887	8.436475	3.759473	0.691135696	low
GSM519436	5	0	6.782317	6.637271	6.006304	7.11589	8.073416	0.883055644	low
GSM519437	5.3	0	6.766834	4.724449	6.413253	8.557008	9.141055	0.942620193	low
GSM519438	4.9	0	6.536452	8.186305	6.084461	10.678373	1.120615	0.573953489	low
GSM519441	3.8	0	7.546678	7.49745	6.296445	8.525554	7.745513	0.808713092	low
GSM519723	3.39	1	7.004521	4.372208	5.541115	8.46626	5.080624	1.024951963	high
GSM519732	8.98	0	7.825589	6.010876	6.944987	5.480543	7.259397	0.93078012	low
GSM519733	7.61	0	8.07526	5.145592	6.806029	4.586638	4.308364	1.001426186	high
GSM519735	9.32	0	6.130457	7.821208	6.413952	8.592077	4.348314	0.625099834	low
GSM519737	4.07	1	8.319711	6.535598	5.808368	8.978661	4.097882	0.911493268	low
GSM519738	8.84	0	8.363697	5.644465	6.683788	3.216917	8.917333	1.171407316	high
GSM519741	4.99	1	8.776143	5.037205	5.465659	9.845551	3.963256	1.087060748	high
GSM519743	7.44	0	6.897364	7.438315	7.06851	5.52373	3.968158	0.694107121	low
GSM519745	7.37	0	8.362471	6.220105	6.513906	5.510949	10.281284	1.096650613	high
GSM519746	7.81	0	9.164041	6.158444	4.828928	9.966929	7.841772	1.231294633	high
GSM519747	7.84	0	9.509132	4.893466	7.285725	3.035037	6.479916	1.212314002	high
GSM519748	3.77	1	8.352591	6.69892	5.832908	7.845527	7.608813	1.011961637	high
GSM519749	5.28	1	9.441802	5.64238	6.588396	8.772964	11.556827	1.162971035	high
GSM519751	7.63	0	8.671356	4.9112	6.300097	6.50026	11.118135	1.270475061	high
GSM519756	6.36	0	8.680359	5.69435	6.126511	7.740617	4.125982	1.00408788	high
GSM519758	4.98	1	7.547689	6.8187	8.286509	9.436719	4.689128	0.582816814	low
GSM519759	6.2	0	8.140361	6.273598	5.549235	6.856296	5.464587	1.04960487	high
GSM519762	6.83	0	6.03098	6.510356	6.837755	9.062793	4.333361	0.635163516	low
GSM519763	7.08	0	8.102802	5.892786	6.515922	5.70147	11.023341	1.108213234	high
GSM519765	6.22	0	6.187515	7.448562	7.224434	5.031345	4.561866	0.652810703	low
GSM519769	6.17	0	8.858956	4.956689	5.380637	4.782953	4.671215	1.341920486	high
GSM519770	2.97	1	8.437344	6.415385	6.354661	6.351616	8.557626	1.040503207	high
GSM519775	4.52	0	9.188735	7.430587	5.125331	7.551224	10.827634	1.2443427	high
GSM519777	1.58	1	8.613036	5.758944	5.971519	6.120719	7.85186	1.165288076	high
GSM519783	7.93	0	8.413246	5.036296	6.492233	5.860278	3.859347	1.034694222	high
GSM519785	5.89	1	8.532082	4.712384	6.13741	5.135867	9.664635	1.318612817	high
GSM519786	11.32	1	8.241463	4.895798	7.00916	6.802603	9.217826	1.047077823	high
GSM519787	1.92	1	9.087962	4.578378	7.070137	6.147735	7.652703	1.13856469	high
GSM519788	6.38	0	4.457682	7.206132	6.594974	7.573184	5.208571	0.573670913	low
GSM519790	13.45	0	6.684727	7.631053	6.261436	7.181995	4.174931	0.713714511	low
GSM519791	7.9	0	6.723838	6.566826	6.738802	8.931818	3.710774	0.678902272	low
GSM519792	13.42	0	9.739987	5.855861	5.698552	6.433534	10.036599	1.393538045	high
GSM519793	13.27	0	7.22636	6.978863	6.832033	7.665658	4.417256	0.722076195	low
GSM519796	12.41	0	7.46073	7.285844	6.624559	7.696516	4.285303	0.739681348	low

GSM519798	7.92	1	7.755736	6.082034	6.52378	6.970217	3.562293	0.853696619	low
GSM519801	4.89	1	9.402604	6.124068	7.379824	8.225701	10.433797	0.991216781	low
GSM519802	9.78	0	10.914272	5.318443	5.899927	8.08303	10.96686	1.528838592	high
GSM519804	9.99	0	5.603543	9.348886	6.596153	6.129476	4.271989	0.557569195	low
GSM519806	3.55	1	9.431214	4.94724	4.772933	7.04976	4.53046	1.429094218	high
GSM519807	5.18	1	6.775402	6.635995	6.846842	11.374138	3.887196	0.618734985	low
GSM519808	6.19	0	9.048183	6.16102	5.45162	4.127087	7.401323	1.340667521	high
GSM602339	7.023287671	0	7.501161	5.738334	6.381364	8.451806	5.165698	0.861613498	low
GSM602346	9.07890411	0	8.975477	6.76766	6.401128	8.21446	7.401226	0.970067247	low
GSM602347	4.64630137	0	6.711301	7.894873	6.868623	5.079384	6.470744	0.727214552	low
GSM602349	6.203835616	0	8.011735	6.152485	6.402814	4.576742	4.711395	0.983940695	low
GSM602350	6.941917808	0	9.695072	5.708817	6.056645	8.750497	7.267977	1.160051206	high
GSM602351	6.858082192	0	10.620074	4.526969	5.852768	6.903198	7.050754	1.513110317	high
GSM602354	2.503561644	1	10.104058	4.099575	6.600355	4.737553	12.344827	1.62325013	high
GSM602355	3.327123288	1	8.078286	6.907684	6.087997	7.655838	5.094333	0.889183314	low
GSM602357	9.863013699	0	5.860706	6.671222	6.720423	11.207439	4.620745	0.588029746	low
GSM602358	9.863013699	0	5.632869	7.479047	6.887232	8.44463	7.997056	0.624847113	low
GSM602367	9.578630137	0	7.507602	6.470291	6.717479	5.874333	5.26632	0.84855631	low
GSM602368	4.315890411	1	9.145033	6.642183	5.437812	5.791595	12.740538	1.395509257	high
GSM602369	9.82109589	0	7.598403	5.784447	6.01907	4.496388	10.029914	1.161803683	high
GSM602372	8.016164384	1	6.983282	6.727878	6.822789	10.381487	5.823628	0.67927527	low
GSM602373	3.330410959	1	8.852895	5.346995	6.083967	9.770853	7.091043	1.054857017	high
GSM602375	9.863013699	0	9.708629	5.758246	4.401508	5.290099	12.670559	1.853284657	high
GSM602377	9.734794521	0	9.347783	4.540173	5.701659	7.486426	9.085762	1.402984562	high
GSM602381	7.024931507	1	6.792375	7.40779	6.80606	11.356005	9.47709	0.667185504	low
GSM602388	8.099178082	0	9.216424	6.040747	5.33278	6.37291	8.434142	1.328709728	high
GSM602390	7.846849315	0	7.89366	5.263344	6.721112	9.04574	10.716657	0.984243072	low
GSM602392	4.563287671	1	8.769994	4.575219	5.683291	6.569571	8.468409	1.348819032	high
GSM602393	3.002465753	1	8.038015	6.217309	5.828317	8.650705	7.107399	0.981412002	low
GSM602395	7.76630137	0	7.594627	7.718388	7.110334	5.358464	4.316516	0.731974479	low
GSM602396	1.92739726	1	10.147308	4.360598	6.215418	4.217915	4.418955	1.433096498	high
GSM602397	1.682465753	1	7.80076	6.404946	5.60035	9.015258	5.403348	0.927867582	low
GSM602399	8.012054795	0	7.941665	5.834487	6.434785	8.692314	5.725358	0.88984679	low
GSM602400	8.01369863	0	7.256407	7.41012	6.948653	10.558404	4.655486	0.629233744	low
GSM602402	7.359452055	1	8.996878	5.361597	5.435024	7.537723	5.297627	1.210565402	high
GSM602403	7.929863014	0	5.95645	7.301282	6.524452	6.305296	5.07981	0.691177814	low
GSM602405	7.60109589	0	9.096457	4.758578	5.859784	10.19404	6.443008	1.133291782	high
GSM602407	9.863013699	0	6.192824	7.365557	6.499604	9.276755	6.087436	0.65445823	low
GSM602410	8.591506849	0	7.66884	6.716497	6.682617	4.156233	5.353726	0.901533602	low
GSM602413	6.19890411	0	8.890975	4.121949	5.743749	5.018974	4.430984	1.346097422	high
GSM602416	6.123287671	0	8.546326	7.286965	6.434785	7.466126	9.063475	0.947462056	low
GSM602418	6.124931507	0	8.431517	4.795106	6.493089	5.79146	4.661359	1.077767426	high
GSM602419	6.120821918	0	7.757338	4.928539	6.267896	11.478803	4.283578	0.846694972	low
GSM602420	4.230410959	1	9.910032	6.067637	6.295189	9.829584	5.076015	1.022422708	high
GSM602421	6.043561644	0	7.18108	7.665933	7.171253	6.47197	11.39397	0.792457107	low
GSM602422	6.039452055	0	7.634533	6.154628	6.363291	6.827473	4.591003	0.882724419	low
GSM602423	6.120821918	0	7.14023	6.763461	6.624817	6.803762	4.98676	0.781534362	low
GSM602425	6.039452055	0	9.531492	4.69001	6.61121	7.051052	7.91887	1.227000065	high
GSM602427	6.039452055	0	7.865075	5.44033	5.892836	10.127224	12.038977	1.081615274	high
GSM602428	5.961369863	0	6.765257	7.310378	6.835509	9.56146	4.584066	0.634169985	low
GSM602431	5.95890411	0	8.62238	6.108396	7.171962	4.705701	4.957313	0.940866033	low
GSM602433	5.875890411	0	8.621809	3.917516	5.475435	5.169858	4.466917	1.378014952	high
GSM602434	5.870136986	0	6.823625	8.504022	6.278303	8.021585	5.271544	0.672542729	low
GSM602435	5.873424658	0	7.407266	6.4574	7.027581	3.949779	4.601734	0.845620286	low
GSM602436	5.877534247	0	9.110773	4.819271	6.025735	10.132945	10.15266	1.202942318	high
GSM602437	5.875890411	0	8.331921	6.2876	7.032631	5.455214	11.162395	1.033449879	high
GSM602439	9.780821918	0	5.932997	6.615499	6.958307	9.763865	8.903497	0.665053841	low
GSM602440	8.265205479	0	9.513406	5.681818	6.278666	8.000059	11.334059	1.244966931	high

GSM602442	5.873424658	0	7.964145	5.466802	5.90525	8.779234	4.376028	0.955910037	low
GSM602444	5.791232877	0	7.185336	5.895048	6.800771	7.207017	5.075086	0.809567637	low
GSM602445	6.122465753	0	8.359893	4.533002	6.717778	4.508999	4.364434	1.096890877	high
GSM602448	5.871780822	0	6.783381	6.855612	6.597728	6.128518	5.0739	0.771226907	low
GSM602449	5.874246575	0	7.962706	5.1516	7.155823	7.889946	7.822167	0.914689327	low
GSM602450	6.050136986	0	8.007506	6.645015	6.468569	10.212159	4.669354	0.777038469	low
GSM602452	2.091780822	1	9.445213	6.074357	5.526285	7.055011	5.483076	1.205230107	high
GSM602453	4.889589041	1	10.167679	4.83085	5.672254	4.598157	9.28742	1.64868805	high
GSM602454	9.863013699	0	8.478448	6.231933	6.55298	7.650368	4.977503	0.909697606	low
GSM602455	5.876712329	0	7.681369	5.384553	7.626766	4.479735	4.86475	0.856898179	low
GSM602456	6.703561644	0	9.38131	4.647963	6.13654	5.936789	4.99342	1.259586258	high
GSM602458	5.136164384	0	6.604243	7.201886	6.495488	9.861964	5.492886	0.667627566	low
GSM602459	5.055616438	0	7.716723	6.372805	6.043622	6.264224	4.548531	0.931580876	low
GSM602461	4.889589041	0	6.240616	7.370353	6.883399	8.927127	5.42893	0.620572322	low
GSM602463	5.477260274	0	8.357484	6.509614	6.070449	5.215169	5.462906	1.032755384	high
GSM602467	4.562465753	0	7.024275	7.756211	6.858439	8.412436	7.077385	0.688484094	low
GSM602472	4.479452055	0	8.019177	7.336661	6.570456	6.886015	5.073246	0.819440938	low
GSM602474	7.154794521	0	8.418375	6.0668	5.926036	7.490709	10.570225	1.142293777	high
GSM602475	4.398082192	0	6.523586	8.134668	6.360625	11.362772	4.600694	0.585645419	low
GSM602476	4.48109589	0	6.315747	6.926622	6.836485	10.252563	4.805084	0.61432201	low
GSM602479	4.480273973	0	8.33922	6.272166	6.751156	7.442876	4.816454	0.872970696	low
GSM602480	4.482739726	0	8.224727	7.692681	6.687115	6.142211	5.116381	0.820753026	low
GSM602483	4.396438356	0	8.113925	7.282575	6.852639	6.355983	4.841527	0.8079861	low
GSM602484	4.314246575	0	6.932334	8.313339	6.588978	9.288706	5.425411	0.635156113	low
GSM602486	4.31260274	0	8.85729	6.042484	7.224797	5.652907	4.548056	0.921775461	low
GSM602488	4.318356164	0	8.529528	6.923186	7.067814	7.886316	10.533475	0.907344549	low
GSM602489	2.839726027	1	9.715095	4.802606	6.064917	4.2531	4.842378	1.368439229	high
GSM602491	4.316712329	0	8.467856	5.477161	5.591962	4.865252	10.490903	1.37152742	high
GSM602492	4.269041096	0	9.3241	5.386946	6.110876	6.402691	11.670584	1.36075123	high
GSM602493	4.273972603	0	6.033462	8.692746	6.914542	7.852698	9.764185	0.625388949	low
GSM602494	4.315890411	0	8.361826	5.746025	5.964233	5.421503	9.203914	1.202912679	high
GSM602496	4.232054795	0	9.095209	5.464639	5.829892	6.672156	11.503444	1.359053462	high
GSM602497	4.235342466	0	8.765024	7.980215	6.397417	5.32365	4.636978	0.89537907	low
GSM602498	4.396438356	0	8.927512	5.862174	6.949052	5.508148	4.999299	0.993182209	low
GSM602502	4.232054795	0	8.558879	5.328489	6.67127	6.605946	4.743508	0.99566059	high
GSM602503	4.232054795	0	8.637134	5.463845	6.912411	9.634157	5.022738	0.873204344	low
GSM602507	4.149041096	0	7.542403	5.853268	7.934452	6.436317	5.205266	0.737333724	low
GSM602508	4.149863014	0	7.828109	5.925816	6.521948	8.701618	4.541774	0.84013337	low
GSM602509	4.234520548	0	11.040551	3.972368	3.862353	4.262772	5.503169	2.294666472	high
GSM602514	4.232054795	0	8.590578	5.48741	6.148182	4.751784	10.382627	1.283927041	high
GSM602515	3.493150685	1	7.237564	6.560504	6.695769	4.698812	9.338117	0.939367369	low
GSM602516	3.987123288	0	8.089417	6.805497	7.006748	5.09807	4.930879	0.855072527	low
GSM602517	3.988767123	0	9.081676	5.364213	6.592163	7.397573	8.691815	1.125067262	high
GSM602519	3.990410959	0	6.638352	5.875572	5.674218	7.186341	6.303309	0.927562438	low
GSM602520	3.901643836	0	8.939982	8.463687	6.579545	4.478243	4.736107	0.881372516	low
GSM1045208	8.290410959	0	8.773448	5.539726	6.500951	7.526061	4.800734	0.994733384	high
GSM1045212	6.095890411	0	9.247623	4.831383	5.78267	5.191099	7.416213	1.395744257	high
GSM1045213	0.780821918	1	7.345649	8.653694	6.388977	8.799658	10.015562	0.747084409	low
GSM1045214	5.857534247	0	8.264258	5.476668	5.962389	5.000327	5.439166	1.132990134	high
GSM1045216	6.728767123	1	9.906689	6.528966	5.971317	7.309161	9.902057	1.252728552	high
GSM1045220	7.446575342	0	8.756575	6.792397	6.773272	8.019634	4.646119	0.850473733	low
GSM1045221	2.101369863	1	9.862348	5.441935	5.236516	4.656404	12.558502	1.745625206	high
GSM1045222	3.331506849	1	8.70082	6.679931	6.901009	8.920047	5.040797	0.820682318	low
GSM1045223	6.745205479	0	7.071887	5.769864	6.189486	11.891845	8.098168	0.807747251	low
GSM1045226	8.271232877	0	6.46648	10.818947	6.708528	8.619441	4.371876	0.491211676	low
GSM1045227	0.424657534	1	10.35577	4.412832	5.347198	6.390287	8.640878	1.685243649	high
GSM1045229	1.512328767	1	7.373019	6.314456	5.904859	7.548889	9.198467	0.983129326	low
GSM1045230	7.953424658	0	8.23669	5.668415	6.710317	6.148514	5.210454	0.959491048	low

GSM1045232	4.457534247	0	8.58229	5.275813	6.889657	6.161837	4.815905	0.987702826	low
GSM1045233	4.679452055	1	8.535274	5.922588	5.948049	5.279756	4.734538	1.097750612	high
GSM1045234	6.128767123	0	9.000881	4.993116	6.207144	5.782577	5.349992	1.18616232	high
GSM1045235	2.315068493	1	9.147746	5.380506	5.742394	5.299299	10.869145	1.436083175	high
GSM1045236	8.101369863	0	5.320733	8.743447	5.904477	6.510552	8.525298	0.68164078	low
GSM1045237	2.501369863	1	8.702776	6.756411	6.093517	6.01215	8.409727	1.087272022	high
GSM1045239	2.898630137	1	8.533647	6.630152	5.655872	9.715771	11.545484	1.090747923	high
GSM1045240	7.55890411	0	7.347236	6.408903	6.775874	8.004309	5.130074	0.773295573	low
GSM1045241	7.57260274	0	7.989952	5.514461	5.300299	8.199819	4.481108	1.062466115	high
GSM1045242	3.169863014	1	9.203824	5.38894	5.228679	6.478757	9.494029	1.445670541	high
GSM1045243	1.495890411	0	6.625333	7.558477	6.401121	10.621497	5.047967	0.636601668	low
GSM1045245	6.739726027	0	6.525252	5.322018	6.69915	4.617997	7.181334	0.920936207	low
GSM1045246	3.665753425	1	8.439541	6.102172	6.105638	7.716451	5.895401	0.993330421	low
GSM1045247	7.194520548	0	8.630232	9.191052	6.124172	6.683141	4.257547	0.792647111	low
GSM1045249	6.619178082	0	7.903255	8.366205	6.360742	5.991702	4.548101	0.784671634	low
GSM1045251	7.936986301	0	5.968857	7.031432	6.28952	8.487196	6.988058	0.709342849	low
GSM1045255	7.506849315	0	8.575742	5.350471	6.614619	6.826538	4.578182	0.992495469	low
GSM1045256	3.383561644	0	8.649835	5.264672	7.304586	10.312589	4.727355	0.815873771	low
GSM1045257	5.731506849	1	9.398514	4.184021	5.625535	5.429695	4.668036	1.418693046	high
GSM1045258	6.945205479	0	8.620266	6.007228	6.015634	9.272953	5.022585	0.959976126	low
GSM1045260	6.232876712	1	7.669567	5.988142	6.878359	6.449531	5.248338	0.857651771	low
GSM1045262	5.983561644	0	8.433205	7.310657	6.789982	7.112859	4.707183	0.815471623	low
GSM1045263	2.517808219	1	8.806181	5.25083	7.505758	4.625753	11.232711	1.128907946	high
GSM1045266	2.676712329	0	7.586847	5.518612	5.829425	4.619732	5.081421	1.082809084	high
GSM1045268	5.079452055	0	8.836778	5.884801	5.825873	6.577212	5.817012	1.13192296	high
GSM1045274	6.819178082	0	10.599125	4.018958	7.363032	5.200237	5.679887	1.302276296	high
GSM1045275	3.649315068	1	9.557845	5.254354	7.839111	4.656616	4.349727	0.989121886	low
GSM1045276	5.246575342	1	8.971014	5.426925	6.142974	4.588107	4.559722	1.179553789	high
GSM1045280	4.635616438	0	5.089574	7.018553	7.0739	7.700903	8.21122	0.616460094	low
GSM1045282	1	1	7.418458	5.421442	4.654219	4.599487	9.235246	1.39298271	high
GSM1045285	5.106849315	0	8.580726	6.921246	6.962685	6.971448	7.925792	0.899271701	low
GSM1045287	1.44109589	1	7.438294	5.35773	5.826539	7.275573	6.817197	1.029623822	high
GSM1045289	7.260273973	0	6.809792	7.021443	6.868421	8.598092	4.723285	0.671486671	low
GSM1045291	7.367123288	0	7.738869	5.519054	6.611489	6.974822	4.566911	0.899426876	low
GSM1045294	4.608219178	0	6.357071	6.516546	7.077761	5.049065	10.786869	0.838120493	low
GSM1045296	1.301369863	0	9.847095	5.620432	6.042327	4.692448	11.773727	1.505770745	high
GSM1045297	6.145205479	0	8.377155	7.407541	6.526151	4.764502	4.579549	0.900759115	low
GSM1045298	6.019178082	0	8.661973	6.202694	5.947044	6.926447	4.584914	1.026160859	high
GSM1045300	7.002739726	0	8.979451	5.905174	6.784679	5.458408	10.989369	1.168561829	high
GSM1045301	6.095890411	0	4.819208	6.570646	6.279298	10.76486	4.48849	0.577076397	low
GSM1045306	7.969863014	0	7.056098	4.541537	6.084181	6.322042	4.482678	0.997755504	high
GSM1045307	8.087671233	0	7.190997	6.564673	7.0583	9.320959	7.897785	0.737219712	low
GSM1045309	5.767123288	0	7.544539	5.195764	6.138865	9.865814	4.224393	0.871878704	low
GSM1045310	2.539726027	1	8.177018	4.861553	6.119419	8.563607	4.738346	1.007449232	high
GSM1176874	4.17	0	7.85968	6.571076	6.46458	8.375701	10.204182	0.92922722	low
GSM1176878	3.67	0	8.753953	6.233509	5.955906	9.2851	4.098263	0.943244588	low
GSM1176879	3.67	0	8.918702	6.424507	6.236683	7.164906	3.945835	0.970630774	low
GSM1176883	2.08	0	11.297196	3.671363	5.8919	5.226422	4.731044	1.721311472	high
GSM1176884	3.17	0	8.05637	6.61426	6.372165	7.992617	5.566098	0.871613021	low
GSM1176885	5.58	0	8.905506	5.394768	6.021597	4.337136	11.972081	1.426447209	high
GSM1176887	1.25	0	6.621989	5.990124	5.638318	7.966797	8.823267	0.95193881	low
GSM1176888	5.5	0	7.488609	4.882878	6.010278	10.023022	7.477473	0.968972488	low
GSM1176890	1.75	0	9.029356	6.280893	4.834482	4.413876	11.185287	1.561239942	high
GSM1176893	5.67	0	6.622794	5.784073	5.992474	4.797136	4.549093	0.927862318	low
GSM1176896	4	0	7.720366	6.24464	5.607986	5.727766	9.520745	1.140625751	high
GSM1176897	4.75	0	8.785438	5.73783	5.726042	5.034421	4.422441	1.178212535	high
GSM1176902	4.92	0	9.579276	4.288482	6.613731	6.013828	5.326629	1.240392194	high
GSM1176903	4.92	0	10.209965	4.574382	5.954684	2.489685	11.570371	1.83322364	high

GSM1176904	5.58	0	9.493091	4.625304	6.221297	7.534245	4.774101	1.189182038	high
GSM1176906	5	0	8.842477	4.842143	5.989031	6.102095	8.102157	1.283707228	high
GSM1176908	4.75	0	6.422638	9.390789	6.239759	8.758886	8.138064	0.632671041	low
GSM1176909	4.83	0	9.101068	6.787116	6.444498	5.571906	7.167976	1.058321653	high
GSM1176910	1.58	1	6.655845	5.927478	5.743108	11.475451	8.295967	0.831458468	low
GSM1176913	4.17	0	7.960404	6.084225	6.618292	9.219027	5.299384	0.829247112	low
GSM1176914	4.08	0	6.682012	7.146337	6.43909	6.487372	4.128336	0.73854188	low
GSM1176916	1.08	1	9.951497	5.563332	6.739785	8.987948	6.918005	1.075024287	high
GSM1176920	1.17	1	9.421506	4.469716	6.783805	8.481332	5.534808	1.088641891	high
GSM1176923	2.58	0	7.58688	5.822986	6.393049	7.234466	4.718684	0.888212715	low
GSM1176924	4.5	0	7.784598	7.328761	7.116915	9.30213	5.929297	0.698362274	low
GSM1176926	3.42	1	7.875652	6.790626	6.740722	8.246597	11.182906	0.903762704	low
GSM1176928	2.83	0	7.535014	8.279711	7.119282	11.008942	4.708229	0.581943744	low
GSM1176929	4.58	0	8.776813	5.1125888	5.6134594	3.9215185	4.1708822	1.29513774	high
GSM1176932	2.92	0	7.553087	7.320451	6.723436	8.896802	4.504145	0.708805164	low
GSM1176934	0.5	0	9.105891	5.391872	5.730218	4.627532	4.221744	1.258605542	high
GSM1176935	4.17	0	7.635811	6.961662	6.636192	9.286718	4.18771	0.728804682	low
GSM1176937	4.5	0	8.214693	6.775863	6.780693	4.14577	8.842292	1.010248488	high
GSM1176938	4.42	0	7.550099	6.190299	7.126277	5.692244	4.766883	0.817605681	low
GSM1176941	3	0	6.137349	5.945031	6.574958	5.025342	4.328822	0.79551528	low
GSM1176942	2.92	0	7.616763	6.42148	6.736109	7.929993	9.536164	0.883486803	low
GSM1176943	4.67	0	8.945623	6.945432	7.154911	4.737156	3.719267	0.885837998	low
GSM1176944	2.5	0	6.600535	6.503977	6.311544	9.998491	4.345085	0.701046309	low
GSM1176946	4.25	0	8.16309	7.21686	7.12035	3.844258	5.137295	0.85996869	low
GSM1176948	3.25	0	8.589606	6.119536	7.619965	4.958567	7.462261	0.923510387	low
GSM1176951	4.33	0	6.451746	6.522796	6.1863	7.641962	9.312533	0.850362225	low
GSM1176952	4.25	0	7.96161	6.099704	6.406376	9.071275	4.710473	0.846256928	low
MB-0346	1.679452055	1	9.78056	4.356086	4.770822	4.094145	8.054732	1.849771309	high
MB-0503	8.320547942	0	7.080915	7.449024	5.785588	9.053385	5.02475	0.770361555	low
MB-0201	10.33150685	0	8.506017	6.77286	5.166398	4.618776	9.379825	1.300066132	high
MB-0316	15.03287671	0	9.592184	4.809995	6.523231	4.974549	10.506578	1.406924657	high
MB-0189	0.745205479	1	7.536437	6.960699	6.368612	8.658531	5.009784	0.7798984	low
MB-0605	9.419178082	0	6.129535	8.814321	7.097062	9.350605	4.837503	0.518410818	low
MB-0258	7.079452055	0	7.399199	6.967069	6.031925	10.308359	4.266007	0.750772387	low
MB-0420	6.306849315	0	9.183324	5.136048	5.250077	4.56099	10.69142	1.606569575	high
MB-0155	4.915068493	0	5.493587	6.844538	5.845798	11.305525	4.922578	0.635745202	low
MB-0117	0.197260274	0	7.789414	6.564201	5.90835	10.467897	4.574891	0.819727102	low
MB-0906	5.249315068	1	8.27545	4.752648	5.058949	8.989283	9.379825	1.304779999	high
MB-0249	15.47945205	0	8.725113	4.498692	4.461798	8.608573	12.832645	1.655752159	high
MB-0497	6.057534247	0	7.296901	5.447306	5.772001	7.365181	5.019704	0.972871724	low
MB-0434	3.739726027	1	9.159651	5.337028	5.277746	5.649622	8.428228	1.440175372	high
MB-0653	2.038356164	1	9.546385	4.318417	6.174849	4.387226	6.654443	1.42765823	high
MB-0384	5.021917808	1	8.213843	6.988028	6.724749	6.607778	6.723827	0.879495624	low
MB-0637	6.630136986	0	9.498496	4.980106	5.95467	6.417291	5.199684	1.259550534	high
MB-0292	4.063013699	1	9.553908	5.875733	5.21488	4.575122	8.532456	1.503994893	high
MB-0140	12.15890411	0	7.929659	8.086016	6.721622	6.774217	5.445592	0.759749816	low
MB-0598	7.345205479	1	9.457324	5.719264	5.959279	7.78538	11.74503	1.31295406	high
MB-0453	3.545205479	1	9.01624	4.593345	6.536643	7.262846	12.375858	1.306168276	high
MB-0579	7.624657534	1	7.903676	7.750443	5.956394	5.028065	5.653905	0.92242563	low
MB-0310	15.21917808	0	7.441301	7.636879	6.325941	10.544203	4.742926	0.688832021	low
MB-0614	9.808219175	0	8.889211	6.984799	5.85065	8.152508	5.821437	0.988296124	low
MB-0144	12.49863014	1	6.964708	7.448369	6.253692	7.613177	8.624693	0.811967831	low
MB-0596	8.802739726	1	7.005745	7.365427	6.713583	9.543468	4.377094	0.654796841	low
MB-0164	0.890410959	0	9.198929	6.81673	5.938181	4.492503	9.526907	1.251895564	high
MB-0215	10.04383562	0	8.38661	7.079483	6.43414	5.700662	5.69101	0.931296845	low
MB-0146	10.04109589	1	9.038833	8.128186	6.343694	5.532798	4.470229	0.906003237	low
MB-0516	9.408219181	0	9.658829	4.809995	6.373627	4.071662	9.565862	1.458715002	high
MB-0272	10.02739726	1	8.681111	8.834943	6.453212	6.936844	9.396598	0.87112255	low

MB-0494	3.010958904	1	9.331217	4.820363	5.860024	4.360507	9.187109	1.490788852	high
MB-0306	3.531506849	1	7.831177	5.715916	6.502195	7.72505	9.597869	0.992496381	low
MB-0198	11.89863014	0	8.735279	6.597883	6.926362	4.833004	5.083561	0.946820719	low
MB-0607	7.219178082	0	8.552161	6.295382	6.30818	8.447623	5.409181	0.928064869	low
MB-0631	3.898630137	1	7.959694	6.841008	6.22448	9.396704	5.011421	0.816491119	low
MB-0363	7.389041096	1	9.386121	5.463112	5.750827	6.179642	4.918895	1.237195065	high
MB-0427	9.542465753	0	8.088858	7.187619	6.628722	8.222539	5.146162	0.793027317	low
MB-0519	9.120547948	0	8.433362	7.00854	5.770885	9.523519	7.621348	0.950262793	low
MB-0348	10.08493151	1	7.660835	7.67103	6.474	4.665799	5.487011	0.84985257	low
MB-0261	7.416438356	1	7.81873	7.368595	6.768506	6.858084	6.657227	0.809041892	low
MB-0576	2.547945205	1	9.298848	7.774191	6.308996	7.27923	6.770006	0.953873671	low
MB-0385	2.994520548	1	9.005146	6.279268	6.59034	8.369741	5.052998	0.927759944	low
MB-0270	27.70136986	0	9.04887	5.577579	5.769046	7.156671	7.045228	1.202913579	high
MB-0527	9.027397258	1	5.2118	7.796604	5.457722	10.874867	4.363241	0.608114331	low
MB-0273	15.33150685	0	7.34324	6.858288	5.935109	8.42718	5.040568	0.827084839	low
MB-0050	6.191780822	0	6.465587	7.139379	6.100801	7.2046	6.291075	0.778444106	low
MB-0460	9.369863014	1	7.514146	7.523302	5.622451	11.397868	4.586925	0.74842011	low
MB-0454	3.849315068	1	6.769745	8.136115	6.411779	4.504599	5.79703	0.768456228	low
MB-0392	3.134246575	1	9.206046	7.285557	5.524295	8.507085	4.979088	1.010643518	high
MB-0336	14.02465753	1	9.315616	5.581184	6.139272	9.779634	5.188546	1.029031093	high
MB-0176	9.323287668	0	8.949066	6.952798	5.965385	6.630761	7.189637	1.063966162	high
MB-0429	6.120547945	1	8.083356	5.184417	5.720297	7.865072	5.251098	1.066686184	high
MB-0397	4.704109589	0	5.711921	5.532679	6.49144	7.568734	4.361696	0.733445872	low
MB-0571	12.31780822	0	8.353106	5.563851	6.568238	8.123706	5.25543	0.935478965	low
MB-0426	10.77534247	0	7.705132	7.966439	6.619068	5.771243	5.273631	0.784046915	low
MB-0135	9.586301367	0	8.61208	7.325194	6.096465	5.617759	5.155949	0.970203874	low
MB-0112	3.219178082	1	7.611524	7.631086	6.515049	10.043652	12.047182	0.818805251	low
MB-0644	7.668493151	0	7.202659	5.881573	6.353224	11.566961	4.754887	0.742479044	low
MB-0568	14.9150685	0	7.452347	8.282749	6.014403	8.479852	6.667064	0.766957823	low
MB-0636	2.682191781	1	8.145927	5.345275	5.994728	10.467897	4.47947	0.91889295	low
MB-0145	12.1369863	0	4.934515	6.168282	5.93354	11.207482	5.511346	0.637405856	low
MB-0422	2.778082192	1	7.515694	7.210528	5.767558	7.076287	8.560862	0.949417749	low
MB-0483	6.120547945	1	9.271566	5.642983	5.743513	6.786855	4.544509	1.173788459	high
MB-0486	7.394520548	0	8.075878	8.276299	5.842836	7.959141	5.389616	0.825105086	low
MB-0257	7.539726027	1	7.777263	6.245895	5.706918	9.141155	5.173862	0.914607879	low
MB-0345	5.97260274	0	6.603007	6.700503	5.877352	9.360812	5.001123	0.761211163	low
MB-0375	11.76712329	0	8.416837	7.101928	6.24354	4.759867	6.263398	1.001147363	high
MB-0419	8.57260274	1	7.199449	6.659824	6.472724	4.590481	5.488069	0.88129654	low
MB-0311	12.42465754	1	8.254352	4.802214	6.48479	4.961332	12.445875	1.300735116	high
MB-0324	11.9150685	1	8.059779	5.05113	6.824647	8.156625	5.02717	0.906850506	low
MB-0035	2.980821918	1	7.896325	4.803871	6.896919	5.70813	12.617038	1.16099326	high
MB-0119	7.879452055	1	8.803705	5.557047	6.682538	7.482305	5.007221	0.977131184	low
MB-0650	2.098630137	0	6.897747	7.092932	6.352398	10.321726	6.321719	0.709209403	low
MB-0600	9.123287671	0	8.587731	6.208163	6.299986	6.505225	4.600993	0.983041629	low
MB-0173	0.309589041	0	8.518273	6.01529	5.650303	5.686515	13.040475	1.352054136	high
MB-0131	5.476712329	1	7.39579	8.558635	6.311496	4.29086	4.277243	0.779623708	low
MB-0206	11.60273973	0	10.183928	4.919456	5.697154	7.104406	9.924269	1.525499655	high
MB-0315	15.30410959	0	7.665194	7.835746	6.144041	5.313007	5.099807	0.853137133	low
MB-0361	1.238356165	1	9.51846	6.167468	5.829389	8.785984	12.181421	1.269334	high
MB-0370	7.684931507	1	9.688229	5.039797	5.656751	6.374426	4.333496	1.30754328	high
MB-0431	6.997260274	0	6.750076	6.49517	6.126923	9.778193	4.893457	0.745063208	low
MB-0603	10.02465754	0	7.140022	6.977268	6.490167	5.092644	5.93286	0.847332695	low
MB-0411	11.43835617	0	6.654565	7.201114	5.92096	4.844172	4.84649	0.847044816	low
MB-0359	1.775342466	0	7.510054	7.204667	5.99048	8.862145	6.01869	0.818274486	low
MB-0202	10.55068493	0	8.34044	5.666448	5.315529	4.303004	5.084078	1.250810205	high
MB-0538	1.980821918	1	8.028491	7.032511	6.063952	8.793023	5.528844	0.855173178	low
MB-0148	0.145205479	0	8.202959	5.116135	6.150804	6.996526	8.832664	1.139954188	high
MB-0532	11.76438356	1	9.373019	5.012381	5.910957	6.853599	4.699474	1.217067874	high

MB-0491	10.33972603	0	8.348323	7.939064	6.355696	7.010844	4.702372	0.821803913	low
MB-0404	5.219178082	0	8.430826	6.41392	5.640793	7.925109	5.251416	1.012373505	high
MB-0243	12.30958904	0	8.06784	7.485721	6.142059	7.728705	5.650154	0.851701602	low
MB-0312	13.93972603	1	8.395504	6.488418	6.090608	4.989902	4.581897	1.022412474	high
MB-0060	11.57808219	0	9.441729	4.396768	7.33541	4.199578	4.474302	1.142362627	high
MB-0649	5.049315068	0	7.535411	7.047408	7.131426	8.082544	5.594244	0.718626955	low
MB-0643	1.621917808	1	9.652588	5.282428	6.139272	5.048597	11.773395	1.47868186	high
MB-0581	0.646575342	1	8.593275	4.534449	6.684313	4.217381	10.534051	1.309286555	high
MB-0383	7.046575342	1	8.892607	4.966425	6.077277	11.364101	5.086937	0.98911027	low
MB-0142	1.019178082	0	5.314039	4.982274	5.687453	8.957115	5.335747	0.805230914	low
MB-0613	4.010958904	0	8.308376	5.358324	5.738044	4.794935	7.873143	1.261119922	high
MB-0313	15.18630137	0	10.180776	4.780371	6.424418	6.930253	4.639275	1.24110877	high
MB-0286	15.83013699	0	5.313289	7.684532	6.359863	11.170094	5.643745	0.556128587	low
MB-0589	10.33972603	0	8.462	6.388456	6.302464	7.630094	4.845615	0.927417153	low
MB-0406	7.964383562	1	9.04887	6.878769	6.074171	6.074182	12.422047	1.220707886	high
MB-0452	5.161643836	0	8.74956	6.144717	6.834838	5.98875	4.214393	0.938422971	low
MB-0573	13.41369863	1	7.422383	7.462317	5.98778	10.392824	4.520297	0.730895374	low
MB-0656	1.564383561	1	9.726732	5.749141	5.663484	9.228926	7.482345	1.212578626	high
MB-0597	9.304109589	1	6.724917	7.790976	6.518491	11.057461	9.665171	0.679716795	low
MB-0412	11.19178082	0	10.577192	5.138661	6.26268	8.467494	4.750369	1.222309176	high
MB-0438	11.10958904	0	8.448424	6.039512	6.209888	7.014949	9.410167	1.09146045	high
MB-0162	4.583561644	0	7.398241	8.010797	7.175601	6.306206	8.574819	0.74268806	low
MB-0593	2.849315068	1	8.317686	7.433147	6.103946	4.679144	12.227121	1.131868369	high
MB-0301	10.08493151	1	7.4273	8.151057	7.022412	7.819623	5.042271	0.660648553	low
MB-0106	7.01369863	0	5.690733	6.901562	6.828355	4.954498	4.805863	0.691965102	low
MB-0474	9.131506849	1	8.769024	5.079466	6.136898	5.764377	9.517206	1.28004194	high
MB-0394	5.279452055	0	7.621575	8.326851	6.399992	9.889672	7.282881	0.71204053	low
MB-0247	15.25479452	0	6.913794	7.571513	6.516752	9.612812	4.929766	0.663494288	low
MB-0354	0.953424658	1	8.247629	6.148052	6.042866	7.434514	9.990432	1.086034446	high
MB-0608	5.257534247	1	8.473137	4.995747	7.271526	4.424409	3.808829	0.980035159	low
MB-0559	13.29589041	0	7.038075	5.598569	6.976189	7.205669	5.675417	0.807689506	low
MB-0893	14.43561644	0	6.886499	5.268303	6.745451	12.470725	4.57974	0.690622443	low
MB-0514	1.101369863	0	8.359467	5.368927	6.918414	4.330494	5.308446	1.027357482	high
MB-0481	2.991780822	1	8.925717	4.784237	5.864941	9.773914	11.264256	1.257950102	high
MB-0302	6.920547945	1	7.477945	8.108429	5.906844	8.39485	8.023279	0.818246185	low
MB-0121	12.50958904	0	7.55786	8.26649	6.531856	4.784095	5.037394	0.785904189	low
MB-0364	14.51506849	0	6.212231	7.748932	6.4481	8.282147	4.305588	0.636449259	low
MB-0232	17.06575342	0	6.918831	6.975689	6.989495	5.120264	6.464143	0.78187863	low
MB-0884	7.698630137	1	9.832365	5.280063	7.041699	4.628598	8.64767	1.251670597	high
MB-0123	9.389041093	1	10.039231	6.445902	5.652076	7.320266	5.129271	1.198016261	high
MB-0504	10.72328767	0	7.555249	8.558635	6.390772	7.943035	5.213624	0.70828485	low
MB-0475	10.74246575	0	8.835861	6.429867	6.263909	7.283019	9.209385	1.076413489	high
MB-0482	2.043835617	1	10.831413	5.799284	5.791881	11.529195	12.537747	1.371208211	high
MB-0291	3.221917808	1	8.935099	5.713142	5.573078	9.781037	5.274235	1.065447355	high
MB-0465	2.350684932	1	8.780542	5.987187	5.268193	5.099101	10.99964	1.427711005	high
MB-0630	7.134246575	0	9.061606	4.668374	6.471463	5.530211	3.818384	1.147994418	high
MB-0036	10.85205479	1	7.903058	7.787779	5.794464	7.977917	4.488317	0.83071915	low
MB-0632	4.643835616	0	7.528638	6.524264	6.90435	9.657555	5.69828	0.734417057	low
MB-0507	1.956164384	0	7.496771	8.362131	6.908541	8.83076	4.728367	0.638358035	low
MB-0287	7.78630137	1	8.151483	7.13823	6.646315	7.782952	4.75802	0.803543397	low
MB-4633	26.15342466	0	6.92558	6.776796	6.742745	9.704644	4.354126	0.673221839	low
MB-4618	21.97808219	0	8.405377	4.82688	6.443016	4.566385	4.692768	1.125720979	high
MB-4641	12.21095891	1	6.62417	6.649123	6.986171	7.779351	11.624306	0.802565894	low
MB-4634	9.008219178	1	7.800533	4.870745	6.170422	5.468318	10.880221	1.228414657	high
MB-4665	21.38630137	0	7.571512	6.57986	7.309068	4.126923	5.00909	0.820702222	low
MB-4666	9.169863016	1	7.901231	5.007772	6.752681	7.239765	5.057761	0.933892915	low
MB-4640	16.48767123	0	9.096545	5.590944	6.098026	7.086647	10.819686	1.258683585	high
MB-4671	2.852054795	1	8.587731	4.18577	7.028673	5.234929	5.27268	1.09853055	high

MB-4667	24.2739726	1	8.21167	4.364103	6.520212	7.881382	8.823222	1.114315484	high
MB-4721	1.950684931	1	6.998434	6.589526	6.705129	6.930741	7.369877	0.812113282	low
MB-4718	16.30136986	1	7.055968	6.749836	6.204234	10.343235	4.758945	0.728184811	low
MB-4716	6.257534247	1	8.422601	6.167066	6.566958	9.509965	5.220742	0.857873023	low
MB-4717	3.526027397	1	10.171405	5.000379	5.876218	4.448398	12.220307	1.699166363	high
MB-4752	10.29589041	1	7.546393	6.82932	5.768656	6.332775	4.965247	0.926227349	low
MB-5169	4.271232877	1	8.856632	5.21271	6.120992	6.37579	4.75644	1.126113746	high
MB-4800	9.117808216	1	6.857177	5.482008	6.201012	4.53792	8.304347	1.036282002	high
MB-4745	3.506849315	1	8.984753	4.741193	5.152806	5.159607	11.763908	1.654704499	high
MB-4825	17.62465753	1	8.028491	5.12735	6.205441	6.967607	5.100337	1.021988165	high
MB-4814	6.038356164	1	8.918782	5.27602	6.55872	9.373453	6.432737	0.996622865	high
MB-4757	2.252054795	1	10.003441	5.617785	5.559653	6.431955	7.936408	1.415573774	high
MB-4694	16.29863014	0	10.116774	4.597606	6.405871	5.966406	4.550589	1.292529905	high
MB-4685	3.55890411	1	9.984385	4.734272	5.494121	5.703408	4.719814	1.453989338	high
MB-4712	20.50958904	1	8.537778	5.009018	5.575499	6.32379	6.266867	1.241920283	high
MB-4672	18.15890411	1	9.459097	4.83457	6.648073	4.883777	4.557283	1.193529293	high
MB-4704	5.260273973	1	7.257109	6.577542	6.353224	5.967648	5.041052	0.857644156	low
MB-4651	2.490410959	1	9.187489	5.478181	6.919346	6.901673	4.606226	0.996806056	high
MB-4649	1.383561644	1	8.147288	4.637535	7.309068	6.425524	5.72353	0.949099659	low
MB-4642	23.48219178	1	7.878722	4.955186	6.475697	6.968102	5.203213	0.984971058	low
MB-4648	13.85205479	1	8.362626	6.21232	7.232793	5.867686	4.9719	0.868568811	low
MB-5197	18.39726027	1	8.353895	5.637475	6.722509	8.685093	5.022587	0.888695916	low
MB-5201	7.969863014	1	6.702123	6.060797	6.250025	7.860711	9.405652	0.890121076	low
MB-5189	9.202739729	1	7.500351	6.301792	6.429083	10.149084	4.635918	0.765095154	low
MB-5281	6.517808219	0	6.992857	6.018032	4.959293	6.396238	5.799761	1.065590471	high
MB-5172	15.14520548	0	9.303515	4.92926	6.295909	9.076304	11.721793	1.255575966	high
MB-5185	6.367123288	1	8.014178	5.476759	6.694483	4.190684	5.328637	1.022263801	high
MB-5014	17.5369863	0	8.543127	7.335244	6.237445	6.059588	4.311769	0.912643025	low
MB-5017	11.91232876	0	8.757733	5.345616	5.277143	5.473843	4.90255	1.285514657	high
MB-4982	1.575342466	1	8.902725	4.436592	5.734753	6.588608	11.043919	1.452635974	high
MB-4986	6.55890411	1	9.640256	5.516661	6.22852	7.267241	6.670328	1.184775581	high
MB-5327	13.44109589	0	8.794197	4.971892	5.448132	5.390245	12.187693	1.533854994	high
MB-5341	20.61643835	0	8.002591	5.557403	6.460857	4.692078	5.337363	1.031598002	high
MB-5323	18.58082192	0	8.791043	6.430764	5.989698	8.753815	8.269442	1.03831866	high
MB-5287	10.19178082	1	7.250182	6.045115	6.829733	9.161664	5.958148	0.766739275	low
MB-5324	15.62465753	1	7.054199	6.055978	6.597319	8.237419	5.300836	0.789144418	low
MB-4682	11.46849315	1	8.459459	7.374958	6.298743	5.021499	5.130644	0.943600999	low
MB-4710	4.739726027	0	6.264301	5.514879	6.822814	10.255536	5.176157	0.688744328	low
MB-4686	3.980821918	1	7.931525	6.34889	5.909458	7.880754	8.198366	0.999769533	high
MB-4702	24.72876713	0	9.648383	4.882639	6.432443	5.159302	4.019725	1.221954997	high
MB-4862	15.39452055	0	10.183928	5.261279	6.101191	5.633233	4.637502	1.307067464	high
MB-4894	19.10136986	0	9.913789	5.027285	6.875897	8.625602	5.156229	1.064979507	high
MB-4898	9.690410959	1	7.42386	5.99824	6.067106	6.104308	5.03203	0.944422966	low
MB-4968	6.468493151	0	7.741277	8.190657	6.749544	6.468694	4.54694	0.729636733	low
MB-5052	2.84109589	1	8.238746	5.861075	5.42152	6.512189	7.551148	1.181300995	high
MB-5049	20.98356164	0	8.861081	5.859158	5.808238	6.546757	11.167954	1.288313735	high
MB-5041	14.29589041	0	8.65114	5.043265	6.35197	5.154682	4.774194	1.128082416	high
MB-5044	11.46027397	0	8.202233	5.735283	6.449815	6.409042	4.996463	0.973683523	low
MB-4171	15.18082192	1	8.862183	5.295521	6.857936	7.449664	4.473159	0.967487274	low
MB-5053	12.37808219	1	6.862545	6.741627	6.120992	7.367941	11.528818	0.931732224	low
MB-5074	18.21369863	1	7.804021	5.714069	5.65352	7.916878	5.858032	1.018350597	high
MB-5119	4.912328767	0	7.144473	5.925723	5.823832	7.147802	4.247494	0.907757933	low
MB-5114	1.263013699	1	9.00389	6.582168	5.549658	6.387559	7.865277	1.194736229	high
MB-4230	9.501369863	1	8.660734	5.499673	6.193337	9.093301	12.383363	1.162379708	high
MB-4154	4.073972603	1	9.921397	4.666596	5.904929	6.017264	11.43555	1.580537243	high
MB-5115	6.361643836	0	10.595418	4.391101	5.569959	4.282503	10.282659	1.863986139	high
MB-4737	10.04383562	0	9.895976	5.445544	5.396832	4.891024	4.728367	1.421564528	high
MB-4730	10.79178082	1	5.545434	5.995484	6.428231	6.45491	4.642524	0.733828831	low

MB-4733	1.621917808	1	9.698898	6.283463	4.957323	5.168603	12.012699	1.626193173	high
MB-4758	17.41917808	0	9.005146	5.426698	5.377964	4.376616	10.754358	1.528083362	high
MB-5256	8.882191784	1	7.867177	6.061575	6.618199	10.066818	6.878104	0.829624698	low
MB-5273	5.002739726	1	9.109896	7.094622	5.987399	5.290382	4.593408	1.050254008	high
MB-5238	3.62739726	1	8.23141	5.853798	6.052991	8.523558	8.04961	1.022196589	high
MB-5233	17.18082192	1	8.721016	4.517354	6.754038	4.249406	12.254136	1.365226447	high
MB-5253	8.389041099	1	6.476072	6.345436	6.457865	9.805473	4.663112	0.696349855	low
MB-5260	16.6109589	1	8.562951	4.456884	5.909829	9.989976	4.978628	1.0655032	high
MB-4998	5.389041096	1	8.309945	6.354583	6.259427	9.074281	4.878743	0.879319058	low
MB-4993	6.183561644	1	8.425887	4.638757	5.066278	5.13136	10.698624	1.562938303	high
MB-5001	6.723287671	1	8.16067	6.440536	5.534594	9.105331	4.963719	0.954437441	low
MB-5084	16.28219178	1	6.415398	6.912436	5.958118	9.526018	10.467117	0.818756879	low
MB-4969	16.28219178	1	7.50597	5.505671	6.964399	4.974801	5.758468	0.919845254	low
MB-4999	15.44657534	0	7.566239	5.130228	6.501331	6.562704	4.779922	0.94313633	low
MB-5011	6.616438356	1	7.002758	6.247164	6.642786	8.811372	4.881157	0.747094914	low
MB-4599	15.71232877	0	8.128498	5.265601	6.975248	5.239506	7.994708	1.036145676	high
MB-4623	24.00273972	1	8.946736	6.294961	6.490585	6.299048	5.376062	1.008462971	high
MB-4869	2.408219178	1	8.084734	6.5518	6.158812	4.22133	5.038849	1.01373042	high
MB-4878	1.846575343	1	8.775299	5.545915	5.868324	8.326437	5.164992	1.0673737	high
MB-4851	7.419178082	1	8.544053	6.8695	5.973053	6.222599	7.135697	1.041489374	high
MB-4937	6.347945205	1	9.507539	5.164873	6.268412	4.541997	10.940072	1.441902363	high
MB-4934	5.77260274	0	8.872253	5.502145	5.937806	6.041196	4.961532	1.149715108	high
MB-4899	22.6630137	0	8.219653	6.981033	6.599476	8.311159	4.980203	0.813893403	low
MB-4912	4.109589041	1	8.489467	6.306903	6.904809	7.144688	4.744417	0.871608114	low
MB-4933	22.43013699	0	9.846799	5.308381	5.804852	6.989884	5.093999	1.269527145	high
MB-5338	4.246575342	1	10.113775	4.32659	6.030345	7.308647	4.888895	1.340712025	high
MB-5195	16.18082192	1	8.609229	6.119178	6.047523	6.201042	5.517012	1.060074556	high
MB-5232	17.35890411	1	8.207337	5.110014	5.962325	8.575713	13.288361	1.230192727	high
MB-5160	7.210958904	1	9.038833	6.149702	6.761254	6.764502	9.883326	1.080413028	high
MB-5124	10.20821918	1	8.708993	6.218207	6.594688	6.197553	4.773913	0.967014759	low
MB-4855	3.408219178	1	7.32088	6.055174	5.849171	4.930808	4.622433	0.988969937	low
MB-4173	13.17808219	1	6.961736	6.279268	6.557857	5.245697	4.528611	0.83906201	low
MB-5145	13.08767123	1	7.8288	5.460639	5.701128	6.269541	9.333595	1.181995065	high
MB-4956	5.695890411	1	9.89344	4.791388	5.215167	7.504534	13.186327	1.703907731	high
MB-4952	15.15616438	0	8.892607	4.591714	5.025341	9.247233	11.061961	1.451151064	high
MB-5266	6.873972603	1	10.633039	4.108619	5.890501	4.767548	7.478497	1.687348199	high
MB-5284	5.369863014	1	6.102372	5.67525	7.136845	9.2581	7.807664	0.703168943	low
MB-4148	5.180821918	1	9.223249	4.93346	6.54357	5.357281	4.926042	1.16636164	high
MB-5078	7.15890411	1	9.074482	4.458633	5.749348	5.723621	10.724952	1.503355322	high
MB-5088	16.38630137	1	8.262563	6.661721	5.89924	3.990824	4.370017	1.052666778	high
MB-5107	6.791780822	1	9.502174	4.210177	6.486281	4.874957	11.877352	1.520026089	high
MB-5066	7.783561644	0	6.954534	6.376124	6.185261	6.25978	5.083562	0.858756052	low
MB-5068	15	0	6.193275	5.857644	6.797993	4.447367	4.131169	0.791905712	low
MB-5070	17.58082192	0	8.262563	5.48831	5.458374	5.415089	4.29905	1.167773384	high
MB-5059	21.17534246	0	6.853117	6.730993	6.434987	6.728729	4.455881	0.775304245	low
MB-4771	24.43013698	0	8.473137	7.519875	6.082952	6.550993	9.787327	1.017580486	high
MB-4739	13.28767124	1	7.062507	6.401124	6.252464	8.114224	4.221937	0.79096182	low
MB-4778	16.94246575	1	7.129308	7.356565	6.396618	9.519806	4.689437	0.698867071	low
MB-4763	8.391780822	0	8.53599	4.93346	5.04819	5.444294	8.620806	1.461326757	high
MB-4779	8.347945208	0	5.919421	7.662774	6.454041	7.34649	4.745923	0.648466187	low
MB-4787	6.663013699	1	8.649207	5.11995	6.30818	5.782166	6.064852	1.137735903	high
MB-4767	3.873972603	1	8.213843	4.885252	5.557597	6.306206	5.427462	1.196039509	high
MB-5020	6.97260274	1	7.729386	6.838982	5.872842	4.84266	8.298705	1.052206081	high
MB-5019	3.106849315	1	9.640256	5.978136	5.58631	5.666901	12.375858	1.502675529	high
MB-5015	20.7369863	1	7.821654	5.834186	6.552207	5.854895	8.838617	1.020514028	high
MB-5398	17.50684932	0	6.866658	6.603425	7.381295	8.975063	4.245844	0.633736034	low
MB-5291	12.61917808	0	9.684045	7.240758	7.165166	4.559077	4.925437	0.959881865	low
MB-5224	8.169863014	1	7.375208	7.08506	6.534897	9.877594	4.09365	0.698704494	low

MB-5369	3.230136986	1	7.437298	5.5801	6.949346	4.816975	5.354676	0.90689579	low
MB-5404	6.142465753	1	7.58905	6.343269	7.010458	6.859078	11.080252	0.91534221	low
MB-5395	13.57534247	0	7.630782	6.078535	6.749544	6.452591	4.804481	0.855284628	low
MB-5365	1.893150685	1	7.132859	7.95049	6.674948	8.133263	5.671293	0.687550798	low
MB-5389	10.25479452	1	7.934048	6.439205	7.150729	5.32253	4.30116	0.830844344	low
MB-5361	1.263013699	1	7.973026	4.471966	5.756594	6.304883	5.552978	1.176545553	high
MB-5392	19.50136987	0	8.081307	5.938172	6.061574	7.037701	10.277186	1.104932623	high
MB-5138	3.04109589	1	9.906237	4.902619	6.817243	4.750766	6.5313	1.271532961	high
MB-4127	21.21917808	1	9.514889	4.138512	6.685631	4.7427	6.35786	1.318348063	high
MB-5058	14.69041096	1	7.584775	5.303274	5.59541	5.758784	11.659099	1.271951102	high
MB-5027	15.60547945	0	7.140022	7.518462	6.234613	8.29664	5.475732	0.749460349	low
MB-5060	12.3369863	1	7.899389	6.134389	6.19293	8.238122	5.108379	0.896914859	low
MB-5101	2.821917808	1	9.217537	4.648629	5.805598	6.347254	4.699474	1.272815347	high
MB-5272	16.59726027	1	9.074482	5.619619	6.485418	5.258836	5.606388	1.120154708	high
MB-5264	16.38082192	0	6.649887	6.827792	5.985074	8.813115	5.760029	0.77269337	low
MB-5490	4.208219178	1	7.978813	7.353418	7.065586	9.243963	5.516656	0.710059608	low
MB-5348	13.94520548	0	10.168289	4.973696	5.186311	4.62446	11.309149	1.826730287	high
MB-5429	2.523287671	1	6.357156	6.821218	6.772609	9.648294	6.48861	0.665144161	low
MB-5489	17.83287672	0	7.035493	5.603255	7.305979	9.723847	5.1988	0.701020222	low
MB-5417	8.800000003	0	9.395979	4.723886	6.773512	7.493679	10.640311	1.237566895	high
MB-5409	16.2849315	0	9.43312	5.352131	5.702926	4.226095	12.076885	1.584584469	high
MB-5299	1.37260274	1	10.409729	4.584744	6.242725	4.998866	9.478039	1.572981417	high
MB-5408	8.334246575	0	7.506995	5.219305	5.768656	10.957324	5.351053	0.903854725	low
MB-5475	0.208219178	1	7.944615	8.299165	6.805327	5.529835	5.64488	0.774396895	low
MB-5100	1.635616438	1	11.039272	5.243941	6.38745	5.031361	10.79653	1.601993987	high
MB-5502	15.59452055	0	11.024631	5.442064	6.051022	7.124736	5.029348	1.351559029	high
MB-4293	18.01369863	1	6.584561	5.795876	6.361532	4.272442	4.26284	0.886462076	low
MB-5381	16.58904109	1	8.700959	4.682811	6.678979	10.164693	7.549518	1.003567352	high
MB-4834	2.97260274	1	9.762418	4.303854	6.444718	5.489281	5.433581	1.317298512	high
MB-5498	18.50684932	0	8.440004	4.896131	5.66952	10.347217	5.405447	1.050570977	high
MB-4749	4.230136986	1	8.278455	5.550212	6.578178	9.72656	5.43905	0.883935481	low
MB-4818	6.265753425	1	7.638372	8.444964	6.065939	5.707742	5.349399	0.814701202	low
MB-5296	1.134246575	1	8.922279	5.766803	6.203028	6.777609	6.060269	1.090907675	high
MB-5446	13.38356164	1	7.307589	6.115074	6.092941	9.542235	12.047182	0.964667905	low
MB-5093	11.70684931	1	9.688229	5.472553	5.660314	6.363526	5.665574	1.30323784	high
MB-5350	4.408219178	1	9.255219	6.280109	5.566169	9.963171	4.260348	1.022594633	high
MB-5206	16.90958904	0	7.369862	7.998933	6.185261	7.992133	5.375407	0.749345331	low
MB-4687	6.090410959	1	8.425887	5.314154	4.919707	5.641425	5.183317	1.31357365	high
MB-5442	16.91506849	0	10.158977	4.308952	6.21962	5.538852	13.015708	1.674954257	high
MB-5454	4.164383562	1	8.686049	4.84737	5.566512	9.67488	4.390938	1.094875899	high
MB-5360	13.24383561	1	7.07959	6.697166	6.077661	4.497824	6.005937	0.932114021	low
MB-4827	3.756164384	1	6.261447	6.287297	5.778226	9.994784	5.064813	0.756137662	low
MB-5455	5.079452055	1	8.015499	5.466607	6.355288	7.419235	12.018455	1.122288738	high
MB-5370	9.805479452	1	8.931534	5.889288	6.561766	4.783159	4.7365	1.066249839	high
MB-5290	3.035616438	1	7.916624	5.494679	6.93295	4.835716	7.188591	0.99845167	high
MB-5402	8.857534249	1	8.440004	5.887769	6.628722	4.139983	5.870467	1.056111222	high
MB-5288	10.55342466	1	7.940245	4.55864	6.709994	4.277639	4.75762	1.070107992	high
MB-5384	5.849315068	1	9.305065	4.767558	5.963856	8.238122	6.470912	1.215996968	high
MB-5358	2.369863013	1	7.805799	7.209349	6.210705	8.27428	5.148668	0.815750531	low
MB-4991	10.42739726	0	9.17768	4.939789	6.428661	8.06847	5.201453	1.08468857	high
MB-5401	5.767123288	0	6.951541	5.275684	6.968644	5.340671	3.868595	0.839494297	low
MB-5412	18.10136986	0	7.592786	6.654564	6.366127	4.98912	4.574556	0.898502588	low
MB-5193	4.460273973	1	7.690013	8.293647	5.70801	6.657894	5.137545	0.840083339	low
MB-5182	11.03561644	1	6.382365	5.638938	6.056516	5.688483	5.290755	0.897040902	low
MB-5157	1.498630137	1	8.328614	5.415882	5.135129	8.198725	10.686669	1.303669477	high
MB-5161	14.4109589	0	5.514809	7.398099	5.734395	7.009284	5.857223	0.730614275	low
MB-5211	20.36986301	1	8.473978	5.77509	6.491007	6.790748	9.201342	1.076284379	high
MB-5209	14.39178082	1	10.008954	4.120306	5.684596	4.698033	8.347321	1.67052673	high

MB-5227	15.37260274	1	7.733352	6.326662	6.031925	10.689845	4.779643	0.813851881	low
MB-5213	3.567123288	1	9.179114	4.960325	4.479799	5.542627	11.694406	1.796658491	high
MB-5166	6.635616438	1	9.350327	4.943684	5.444179	5.775706	9.376505	1.502973236	high
MB-5143	10.51506849	1	6.695396	5.898995	5.27507	6.270877	4.808011	0.981365106	low
MB-4000	2.306849315	1	7.258015	5.674882	5.681043	4.147239	4.19371	1.053610387	high
MB-5240	8.534246573	1	6.54645	7.862088	5.746818	8.598921	5.005801	0.723109129	low
MB-5117	17.2	0	8.111198	6.421421	5.917127	6.234506	8.130853	1.065690049	high
MB-5176	9.257534244	1	8.731211	5.643716	4.965255	5.564974	4.838506	1.303463903	high
MB-5144	17.50958904	0	9.987144	4.767295	5.511081	5.135568	5.162151	1.489695757	high
MB-5200	4.298630137	1	10.342582	5.141248	5.645002	4.96742	5.547922	1.491005508	high
MB-5604	11.96986301	0	9.078322	5.246266	6.657327	7.077335	4.914654	1.043173641	high
MB-5596	13.80273972	1	6.404418	9.14453	6.987588	6.892205	5.011892	0.574157632	low
MB-5590	1.465753424	0	8.276912	6.471712	6.293879	6.298157	5.032757	0.951348074	low
MB-5599	18.48219178	0	8.182639	5.651411	6.48158	9.126803	5.131748	0.892384637	low
MB-5579	7.446575342	1	7.696673	7.299024	6.27988	9.985174	4.162378	0.733337905	low
MB-5617	10.5369863	1	6.867496	5.573601	6.297932	6.208443	4.775449	0.887064495	low
MB-5635	1.73150685	1	8.438344	5.991166	6.15322	5.538852	4.413241	1.034376597	high
MB-5647	2.531506849	1	8.037828	6.002179	5.711261	5.279771	4.47411	1.068854066	high
MB-4018	8.68493151	1	8.132676	7.484388	6.517621	10.704716	4.348572	0.71489911	low
MB-5576	15.94520548	0	10.473645	5.435816	6.39036	8.022071	4.977954	1.185068278	high
MB-5575	9.671232879	1	9.41109	4.887593	6.349913	4.162621	4.401149	1.259388347	high
MB-5567	14.67671233	1	7.945253	6.113838	6.516332	8.82721	5.787679	0.858561986	low
MB-5651	1.726027397	1	8.238746	6.187707	6.288092	7.085111	8.999321	1.033720764	high
MB-5603	14.10684931	1	7.852047	6.28517	6.657768	8.710142	5.116211	0.813826109	low
MB-5622	15.03561644	0	9.588324	4.814771	6.034654	6.399441	5.244733	1.27447958	high
MB-5597	2.690410959	1	7.588501	7.3515	6.448527	8.956191	4.81436	0.741295019	low
MB-5548	2.082191781	0	9.355118	6.177334	5.659944	7.129455	6.392907	1.184592597	high
MB-5531	15.12054795	0	8.143118	6.410833	5.57482	7.963687	5.23992	0.992571055	low
MB-5468	8.167123288	0	10.537187	4.250302	6.666574	4.587101	8.471546	1.525329053	high
MB-5514	15.03287671	0	6.591105	6.387563	7.144287	5.496299	4.587898	0.73405384	low
MB-5510	8.473972603	1	6.997559	7.336495	7.33541	4.519559	4.130151	0.706009232	low
MB-5432	8.095890411	1	7.759561	4.999446	6.299566	6.81376	4.987631	0.995006167	high
MB-5525	0.164383562	0	9.031217	7.259409	6.812626	4.469877	4.626908	0.942245705	low
MB-5562	8.463013701	1	7.822854	7.864639	6.754038	7.710751	8.119625	0.784342125	low
MB-5551	14.97808219	0	8.52714	5.188016	7.017153	8.521214	4.801388	0.897932921	low
MB-5560	15.3369863	0	10.02528	4.61312	6.515904	9.004638	9.344547	1.269956943	high
MB-5550	14.07945205	1	7.879954	6.526075	6.725184	6.92976	4.43794	0.828920502	low
MB-4421	21.76164384	1	8.16067	5.19755	6.993788	4.939602	4.573776	0.973838992	low
MB-4408	16.97808219	1	10.346269	3.97819	6.86801	4.933971	9.610295	1.508001984	high
MB-4146	1.284931507	1	8.526259	3.854608	6.220429	4.630851	11.816606	1.486284614	high
MB-5534	6.501369863	1	8.909553	5.808782	5.492765	10.046985	4.750754	1.045105631	high
MB-5521	10.25753425	0	7.80112	5.586229	5.811951	5.576361	8.570963	1.155973276	high
MB-5482	1.452054795	1	9.624125	6.356335	6.000458	7.755201	12.383363	1.282562465	high
MB-5532	6.564383562	1	8.731211	6.438301	6.270054	5.272141	5.323515	1.041430061	high
MB-5556	18.5260274	0	8.119469	5.092491	6.496612	5.007098	5.134802	1.059901713	high
MB-5535	9.8739726	1	8.766952	5.346295	5.462343	7.880119	11.877352	1.355786748	high
MB-5477	9.079452058	0	8.829406	5.736765	6.726547	5.474216	4.58298	1.016534441	high
MB-5540	3.980821918	1	9.500355	4.554598	5.780408	4.99728	9.776518	1.552052036	high
MB-5184	7.342465753	1	5.85434	7.640513	6.587732	10.114291	10.043161	0.651679651	low
MB-5565	15.95342466	1	9.218989	5.413121	7.183992	4.623993	4.957164	1.052639179	high
MB-5040	6.506849315	1	9.306527	6.059976	7.437737	5.015244	4.636085	0.954775725	low
MB-5499	10.16986301	0	5.789134	7.045204	7.168138	8.433267	4.826305	0.586892356	low
MB-5243	3.15890411	1	8.075878	5.268303	6.32469	8.029356	4.3983	0.948731964	low
MB-5457	13.5260274	1	7.243655	6.57893	6.939513	5.816963	9.948158	0.885996201	low
MB-5478	2.819178082	1	6.754051	6.577542	7.304418	10.287321	10.715378	0.704141183	low
MB-4791	14.28767123	1	8.521849	5.134436	6.888023	6.367169	7.654962	1.051940113	high
MB-5118	17.40273972	0	7.844863	5.375788	6.864812	9.114556	5.250479	0.838602029	low
MB-4970	7.298630137	1	7.866558	5.337028	7.175601	4.253452	11.650323	1.096769797	high

MB-2963	18.72602739	1	7.979439	6.713053	5.929731	10.775547	4.873436	0.820027447	low
MB-2954	0.452054795	1	7.66958	6.320645	7.256737	7.934051	5.05224	0.750986959	low
MB-2725	19.4739726	0	5.725599	6.745004	6.059253	10.526409	6.020746	0.668911873	low
MB-2705	13.45753424	0	7.836476	5.374056	6.36528	6.294134	5.148089	0.985251584	low
MB-2728	4.224657534	1	9.407787	5.510604	6.111523	6.041626	8.78573	1.287773486	high
MB-2708	10.95068493	1	7.950881	6.477595	6.07533	9.779634	6.290649	0.870950275	low
MB-3064	13.57534247	1	7.764093	7.272158	5.799274	7.561222	4.975042	0.873896297	low
MB-3049	21.36712329	0	7.247421	7.26915	6.222062	11.748623	4.513525	0.674570801	low
MB-3037	8.123287671	0	7.264097	7.005307	6.61383	9.939949	5.440434	0.707746408	low
MB-3083	6.665753425	1	9.347158	5.995484	6.834838	5.733537	5.253881	1.038425148	high
MB-3046	6.068493151	1	7.754957	4.977041	5.98856	8.041879	6.671288	1.038224101	high
MB-3067	21.33150685	0	9.183324	5.092178	6.119809	8.473671	11.288186	1.269235542	high
MB-3351	19.87945206	0	6.372115	8.175222	7.084181	5.80434	5.143101	0.632838716	low
MB-3361	5.605479452	1	9.51308	6.730993	6.540131	9.152382	9.125167	1.012764895	high
MB-3412	18.80547945	0	6.963861	6.099231	6.851886	9.403605	4.789856	0.715257911	low
MB-3429	20.01369863	0	6.19398	7.410956	6.423581	9.783893	8.248886	0.680871837	low
MB-3355	8.95890411	0	8.975073	4.984112	6.405042	5.249297	11.133067	1.33669532	high
MB-3329	3.120547945	1	8.609229	5.794726	6.462124	7.033044	10.531902	1.11771522	high
MB-3341	12.22739726	1	8.168427	5.907529	6.777106	5.099702	12.168553	1.12432145	high
MB-3388	18.7890411	1	9.644227	4.663112	6.560873	5.367001	6.770966	1.28985398	high
MB-2749	8.035616438	1	6.339344	7.414841	6.182468	10.858497	4.928118	0.638971843	low
MB-2754	16.72876712	1	7.80112	7.211119	6.120992	7.821438	9.996662	0.935783185	low
MB-2747	19.26027397	1	7.783079	8.970136	6.336234	5.4201	6.068193	0.783702264	low
MB-2760	9.70136986	1	8.09355	8.541515	6.244772	10.726763	5.598283	0.701212917	low
MB-2742	2.046575342	1	9.203215	4.454581	6.099991	4.822274	12.944144	1.570560252	high
MB-2750	11.95342465	1	7.432323	7.164849	6.460857	9.027946	6.301994	0.763138225	low
MB-2953	2.476712328	1	8.930366	6.935873	6.126125	7.313038	4.843381	0.963539996	low
MB-3014	21.58630137	0	9.111208	6.088695	6.84958	5.226851	4.707907	1.010170969	high
MB-2951	21.64657535	0	8.305287	7.282503	7.352482	10.093785	5.471897	0.687192087	low
MB-2970	20.78356165	1	6.933627	7.29842	6.942801	9.342593	4.797557	0.643222743	low
MB-3479	19.04109589	0	6.951966	7.336495	6.1353	11.773257	5.007912	0.667155231	low
MB-3490	8.416438356	1	9.144828	6.293693	6.383251	6.211956	5.06179	1.039252361	high
MB-3459	19.84109589	0	10.17773	6.733916	6.2273	9.649583	7.961429	1.080731315	high
MB-3492	9.709589038	1	8.544053	5.184417	5.92936	7.74193	6.256841	1.112353471	high
MB-3303	20.04657534	0	7.785377	6.510061	6.348242	5.682539	4.826472	0.912006717	low
MB-3300	20.30136986	0	8.484165	5.110014	6.783915	8.638687	4.502689	0.919784709	low
MB-3272	1.863013699	1	8.289887	5.236625	6.739143	6.208889	5.470499	0.996976857	high
MB-3271	5.380821918	1	9.402681	5.757018	6.296719	4.840554	8.599637	1.275082639	high
MB-2564	23.46027397	0	6.638939	7.230601	6.691345	8.02143	6.035319	0.699803359	low
MB-2624	10.56438356	0	7.398726	7.341523	7.032031	8.989283	4.887729	0.671314064	low
MB-2853	22.16164383	0	8.307603	7.119514	7.363458	9.0783	4.73254	0.706872022	low
MB-3235	19.40821918	0	9.709449	4.349912	6.260626	10.175252	4.607639	1.12552843	high
MB-3165	18.18356164	0	9.220416	4.429328	7.007102	4.262276	6.66753	1.225374982	high
MB-3171	18.05479452	1	7.145364	7.471001	7.213631	6.691985	8.479727	0.740438467	low
MB-3167	11.25479452	0	8.016814	5.873051	6.656431	5.454803	11.439438	1.098390942	high
MB-3222	1.967123287	1	6.867077	6.797686	6.754483	7.866955	4.646248	0.714023725	low
MB-3497	1.273972603	1	7.838264	6.403768	6.390772	6.80691	10.405289	1.004604462	high
MB-3548	19.27671233	1	8.003262	5.599281	6.293038	5.218767	4.718557	1.020204818	high
MB-3506	19.79452055	0	9.878594	5.186036	6.562626	4.179833	9.167833	1.391569362	high
MB-3525	19.45479452	0	8.879005	4.36762	6.399992	5.898344	7.085544	1.240743141	high
MB-3606	9.093150682	0	10.019769	4.984112	5.780036	4.25407	10.216436	1.634355378	high
MB-3545	19.12876712	1	7.134202	7.394865	6.360265	10.257422	6.239513	0.708244903	low
MB-3470	5.139726027	1	8.499016	5.551288	5.97926	5.083661	5.806892	1.155971495	high
MB-2765	18.72876713	1	7.750942	6.497005	6.304094	11.319758	4.456061	0.752705683	low
MB-2769	13.15068493	0	8.309945	8.979164	6.69359	7.571023	4.960874	0.71130593	low
MB-2772	12.38904109	1	8.935099	7.408393	6.202226	9.170983	4.913235	0.865797879	low
MB-2779	22.62191781	0	8.129195	5.3216	6.45576	5.940801	5.171251	1.017093916	high
MB-3005	20.8	0	6.703695	6.684257	6.879629	7.827671	5.485604	0.711001281	low

MB-2977	9.090410959	1	6.6207	6.290281	7.112734	6.322448	4.635666	0.725606618	low
MB-2996	15.71780822	0	7.527631	8.90675	6.985718	8.030021	4.659331	0.622654842	low
MB-2999	21.53424658	0	8.588676	4.793044	6.127712	10.762529	4.1828	0.966080009	low
MB-2994	20.91780822	0	9.04887	6.598806	6.239464	10.891486	8.270147	0.94480816	low
MB-3002	11.55342466	1	8.423442	6.362898	6.767578	7.693381	5.037633	0.869046499	low
MB-2912	21.97808219	0	9.80607	5.156428	5.714885	6.059165	13.078619	1.602431715	high
MB-2851	13.06849315	1	8.095579	6.035508	6.709994	7.987606	4.53014	0.852239378	low
MB-2840	22.10958904	0	6.770557	7.688299	6.449815	6.222599	5.210307	0.737474772	low
MB-2843	19.03013698	1	7.691129	7.180005	6.334997	10.76521	5.111627	0.730794521	low
MB-3013	19.59178082	0	7.211742	8.113351	6.975248	10.999059	4.517483	0.580759935	low
MB-3058	20.89863014	0	7.703411	6.747401	6.090608	4.695029	8.65484	1.03850642	high
MB-3057	2.632876712	1	10.248809	4.334525	6.23623	4.081049	7.308715	1.55103953	high
MB-3008	12.29589041	0	5.308824	7.857127	6.524092	10.300685	5.569759	0.550777963	low
MB-3032	20.55890411	0	7.94711	7.401274	7.145281	8.48999	5.461466	0.714217302	low
MB-3007	9.443835616	1	8.606382	5.881186	6.934378	7.822703	4.957814	0.891259263	low
MB-3437	8.93972603	0	8.058427	7.346503	6.8012	6.146462	4.836167	0.811064272	low
MB-3430	18.0630137	1	7.953363	6.231226	7.244458	7.359113	4.997626	0.792559484	low
MB-3110	8.279452052	0	7.967325	9.165476	6.595575	10.53086	5.117549	0.625538714	low
MB-3103	2.361643835	1	9.163873	5.784541	6.078059	5.502567	11.458455	1.338580764	high
MB-3085	17.33972603	1	6.573149	7.378832	6.468926	8.403051	6.474988	0.707429822	low
MB-3122	21.18630137	0	8.824019	4.713896	6.018257	6.462263	11.602737	1.378963141	high
MB-3104	6.194520548	1	9.231894	5.16685	6.695371	7.179211	8.824943	1.154099991	high
MB-2835	4.523287671	1	7.526069	7.274004	6.010894	6.109054	6.22699	0.894975638	low
MB-2857	20.60273973	0	8.723054	6.028758	6.552207	8.362339	5.372479	0.932436581	low
MB-2827	19.36986302	0	9.72892	5.660218	5.99125	6.736906	5.582887	1.213323753	high
MB-2797	20.79178082	0	8.634804	6.423658	6.440473	7.461488	5.315152	0.937467328	low
MB-2795	22.36438356	0	7.424834	6.257241	6.476938	7.632446	5.573022	0.840841212	low
MB-3850	5.942465753	0	6.16564	8.029233	6.4295	7.161382	4.328135	0.645588934	low
MB-3838	11.0520548	1	7.943366	6.616496	6.576014	6.92273	8.144131	0.920320008	low
MB-3824	10.39452055	1	9.051381	4.755854	7.187023	4.517373	4.407832	1.079551884	high
MB-3781	18.45753425	0	7.626482	6.521987	6.636624	10.63191	10.173224	0.825252956	low
MB-2686	14.56986302	0	10.171405	4.842242	6.131705	8.631367	4.860082	1.221409345	high
MB-2634	22.52328767	0	8.888063	7.233565	6.762161	10.107519	5.423015	0.791699075	low
MB-2613	13.43013699	0	9.498496	5.280739	6.203028	8.486852	5.492972	1.116710715	high
MB-3871	14.20273973	1	7.696673	6.540259	6.311496	11.08271	4.751009	0.756454768	low
MB-3105	20.73972602	0	6.924712	5.542329	6.532287	4.121906	8.590386	1.011220945	high
MB-3852	18.53424658	0	8.363399	5.000076	6.104751	4.973785	4.498387	1.139608185	high
MB-3854	8.156164384	0	6.580769	7.167165	6.695828	10.47631	4.872524	0.627284172	low
MB-3865	16.43561644	0	6.463699	7.665006	6.491865	11.545737	4.818138	0.59204834	low
MB-3028	3.498630137	1	8.435812	5.277382	5.234413	6.253584	9.699059	1.36953893	high
MB-3567	19.4	0	8.586789	6.115074	5.374445	4.924494	9.864549	1.33984711	high
MB-0444	4.55890411	1	8.425887	8.336942	5.915187	5.495174	4.861498	0.901735246	low
MB-0133	12.4109589	0	5.598485	7.395521	5.730395	5.013324	5.171251	0.775496529	low
MB-0048	8.534246573	0	7.506995	7.249739	9.099794	9.21944	5.282316	0.51121385	low
MB-0083	7.073972603	1	7.979439	7.641232	8.385809	9.076304	5.294535	0.576929979	low
MB-0056	5.167123288	0	8.4218	7.051307	8.420247	7.908628	4.57582	0.641361048	low
MB-0068	8.476712326	0	7.629177	8.212487	8.16811	6.44797	5.046979	0.597208153	low
MB-0093	12.59178082	0	7.333431	7.825053	8.179259	5.342051	4.505692	0.611908179	low
MB-0108	3.509589041	1	8.655889	5.372675	8.027773	10.045269	5.204687	0.745727009	low
MB-0006	13.55616438	0	6.578472	7.40644	7.632349	5.199305	11.508838	0.747738085	low
MB-0022	8.180821918	1	7.998008	7.335815	8.021075	11.865475	6.310264	0.581036631	low
MB-0062	12.65479452	0	9.382848	5.10744	7.880426	5.324216	5.203213	0.97522257	low
MB-0081	5.712328767	0	7.033754	6.825775	8.753964	8.809623	5.263411	0.536905455	low
MB-4673	7.150684932	1	8.324725	4.514405	6.058857	5.823498	4.752046	1.160028201	high
MB-2919	19.72876712	1	7.05465	6.695747	6.383666	6.758201	7.224435	0.849436709	low
MB-2932	8.912328764	0	8.577615	5.848423	6.090608	7.78538	5.421647	1.015400078	high
MB-4731	3.687671233	1	9.773667	4.406165	6.162016	5.431209	8.650845	1.467602291	high
MB-4725	8.117808219	1	9.097911	5.466955	6.232579	6.326937	9.883326	1.252080942	high

MB-4707	18.18082192	0	9.785286	4.209183	5.54174	7.583086	5.340937	1.405752124	high
MB-3383	1.906849315	1	10.107823	5.377182	6.304903	5.119038	10.982438	1.468261101	high
MB-3297	19.40273973	0	10.104871	5.589121	6.397871	6.36443	12.9099	1.428622406	high
MB-7114	10.13150685	0	9.503947	5.058996	5.995882	4.109583	9.378747	1.4785884	high
MB-7118	11.55342466	0	6.87698	5.170099	5.829389	6.078937	6.567853	1.023561671	high
MB-7140	7.463013699	1	9.017538	6.331441	6.354882	8.588482	4.403649	0.935496531	low
MB-2952	15.54246575	1	6.658069	6.130648	7.096571	7.850712	4.999497	0.708156662	low
MB-7099	13.41095891	1	8.131963	5.910975	6.840852	6.842777	8.933537	0.973302447	low
MB-7066	7.378082192	0	7.095085	5.96634	6.555239	5.557765	7.111654	0.914158284	low
MB-7063	7.117808219	0	7.561551	7.234167	6.947475	8.340967	4.77851	0.709258223	low
MB-7102	8.556164384	1	7.561551	6.274979	6.813094	5.771635	4.55648	0.84356831	low
MB-7053	10.03835616	0	7.67728	5.183103	6.879629	7.518753	5.000193	0.876339989	low
MB-7074	8.890410962	0	6.530193	6.259362	6.361111	10.863977	4.452879	0.686339475	low
MB-7109	9.66027397	0	9.632093	5.694078	6.667459	5.763186	5.621297	1.126826473	high
MB-7071	8.389041099	0	9.018713	5.404093	6.237445	4.557631	5.061539	1.186025355	high
MB-7059	9.808219175	0	8.743371	4.966122	6.368196	5.525685	9.186092	1.247328923	high
MB-7076	11.7260274	1	7.615252	8.144038	6.87635	5.02204	4.358962	0.742459707	low
MB-7111	2.479452055	1	9.229086	5.345616	6.258192	9.282192	4.593549	1.025048238	high
MB-7026	9.180821918	1	7.099958	6.584013	6.579885	5.41723	5.384671	0.839425694	low
MB-7019	6.635616438	1	7.422878	7.631086	6.363624	4.460649	6.368938	0.869236627	low
MB-3500	19.66849315	0	9.529573	4.72018	6.394549	4.456411	6.010867	1.317194957	high
MB-3435	2.090410959	1	8.882407	4.56926	6.200617	4.421662	11.161469	1.448135559	high
MB-7196	8.802739726	1	9.688229	4.729501	6.268832	4.482334	4.840351	1.323569358	high
MB-7171	13.18356164	1	9.881055	5.471859	6.971928	7.638898	4.845615	1.038402192	high
MB-7145	11.36986301	0	10.656751	5.404789	6.832983	4.996492	12.529436	1.491398041	high
MB-7132	10.81643836	0	7.553696	8.411265	6.826485	6.351776	4.47635	0.698449611	low
MB-7189	11.80273973	1	10.078973	5.381341	5.935506	4.771528	4.529568	1.347073932	high
MB-7138	10.10958904	1	9.318783	6.562346	6.545309	5.499962	4.84052	1.030807076	high
MB-7232	14.59726027	0	6.558995	6.51191	6.805781	6.408605	5.159599	0.747115746	low
MB-7280	16.07123287	0	7.665754	5.168791	6.772609	9.02597	5.146444	0.848971897	low
MB-7227	5.194520548	1	7.74813	5.198542	6.832507	6.373506	5.206164	0.925928163	low
MB-2896	19.50958904	1	6.810284	7.319522	6.742303	9.048484	4.80176	0.659240436	low
MB-2895	16.15616439	1	8.689969	6.473972	5.89314	7.948801	6.906777	1.034508573	high
MB-2939	8.323287674	1	9.132566	6.147635	6.35364	10.648526	9.264291	1.000734912	high
MB-7002	4.761643836	0	8.226995	7.528846	6.125318	10.095511	4.169202	0.772204694	low
MB-7000	8.005479452	0	9.084777	5.165207	6.566534	8.397121	6.106185	1.046163183	high
MB-7006	9.035616436	0	5.673332	7.565224	6.451921	7.420042	5.117822	0.642084398	low
MB-7011	5.75890411	1	9.626102	5.709969	5.968452	4.680825	9.819071	1.415460544	high
MB-7001	8.44109589	0	7.868994	8.898276	6.356966	8.728863	4.535922	0.685462052	low
MB-7009	4.501369863	1	9.298848	6.075712	6.524963	7.560653	4.719592	0.997604374	high
MB-7005	3.726027397	0	7.623755	7.371809	6.22852	4.694435	4.262613	0.871666751	low
MB-3153	18.73424657	0	9.801494	4.673439	6.851427	4.596022	9.83171	1.381480347	high
MB-3211	11.95890411	0	9.223249	5.399966	5.789303	5.621986	4.72191	1.234502001	high
MB-3181	14.64383562	1	8.475677	4.857937	6.771244	7.599245	6.079143	1.007238612	high
MB-3252	8.194520548	0	6.696988	6.725176	6.342437	10.2892	6.799187	0.72526369	low
MB-3266	4.208219178	1	8.414362	7.262451	6.740471	5.188267	4.878561	0.880547642	low
MB-7082	8.076712329	0	9.343951	4.870446	7.042684	6.549599	11.504853	1.233592904	high
MB-7091	9.063013701	0	6.837499	6.086627	5.707643	4.419098	8.94346	1.077899267	high
MB-7085	9.049315068	0	6.7197	6.964412	7.661624	7.117944	5.564655	0.640220468	low
MB-7018	7.112328767	1	7.991011	6.572463	6.283175	9.547275	5.764307	0.839337481	low
MB-7080	7.498630137	0	6.71088	7.251523	6.603802	9.151345	5.945854	0.684606889	low
MB-7161	9.989041093	0	7.779576	5.767923	6.919346	5.425077	5.445935	0.911052933	low
MB-7123	11.3150685	0	7.512102	7.543396	6.044425	6.997568	6.423306	0.849655058	low
MB-7121	7.301369863	1	9.858995	4.74437	5.725707	7.244566	10.284428	1.497827786	high
MB-7172	12.27123288	0	7.907361	5.791341	6.00935	7.382853	5.078363	0.972480366	low
MB-7127	11.12328767	0	8.012238	5.823591	5.877352	4.082599	4.852684	1.108415273	high
MB-7157	18.05205479	0	8.310715	5.579382	6.318941	5.540711	4.643388	1.035747698	high
MB-3536	7.087671233	1	8.16067	6.203177	6.151613	6.817192	9.691146	1.070684253	high

MB-3530	9.28493151	1	8.162085	6.24925	6.61383	7.878233	4.82841	0.864137098	low
MB-3582	18.8520548	1	8.064491	6.532952	6.297932	5.733935	4.97412	0.943433746	low
MB-3528	9.342465756	0	8.679186	5.347322	6.37736	6.759653	5.101392	1.051504606	high
MB-3576	2.043835617	1	9.82748	5.626494	6.269238	4.608469	4.864362	1.247028755	high
MB-2771	3.684931507	1	8.530649	6.136858	5.708371	7.964332	5.433918	1.037327903	high
MB-7143	15.31780822	0	8.852202	6.072507	5.176193	4.159931	6.036978	1.333506554	high
MB-7197	15.44109589	1	7.942116	6.106066	6.106349	4.934455	12.445875	1.204795475	high
MB-7153	11.7369863	0	7.660835	5.778863	6.290552	4.386579	9.093876	1.105791885	high
MB-7199	6.246575342	1	8.303765	7.070061	6.23623	5.444666	4.853038	0.940741706	low
MB-7215	17.17534247	1	6.730508	6.557745	6.253275	11.50038	4.416331	0.679146007	low
MB-7200	8.021917808	1	7.238565	7.31144	6.506505	4.090887	4.792489	0.837883785	low
MB-7256	2.852054795	1	8.443369	5.614504	6.076496	9.19076	5.658776	0.981227086	low
MB-7194	3.835616438	1	9.692455	5.112912	6.136111	5.875913	4.728844	1.247251724	high
MB-2990	21.36986301	0	7.168186	6.011739	6.811719	7.665902	4.992981	0.786163351	low
MB-7154	8.915068496	1	8.822899	4.48349	6.701558	7.045324	4.925437	1.074267225	high
MB-7151	11.87123287	0	10.379326	4.458633	5.864941	5.437747	7.08097	1.558831525	high
MB-7228	19.65753425	0	7.109708	6.533399	6.440473	11.95302	5.866062	0.699804908	low
MB-7218	13.59726027	0	7.014808	5.962	6.56478	8.364573	5.138944	0.789253136	low
MB-7147	13.85753425	1	8.873384	6.78025	6.332514	4.206716	4.925437	1.046524759	high
MB-7219	13.57808219	0	7.368902	4.931358	6.27457	10.489269	5.201754	0.859627003	low
MB-3436	4.17260274	1	9.324961	5.152234	6.424418	5.813316	4.814833	1.157069156	high
MB-3417	8.504109592	0	7.520853	7.327695	7.117624	5.279103	4.735058	0.757432753	low
MB-2721	20.68219178	0	7.379573	6.017248	7.242929	9.771032	4.230216	0.691620976	low
MB-3092	12.52054794	1	7.733352	5.984639	6.605109	6.736906	5.25234	0.888600002	low
MB-2850	0.775342466	1	9.05014	7.038568	6.735094	9.043555	9.479233	0.930982824	low
MB-2823	22.18356164	0	7.929046	6.080554	6.827446	11.919414	4.993219	0.728914507	low
MB-2792	9.104109592	1	8.221104	6.396746	5.958118	7.415624	8.020761	1.028984627	high
MB-7273	17.11232877	0	8.406999	4.399363	5.38817	4.2068	5.271448	1.384461625	high
MB-7283	2.37260274	1	9.136694	5.605815	5.784832	6.676575	4.885089	1.168462108	high
MB-7287	7.969863014	1	8.730172	4.920049	4.952494	6.205816	11.092782	1.558049875	high
MB-7238	1.912328767	1	9.017538	4.488457	5.696804	9.427287	11.312645	1.346485473	high
MB-7212	4.315068493	1	7.174947	5.604347	6.063164	9.164735	5.131199	0.860948891	low
MB-7266	5.602739726	1	7.252939	5.390346	6.119416	7.295516	4.397502	0.915664034	low
MB-7201	13.43287671	0	9.056481	5.558468	6.405042	9.179294	10.27356	1.109002987	high
MB-2778	14.89589041	1	7.884177	7.526085	6.470613	4.82514	4.524166	0.854993505	low
MB-2815	21.16986302	0	6.112646	7.491079	5.946226	9.187646	5.789625	0.692655114	low
MB-2833	21.35068493	0	8.292943	6.156238	5.78078	7.816561	4.92935	0.995488102	high
MB-3614	19.34794521	0	8.932747	5.11899	6.499181	5.137064	4.285792	1.116918101	high
MB-7052	8.723287668	0	10.262146	4.978262	6.212319	8.467494	5.502848	1.229917982	high
MB-7133	1.958904109	1	9.78758	4.747038	6.552207	4.900586	6.51856	1.313722169	high
MB-7095	9.504109586	0	8.482482	7.334629	6.699775	4.595917	12.107019	1.065530412	high
MB-7072	4.301369863	1	9.358444	5.31889	6.069865	5.097924	4.899645	1.236035967	high
MB-7022	7.02739726	0	8.176211	5.539824	5.920582	8.658531	11.892844	1.153017908	high
MB-7035	2.635616439	1	9.157005	5.878059	5.767558	6.017264	5.704365	1.196729965	high
MB-7062	7.879452055	0	7.321351	7.361619	6.500035	7.18711	4.806485	0.759949445	low
MB-5463	4.778082192	1	9.4297	5.2962	6.144041	5.316065	5.108108	1.230679711	high
MB-4484	4.298630137	1	7.872019	7.185288	6.211107	7.66061	4.759998	0.832032132	low
MB-4750	3.101369863	1	8.956132	4.373131	6.442597	8.432517	11.801973	1.270770454	high
MB-4843	21.79178082	0	7.385454	5.308722	7.014274	7.062388	8.176198	0.903513464	low
MB-5563	9.671232879	1	6.394483	7.918108	6.352814	5.62664	5.531769	0.727784409	low
MB-5530	15.28219178	0	8.631964	5.866864	6.142059	8.564478	10.442686	1.1052156	high
MB-4836	1.926027397	1	9.973839	4.044247	6.278639	7.031493	4.895581	1.317726795	high
MB-4828	3.493150685	1	10.17773	5.002837	5.305998	6.096192	6.213568	1.521341322	high
MB-4697	9.698630137	1	8.397108	7.190523	6.112325	10.329546	4.730388	0.811258893	low
MB-4653	3.956164384	1	7.582101	5.994688	6.812626	9.48153	10.64958	0.876931358	low
MB-4660	3.276712329	1	9.989817	4.157808	7.948168	4.146858	13.078619	1.370758167	high
MB-5275	6.243835616	1	8.540455	4.805528	7.30804	6.783455	8.476028	1.023760685	high
MB-4654	5.284931507	1	8.532462	4.822355	6.582936	10.220449	4.830845	0.929071872	low

MB-2629	22.93972603	0	8.176211	6.452106	7.279774	8.152508	5.048482	0.77239043	low
MB-6263	6.235616438	0	7.731649	5.426341	6.078059	10.53086	6.659115	0.909284477	low
MB-6187	3.104109589	1	5.985118	6.777284	6.361957	9.378092	7.16741	0.699093051	low
MB-6330	11.53424657	0	7.682202	4.679253	5.633752	4.445882	10.965366	1.378015164	high
MB-6283	7.594520548	1	7.922245	6.047515	6.63313	11.032557	5.71198	0.785760353	low
MB-6359	6.989041096	1	7.948973	5.109032	6.082377	6.166047	4.48102	1.046462974	high
MB-6207	9.723287671	1	7.440284	6.103659	7.264423	6.630285	5.135081	0.78070364	low
MB-6281	0.287671233	1	7.731649	4.821225	6.309819	4.972019	4.67183	1.060451571	high
MB-6322	6.605479452	1	7.735577	5.272298	6.71984	8.091315	11.801973	1.02507279	high
MB-6190	2.024657534	1	6.685864	6.221148	6.627406	7.218411	4.453153	0.759802912	low
MB-6306	5.309589041	1	8.751609	5.159976	5.968835	9.849258	4.533704	1.013676555	high
MB-6188	1.953424658	0	9.873692	4.775202	6.363624	4.683093	5.200581	1.326894654	high
MB-6318	8.978082189	1	9.640256	5.009018	5.92403	5.115134	9.123189	1.460775837	high
MB-6228	16.44657534	0	8.293703	6.639017	5.95467	7.607941	5.423347	0.953062231	low
MB-6225	9.687671236	0	6.934053	7.096872	5.62139	7.784167	4.879855	0.830432777	low
MB-6280	2.290410959	1	9.211813	5.694448	7.179081	4.371874	12.162259	1.223458522	high
MB-6253	15.97534247	0	6.930642	7.361619	6.587307	8.966511	6.871291	0.714105593	low
MB-6098	0.873972602	1	9.778292	4.739333	6.081982	9.065314	4.66404	1.17136259	high
MB-6179	4.805479452	1	8.135468	4.631194	6.197404	9.115614	4.712996	0.991328626	low
MB-6050	15.79726027	0	7.647109	8.795318	6.083536	5.070371	5.284188	0.807412699	low
MB-6169	6.964383562	1	9.092633	5.495762	5.396832	6.835402	10.78388	1.409679398	high
MB-4548	26.53424657	0	9.760186	3.944385	6.474427	3.830217	6.404102	1.456703449	high
MB-4591	3.383561644	1	8.600778	4.493343	6.196996	4.543286	5.159318	1.232515909	high
MB-4557	22.99726027	0	6.638553	5.630144	7.790256	7.006723	4.545985	0.67822911	low
MB-4601	4.624657534	1	9.211813	4.60188	5.63799	7.225322	11.181062	1.471069883	high
MB-4598	9.810958907	1	9.414418	6.154197	6.338706	5.060429	4.863097	1.122239359	high
MB-4578	16.21917808	1	7.574169	5.772052	7.116632	4.898712	4.886037	0.872588614	low
MB-4978	16.30958904	0	8.232144	4.865782	5.901894	10.16647	4.778369	0.990704345	low
MB-5150	9.109589038	1	7.894487	6.577093	6.429083	5.581741	4.605708	0.905002214	low
MB-4695	1.750684932	1	6.493712	7.336495	6.026052	11.428551	5.118356	0.657337921	low
MB-4714	6.876712329	1	9.878594	4.575673	6.423163	4.523595	6.563709	1.386273667	high
MB-4879	6.57260274	1	9.092633	5.000379	6.264723	6.976698	11.02613	1.297154164	high
MB-4881	22.5369863	0	10.372081	4.72601	5.576528	6.014268	12.9099	1.779795261	high
MB-4224	5.383561644	1	8.357869	5.063671	7.42378	5.263178	5.592772	0.955680623	low
MB-4845	2.093150685	1	6.442847	6.482984	6.629601	6.235827	4.888895	0.75874677	low
MB-4904	10.62191781	1	9.837218	5.272298	5.890873	5.004708	10.076817	1.503259472	high
MB-4860	19.28219178	0	8.789992	7.45836	6.578612	7.294975	6.235221	0.884660312	low
MB-5081	4.767123288	1	8.678198	8.152026	6.387853	5.471623	5.030583	0.880926273	low
MB-4896	5.331506849	1	9.231894	5.587308	5.526676	5.561921	9.043193	1.396851993	high
MB-4416	19.85753425	1	9.984385	4.067365	6.212319	4.832589	6.537709	1.484267327	high
MB-4091	23.1260274	1	7.509549	5.796632	6.826029	4.495885	4.594254	0.907812552	low
MB-4254	14.6630137	0	9.677664	5.062737	6.740471	4.182452	13.065844	1.46817429	high
MB-4742	6.832876712	1	6.655341	6.630534	6.040148	8.811372	4.875628	0.763450342	low
MB-5239	15.79178082	0	8.492948	6.432532	6.506924	8.99406	4.382631	0.851756855	low
MB-5251	9.243835619	1	6.996707	8.354052	6.207056	8.631367	5.958148	0.695548671	low
MB-4996	8.2	1	5.73891	6.531582	6.981944	8.662681	5.029348	0.62196802	low
MB-4603	19.6630137	0	6.993717	4.303722	8.025117	4.708029	8.170188	0.881748227	low
MB-4621	22.34520548	0	10.706363	4.061847	7.64778	8.442325	7.155566	1.169699725	high
MB-4222	4.539726027	1	9.890937	4.231971	7.475311	4.193512	4.832335	1.194554283	high
MB-4859	12.96986301	1	6.475327	6.482984	5.737308	8.125751	12.617038	0.966098539	low
MB-4212	27.15342466	0	5.482317	7.113865	5.944301	5.005232	5.824218	0.772037002	low
MB-4949	17.83287672	0	10.036423	4.853657	5.785588	6.133332	5.190006	1.384632373	high
MB-4928	18.0849315	0	9.817936	4.984724	6.144041	6.709878	11.577763	1.447342201	high
MB-5098	15.32054795	1	9.74444	5.406178	6.118628	6.652165	4.986275	1.204181677	high
MB-5122	10.33150685	1	6.826917	6.922813	6.069865	8.5813	4.412139	0.753513642	low
MB-4213	6.945205479	1	7.780732	7.338388	5.906079	5.431922	4.818453	0.917501999	low
MB-4126	14.36712329	1	8.477391	3.748935	6.248	4.777246	4.951748	1.264884363	high
MB-4957	16.24657535	1	9.667047	4.868381	6.360683	4.214248	4.471881	1.290673656	high

MB-4976	18.41917808	1	6.057439	7.30275	6.225288	5.274129	13.448596	0.911078663	low
MB-5110	1.8	1	7.113285	5.442064	5.810447	8.055719	12.156007	1.09306164	high
MB-4141	12.15068493	1	9.127183	4.169131	8.394314	6.424161	4.404817	0.900887504	low
MB-4738	14.47123288	0	5.643271	7.120075	5.87096	7.263938	8.55555	0.781612586	low
MB-4264	13.41369863	1	9.023669	4.87334	6.346157	4.975324	5.992701	1.226346462	high
MB-4794	3.739726027	1	8.186953	7.489037	6.491007	6.715159	8.982586	0.914831211	low
MB-4390	13.50684931	1	6.447663	6.225318	6.687843	5.791431	11.080252	0.897221087	low
MB-4381	5.052054795	1	8.24466	4.118766	7.97794	4.525446	6.639989	0.987090786	low
MB-6021	24.75890411	0	5.755515	5.326783	7.00091	7.325185	5.417563	0.719232211	low
MB-6059	22.88767124	0	8.26182	6.769407	6.486727	7.527387	4.864891	0.863997331	low
MB-6097	13.1260274	1	8.965608	4.448677	7.377631	5.294047	6.206262	1.083494874	high
MB-6023	19.14794521	0	8.172626	5.65691	6.610373	9.054386	4.783054	0.870308701	low
MB-6029	22.87945206	1	7.264565	6.372562	6.879629	8.694194	4.818933	0.735733901	low
MB-5092	14.34794521	1	6.0162	6.191876	7.248553	12.171948	4.325196	0.553139023	low
MB-6068	15.00821918	1	8.012852	5.940507	6.223678	7.257985	10.234006	1.063813873	high
MB-6039	6.010958904	1	9.19035	4.450375	5.718514	6.785872	4.225577	1.272313711	high
MB-6125	12.64931507	1	7.625401	7.402573	5.604549	9.185545	4.640552	0.824954749	low
MB-6042	21.65205479	0	6.989408	6.479388	6.19293	7.277073	6.630222	0.855004902	low
MB-6077	13.99452055	1	9.815606	6.530678	6.325941	10.038663	4.522852	0.954513552	low
MB-6052	20.9890411	0	7.759561	7.223517	6.261865	9.938352	8.694176	0.825986814	low
MB-4189	29.19452055	1	9.397674	4.619849	7.495985	4.224201	4.326503	1.088521379	high
MB-5134	16.15342465	0	5.037458	6.510528	6.589903	5.35382	4.637502	0.680777475	low
MB-4139	13.59452055	1	9.724529	5.583338	6.751796	5.038548	4.25921	1.125398667	high
MB-5135	3.071232877	1	9.369746	4.464876	6.166011	4.462156	5.110011	1.338228997	high
MB-4880	17.33972603	0	10.732148	4.374438	5.969963	4.501005	10.527649	1.784502994	high
MB-5057	4.630136986	1	9.628108	4.811952	5.501248	7.183965	11.450867	1.547550996	high
MB-4098	14.49589041	0	7.784792	4.022414	7.302381	4.376664	4.271321	0.996403051	high
MB-5420	15.9369863	0	8.307603	5.9864	6.907138	7.495396	6.149022	0.895595523	low
MB-5458	15.07671233	0	8.745429	5.223956	5.503283	5.181077	8.184881	1.365932189	high
MB-5410	16.66575343	0	7.275754	5.028849	7.58699	6.514989	6.680643	0.829539451	low
MB-5435	8.547945205	1	8.49121	4.875074	6.685189	5.093225	4.956294	1.080398867	high
MB-4005	15.48219178	0	8.412767	5.664999	6.977133	10.055139	4.854261	0.818972751	low
MB-6288	8.243835616	1	8.92921	7.130286	5.807116	4.708345	4.526719	1.07475348	high
MB-6218	12.14520548	1	6.93913	5.534817	5.543116	7.049998	4.373086	0.960088301	low
MB-6246	4.347945205	1	9.644227	4.285791	5.604893	6.788318	6.757574	1.448861226	high
MB-6308	12.48767123	1	7.068648	6.142235	5.249189	5.404777	4.519863	1.024588095	high
MB-6271	12.96438356	0	7.444345	5.893941	5.300547	6.432408	5.949548	1.073342006	high
MB-6245	14.6	0	9.785286	4.688199	4.517069	5.593946	8.418766	1.793209726	high
MB-4343	16.32328767	1	8.288365	4.586801	6.596885	4.592342	7.608875	1.18460876	high
MB-4341	2.769863014	1	8.724076	6.03154	5.566864	4.769536	8.915734	1.308304122	high
MB-4274	4.926027397	1	8.445893	6.315487	6.551342	6.315202	5.093999	0.944633677	low
MB-3526	7.073972603	1	8.128498	6.112622	6.35364	5.810472	10.317553	1.095754508	high
MB-6138	2.106849315	1	7.901231	7.119514	6.196996	10.880403	4.456466	0.749784824	low
MB-6049	21.71780822	0	9.918887	5.056168	6.772146	7.478925	6.006357	1.14257249	high
MB-6055	16.4739726	0	10.006231	4.549681	5.78596	5.589717	7.492242	1.516410203	high
MB-6108	15.94246576	0	7.4278	6.993957	5.980039	5.58288	4.894218	0.89830702	low
MB-6044	18.00821918	1	8.065837	4.470381	6.192117	4.41855	4.840019	1.169795448	high
MB-6181	4.515068493	1	7.158785	4.854522	6.518059	4.528178	7.701583	1.056706052	high
MB-6079	19.65753425	1	8.235073	5.958475	6.83159	9.31891	4.551013	0.817574131	low
MB-6103	19.67671233	0	7.263606	5.82961	6.609947	10.05682	5.997212	0.782246438	low
MB-6016	11.79726027	1	6.51775	7.105306	7.42378	9.58946	4.66302	0.579568419	low
MB-6149	6.15890411	1	9.079556	4.570871	7.378168	10.15952	4.875075	0.895489774	low
MB-6007	5.797260274	1	8.553076	5.470794	7.162645	6.055764	11.577763	1.092507395	high
MB-6147	19.56712329	1	6.085245	7.14802	6.016711	9.633708	4.844415	0.677322948	low
MB-6167	10.09315068	1	6.720904	5.918703	6.416812	10.580449	5.024038	0.728244894	low
MB-6146	7.364383562	1	8.005182	4.891125	5.843948	4.628112	6.952774	1.231912903	high
MB-6160	20.33150685	0	8.205879	4.554376	5.640793	5.668458	6.25293	1.261186207	high
MB-6075	14.98630137	1	8.848871	5.266277	7.10543	6.92221	4.716959	0.957085583	low

MB-6071	4.717808219	1	8.239451	7.130286	6.616444	7.124736	7.583327	0.887515727	low
MB-6214	14.34246575	1	6.373226	6.043106	6.529278	7.812897	4.435783	0.742724676	low
MB-6334	10.52876712	0	9.399339	4.270286	6.261029	8.058427	4.808625	1.184600361	high
MB-6336	1.41369863	1	9.592184	4.225954	6.094485	5.068363	5.534981	1.392117078	high
MB-6232	6.98630137	1	6.948993	7.874639	5.7753	6.21152	6.037794	0.829013715	low
MB-6300	5.246575342	1	7.181283	7.182355	6.266364	6.656934	4.7365	0.798353638	low
MB-4236	16.74246575	1	8.715977	4.616962	6.603802	4.582751	4.605337	1.149592458	high
MB-4234	20.83835616	1	7.556302	4.828299	6.783007	6.630285	4.414298	0.917212609	low
MB-4318	1.408219178	1	10.122805	4.437532	7.42697	5.314354	4.710215	1.163012963	high
MB-4322	16.14794521	1	7.484006	4.538597	7.173125	4.716102	4.999728	0.952174365	low
MB-4351	5.6	1	9.632093	4.129128	7.97079	6.274899	6.131211	1.052595214	high
MB-5063	17.68493151	0	9.073131	4.625386	5.682832	8.515724	10.456934	1.356784868	high
MB-4360	0.975342466	1	11.004162	3.996176	7.813809	5.544138	11.698879	1.443061128	high
MB-4746	20.09041096	0	8.564744	7.198162	5.929731	8.98073	5.301794	0.895805223	low
MB-4838	16.50136987	1	8.462	4.900571	6.71714	4.34711	4.977507	1.097831516	high
MB-4829	14.48767124	1	8.664624	6.536571	5.857763	10.440407	6.695221	0.945507036	low
MB-4802	3.608219178	1	9.193228	6.010947	6.186888	5.212358	7.877333	1.209120955	high
MB-5431	1.304109589	1	8.762877	6.434334	6.115847	8.700126	10.489876	1.07152478	high
MB-5447	12.23561644	1	8.344355	5.987187	6.026443	4.553754	4.43662	1.079170895	high
MB-5434	3.712328767	1	8.020059	5.529126	7.079298	4.4876	6.483608	0.980888244	low
MB-5231	1.361643836	1	8.568419	5.102008	5.590858	6.668935	5.790401	1.206787266	high
MB-5425	4.065753425	1	7.787116	7.15495	5.869089	6.976206	5.65915	0.906327507	low
MB-5334	7.019178082	1	7.47442	5.757389	6.023701	6.780553	4.68295	0.943643243	low
MB-5326	7.906849315	1	6.562449	6.287297	6.113103	12.365026	4.218912	0.673190943	low
MB-4059	14.1369863	1	7.230689	6.821218	6.237445	6.537863	4.97412	0.836014111	low
MB-6150	6.383561644	1	7.6531	5.649935	5.729682	9.562389	4.578674	0.917652673	low
MB-6024	20.73424658	1	7.165924	6.104854	7.262866	8.948673	4.558953	0.694837794	low
MB-6171	18.10684932	0	7.312333	5.234622	6.952173	7.608519	8.55162	0.901520116	low
MB-6060	3.126027397	1	10.39819	4.722561	6.279052	7.376209	4.656529	1.280787572	high
MB-6008	5.679452055	1	9.355118	5.506382	7.297234	7.407857	10.861527	1.086394841	high
MB-6036	23.66575342	0	8.82835	4.781742	5.791147	7.531952	5.661028	1.194718623	high
MB-6178	2.723287671	1	9.206046	5.434062	5.611236	6.56364	5.291698	1.237500294	high
MB-6062	23.20821918	0	9.671274	4.609746	6.002008	4.303255	11.092782	1.605719236	high
MB-6006	5.224657534	1	7.934048	6.564651	6.463393	10.122842	4.907079	0.783456782	low
MB-6048	13.82465753	1	8.97628	5.213698	5.789303	9.434397	11.887624	1.267816242	high
MB-6116	15.8739726	0	9.109896	6.230807	6.225288	6.793682	4.950891	1.04120486	high
MB-4300	15.5369863	0	9.334394	4.06267	6.731091	5.146472	12.430138	1.466661817	high
MB-4417	1.37260274	1	10.183928	4.540374	7.496514	3.995889	8.363795	1.30478175	high
MB-4079	28.84931507	1	10.041911	4.168607	8.149153	5.090879	5.287949	1.086336778	high
MB-4317	14.62191781	0	10.183928	4.545658	6.891783	5.025584	4.56886	1.259027496	high
MB-4332	25.30958904	0	10.02528	4.056653	7.029147	4.445464	11.312645	1.500806362	high
MB-7226	4.38630137	1	10.568234	5.760035	5.821224	5.683348	4.485847	1.350726737	high
MB-7160	12.16986302	0	9.934172	4.348853	5.352555	4.518696	4.491391	1.57363893	high
MB-7167	11.59178082	0	7.497275	6.138506	6.072229	5.845901	7.938257	1.013060094	high
MB-7241	14.0630137	0	7.969835	5.971808	6.185668	7.13573	4.677325	0.940300827	low
MB-7168	12.36986301	0	7.567762	6.850088	5.732816	9.222544	5.226581	0.851045373	low
MB-7162	11.52328767	0	7.543764	6.053576	6.067894	9.999593	4.879112	0.832755034	low
MB-4353	10.56438356	1	8.368158	5.136704	6.693136	5.17825	4.867942	1.040082689	high
MB-4354	1.263013699	1	7.982633	4.196094	6.068675	6.427361	4.391973	1.116724398	high
MB-6083	13.48219178	1	6.977433	5.921045	7.260811	7.198824	5.075278	0.742419633	low
MB-6026	4.087671233	1	9.995204	4.451899	7.488337	7.315259	4.246316	1.053243465	high
MB-6101	6.369863014	1	8.717983	4.891125	5.847691	5.914959	8.199052	1.299916485	high
MB-6182	17.16164384	0	9.116465	4.464876	6.29552	5.65236	13.756618	1.500181397	high
MB-6080	4.446575342	1	8.555817	4.838533	6.171627	5.616217	6.123561	1.182774374	high
MB-0367	11.70684931	0	6.505669	5.777356	5.284389	10.279755	4.621326	0.846557185	low
MB-5178	15.15068493	0	7.749839	5.244948	5.414693	9.074281	4.593131	1.015731878	high
MB-5280	6.4	1	8.395504	5.828859	5.777499	7.01547	4.937675	1.059659475	high
MB-5148	20.15616438	1	6.235894	6.000199	5.942006	10.351097	5.048482	0.744449132	low

MB-4342	19.13150685	1	6.333906	6.202345	5.911702	6.646399	5.233593	0.844001154	low
MB-5225	3.556164384	1	10.107823	4.919456	5.757324	8.130527	10.595044	1.473479661	high
MB-6257	7.479452055	0	8.622435	5.674882	6.450662	4.729399	4.401775	1.062620232	high
MB-6242	13.59726027	0	10.190387	4.417798	5.004838	6.537863	5.02717	1.594855431	high
MB-6237	8.646575342	1	8.856632	5.260281	4.711719	4.495314	7.416643	1.547454253	high
MB-6183	6.504109589	1	8.475677	7.365427	6.163616	4.900121	6.231788	0.992371966	low
MB-6231	5.964383562	1	8.506017	4.912957	6.1206	5.904992	11.520758	1.319883039	high
MB-5175	16.00273973	1	7.879343	6.828294	5.143544	8.859488	4.917084	0.959340469	low
MB-5173	1.819178082	1	9.851655	5.36927	5.137567	7.292829	8.956986	1.500982625	high
MB-3978	8.290410962	1	9.198929	4.885838	6.235007	5.73715	8.610583	1.309684355	high
MB-7288	2.224657535	1	7.904296	5.549847	6.787058	4.435257	11.851429	1.142053502	high
MB-0402	8.197260274	0	5.920076	7.833262	5.770149	10.359112	4.610131	0.635604252	low
MB-5235	16.78356164	1	9.794406	5.887383	5.207664	8.280029	11.694406	1.462399803	high
MB-4931	2.495890411	1	9.726732	5.143502	4.908457	5.613563	9.227815	1.658290049	high
MB-5332	8.123287671	1	9.746649	4.215549	5.156126	5.260156	5.459731	1.600994802	high
MB-7045	7.802739726	1	7.615252	5.763791	5.336597	9.288827	9.27156	1.075442989	high
MB-7049	8.386301367	0	9.306527	5.031666	6.60121	7.382299	9.45017	1.199228757	high
MB-7061	7.953424658	1	7.809965	7.628132	5.535615	4.685677	7.729505	1.038203445	high
MB-7078	0.342465753	1	9.825088	4.690503	5.942006	6.915731	4.417905	1.286674567	high
MB-7058	10.06849315	0	6.155531	6.165414	6.359436	9.895651	5.326389	0.702601182	low
MB-7038	6.660273973	0	9.582571	7.54693	5.986223	6.109925	9.451364	1.153701163	high
MB-0388	8.019178082	0	7.208106	5.942841	6.708229	6.135528	4.94149	0.846099361	low
MB-0116	10.04931507	0	8.505143	7.357192	6.762161	8.281449	5.961401	0.812948514	low
MB-0472	2.252054795	0	7.834122	6.297103	7.00471	4.143531	6.065684	0.919342039	low
MB-0191	1.257534247	1	9.42455	4.923305	5.726069	5.265828	10.628763	1.524822398	high
MB-0231	1.610958904	0	5.153319	9.257692	6.360683	7.73529	7.301316	0.564436093	low
MB-0393	1.712328767	1	8.888063	6.676566	5.501586	9.2354	9.905111	1.125116318	high
MB-0553	7.046575342	1	8.090857	6.668442	6.378642	4.24736	7.497685	1.029690393	high
MB-0587	7.501369863	0	7.653646	6.162055	6.609507	5.892149	4.947879	0.887856917	low
MB-0222	17.48219178	0	6.599914	8.673957	6.646315	8.044514	5.90135	0.625201254	low
MB-0128	11.3260274	0	6.623773	7.649346	6.072616	9.743199	4.62355	0.675068926	low
MB-6363	1.37260274	1	8.284541	6.101243	5.007173	9.844892	5.258786	1.049098314	high
MB-6239	16.46575342	0	8.270162	6.409046	5.672373	7.933418	5.692535	1.002483968	high
MB-6208	18.18082192	0	8.528881	5.709593	5.708371	6.854095	10.164755	1.238506792	high
MB-6273	15.56986301	0	6.682382	5.242284	6.583379	4.225931	4.891742	0.919674246	low
MB-6344	12.56986301	1	10.146743	4.965823	5.277746	8.130527	9.259032	1.5295872	high
MB-6212	18.43013698	0	6.072715	8.043265	5.465317	6.826018	4.477501	0.74284883	low
MB-6327	1.501369863	1	8.168427	6.838982	5.742066	6.636864	5.505311	0.988569766	low
MB-6287	2.893150685	1	9.289596	4.63144	4.641758	6.665123	4.76639	1.49841956	high
MB-6251	1.208219178	1	9.345531	6.008936	5.20825	8.140115	10.125963	1.344920783	high
MB-0489	7.443835616	0	5.898775	6.765008	6.017867	7.918788	7.658402	0.773221263	low
MB-3253	4.589041096	1	9.837218	4.788356	6.405042	4.527613	9.788671	1.465071214	high
MB-3466	19.24383561	1	9.233293	4.533158	5.794725	5.103123	4.73512	1.343643604	high
MB-7286	13.03835616	1	8.09355	6.936388	6.148415	9.177202	4.982443	0.835400989	low
MB-7292	6.449315068	1	8.993061	5.346985	6.312323	6.643029	4.563083	1.084719444	high
MB-7275	3.649315068	1	8.691958	5.58586	6.159618	10.268523	4.672595	0.939198381	low
MB-7297	14.4630137	1	9.360167	4.755854	6.592948	4.63241	7.882812	1.303626497	high
MB-7294	6.8	1	8.544948	5.272298	6.086309	4.585985	4.407579	1.150955904	high
MB-7037	7.101369863	0	7.131952	7.459682	6.509068	8.197332	4.788679	0.714899783	low
MB-7040	5.904109589	0	7.459878	6.543939	6.794315	8.365318	4.575884	0.752872119	low
MB-7020	5.887671233	1	8.970342	6.591413	5.594365	4.499698	11.650323	1.372274872	high
MB-7042	7.273972603	0	8.389838	6.076523	6.414292	7.512538	5.175022	0.939344371	low
MB-7029	6.317808219	0	7.470437	8.987482	5.645002	11.042023	7.15248	0.711662143	low
MB-7027	3.038356164	1	9.422857	4.617447	5.488091	7.042774	8.652426	1.454793486	high
MB-7060	9.260273975	1	8.37216	5.70108	7.203546	7.442345	4.606005	0.854655611	low
MB-7034	6.17260274	0	6.85636	6.737821	6.639687	4.441807	4.62164	0.815679406	low
MB-5204	15.77534246	1	6.911649	6.591413	5.187165	6.903674	7.376903	0.998271788	high
MB-5155	21.36438356	0	7.095529	5.995878	4.943395	6.400367	5.140328	1.064236796	high

MB-5190	19.9369863	0	9.533283	4.468614	5.0688	5.845901	9.730665	1.682664989	high
MB-5592	2.164383561	1	8.50159	5.888906	6.747292	9.621984	7.505461	0.903565182	low
MB-0437	7.443835616	0	8.383391	6.848125	6.322218	9.493855	4.437853	0.824921603	low
MB-0309	3.479452055	1	6.07715	8.365596	6.711802	8.644502	4.627724	0.574422997	low
MB-5655	15.76438356	0	9.289596	4.276264	7.247527	4.655237	6.790603	1.194348552	high
MB-5620	3.457534247	1	7.569933	6.593724	7.067582	7.205669	4.858312	0.762915568	low
MB-0544	6.520547945	1	6.967668	7.10474	6.18085	7.12633	6.14731	0.809523288	low
MB-0233	5.953424658	1	7.161917	5.06907	6.779375	8.840572	5.538218	0.826550738	low
MB-5588	6.668493151	1	7.888394	6.22408	6.440473	7.675448	11.391154	1.009874877	high
MB-5628	3.501369863	1	8.792075	4.709967	6.134483	4.64577	5.894889	1.262234356	high
MB-0253	12.51780822	0	6.561288	8.112354	6.664375	6.5257	5.067354	0.669641542	low
MB-0226	4.736986301	0	5.694687	7.634713	7.257755	9.017171	8.25431	0.581756956	low
MB-0266	7.452054795	1	5.78756	8.174197	6.824647	9.388544	5.051989	0.549620764	low
MB-0531	13.46849315	0	5.93528	7.244348	6.116639	8.257194	6.835648	0.715651819	low
MB-5641	14.94794521	0	9.161033	4.571781	5.623148	6.120308	5.963858	1.354862833	high
MB-5640	14.16164384	0	6.908277	5.900543	6.051421	6.766967	5.112976	0.889285293	low
MB-0230	16.46575342	0	8.870025	6.294126	5.537312	5.638304	12.088798	1.363286653	high
MB-0109	9.238356164	1	9.590265	4.04654	6.332921	4.126923	4.391558	1.371952028	high
MB-0304	9.167123285	1	9.646373	4.069798	5.906468	6.48158	4.503463	1.355490996	high
MB-0147	4.254794521	1	7.22095	5.868396	7.848326	6.256668	5.618309	0.733897013	low
MB-0442	8.504109592	1	5.42202	7.945182	5.479631	10.653308	6.008393	0.639586757	low
MB-0280	14.2109589	1	7.873869	7.240177	5.454401	5.94287	5.68263	0.99674746	high
MB-0178	8.586301373	1	9.694586	4.245706	5.866059	5.997237	5.236889	1.39611022	high
MB-0242	16.36438356	0	7.846044	7.327695	5.982747	8.640352	6.239513	0.848589317	low
MB-5624	11.17808219	0	9.885922	4.646401	6.261865	7.618442	13.053169	1.475702059	high
MB-5593	7.602739726	1	9.384477	4.83144	6.146041	6.580149	11.565683	1.409917073	high
MB-5605	10.16712329	0	8.170522	7.152643	6.813094	7.972073	5.132021	0.787189268	low
MB-5636	8.901369863	1	9.8444	5.94245	6.773961	5.69831	4.867407	1.094955735	high
MB-5566	19.26575342	0	9.839714	4.98928	5.809341	5.034653	9.120215	1.519666008	high
MB-0127	10.85479452	0	7.609379	6.520599	6.042866	9.910825	5.248363	0.820455054	low
MB-0414	6.298630137	0	10.155926	5.049254	5.728615	4.770619	4.669449	1.438156131	high
MB-0259	0.975342466	1	10.572754	4.950329	5.866059	4.481244	6.962577	1.573915246	high
MB-0526	11.47671233	1	8.911891	6.501524	6.386616	5.773261	5.776373	1.030803954	high
MB-0586	6.347945205	1	6.102372	6.057564	5.696431	8.730576	4.59854	0.791237698	low
MB-0288	5.243835616	1	8.208775	7.143427	6.021362	8.701794	6.814573	0.89638552	low
MB-0582	1.276712328	1	8.921132	4.520558	6.753144	4.774651	11.545211	1.345705789	high
MB-0469	10.7890411	0	9.718046	5.644803	5.803347	9.313343	5.324479	1.137037085	high
MB-0478	10.8739726	0	6.09519	7.811106	5.909829	11.614908	4.450267	0.606741138	low
MB-0421	7.643835616	1	8.363399	6.973502	5.65709	4.348734	11.417107	1.24522267	high
MB-0149	4.249315068	1	9.803759	4.516518	5.559312	4.528423	11.607136	1.750869408	high
MB-0512	9.989041093	0	6.888585	7.12745	6.024082	5.036869	5.360629	0.863295943	low
MB-0495	5.901369863	1	7.31991	7.49791	5.917903	5.385981	4.956509	0.869480997	low
MB-0661	1.643835616	1	7.018251	7.04686	6.440887	4.253862	4.313832	0.831324773	low
MB-0125	0.104109589	0	6.758462	5.99234	7.16964	4.037524	4.571109	0.804167306	low
MB-0487	7.142465753	0	5.339424	6.841509	6.101585	11.763419	9.783275	0.664716518	low
MB-0502	6.747945205	0	7.202659	5.014228	5.935506	12.514282	4.921757	0.819036341	low
MB-0289	5.884931507	1	7.203584	5.194589	6.418943	12.566951	5.203802	0.758118654	low
MB-0129	3.169863014	1	7.690572	7.196984	5.251564	6.444743	11.092782	1.124377178	high
MB-0458	11.05479452	0	7.373269	8.25575	6.611648	9.376898	6.682517	0.680805661	low
MB-0570	22.37260274	0	8.606382	4.398834	5.716351	4.186711	4.389378	1.321767524	high
MB-0484	6.791780822	0	8.538671	5.103265	6.158812	4.766296	4.744417	1.155449238	high
MB-0436	6.205479452	1	8.285276	6.648884	5.555501	4.012832	7.688868	1.194368701	high
MB-0227	9.194520551	0	7.532281	7.728659	6.909484	8.486028	4.995759	0.684483277	low
MB-0353	3.438356164	1	7.138678	5.304973	7.314252	5.488183	5.406113	0.837087273	low
MB-0522	0.205479452	0	8.363399	6.06683	6.37445	8.39334	11.365628	1.05403293	high
MB-0293	5.290410959	0	5.575197	8.128186	5.937048	10.178868	8.481973	0.644842003	low
MB-0256	16.49589041	0	5.192507	8.205157	7.581394	5.657083	5.257876	0.529334291	low
MB-0124	9.715068493	0	7.969185	7.409025	6.348242	9.456008	7.774396	0.816831563	low

MB-0365	7.169863014	1	8.531559	7.062913	5.50834	8.063084	9.3511	1.082323788	high
MB-0290	16.4	0	5.270174	8.021857	6.941869	5.955509	7.551715	0.617427422	low
MB-0113	3.547945205	0	8.436642	5.754007	6.574705	5.410472	7.245184	1.063112649	high
MB-0620	9.271232877	0	5.341201	7.322034	6.694042	10.63191	6.374239	0.56629365	low
MB-0282	15.96438356	0	5.481188	6.319786	6.579023	9.476658	4.690471	0.630422216	low
MB-0228	0.890410959	1	5.811608	7.035824	6.55436	10.855744	6.060695	0.608921694	low
MB-0578	9.049315068	0	5.903675	7.612197	6.611227	9.986788	6.868869	0.611121097	low
MB-0509	9.476712329	0	8.982342	5.11423	6.607737	9.304339	4.708235	0.971852964	low
MB-0279	15.80273973	1	8.051717	8.072582	5.656751	8.808745	5.65915	0.83966541	low
MB-0168	10.09041096	0	7.170885	7.748932	5.358568	9.081287	4.574958	0.797414882	low
MB-0588	9.841095888	0	6.893581	7.280035	5.571703	8.122354	11.071057	0.934805095	low
MB-0554	9.136986304	1	6.640113	7.014491	5.796668	4.561171	6.445763	0.914189993	low
MB-0193	1.561643836	0	8.094903	6.636185	6.246395	4.204278	10.996836	1.140786042	high
MB-0188	2.57260274	1	8.930366	5.30023	6.909484	5.18513	11.120616	1.211984559	high
MB-7225	4.520547945	1	10.545923	5.474634	5.822353	4.285263	9.337882	1.611441439	high
MB-7234	18.2630137	0	7.94711	4.610219	5.191118	7.046897	3.988279	1.18392213	high
MB-7089	9.77260274	0	9.329672	5.475681	5.501919	6.536003	9.57805	1.398669263	high
MB-7182	4.065753425	1	9.590265	4.490697	5.876973	8.497656	4.181303	1.216564691	high
MB-7039	4.043835616	1	7.666846	7.475627	5.48707	8.376336	5.692535	0.8809902	low
MB-3797	18.76712328	0	7.713559	7.330825	6.195388	4.364768	4.473633	0.900236955	low
MB-0308	15.0630137	0	6.069653	7.277625	5.400047	8.619859	5.44938	0.765835025	low
MB-7230	15.00821918	0	7.24554	5.914069	7.301376	7.10024	4.836003	0.75629569	low
MB-5452	1.328767124	1	7.605557	5.358227	6.688643	6.678866	4.653341	0.899881803	low
MB-6195	16.62191781	0	6.591884	8.85299	6.82511	10.222292	4.609979	0.542677956	low
MB-6317	2.402739726	1	8.488574	5.507447	7.234818	7.543457	4.373701	0.865956493	low
MB-5464	9.578082189	1	6.415398	5.558468	6.077661	6.414071	4.291414	0.861509259	low
MB-4820	4.709589041	1	7.041981	6.587688	7.369743	6.296379	4.835162	0.716122503	low
MB-5527	14.84109589	0	8.650163	5.678211	6.230969	6.8374	10.02888	1.164012325	high
MB-5453	4.709589041	1	6.607584	7.060141	7.359303	8.617447	7.190659	0.647604329	low
MB-5471	15.26849315	0	7.813436	5.276363	6.61383	7.468231	4.727314	0.911053926	low
MB-4313	24.71506849	1	7.518767	4.57706	8.273355	8.162861	5.302103	0.73244409	low
TCGA-A1-A0SM	0.663013699	0	8.504426	6.934159	5.912494	3.635173	6.261084	1.112418123	high
TCGA-E9-A22H	3.375342466	0	8.570873	7.156122	7.417278	5.781756	3.936184	0.786312779	low
TCGA-BH-A18S	5.504109589	1	5.895022	7.513918	7.195546	5.693187	4.866502	0.624541457	low
TCGA-BH-A0BR	6.383561644	0	8.407466	5.246035	5.695689	8.914773	10.925931	1.207514144	high
TCGA-A7-A0CD	3.191780822	0	7.709208	7.112777	6.570563	7.304542	5.081474	0.798138103	low
TCGA-D8-A27W	1.021917808	0	9.495542	6.720239	6.161228	5.66727	3.760304	1.060971985	high
TCGA-A2-A0EU	2.857534247	0	8.246341	5.102846	6.632582	7.621582	4.873104	0.958452922	low
TCGA-E2-A15F	1.802739726	0	8.054428	6.708352	6.3958	7.822495	3.755995	0.832210134	low
TCGA-E2-A1LA	2.049315068	0	9.248165	4.861466	5.556161	7.046128	5.712651	1.30039138	high
TCGA-EW-A1P4	2.484931507	0	9.956905	5.287914	5.265248	5.112083	3.822207	1.433907646	high
TCGA-BH-A0BQ	6.178082192	0	6.656078	6.263509	6.808668	8.559796	8.055531	0.764049942	low
TCGA-E2-A14P	3.41369863	0	10.519432	4.692116	5.860285	9.559982	11.129038	1.48434227	high
TCGA-BH-A1F5	7.430136986	1	7.233456	7.032288	7.163798	5.973722	4.908285	0.734635188	low
TCGA-A8-A08F	2.750684932	0	9.412174	5.584458	5.94789	4.001483	9.103907	1.413061414	high
TCGA-A7-A0CJ	2.550684932	0	9.024025	4.409155	6.101178	3.617056	3.553809	1.302373656	high
TCGA-C8-A12W	1.054794521	0	9.775831	4.895499	6.022145	6.335699	6.651584	1.336739754	high
TCGA-A7-A4SB	1.145205479	0	8.373058	5.071523	5.739635	8.010106	4.603059	1.082138165	high
TCGA-E2-A14Q	3.18630137	0	7.898785	5.027634	6.573526	7.789001	7.412244	0.990359956	low
TCGA-BH-A0AU	5.243835616	0	8.542716	5.536012	6.135538	8.110565	4.086088	0.988622864	low
TCGA-AR-A24X	8.230136986	0	7.393839	7.00803	7.410263	8.131655	4.500015	0.665776489	low
TCGA-B6-A0WV	6.621917808	1	9.280264	4.82996	6.391023	7.793634	4.605425	1.105796748	high
TCGA-AR-A24P	0.230136986	0	7.799875	7.660464	7.13939	5.170693	7.116371	0.800940159	low
TCGA-E2-A154	1.619178082	0	7.951717	7.035309	7.085002	3.675791	9.835713	0.960916252	low
TCGA-AO-A125	9.468493151	0	7.694162	5.266294	6.873184	3.820258	5.263309	0.993103168	low
TCGA-AC-A2FO	6.178082192	0	7.083593	6.699693	6.428238	9.375759	5.29546	0.742409274	low
TCGA-BH-A18V	4.263013699	1	9.8157	6.021239	6.342492	7.45912	9.356377	1.20453687	high
TCGA-E2-A1L7	5.030136986	0	10.259139	4.471846	6.550842	8.076091	4.207847	1.199225597	high

TCGA-E2-A15S	1.17260274	0	9.165055	4.679091	6.3667	7.65402	6.319334	1.159660515	high
TCGA-AC-A3EH	0.539726027	1	7.664681	6.926504	6.371577	4.463735	8.116469	0.976374156	low
TCGA-A2-A4RY	1.775342466	0	6.371048	6.842114	6.613235	9.23054	7.859809	0.711398011	low
TCGA-AO-A1KS	0.95890411	0	9.025262	6.184982	6.68499	7.156275	3.721352	0.933326681	low
TCGA-AR-A1AP	7.824657534	0	8.530773	6.397883	5.834903	8.200061	10.891705	1.121711495	high
TCGA-B6-A0RG	5.704109589	0	8.737104	4.487427	5.990224	4.862763	5.606865	1.285739979	high
TCGA-E2-A15D	1.44109589	0	6.043868	6.936107	6.948113	10.879768	4.824066	0.576755848	low
TCGA-AC-A3HN	1.35890411	0	7.444487	7.773464	6.380296	9.750602	5.370654	0.704685874	low
TCGA-LD-A66U	1.769863014	0	7.305175	6.707168	6.410449	8.446411	6.253681	0.801011334	low
TCGA-AO-A124	9.605479452	0	7.98963	5.074227	6.297371	3.472415	9.593336	1.255439757	high
TCGA-A2-A0CR	8.994520548	0	7.773801	6.204015	5.571432	9.111259	7.158526	0.979044139	low
TCGA-A2-A3XT	7.589041096	0	9.926797	4.735438	6.153061	9.321111	7.83096	1.253868876	high
TCGA-B6-A0X0	10.80821918	1	7.597549	6.077494	7.033284	4.579819	9.414274	0.968133683	low
TCGA-B6-A0IK	1.564383562	1	9.127643	6.20703	4.716305	8.173468	5.69252	1.255419763	high
TCGA-A2-A0T7	1.728767123	0	7.970443	6.495792	6.235658	6.364451	11.240818	1.067994409	high
TCGA-AR-A0U4	8.934246575	0	9.609015	6.752438	5.766508	9.242969	10.439172	1.168952122	high
TCGA-A2-A0D0	5.610958904	0	11.505844	5.212806	5.540904	3.376309	6.583641	1.817029919	high
TCGA-EW-A3U0	1.457534247	0	8.838012	5.323102	6.276046	10.103965	9.993713	1.085065009	high
TCGA-C8-A27B	1.202739726	0	9.202929	5.489707	6.007665	3.609061	4.3506	1.257991088	high
TCGA-LQ-A4E4	2.326027397	0	8.728612	5.505517	6.034274	9.288277	11.866069	1.174370133	high
TCGA-BH-A5J0	1.95890411	0	8.446488	5.740117	6.125648	7.763774	10.580608	1.132038108	high
TCGA-E2-A14Z	1.542465753	1	8.60491	6.368748	6.365804	8.005745	5.455393	0.934627101	low
TCGA-AR-A1AN	8	0	7.205691	5.722622	6.247295	10.198824	4.135785	0.787541649	low
TCGA-B6-A0RM	6.501369863	1	7.989424	5.798517	6.470671	6.608787	4.590885	0.931582408	low
TCGA-B6-A0X7	4.879452055	1	6.87447	6.599497	6.378601	9.458923	4.931686	0.730285968	low
TCGA-B6-A0X5	5.745205479	1	9.379948	5.133563	3.596613	4.557071	10.633211	2.064707617	high
TCGA-BH-A18K	7.569863014	1	8.522004	4.754669	6.626519	7.773143	9.027338	1.106560473	high
TCGA-AR-A0U2	6.989041096	1	10.644187	4.68106	6.422634	5.294722	9.125303	1.529501733	high
TCGA-A7-A3J0	0.857534247	0	7.823553	6.591348	6.700089	6.052426	4.372558	0.846295389	low
TCGA-GM-A3NW	9.208219178	0	7.275766	5.734524	6.173976	8.366862	5.083439	0.869169486	low
TCGA-BH-A0HI	1.698630137	0	7.282916	7.927946	6.849452	6.249631	7.327806	0.753738571	low
TCGA-E2-A14U	3.610958904	0	4.712075	6.098288	6.442974	7.967904	6.518258	0.665281896	low
TCGA-A2-A0EO	6.690410959	0	7.084766	6.892807	6.192847	8.097804	6.522905	0.811307317	low
TCGA-B6-A0IQ	11.73972603	0	9.921956	4.418091	6.325612	3.523156	9.765885	1.588437741	high
TCGA-AR-A5QQ	0.882191781	1	10.068507	4.551018	5.484358	7.15445	11.704768	1.662672336	high
TCGA-LL-A440	2.079452055	0	6.980473	6.828263	5.868393	11.175625	6.192547	0.757046263	low
TCGA-AC-A2QJ	1.221917808	1	8.712903	5.451612	4.142066	7.015636	10.729716	1.615581897	high
TCGA-BH-A1EW	4.64109589	1	8.274072	5.47785	6.739799	7.797402	6.017004	0.938300132	low
TCGA-BH-A201	2.345205479	0	7.551986	5.93856	6.560594	9.585128	4.890428	0.795562038	low
TCGA-A2-A3XU	2.498630137	1	8.442244	4.471622	5.856422	5.570794	4.754946	1.221485886	high
TCGA-AR-A24V	8.775342466	0	7.428679	7.041592	6.532755	4.101084	6.934785	0.911143736	low
TCGA-AO-A12D	6.890410959	0	7.398487	6.85744	6.082939	4.551012	10.320154	1.044557304	high
TCGA-A8-A08I	1	0	8.82906	5.636546	6.253203	4.829641	3.340824	1.087625709	high
TCGA-AR-A24W	4.246575342	0	5.617014	7.182029	6.323307	9.648506	6.604039	0.643340574	low
TCGA-C8-A3M7	2.832876712	1	7.951295	6.04583	6.612844	8.615187	11.889714	0.98556242	low
TCGA-E2-A15R	4.745205479	0	8.502727	7.162857	7.109435	6.106923	3.828317	0.804616421	low
TCGA-A2-A0CK	11.39452055	0	8.050206	6.816349	6.159455	9.699877	6.8199	0.859046304	low
TCGA-AN-A0FD	0.536986301	0	8.330638	5.573684	5.539039	3.735506	6.276722	1.276981515	high
TCGA-AR-A0TZ	8.936986301	1	8.961865	6.811594	6.047036	5.046505	5.067036	1.069394437	high
TCGA-A8-A085	3.079452055	0	7.919504	4.420342	5.945346	4.608825	4.093027	1.171219114	high
TCGA-A2-A0YH	1.805479452	0	8.738664	6.107872	6.752524	4.883362	7.519264	1.063638911	high
TCGA-AR-A1AT	3.484931507	1	8.723427	5.513282	6.248236	6.37475	9.2839	1.182583536	high
TCGA-BH-A5IZ	1.553424658	0	9.184401	4.276156	6.514595	4.91515	10.195193	1.404159241	high
TCGA-EW-A1J1	1.575342466	0	7.785461	6.883943	7.260175	4.482535	4.681837	0.808294597	low
TCGA-AC-A2FK	7.260273973	0	4.978123	7.792335	6.541334	8.693683	7.804103	0.593616324	low
TCGA-AQ-A1H3	2.709589041	0	7.617857	6.736464	7.066058	9.736102	7.60543	0.74191459	low
TCGA-A8-A07O	0.832876712	0	9.640572	5.42808	5.865352	3.705737	9.99732	1.524438034	high
TCGA-BH-A1ES	9.484931507	1	8.202003	5.959459	6.144928	4.801953	4.139986	1.033470741	high

TCGA-A7-A4SA	1.243835616	0	8.958544	6.22398	5.939243	9.182213	5.888091	1.008511157	high
TCGA-E2-A1II	2.808219178	0	9.566415	5.549926	6.938295	4.668082	6.007745	1.140256064	high
TCGA-D8-A27K	4.002739726	0	6.685325	8.08975	6.940133	6.818029	6.163677	0.663012428	low
TCGA-AC-A6IX	1.021917808	0	7.244019	7.980703	6.039214	10.233251	4.603252	0.6901948	low
TCGA-E9-A1R7	4.019178082	0	8.138602	4.754324	5.939819	7.67391	5.986467	1.099933805	high
TCGA-AC-A5XU	1.246575342	0	8.919329	6.187118	5.880516	6.758182	5.806186	1.099178611	high
TCGA-BH-A0B4	3.263013699	0	8.895898	5.505724	5.217804	6.090599	5.209728	1.280106937	high
TCGA-BH-A0C1	3.865753425	1	8.01865	6.528273	6.563742	6.023082	4.825077	0.893386763	low
TCGA-A7-A0DA	2.97260274	0	8.544267	5.687393	6.065434	3.901827	11.086022	1.330980217	high
TCGA-C8-A12U	1.054794521	0	10.006025	4.282291	6.100884	5.097607	7.845035	1.517400152	high
TCGA-LL-A740	1.208219178	0	6.99383	6.753202	5.633297	5.987267	11.044368	1.045675768	high
TCGA-B6-A0RE	21.30684932	0	9.88717	4.220758	6.377099	6.383906	11.670744	1.515188706	high
TCGA-E2-A1IF	3.117808219	0	8.275917	6.036234	6.28334	9.167873	11.953924	1.04812019	high
TCGA-OL-A5RU	3.339726027	0	6.19831	5.364669	6.433884	8.55891	5.026037	0.771170149	low
TCGA-A7-A3IZ	0.882191781	0	8.360539	6.503639	6.633391	6.080526	4.099797	0.899362428	low
TCGA-E9-A229	3.145205479	0	8.508771	6.554233	7.074285	5.997114	4.495375	0.864130813	low
TCGA-D8-A142	1.164383562	0	8.738528	5.557507	5.722706	5.800356	9.26374	1.294879217	high
TCGA-E9-A1NC	3.295890411	0	8.042473	5.255155	5.429241	6.804756	11.688959	1.320099009	high
TCGA-EW-A2FR	4.583561644	0	7.699127	5.654314	5.977917	8.267367	9.185276	1.031423544	high
TCGA-EW-A2FW	1.84109589	0	9.060117	5.452034	6.680802	6.843061	4.500231	1.019878672	high
TCGA-D8-A3Z5	2.780821918	0	6.221533	6.692045	6.059524	9.668978	7.635804	0.752002651	low
TCGA-LL-A7SZ	1.62739726	0	8.255567	4.093372	5.000955	5.518954	4.872953	1.399624231	high
TCGA-AR-A2LE	13.86849315	0	6.314584	6.348709	6.084568	6.779739	4.17832	0.790146411	low
TCGA-BH-A0W4	2.079452055	0	7.655655	5.677797	6.956258	10.252826	4.108434	0.745068249	low
TCGA-A7-A26E	2.61369863	0	6.338276	8.374648	6.779192	6.971362	4.042012	0.608308648	low
TCGA-C8-A12Y	4.043835616	0	8.819462	4.925251	7.149568	6.175171	4.00549	0.98226123	low
TCGA-AC-A3W5	1.380821918	0	8.720171	7.058256	5.788249	8.895468	11.441643	1.081177284	high
TCGA-A8-A08Z	3.334246575	0	7.579245	6.980531	7.164308	8.064173	6.833829	0.743138339	low
TCGA-AO-A1KO	1.704109589	0	4.602859	5.737274	6.296458	9.306222	7.543035	0.676538099	low
TCGA-A2-A0YJ	1.550684932	0	9.363681	4.1409	5.431821	3.695722	11.157136	1.7898257	high
TCGA-AO-A03N	5.564383562	0	9.052221	4.37174	5.735233	4.869128	9.417701	1.511289639	high
TCGA-BH-A1ET	6.904109589	1	5.899718	7.924006	7.285388	7.726905	4.225184	0.550558166	low
TCGA-E2-A576	2.857534247	0	8.926332	4.852481	6.087876	8.859324	7.893035	1.157974375	high
TCGA-AO-A12E	5.868493151	0	8.637839	7.727144	6.474474	9.687412	9.032202	0.852726724	low
TCGA-A8-A09E	4.087671233	0	8.912008	5.041161	5.909075	8.4295	4.599873	1.100059312	high
TCGA-A1-A0SN	3.276712329	0	8.731656	5.97076	6.137101	5.70302	10.591165	1.22244955	high
TCGA-JL-A3YW	0.98630137	0	8.798538	4.649197	5.037879	7.226318	11.527933	1.544767146	high
TCGA-E2-A14X	2.663013699	0	8.78191	5.493997	6.65231	7.609121	11.463402	1.134720028	high
TCGA-A8-A08G	1.663013699	0	9.168758	5.106211	6.302868	7.353138	9.079293	1.21799502	high
TCGA-A8-A07J	1	0	6.463586	8.87127	6.476923	11.765714	3.268663	0.518000912	low
TCGA-BH-A0B8	4.298630137	0	7.757061	6.775814	6.024263	8.564018	4.539538	0.841920617	low
TCGA-D8-A73X	2.101369863	0	7.046077	7.24552	7.110691	5.466723	6.354521	0.75147665	low
TCGA-AC-A2QI	1.610958904	0	6.071921	8.710612	6.189107	6.737485	8.347963	0.697707468	low
TCGA-C8-A130	1.01369863	0	9.473727	4.99051	5.568297	6.941601	4.56332	1.284148622	high
TCGA-A1-A0SB	0.709589041	0	6.80668	7.027857	6.455404	7.489834	10.88946	0.848827919	low
TCGA-AC-A7VB	0.684931507	0	8.867586	4.607041	6.672716	8.777564	4.91472	1.012698455	high
TCGA-D8-A1XJ	1.819178082	0	8.856042	5.393441	6.428522	4.482162	11.922717	1.332758107	high
TCGA-D8-A1JC	1.315068493	0	9.362446	5.892731	6.220549	4.951043	4.847085	1.162334974	high
TCGA-E9-A1ND	3.468493151	0	8.817965	5.375271	5.927323	3.637974	9.013235	1.373752244	high
TCGA-AR-A1AR	1.435616438	1	8.961395	6.171017	7.018294	10.338507	3.929764	0.800818524	low
TCGA-B6-A408	5.676712329	0	7.488076	5.606854	7.62807	4.377099	6.99265	0.870521621	low
TCGA-D8-A1Y0	1.293150685	0	8.989973	8.281896	6.61056	6.208118	7.037971	0.889585592	low
TCGA-D8-A27E	1.452054795	0	5.580068	8.013522	7.008944	4.588335	3.279711	0.598858689	low
TCGA-A8-A06U	2.419178082	1	8.832049	5.295401	5.782495	4.799781	5.405468	1.25214438	high
TCGA-AC-A2FG	5.076712329	0	6.037465	7.455386	6.665282	8.834135	5.988572	0.633348742	low
TCGA-A8-A09A	0.832876712	0	7.855596	6.479023	5.799348	7.889091	6.485057	0.959538041	low
TCGA-D8-A1XC	1.032876712	1	6.563927	7.507201	6.382219	3.737096	3.480867	0.772918477	low
TCGA-B6-A1KI	6.126027397	0	6.376673	6.550591	6.484025	8.72988	4.569176	0.699501965	low

TCGA-AR-A1AJ	6.528767123	0	9.500771	6.277579	6.632082	9.812849	5.733465	0.936628857	low
TCGA-A7-A4SF	1.493150685	0	9.661369	4.783527	6.088411	4.545164	7.988518	1.445470546	high
TCGA-A2-A3XW	4.690410959	0	7.571352	6.942519	4.8237	5.533326	11.897321	1.263967451	high
TCGA-V7-A7HQ	5.569863014	0	7.264004	5.928692	5.998977	8.478834	9.332616	0.9618828	low
TCGA-E2-A107	2.868493151	0	8.381984	6.010197	6.358624	7.589859	12.40688	1.117842804	high
TCGA-E9-A1RH	3.882191781	0	9.631049	4.724267	6.097318	4.725198	6.353451	1.384988599	high
TCGA-AR-A5QM	6.112328767	0	8.054527	6.914446	6.113664	9.651725	8.827012	0.899935838	low
TCGA-A8-A09I	3.756164384	0	9.995579	5.001998	6.273818	5.915858	4.97199	1.27575332	high
TCGA-AC-A3QQ	2.010958904	0	6.305196	7.075053	5.907305	6.642107	3.169764	0.751322828	low
TCGA-A7-A426	0.997260274	0	7.382798	6.868269	6.310373	9.424747	6.790164	0.792086306	low
TCGA-BH-A0DZ	1.356164384	0	9.176331	5.54566	5.856806	5.795648	5.150992	1.208470206	high
TCGA-A2-A0YC	2.712328767	0	7.645484	6.451576	6.999437	9.095873	3.663718	0.71695191	low
TCGA-B6-A0RH	17.68767123	1	8.720064	4.928225	6.777251	4.495381	11.254602	1.278038942	high
TCGA-AO-A1KP	8.090410959	0	8.84519	5.21302	7.135715	5.393041	3.593515	0.981133096	low
TCGA-OL-A6VQ	1.643835616	0	6.849838	6.29434	6.593542	8.321245	6.149109	0.772669756	low
TCGA-B6-A0WZ	17.23835616	0	7.909977	5.058854	7.030555	4.318539	5.570762	0.998162215	high
TCGA-A2-A0SY	3.690410959	0	7.544076	7.01018	6.112659	9.545787	4.460291	0.772894735	low
TCGA-A8-A09D	4.169863014	0	8.394622	5.87501	5.632355	10.699266	5.462302	0.964797591	low
TCGA-A8-A09Q	2.084931507	0	8.296811	5.157135	5.920096	7.201382	5.498767	1.090677708	high
TCGA-E2-A15E	1.726027397	0	7.520046	6.190982	6.117376	7.249804	7.182816	0.942617066	low
TCGA-AC-A2BM	8.279452055	0	8.310528	5.94634	6.760024	6.389113	3.52644	0.896755402	low
TCGA-D8-A1JT	1.109589041	0	8.781099	4.878016	7.820457	3.616224	3.821519	0.968949844	low
TCGA-AC-A3BB	2.704109589	0	6.973202	7.09539	6.365945	10.378303	7.574685	0.73210162	low
TCGA-AR-A1AQ	8.276712329	0	9.759879	4.892723	6.380362	3.789501	7.09173	1.395316653	high
TCGA-E9-A1N5	3.068493151	0	8.263022	6.221578	6.239843	8.462528	3.948501	0.88612764	low
TCGA-A8-A06T	4.421917808	0	7.137159	5.428544	7.333994	7.412164	4.247004	0.755314757	low
TCGA-AQ-A04L	10.84109589	0	7.311231	5.839074	5.535193	4.459165	5.526939	1.088870069	high
TCGA-BH-A0B7	7.010958904	0	7.999397	6.506663	5.257385	9.115381	11.373064	1.124199298	high
TCGA-AO-A0J2	2.731506849	0	9.583759	6.330989	5.314052	5.291027	8.666143	1.40648884	high
TCGA-A8-A06Z	0.084931507	0	8.080058	5.294294	6.389365	5.137706	5.515822	1.05993815	high
TCGA-AR-A1AS	3.150684932	0	8.063494	5.82974	6.906551	5.762214	10.864865	1.044124293	high
TCGA-A2-A25A	8.975342466	0	7.784145	7.922898	6.522088	7.78709	7.713867	0.79435373	low
TCGA-A2-A3XX	3.942465753	1	9.883499	4.63938	5.753064	5.813309	8.634664	1.523090543	high
TCGA-OL-A97C	0.742465753	0	8.747203	4.874173	6.109782	6.738399	3.899469	1.110065001	high
TCGA-4H-AAAK	0.953424658	0	7.847835	7.050917	6.801144	6.754339	7.940962	0.855101786	low
TCGA-BH-A0DX	5.906849315	0	8.194434	5.147552	5.928979	11.392513	4.360244	0.915001435	low
TCGA-EW-A6SC	2.608219178	0	8.585251	6.441835	6.446109	8.382779	5.792226	0.912702137	low
TCGA-GM-A3XN	5.531506849	0	7.210378	5.677782	6.220434	10.766062	6.105434	0.814448365	low
TCGA-A8-A07G	1.580821918	0	6.624315	7.212014	5.967836	5.559103	7.710214	0.873660457	low
TCGA-A2-A1G6	1.37260274	0	4.773982	8.020257	6.7711	9.536307	5.117908	0.506494031	low
TCGA-A2-A0T6	1.575342466	0	5.532987	8.595781	6.375748	10.042927	3.584674	0.523348955	low
TCGA-D8-A1JU	1.224657534	0	4.857598	7.579179	6.499819	10.911915	3.685903	0.507540759	low
TCGA-A7-A3IY	0.945205479	0	7.687666	8.757509	6.597537	5.881824	6.778541	0.761454686	low
TCGA-A8-A0A9	2.252054795	0	8.811427	5.681267	6.280509	7.302622	6.126322	1.057656298	high
TCGA-A1-A0SP	1.6	0	10.141685	4.628601	6.580943	7.544834	7.65188	1.283935858	high
TCGA-EW-A1IZ	1.517808219	0	8.430457	5.612265	6.440936	6.57143	6.520313	1.035950187	high
TCGA-A1-A0SE	3.619178082	0	8.199722	6.467572	6.852658	7.43932	6.788496	0.874794039	low
TCGA-BH-A1FH	2.832876712	1	6.077939	7.544575	6.269077	8.195486	5.240813	0.670547699	low
TCGA-D8-A27M	1.123287671	0	8.554915	6.397559	5.873595	8.999998	10.238696	1.072848623	high
TCGA-EW-A1PD	1.161643836	0	8.620535	6.070052	5.817696	9.924144	4.174667	0.942977425	low
TCGA-AR-A0TQ	8.194520548	0	9.45204	4.968701	6.252533	8.233949	9.865363	1.259615365	high
TCGA-E2-A56Z	0.690410959	0	9.405872	5.246949	8.772727	7.093909	5.84208	0.815723505	low
TCGA-A8-A07B	3.583561644	0	8.042069	5.399128	6.036593	3.867032	3.169764	1.08881421	high
TCGA-A7-A4SC	1.221917808	0	7.047886	7.038735	6.318697	8.97642	4.514365	0.728676119	low
TCGA-LL-A6FQ	0.219178082	0	8.281533	4.838645	5.626239	7.890334	6.984351	1.176063067	high
TCGA-B6-A0RU	23.57534247	0	9.597964	7.130531	6.616075	8.758861	10.009658	1.012571626	high
TCGA-AR-A24H	13.40821918	0	9.609524	4.69031	7.855422	7.745688	6.099868	0.9727411	low
TCGA-EW-A1P1	3.315068493	0	7.217294	6.050231	5.956699	11.705005	9.743837	0.865247545	low

TCGA-AC-A3QP	1.849315068	0	6.396835	8.46619	6.357601	8.453527	7.166494	0.658760802	low
TCGA-D8-A145	1.123287671	0	7.109633	7.306639	6.576764	8.441415	6.251769	0.733190958	low
TCGA-AO-A0JJ	5.169863014	0	7.885333	8.719302	6.284514	9.433269	7.062676	0.727557912	low
TCGA-B6-A1KC	3.632876712	0	7.760682	5.917586	6.282975	5.465104	3.343923	0.936143121	low
TCGA-AO-A03V	3.701369863	0	7.693317	5.695571	6.050454	9.155579	7.269747	0.945452731	low
TCGA-A7-A13E	1.682191781	1	8.545863	5.302646	6.677849	7.492274	8.244157	1.046592807	high
TCGA-AN-A0FF	0.471232877	0	8.379064	7.17425	6.796845	8.81648	4.696988	0.773736505	low
TCGA-B6-A3ZX	3.156164384	1	8.782321	5.989087	6.006576	5.168198	10.684436	1.274582372	high
TCGA-E2-A10E	2.369863014	0	7.786812	4.94714	6.592792	9.474147	5.637493	0.892839627	low
TCGA-OL-A66N	2.169863014	0	6.010587	5.933986	5.711154	7.282876	8.284371	0.901553555	low
TCGA-A7-A0D9	3.120547945	0	6.535119	5.656941	6.539835	9.159645	4.541751	0.74389603	low
TCGA-E9-A54Y	1.98630137	0	10.11929	3.703892	5.849524	3.66987	6.617314	1.694841525	high
TCGA-E9-A1N3	2.901369863	0	7.340484	6.740356	6.8374	9.073675	3.322776	0.691669991	low
TCGA-C8-A12X	1.054794521	0	8.137049	6.211708	5.78351	6.116878	4.389506	1.020233023	high
TCGA-A8-A07U	2.082191781	0	9.318061	5.334357	6.045188	9.299568	8.723316	1.170536796	high
TCGA-E2-A10B	3.126027397	0	8.297495	7.292079	7.051527	8.066514	5.183667	0.760811369	low
TCGA-AO-A03U	4.912328767	1	6.735352	9.200705	5.872047	5.51169	3.532104	0.699178681	low
TCGA-A8-A075	1.419178082	0	9.069984	4.624176	5.724356	7.15404	6.496532	1.289615573	high
TCGA-EW-A1OY	2.487671233	0	9.901906	4.956261	6.037895	5.145035	6.789358	1.402426366	high
TCGA-Z7-A8R6	8.920547945	0	9.881903	4.471814	6.352113	7.305997	5.594288	1.259120537	high
TCGA-GM-A2DM	8.838356164	0	6.388065	7.962713	7.103173	5.267782	3.169764	0.62546449	low
TCGA-C8-A26W	1.043835616	0	9.460162	7.68394	6.787028	7.820869	5.933918	0.87892942	low
TCGA-AQ-A7U7	1.6	1	7.617134	8.65394	6.449458	6.026625	5.360884	0.750140173	low
TCGA-BH-A0E7	3.734246575	0	6.691389	6.553264	7.056856	5.151311	3.970806	0.738941495	low
TCGA-A7-A13F	2.095890411	0	9.846	6.196449	6.767023	5.917219	4.492634	1.058051457	high
TCGA-BH-A0HQ	3.071232877	0	7.268436	6.918703	5.930185	5.964925	5.470712	0.896391146	low
TCGA-B6-A0RN	21.93972603	0	6.031788	6.686137	6.678359	7.290103	5.197188	0.693267637	low
TCGA-AC-A2B8	1.854794521	0	6.767898	7.762263	6.603045	10.107457	3.598762	0.607812258	low
TCGA-A1-A0SI	1.739726027	0	9.005858	6.660275	6.164104	6.328877	10.748634	1.1653201	high
TCGA-E2-A14W	2.668493151	0	8.827429	5.155669	6.273294	3.526011	3.597862	1.182165825	high
TCGA-D8-A1X7	1.394520548	0	7.475351	7.700643	7.41153	7.202406	4.587935	0.657229345	low
TCGA-LL-A5YN	1.224657534	0	8.609877	6.289116	5.923173	6.131593	6.393872	1.088746928	high
TCGA-D8-A1XY	1.378082192	0	6.070386	5.444896	6.962069	6.0488	11.990498	0.894891823	low
TCGA-A7-A0DB	2.75890411	0	6.775953	6.728811	6.481654	10.135622	7.245976	0.727429332	low
TCGA-E9-A22A	3.257534247	0	9.003809	6.224412	5.466744	5.518395	4.4158	1.182642155	high
TCGA-A2-A0T1	1.42739726	0	7.981969	5.02435	4.54865	8.19048	11.043209	1.423327245	high
TCGA-EW-A2FV	2.15890411	0	8.888403	5.716129	6.019159	7.752871	11.15016	1.217489591	high
TCGA-E9-A1R6	0.928767123	0	8.31558	6.516492	6.291272	4.216275	3.692058	0.989582091	low
TCGA-A7-A0DC	2.482191781	0	5.171658	4.484366	5.756073	4.651987	3.207964	0.898752992	low
TCGA-A2-A04Q	6.534246575	0	8.978223	5.434611	5.126155	5.667649	5.923805	1.354696802	high
TCGA-AC-A3W7	1.290410959	0	7.685915	7.345884	6.145469	8.011594	10.750633	0.922628375	low
TCGA-D8-A27N	1.421917808	0	8.234897	5.802449	5.443792	9.641541	10.122929	1.129768075	high
TCGA-EW-A1PA	1.575342466	0	9.508376	5.785855	6.179991	7.328626	5.025531	1.109002701	high
TCGA-B6-A0X1	20.42465753	1	9.548679	5.462121	7.57824	3.332448	7.913518	1.143275183	high
TCGA-E2-A156	1.989041096	0	7.06143	6.81154	7.646537	3.650397	3.654114	0.72096806	low
TCGA-B6-A0IH	8.123287671	1	5.233444	7.580719	6.649094	7.193407	5.40418	0.606299388	low
TCGA-E2-A15T	4.282191781	0	8.478742	5.050726	7.080732	7.388894	7.38251	0.985732939	low
TCGA-D8-A140	1.104109589	0	8.109151	5.371459	6.780797	7.704923	3.461985	0.876226957	low
TCGA-B6-A40C	5.928767123	0	8.440535	6.538937	6.025643	9.612769	6.016558	0.914503867	low
TCGA-BH-A1EU	3.523287671	1	6.415232	7.987141	7.034732	11.030839	3.813808	0.529459158	low
TCGA-A2-A0EV	2.652054795	0	7.690648	6.179106	6.411288	8.925249	7.679546	0.880240506	low
TCGA-EW-A1PH	1.663013699	0	9.717988	5.260158	6.479954	5.637879	7.129805	1.253660078	high
TCGA-E2-A1IN	1.849315068	0	7.987361	7.157125	6.648625	3.739487	5.516251	0.918842148	low
TCGA-LL-A73Z	0.621917808	1	8.26232	6.511576	5.840206	9.257911	11.506337	1.059957755	high
TCGA-A7-A2KD	1.860273973	0	9.576408	7.236472	6.577721	4.691087	8.586122	1.116900814	high
TCGA-A7-A13G	1.967123288	0	5.063749	7.524882	5.75256	8.002854	3.673161	0.636107795	low
TCGA-E2-A14N	3.928767123	0	9.796997	4.372138	6.306308	8.302173	4.802286	1.203523899	high
TCGA-C8-A132	1.049315068	0	8.12362	6.637163	6.610299	6.560223	7.117305	0.920022051	low

TCGA-A8-A06R	1.498630137	0	9.023783	4.659022	6.00138	5.678728	11.680129	1.455028264	high
TCGA-E2-A1LL	3.58630137	0	9.866701	4.339209	6.069563	8.261565	11.255384	1.456243901	high
TCGA-GI-A2C8	0.616438356	0	4.810085	8.549548	5.759005	4.405678	4.167148	0.653077851	low
TCGA-E9-A1N6	1.857534247	1	9.15529	4.958665	6.907419	6.579631	3.51838	1.021728998	high
TCGA-BH-A0E2	1.191780822	0	9.590287	6.089963	6.250605	6.76451	4.815752	1.096269394	high
TCGA-D8-A1XQ	1.367123288	0	8.842049	5.62623	6.124765	5.405509	9.257109	1.245368444	high
TCGA-AN-A0FJ	0.663013699	0	8.594832	5.317914	5.557464	4.759005	10.015616	1.40222833	high
TCGA-AR-A24Q	8.690410959	0	8.682428	7.419611	5.811747	4.358127	8.926346	1.146733028	high
TCGA-A8-A091	2.750684932	0	7.287964	7.808067	6.118021	7.611112	3.624828	0.741302964	low
TCGA-E9-A1RG	1.77260274	0	9.840439	4.673485	5.716858	7.09445	10.86292	1.532785332	high
TCGA-E2-A15J	4.493150685	0	7.428388	6.457355	7.276191	4.606799	11.330396	0.93275405	low
TCGA-B6-A0WY	9.482191781	1	8.545378	7.223366	6.42062	5.899196	5.513214	0.927075578	low
TCGA-C8-A8HR	1.117808219	0	8.765128	7.018917	5.690429	8.664274	5.806296	0.979057419	low
TCGA-BH-A28O	3.068493151	0	5.632163	6.588367	6.177557	9.777445	8.355705	0.713400289	low
TCGA-A2-A0ER	6.2	0	8.129113	6.694692	6.643902	6.586172	3.782654	0.844808539	low
TCGA-MS-A51U	1.865753425	0	7.302125	6.598526	6.965533	8.186954	5.677823	0.743463173	low
TCGA-A2-A0YI	4.123287671	0	6.042601	8.921598	6.618906	7.287468	4.688874	0.582127248	low
TCGA-C8-A12Z	1.046575342	0	8.510107	4.777813	6.488735	4.181231	11.178341	1.331170297	high
TCGA-PE-A5DD	5.350684932	0	8.467975	5.360823	6.181219	8.580001	7.178497	1.04405204	high
TCGA-A2-A3XV	2.728767123	0	7.760194	4.360724	4.608013	7.639958	12.009106	1.514021142	high
TCGA-AR-A24K	4.24109589	0	7.852463	6.637439	7.76435	3.905878	4.982741	0.792679123	low
TCGA-BH-A18F	2.742465753	0	8.989168	6.152011	6.989749	10.543134	6.881152	0.857537049	low
TCGA-BH-A42V	1.739726027	0	6.743212	6.66377	6.321504	9.874211	5.591921	0.724188054	low
TCGA-BH-A0HL	0.197260274	0	8.304604	4.816279	6.617272	5.548472	5.24597	1.067041279	high
TCGA-BH-A1EY	1.473972603	1	7.064326	6.008583	6.048232	8.003884	10.195994	0.96482065	low
TCGA-BH-A0DP	1.304109589	0	6.837238	7.712821	6.605772	8.777124	5.681121	0.672866179	low
TCGA-E2-A573	2.909589041	0	10.47832	5.444262	5.914231	5.65927	10.526867	1.554789194	high
TCGA-BH-A1FU	4.624657534	1	8.200232	6.528654	5.583753	5.815856	10.368771	1.192037685	high
TCGA-AO-A0J3	1.783561644	0	8.562439	4.739766	7.188435	5.86547	4.994133	0.99893669	high
TCGA-D8-A1XA	2.298630137	0	8.14066	5.832564	6.336404	7.574051	3.502349	0.907702691	low
TCGA-E2-A158	1.232876712	0	8.240673	3.995744	7.111816	4.223785	10.283494	1.234869672	high
TCGA-AR-A250	7.416438356	0	8.899182	5.028664	5.851215	4.013648	3.608861	1.255546601	high
TCGA-E2-A1LB	6.317808219	0	7.214995	4.896161	5.171305	5.006544	6.63368	1.229867513	high
TCGA-A2-A0CW	8.994520548	0	11.127615	4.681123	6.930352	5.500728	10.958756	1.54471747	high
TCGA-BH-A208	4.819178082	1	7.284804	6.3434	5.331029	5.948747	10.002411	1.132651512	high
TCGA-D8-A1XK	1.208219178	0	9.702296	4.652004	6.200305	5.713767	3.550274	1.254907724	high
TCGA-XX-A899	1.279452055	0	7.558814	6.356648	6.146273	10.48429	5.323573	0.800886734	low
TCGA-A2-A0EM	8.476712329	0	7.071211	7.809145	6.626688	6.984314	3.782384	0.692419782	low
TCGA-A2-A0SX	4.202739726	0	9.204191	5.549978	6.028938	9.332497	10.086795	1.175865368	high
TCGA-A1-A0SD	1.197260274	0	7.881825	6.693315	7.054273	8.972403	6.395112	0.763241014	low
TCGA-GM-A3XG	3.643835616	0	5.829909	5.25324	6.381388	7.515047	8.548819	0.848231447	low
TCGA-EW-A1J3	1.380821918	0	8.858112	5.891813	6.783455	7.486496	3.456496	0.910881722	low
TCGA-LL-A441	2.728767123	0	9.417654	6.647351	5.372521	8.333151	10.61729	1.265640732	high
TCGA-C8-A1HI	0.939726027	0	7.60963	6.683952	6.634485	5.174427	6.883047	0.905579602	low
TCGA-D8-A1JI	1.580821918	0	7.306199	7.842754	6.279978	6.710288	3.486	0.743578753	low
TCGA-E9-A248	0.161643836	0	8.926678	4.945131	6.602482	5.388034	10.695825	1.279131765	high
TCGA-C8-A12V	1.054794521	0	8.123225	6.383353	6.214033	6.529892	4.611753	0.938104241	low
TCGA-A7-A6VY	0.728767123	0	9.734117	4.558362	5.435608	3.863482	5.645815	1.57497367	high
TCGA-E2-A1IU	0.923287671	0	6.84471	7.019579	6.823858	6.680671	6.658745	0.755233734	low
TCGA-A2-A0YT	1.980821918	1	9.440529	5.342553	6.148247	6.130925	6.261619	1.225749265	high
TCGA-E9-A249	0.594520548	0	8.248075	5.315724	7.368427	7.863298	3.369653	0.814993631	low
TCGA-BH-A1FD	2.764383562	1	8.564577	5.681563	5.676841	4.559082	4.006738	1.173910067	high
TCGA-E9-A1R5	0.252054795	0	6.950469	6.016814	6.692442	8.31662	7.652801	0.813531355	low
TCGA-AO-A1KT	1.482191781	0	9.102791	6.310283	6.703319	4.909774	3.680535	0.999993507	high
TCGA-LL-A5YO	1.205479452	0	9.093465	5.762507	5.403742	5.832745	10.845347	1.428469579	high
TCGA-B6-A0IJ	19.46849315	0	10.516918	5.460575	5.377454	4.321267	11.235693	1.786076113	high
TCGA-EW-A1J5	1.306849315	0	8.268817	6.622956	7.145477	7.207546	3.37873	0.778696822	low
TCGA-AR-A2LR	4.77260274	0	9.657829	6.228399	6.338983	9.202598	8.081497	1.070809913	high

TCGA-A7-A26I	1.810958904	0	7.133676	5.438561	5.254052	4.122931	5.302361	1.155624741	high
TCGA-GM-A4E0	6.002739726	0	6.044713	6.887482	5.825324	11.633553	4.836018	0.661580727	low
TCGA-E2-A1IH	2.810958904	0	7.931019	6.795164	6.262558	9.401759	4.232559	0.798361247	low
TCGA-D8-A1JP	1.750684932	0	8.208498	8.162781	6.24897	4.952069	5.9913	0.892053249	low
TCGA-E2-A152	5.830136986	0	8.14969	6.209011	5.850612	4.345202	5.813968	1.108814311	high
TCGA-A2-A0YE	1.517808219	0	10.232492	4.32168	5.155699	5.298225	4.418159	1.623200921	high
TCGA-OL-A5S0	1.698630137	0	9.551788	4.060062	6.133565	8.708438	10.146258	1.374345243	high
TCGA-A2-A1FX	5.060273973	0	9.260061	8.240594	6.292941	5.675519	4.177754	0.913651459	low
TCGA-AC-A2FM	2.169863014	1	8.750312	5.263842	6.297873	9.046348	5.167089	1.00017646	high
TCGA-BH-A18H	1.78630137	0	7.541605	5.086008	5.998125	5.759488	5.633946	1.061307474	high
TCGA-3C-AALK	3.967123288	0	8.126609	6.376091	6.148812	5.823327	10.077252	1.098423064	high
TCGA-D8-A1JE	1.575342466	0	8.08311	9.141219	6.426306	4.22882	4.897234	0.796368593	low
TCGA-AR-A0TR	0.438356164	1	6.624627	7.674263	6.939622	5.622082	3.369826	0.664736673	low
TCGA-XX-A89A	1.336986301	0	7.838587	6.625197	6.188656	10.714487	7.377651	0.832974894	low
TCGA-OL-A5DA	4.884931507	0	7.550408	6.343434	6.63581	4.746762	5.285458	0.904063881	low
TCGA-E2-A1LK	0.728767123	1	9.302337	4.496299	5.692554	3.50354	12.43426	1.728807492	high
TCGA-A2-A4RW	0.608219178	0	7.722954	6.612568	6.277163	8.231739	5.11767	0.840139252	low
TCGA-OL-A5D8	2.665753425	0	8.219112	5.597483	7.088511	5.892467	5.809319	0.933586229	low
TCGA-BH-A0H7	1.923287671	0	8.704379	5.096878	5.449369	5.365843	11.504402	1.483759067	high
TCGA-EW-A1OW	1.901369863	0	9.480116	5.158678	6.739224	8.515696	8.483641	1.115730491	high
TCGA-AO-A0J4	4.347945205	0	8.941026	5.431202	5.432742	5.886121	10.494749	1.424284391	high
TCGA-C8-A12L	0.994520548	0	9.411741	4.402292	5.60765	3.852785	5.396448	1.500295659	high
TCGA-D8-A143	1.180821918	0	9.989748	4.964402	5.845402	5.709073	9.503452	1.516007991	high
TCGA-S3-A6ZF	1.567123288	0	9.108919	5.747135	6.954154	7.794427	4.402982	0.931684399	low
TCGA-AN-A0XV	0.443835616	0	7.829447	5.503188	6.441748	8.207032	4.242751	0.886504745	low
TCGA-B6-A401	7.112328767	0	8.187726	5.255159	5.87291	7.942226	7.250252	1.09474366	high
TCGA-EW-A1IX	3.309589041	0	5.902867	8.929229	6.921984	8.14813	4.758743	0.53533727	low
TCGA-AQ-A04H	2.065753425	0	9.059044	4.946125	6.641405	6.831448	6.200491	1.108749553	high
TCGA-D8-A13Z	1.739726027	0	9.486058	5.33559	6.174375	5.302385	10.887857	1.401410282	high
TCGA-BH-A28Q	3.065753425	0	6.659933	7.23322	7.15052	7.045024	4.630663	0.657527517	low
TCGA-EW-A1OX	2.495890411	0	7.337792	5.092249	6.632746	5.61679	3.87133	0.918197708	low
TCGA-AO-A03P	7.975342466	1	7.124411	5.702759	6.825082	3.758541	3.219345	0.87529602	low
TCGA-D8-A1XR	1.320547945	0	8.150948	7.061541	7.410212	8.68539	7.704855	0.753135336	low
TCGA-AN-A04A	0.246575342	0	7.901383	5.870639	5.477485	5.747331	5.501508	1.10995799	high
TCGA-AR-A0TU	1.942465753	0	10.341681	4.558837	6.652045	7.047699	10.128155	1.401286285	high
TCGA-BH-A18Q	4.635616438	1	9.198203	6.999296	6.599767	8.328041	5.208807	0.89728026	low
TCGA-BH-A0HB	2.208219178	0	9.565226	4.594939	5.86002	8.625395	5.662736	1.24280231	high
TCGA-BH-A0DL	6.523287671	0	8.786794	4.983373	5.310399	5.942421	10.897924	1.488717789	high
TCGA-A8-A08P	2.583561644	0	9.252181	4.828732	6.371011	6.793439	8.338795	1.24467	high
TCGA-GM-A2DK	7.246575342	0	9.350298	4.551236	5.90972	10.879263	6.141647	1.137633402	high
TCGA-AN-A04D	0.142465753	0	10.417135	4.334308	6.705716	4.814167	6.218148	1.404770028	high
TCGA-A2-A04V	5.260273973	1	8.465389	5.230309	6.123447	9.120184	3.227249	0.954400817	low
TCGA-GM-A2DA	18.0630137	1	8.089613	4.91335	6.099092	7.91925	7.081127	1.075215547	high
TCGA-AO-A126	9.060273973	0	8.33235	6.422117	6.594323	5.285034	4.238363	0.934836258	low
TCGA-E2-A159	2.087671233	0	9.533935	4.898667	6.649384	5.643803	11.052658	1.3516442	high
TCGA-E2-A15O	0.791780822	0	8.974612	5.467515	6.840474	7.830092	5.018299	0.967247184	low
TCGA-GM-A2DH	6.008219178	0	9.366835	7.10942	7.098528	6.229809	7.819672	0.959071083	low
TCGA-AC-A2FF	7.55890411	0	7.174457	7.450626	6.493891	9.772827	4.803235	0.683356643	low
TCGA-A2-A0ET	2.920547945	0	7.654999	7.693545	7.079721	7.617852	3.406517	0.673161215	low
TCGA-E9-A1N8	2.846575342	0	8.640586	5.223978	6.736115	3.51833	9.639095	1.2415899	high
TCGA-5T-A9QA	0.830136986	0	9.237353	4.007042	6.458802	3.995083	3.472305	1.285333029	high
TCGA-S3-AA15	1.438356164	0	7.759967	5.013227	5.121933	9.80364	5.829269	1.082813932	high
TCGA-EW-A6SA	1.397260274	0	9.057287	4.525412	6.919952	6.224898	5.192572	1.098108239	high
TCGA-A7-A4SE	1.764383562	0	10.284069	5.609721	6.111092	7.201429	8.280159	1.322809164	high
TCGA-BH-A1FC	9.512328767	1	9.913071	4.925374	6.346973	3.788772	10.20039	1.523088128	high
TCGA-A8-A07E	1.665753425	0	8.50074	7.267727	5.878663	8.873914	11.663016	1.034296232	high
TCGA-BH-A0E9	6.819178082	0	7.108859	7.019951	6.565151	8.059804	5.826833	0.753061745	low
TCGA-BH-A0HN	1.41369863	0	7.483313	7.508439	6.759315	3.516894	7.256632	0.878952546	low

TCGA-E9-A243	1.676712329	0	7.802022	5.446139	6.062852	4.394814	4.418097	1.067615799	high
TCGA-AR-A1AL	8.139726027	0	6.904186	7.75171	7.008235	8.958179	5.00763	0.62451569	low
TCGA-BH-A0HA	4.41369863	0	8.341931	6.604379	6.622736	7.461008	4.559552	0.861048784	low
TCGA-AR-A255	5.920547945	0	8.539118	6.902393	6.507082	8.855333	5.16983	0.843818389	low
TCGA-E9-A22G	3.394520548	0	9.499402	4.867811	5.780212	4.169789	9.607056	1.551133604	high
TCGA-AC-A62Y	1.452054795	0	7.801896	7.267506	6.634315	9.601638	3.503588	0.704889527	low
TCGA-D8-A1X6	1.482191781	0	7.84363	8.861739	6.982908	6.070555	6.137934	0.711301571	low
TCGA-B6-A409	1.569863014	1	9.633936	5.122469	5.603078	8.083044	12.173707	1.470248659	high
TCGA-A8-A08X	3.583561644	0	9.109135	4.85232	6.093692	4.24849	10.248773	1.448249126	high
TCGA-D8-A1XU	1.082191781	0	6.956455	8.409813	5.705838	5.528686	4.080153	0.786523845	low
TCGA-A2-A04T	6.153424658	0	9.332226	4.826133	5.61352	6.531084	10.113988	1.46603808	high
TCGA-OL-A66H	2.224657534	0	8.095895	6.31041	6.692829	6.320237	3.399715	0.861642604	low
TCGA-5L-AAT1	4.030136986	0	5.936415	6.920508	6.443898	6.38989	5.727138	0.727143149	low
TCGA-B6-A0RT	7.454794521	0	9.948986	5.738964	6.058741	8.194932	10.428681	1.297738194	high
TCGA-C8-A12N	0.980821918	0	7.170536	7.469536	7.060214	3.665846	5.385834	0.781896542	low
TCGA-E9-A228	3.520547945	0	9.013291	4.983615	6.92703	7.689533	3.227603	0.960402393	low
TCGA-PL-A8LZ	0.82739726	0	9.343823	5.709911	6.830667	9.811122	3.894718	0.899017207	low
TCGA-AO-A128	8.898630137	0	9.281791	5.262105	6.309826	4.795405	6.266649	1.24138111	high
TCGA-B6-A0I5	23.44109589	0	8.363344	5.669858	5.996964	6.216336	6.263755	1.097374917	high
TCGA-A2-A1FW	1.446575342	0	9.198451	5.30596	6.330066	8.60789	4.180245	1.028144324	high
TCGA-C8-A1HG	0.945205479	0	10.188324	4.368689	5.810227	6.650841	3.401719	1.371940754	high
TCGA-BH-A203	3.216438356	1	9.102768	4.95096	5.9264	9.02278	11.069382	1.278348927	high
TCGA-E9-A1RA	3.750684932	0	7.362713	6.683755	6.763519	9.180212	6.628141	0.755667007	low
TCGA-A1-A0SH	3.936986301	0	6.59424	8.642862	6.009888	4.289154	5.033733	0.761089438	low
TCGA-BH-A0WA	1.920547945	0	9.077484	5.163021	6.014818	5.122083	9.840568	1.371749562	high
TCGA-AO-A03L	6.690410959	0	9.611792	6.033986	5.927127	6.232648	11.336072	1.363063382	high
TCGA-A2-A04N	11.92876712	0	7.254487	7.626481	6.725267	5.742692	6.351852	0.77880927	low
TCGA-A2-A1G0	1.687671233	0	6.057031	7.807706	6.983835	8.971516	4.219616	0.564562174	low
TCGA-E2-A105	3.583561644	0	9.479048	4.845002	6.95135	8.867676	7.789459	1.07925868	high
TCGA-D8-A3Z6	1.542465753	0	6.895581	6.3119	6.647124	5.956761	8.14417	0.870722653	low
TCGA-E2-A14T	6.331506849	0	8.360155	4.462189	6.258311	10.097055	4.384597	0.977586357	low
TCGA-A8-A095	3.498630137	0	8.630576	6.424099	6.910761	9.000003	6.351916	0.853107979	low
TCGA-A8-A092	2.580821918	0	9.650364	4.680146	6.434564	6.330743	7.528206	1.292731226	high
TCGA-E9-A5FK	2.224657534	0	7.205346	5.567046	5.60806	9.774041	6.456712	0.932452654	low
TCGA-C8-A137	1.038356164	0	9.132972	4.974015	5.992603	5.431721	4.698448	1.23617318	high
TCGA-E9-A1RB	2.673972603	1	9.597483	4.583572	6.342967	4.901878	3.39723	1.253156478	high
TCGA-AR-A2LQ	3.378082192	0	6.491984	5.736873	5.783032	9.949943	10.243541	0.908202297	low
TCGA-LL-A5YL	1.421917808	0	8.325163	6.262782	5.925322	7.84161	7.712481	1.032934162	high
TCGA-E9-A3Q9	2.742465753	0	4.950079	8.000929	6.268302	10.567573	3.169764	0.511963056	low
TCGA-A2-A0T3	4.153424658	0	8.860553	5.545132	6.462547	7.834645	5.753119	1.019682493	high
TCGA-D8-A27G	1.120547945	0	7.950632	6.029643	6.289007	8.148879	4.635933	0.88952762	low
TCGA-A8-A07P	0.915068493	0	7.905402	6.969189	6.078504	7.063247	8.227033	0.954428892	low
TCGA-D8-A1JN	1.698630137	0	6.269432	6.866501	6.323235	6.962889	11.84427	0.864676749	low
TCGA-A8-A08O	2.583561644	0	8.186633	6.257625	7.104056	8.896136	5.987218	0.801812547	low
TCGA-AR-A24M	10.02739726	0	6.979711	8.824837	6.872843	5.889959	6.960097	0.683233631	low
TCGA-E9-A1NF	2.936986301	1	8.703232	7.16917	5.784215	5.11872	9.69081	1.166754347	high
TCGA-AR-A0TS	7.008219178	0	9.122288	7.231979	5.987332	9.22288	9.726886	1.025833896	high
TCGA-A2-A1G4	1.630136986	0	8.118357	6.266944	6.863508	7.676621	4.153003	0.822486296	low
TCGA-A2-A3KD	3.304109589	0	7.590722	7.161246	6.244903	7.451688	6.746026	0.850582344	low
TCGA-B6-A0I1	6.468493151	1	9.265028	3.975946	6.021928	3.800047	9.086112	1.570959103	high
TCGA-B6-A0RP	8.564383562	1	6.766355	8.079317	6.617667	8.069473	5.547832	0.661975832	low
TCGA-AO-A12G	4.490410959	0	7.218483	6.681961	5.802544	6.169789	3.819003	0.884907088	low
TCGA-EW-A1OV	2.161643836	0	8.392183	5.490423	5.93867	7.183182	7.765695	1.127217918	high
TCGA-GM-A2DI	7.095890411	0	7.507232	6.150777	6.401133	7.780434	6.409814	0.875981492	low
TCGA-BH-A8FY	0.808219178	1	8.236684	7.905523	6.512318	6.335104	3.624521	0.795716063	low
TCGA-A2-A0EP	9.871232877	0	7.3857	6.216295	5.590341	10.990603	10.526114	0.952957143	low
TCGA-EW-A423	1.460273973	0	8.369157	6.868566	5.689948	6.088791	8.381087	1.101300016	high
TCGA-E9-A1R0	2.356164384	0	8.055948	6.429809	6.457279	11.240691	5.683225	0.786199944	low

TCGA-C8-A8HQ	1.04109589	0	9.427147	5.207206	6.359538	7.353441	9.528849	1.241775531	high
TCGA-A7-A5ZV	1.008219178	0	9.08629	6.336055	4.834321	4.830246	3.629369	1.298467565	high
TCGA-E2-A10A	3.367123288	0	8.690878	6.361489	6.946339	6.833211	3.832344	0.870724915	low
TCGA-AO-A03M	5.112328767	0	9.81034	5.839897	6.344463	6.572749	6.363725	1.174425953	high
TCGA-A8-A06Q	0.084931507	0	8.154966	4.416471	5.977769	4.472292	3.784855	1.190077781	high
TCGA-E9-A54X	1.991780822	0	8.383283	4.998614	6.548373	5.13519	5.8091	1.099260477	high
TCGA-S3-AA17	1.161643836	0	8.751671	5.040539	5.773942	5.354146	6.860744	1.287411693	high
TCGA-A8-A09T	1.58630137	0	7.257747	7.965718	7.303989	4.340109	7.399691	0.750547364	low
TCGA-A7-A56D	1.22739726	0	8.418131	4.462065	5.374416	4.038906	10.482781	1.564372059	high
TCGA-BH-A0B5	5.852054795	0	8.55436	5.700254	6.665965	8.634239	5.059034	0.911161787	low
TCGA-A2-A0D2	2.81369863	0	10.319754	4.667427	5.762403	6.247798	7.667168	1.526368453	high
TCGA-A2-A0D3	5.131506849	0	5.425025	6.115456	7.318413	5.848233	9.425601	0.721159883	low
TCGA-E2-A15K	0.753424658	0	8.522573	6.684568	6.692457	6.590173	9.473702	0.992455356	low
TCGA-AC-A8OQ	0.093150685	0	9.15454	4.906119	5.339154	9.698253	9.475011	1.32056448	high
TCGA-LL-A5YM	1.079452055	0	8.827087	4.379141	5.626476	10.845378	3.62001	1.078444251	high
TCGA-AO-A0JG	2.18630137	0	5.879695	6.048815	6.36443	7.529802	4.959772	0.740140022	low
TCGA-A2-A0YF	4.205479452	0	8.367475	8.996343	6.715246	8.532015	6.580535	0.715599083	low
TCGA-AN-A0AL	0.621917808	0	9.368111	4.804067	5.183009	5.308422	3.624657	1.407156555	high
TCGA-D8-A1XM	1.473972603	0	8.096347	7.449113	6.978268	10.447257	4.46763	0.677044993	low
TCGA-BH-A0DT	6.583561644	0	6.903107	6.907966	7.033518	8.336006	4.713866	0.673509192	low
TCGA-AR-A2LK	4.517808219	1	8.915521	5.722843	6.590101	6.148196	4.53529	1.021736141	high
TCGA-C8-A12M	0.980821918	0	7.820767	4.454818	6.907273	6.082514	3.276474	0.944395303	low
TCGA-D8-A1XD	1.430136986	0	6.986753	6.700892	6.455506	3.509448	5.275928	0.890040175	low
TCGA-A8-A09W	0.082191781	0	8.900219	5.791818	5.563089	6.935566	9.25479	1.271808965	high
TCGA-D8-A1XF	1.268493151	0	9.169613	4.426226	6.790633	5.934287	3.429997	1.105072207	high
TCGA-D8-A73U	1.347945205	0	5.321739	6.660013	5.68797	5.99754	11.695727	0.902476655	low
TCGA-EW-A1J6	2.397260274	0	8.930232	5.017796	6.078899	7.054216	3.491499	1.100264231	high
TCGA-AO-A0J6	3.123287671	0	9.11366	4.485971	5.805177	3.440653	4.519755	1.39991669	high
TCGA-BH-A0H0	1.263013699	0	8.506596	5.652738	6.260934	8.927347	3.372863	0.918534429	low
TCGA-AC-A3YJ	2.065753425	0	7.306309	8.734712	6.88326	5.293837	7.239207	0.72777352	low
TCGA-D8-A1XB	1.512328767	0	7.286327	6.994384	7.033144	4.348329	6.967808	0.83435426	low
TCGA-BH-A0C3	7.421917808	0	7.87124	5.719125	5.847562	11.307299	4.931978	0.872100548	low
TCGA-OL-A66K	3.493150685	1	6.898613	7.066935	7.265658	5.924863	5.268547	0.706181529	low
TCGA-B6-A0X4	2.356164384	1	6.318928	6.432125	7.190548	3.735384	3.279238	0.728653861	low
TCGA-BH-A0BJ	1.808219178	0	6.973298	7.477175	6.80285	8.327688	4.782958	0.671585903	low
TCGA-A1-A0SF	4.008219178	0	7.894278	4.711499	6.357917	7.961758	5.400109	0.993226699	low
TCGA-LD-A7W6	1.106849315	0	7.893928	6.484424	6.781928	8.516419	4.950405	0.792804799	low
TCGA-E2-A574	3.230136986	0	11.575318	3.829902	5.861702	5.486406	11.44965	2.025750285	high
TCGA-D8-A141	1.715068493	0	7.227519	7.284147	6.908426	8.038177	4.4296	0.689378954	low
TCGA-AO-A12H	3.380821918	0	7.527708	7.518158	6.674123	3.916222	3.226398	0.80383948	low
TCGA-D8-A1XW	3.58630137	0	7.165499	8.671742	6.446593	3.467915	8.673711	0.842276988	low
TCGA-A2-A0SW	3.739726027	1	9.087976	4.399624	4.857608	8.339903	10.926022	1.578928963	high
TCGA-A2-A0EX	2.060273973	0	5.995719	7.098178	6.216119	5.938795	4.185744	0.730098578	low
TCGA-A7-A26F	2.021917808	0	6.699155	4.954901	3.933111	4.343812	7.189523	1.435806479	high
TCGA-C8-A273	1.405479452	0	8.622602	5.941177	5.66804	6.268386	4.663749	1.110942245	high
TCGA-AN-A0FT	0.58630137	0	7.886551	4.939078	5.801132	7.223709	6.660415	1.112375171	high
TCGA-AR-A1AU	7.857534247	0	8.068258	5.086343	6.445165	9.945774	6.404678	0.928285217	low
TCGA-A7-A6VW	0.780821918	0	9.812776	4.038806	5.988287	6.981446	9.501045	1.503836549	high
TCGA-AR-A2LM	5.301369863	0	6.102344	7.93811	6.961751	7.503346	5.109612	0.603354703	low
TCGA-AR-A24N	8.315068493	0	7.822789	5.652827	7.606475	5.782958	6.642015	0.850767867	low
TCGA-AR-A0TT	9.084931507	0	8.395442	5.932025	6.767321	7.845073	4.495547	0.880644091	low
TCGA-AN-A0AJ	0.830136986	0	10.463144	4.478523	6.332793	7.196735	4.740171	1.313752916	high
TCGA-E9-A1RC	3.353424658	0	8.019357	5.05119	6.790804	7.349303	7.790082	0.992745604	low
TCGA-S3-AA11	1.153424658	0	8.203008	5.940504	7.11265	7.432922	6.936491	0.881803728	low
TCGA-B6-A0I2	11.94794521	0	9.049436	4.380499	6.353788	3.765316	4.004749	1.269449419	high
TCGA-GM-A2D9	4.964383562	1	7.460371	6.434447	6.269896	8.300376	8.734068	0.900479621	low
TCGA-AN-A0FL	0.632876712	0	8.225429	5.040656	4.901864	4.563419	5.171943	1.367046617	high
TCGA-AO-A1KQ	5.156164384	0	10.186961	4.72061	6.351093	6.986352	7.870042	1.354046967	high

TCGA-EW-A6SD	2.767123288	0	8.459255	5.044524	5.299769	3.896107	8.249614	1.44914664	high
TCGA-E2-A570	2.550684932	0	8.52019	6.534674	6.95211	6.705456	4.925214	0.86936176	low
TCGA-AC-A5EH	1.4	0	8.522297	7.37208	4.218839	4.883895	10.853341	1.45622934	high
TCGA-A2-A0T0	1.460273973	0	9.136784	4.574385	5.549926	3.437846	12.333248	1.725329586	high
TCGA-AR-A0TW	8.243835616	0	7.127032	4.220658	5.962506	3.873288	8.909547	1.256774919	high
TCGA-D8-A1JD	1.512328767	0	8.920112	6.086205	6.630541	3.761436	3.840537	1.053126812	high
TCGA-E9-A1RD	0.093150685	0	7.346266	7.161347	7.375456	4.454651	3.970109	0.734847097	low
TCGA-BH-A18R	3.128767123	1	9.041651	6.021292	6.489028	6.690035	4.889066	1.014981429	high
TCGA-A2-A25F	0.882191781	0	9.138411	6.716777	5.82278	10.535368	3.31508	0.905462679	low
TCGA-BH-A0BO	6.019178082	0	5.336507	5.927666	6.416	9.980686	5.263602	0.653248043	low
TCGA-GM-A2DB	6.591780822	0	8.56458	4.679805	4.757695	5.114563	11.675417	1.687075471	high
TCGA-A2-A0CO	4.021917808	0	5.810048	6.079432	5.975728	10.253323	10.035777	0.794167144	low
TCGA-BH-A2L8	1.676712329	0	7.297261	5.610456	7.230591	8.98659	6.314597	0.763348197	low
